# Transporter Genes and fosA Associated With Fosfomycin Resistance in Carbapenem-Resistant *Klebsiella pneumoniae*

**DOI:** 10.3389/fmicb.2022.816806

**Published:** 2022-01-31

**Authors:** Yu-Ping Wang, Yen-Hao Chen, I-Cheng Hung, Po-Hsun Chu, Yu-Han Chang, Yi-Tsung Lin, Hung-Chih Yang, Jin-Town Wang

**Affiliations:** ^1^Department of Microbiology, National Taiwan University College of Medicine, Taipei, Taiwan; ^2^Division of Infectious Diseases, Department of Internal Medicine, Fu Jen Catholic University Hospital, New Taipei City, Taiwan; ^3^Division of Infectious Diseases, Department of Medicine, Taipei Veterans General Hospital, Taipei, Taiwan; ^4^Department of Internal Medicine, National Taiwan University Hospital, Taipei, Taiwan

**Keywords:** *Klebsiella pneumoniae*, carbapenem resistance enterobacteriaceae, fosfomycin resistance mechanism, fosfomycin resistance gene, glpT and uhpT transporters

## Abstract

Infections caused by carbapenem-resistant *Klebsiella pneumoniae* (CRKP) are of significant clinical concern worldwide. Fosfomycin is one of the limited treatment options for CRKP. However, resistance to fosfomycin in CRKP has been observed. In this study, we aimed to investigate the fosfomycin resistance mechanism of CRKP. Fosfomycin-resistant *Klebsiella pneumoniae* isolates were collected from four medical centers in Taiwan from 2010 to 2018. The genes that contributed to fosfomycin resistance were amplified and sequenced. Carbohydrate utilization assays and mutagenesis studies were performed to determine the mechanisms underlying fosfomycin resistance. Forty fosfomycin-resistant CRKP strains were collected and used for further analysis. Fourteen strains exhibited low-level resistance (MIC = 256–512 mg/dl), while 26 strains showed high-level resistance (MIC ≥ 1,024 mg/dl). Chromosomal fosA^KP^ I91V was detected in 39/40 fosfomycin-resistant CRKP strains. We observed that amino acid substitution of chromosomal fosA^KP^ I91V increased the MIC of fosfomycin by approximately eight folds, and this was the only mechanism elucidated for low-level fosfomycin resistance. Among the 26 high-level resistance strains, fosA^KP^ I91V combined with transporter deficiencies (18/26, 69.2%) was the most common resistant mechanism, and one strain showed transporter deficiency only. Plasmid-borne fosA3 accounted for 27.0% (7/26) of high-level resistance. Various G3P and G6P transporter gene mutations, including three novel single amino acid mutations (glpT E299D, glpT D274V, and uhpC A393V) were detected in 19 strains. No murA mutation was found in this study. Our study highlights the need for new therapeutic agents for CRKP infections in Taiwan.

## Introduction

The emergence of multidrug-resistant bacteria has raised great clinical concern worldwide, and *Klebsiella pneumoniae* is one of the most important pathogens that cause several types of healthcare-associated infections (Podschun and Ullmann, 1998; Juan et al., 2019; Nordmann and Poirel, 2019). Carbapenems are considered as the last resort of treatment of multidrug-resistant *K. pneumoniae*, but an increasing prevalence of carbapenem-resistant *K. pneumoniae* (CRKP) has been recorded worldwide (Chiu et al., 2013; Logan and Weinstein, 2017).

Fosfomycin is one of the limited treatment options for CRKP (Morrill et al., 2015). Fosfomycin is a phosphonic acid antibiotic discovered in a fermentation broth of *Streptomyces fradiae* in Spain (Gadebusch et al., 1992). It inhibits N-acetylglucosamine enolpyruvyl transferase (murA), which is an essential enzyme in the early stage of cell wall synthesis. In previous studies, fosfomycin was found to be active against most CRKP, with Clinical and Laboratory Standards Institute (CLSI) breakpoints, (Endimiani et al., 2010; Falagas et al., 2010) and several clinical studies have reported its promising efficacy and low toxicity (Michalopoulos et al., 2010; Pontikis et al., 2014). However, fosfomycin-resistant CRKP has been observed, and is a problem in clinical settings (Yang et al., 2019).

Currently, several mechanisms of resistance have been reported. First, mutation in murA reduces the affinity of fosfomycin (Kumar et al., 2009). Second, fosfomycin enters the cell through two different uptake pathways: the glycerol-3-phosphate (G3P) and the glucose-6-phosphate (G6P) transporter systems. Deficiency of these transporters decreases fosfomycin uptake, leading to resistance (Takahata et al., 2010). Third, three major types of fosfomycin-modifying enzymes, namely fosA, fosB, and fosX, were described to contribute to resistance. Glutathione S-transferase (GST) homologs are most commonly found in gram-negative bacteria, including *K. pneumoniae*, and they reduce fosfomycin susceptibility (Ito et al., 2017). Several subtypes of plasmid-mediated fosA homologs were reported to confer high-level resistance (Yang et al., 2019).

In this study, we aimed to evaluate the mechanisms of fosfomycin resistance in CRKP strains in Taiwan. We analyzed the capsular type distribution of fosfomycin-resistant CRKP and attempted to clarify the resistance mechanism. Chromosomal mutagenesis, plasmid transformation, and sole carbohydrate growth assays were performed to confirm that the phenotypic changes were caused by genetic changes.

## Materials and Methods

### Clinical Strains

Clinical fosfomycin-resistant CRKP strains which caused infections were retrospectively collected from the National Taiwan University Hospital, Taipei Veterans General Hospital, National Cheng Kung University Hospital, and Linkou Chang Gung Memorial Hospital from January 2010 to August 2018. If multiple strains were isolated from a same patient, only one strain will be enrolled. CRKP was defined as strains with a minimum inhibitory concentration (MIC) of ≥4 mg/L for imipenem or meropenem, based on the CLSI guidelines.

### Antimicrobial Susceptibility Testing

Following the CLSI guidelines, the MICs of fosfomycin (Sigma-Aldrich, St. Louis, MO, United States) were determined by the agar dilution method using Mueller-Hinton agar plates (BD, France) supplemented with 25 mg/L G6P (Sigma-Aldrich). *Escherichia coli* ATCC 25922 was used as quality control for antimicrobial susceptibility testing. High-level fosfomycin-resistant *K. pneumoniae* strains were defined as those with MIC ≥ 1,024 mg/dL.

### Sequence Analysis of Capsular Types, Fosfomycin-Related Genes, Carbapenemase Genes, and Multilocus Sequence Typing (MLST)

The capsular types of the CRKP strains were determined using *wzc* genotyping and whole sequencing, as previously reported (Pan et al., 2015). Enzymatic mediated fosfomycin resistance genes (*fosA3, fosB, fosC, fosX*, *fomA*, and *fomB*), and fosfomycin-related genes (*murA*, *glpT*, *glpR*, *uhpT*, *uhpA*, *uhpB*, *uhpC*, *ptsI*, and *cyaA*) were amplified and sequenced; the primers used are listed in Supplementary Table 1. Two fosfomycin-susceptible strains, NTUH-K2044 (Fang et al., 2004) (GenBank: AP006725.1) and MGH 78578 (GenBank: CP000647.1) were used as reference strains to identify amino acid substitutions, excluding polymorphisms, observed in the fosfomycin-susceptible strains. Most common carbapenemase genes (*bla*_*KPC*_, *bla*_*NDM*_, *bla*_*IMP*_, *bla*_*VIM*_, *bla*_*GES*_, *bla*_*OXA–*23–like_, *bla*_*OXA–*48–like_) were amplified for the presence of carbapenemase. If the carbapenemase genes were not detected by PCR method, The Simplified Carbapenem Inactivation Method (Jing et al., 2018) was performed to identify carbapenemase production. Sequence types (STs) were determined by sequence alignment using the Pasteur Institute database. Next generation sequencing was performed for strains in which the genes could not be amplified with the various primer pairs. The genomes were sequenced using the Pacific Biosciences RS II platform (Menlo Park, CA, United States). Assembly of the data was performed using the hierarchical genome assembly process (HGAP) compiled specifically for quality trimming, *de novo* assembly, and polishing of PacBio data.

### Plasmid Transformation

Nucleobond PC100 Plasmid Midiprep (Takara Bio, Mountain View, CA, United States) was used to purify the plasmid of *fosA3*-positive strains and controls. Purified plasmids were transformed into recipient *E. coli* DH10B cells through electroporation under the conditions of 2,000 V/200 Ω/25 μF. After 1 h of recovery, the *E. coli* cells were cultured on plates with 16 mg/dL fosfomycin. Insertion of *fosA3* in the *E. coli* cells that grew on the plates was confirmed using PCR amplification.

### Sole Carbohydrates Growth Assay

Utilization of carbohydrates, which indicated the activity of glpT and uhpT transporters, was performed as previously described (Tseng et al., 2015). Briefly, 0.2% (w/v) G6P or G3P was supplied in M9 minimal medium agar as the sole carbon source. Briefly, M9 minimal medium agar was supplemented with 0.2% (w/v) G6P or G3P as the sole carbon source. Overnight bacterial suspensions were washed with an equivalent volume of saline and McFarland No. 4. The suspension was diluted 10-fold with physiological saline, thoroughly mixed, plated (2 μL) on M9 minimal medium agar supplemented with different sole carbohydrates, and then incubated at 37°C for 48 h. A negative phenotype was defined as a lack of colonies on the plate.

### Site-Directed Mutagenesis

Site-directed mutations were generated in the fosfomycin-susceptible strain NTUH-K2044 (MIC = 32) using the pKO3-km plasmid (Link et al., 1997). The DNA fragments, including point mutation sites and flanking regions (glpT, uhpA, uhpC, and fosA^KP^), were amplified using PCR (primers are listed in Supplementary Table 1) and then cloned into the pKO3-km plasmid. The resulting plasmids were used to generate point mutants as in a previous study (Lin et al., 2012), and the mutants were confirmed by sequencing.

### Analysis of Chromosomal fosA

We named the chromosomal fosA in the genomes of *K. pneumoniae* as fosA^KP^, as previously described (Ito et al., 2017). Because no previous reported mechanism was detected in 14 low level resistant strains, sequences and mRNA expression level of fosA^KP^ were analyzed in this study. We designed primer pairs to distinguish fosA^KP^ from the plasmid fosA3 (Supplementary Table 1). There are 13 nucleotides differences between primer pairs of chromosomal fosA and fosA3. Furthermore, we also check surrounding elements of fosA3 (Primer pairs: IS and fosA3) and chromosomal fosA (Primer pairs: FosA frk) to confirm the fosA3 is located on plasmid. Then, fosA^KP^ was amplified, sequenced, and compared with the reference strains.

## Results

In total, 108 CRKP strains were collected in our study. Antibiotic susceptibility test revealed that 54 strains (50.0%) were susceptible to fosfomycin, 14 strains (13.0%) exhibited intermediate resistance, and 40 strains (37.0%) were resistant to fosfomycin. Among the 40 fosfomycin-resistant CRKP strains, 26 exhibited high levels of fosfomycin resistance (MIC > 1,024 mg/dl), while 14 showed low levels of fosfomycin resistance (MIC between 256 and 512 mg/dl) (Table 1). As presented in Table 1, capsular typing results showed that K47 was the most dominant capsular type (52.5%, 21/40), followed by K64 (27.5%, 11/40) and K62 (7.5%, 3/25). Capsular types K5, K15, K24, K54, and KN2 were each identified in one strain (2.5%, 1/40). KPC was detected in 21 of 40 fosfomycin-resistant CRKP strains, and the remaining 19 strains fosfomycin-resistant CRKP strains had no carbapenemase detected by both genotype and phenotype assay.

**TABLE 1:** Characteristics and detected mechanisms of fosfomycin-resistant CRKPs and reference strains.

Strain	Specimen	Capsular type	MLST	Carbapenemase genes	MIC (mg/dl)	fosA enzyme		G3P transporter system			G6P transporter system						
	
						fosA^KP^	fosA3		glpT	glpR		uhpA		uhpB	uhpC		uhpT
NTUH-2044		K1	ST23		32	I91											
MGH 78578		K52	ST38		32	I91											
Low level resistance																	
FO01	Urine	K5	ST76		256	I91V											
FO02	Blood	K47	ST11	KPC-2	512	I91V											
FO03	Sputum	K47	ST11	KPC-2	256	I91V											
FO04	Blood	K47	ST11	KPC-2	256	I91V											
FO05	Blood	K47	ST11	KPC-2	256	I91V											
FO06	Sputum	K47	ST11	KPC-2	256	I91V											
FO07	Pus	K47	ST11	KPC-2	256	I91V											
FO08	Blood	K54	ST29		256	I91V											
FO09	Blood	K64	ST11		512	I91V											
FO10	Blood	K64	ST11		512	I91V											
FO11	Blood	K64	ST11		256	I91V											
FO12	Blood	K64	ST11		256	I91V											
FO13	Blood	K64	ST11		256	I91V											
FO14	Blood	KN2	ST11		512	I91V											
High level resistance																	
FO15	Pus	K47	ST11	KPC-2	>2048	I91V	+										
FO16	Blood	K47	ST11	KPC-2	>2048	I91V	+										
FO17	Urine	K47	ST11	KPC-2	>2048	I91V	+										
FO18	Urine	K47	ST11	KPC-2	>2048	I91V	+										
FO19	Urine	K47	ST11	KPC-2	>2048	I91V	+										
FO20	Pus	K47	ST11	KPC-2	>2048	I91V	+										
FO21	Blood	K64	ST11		>2048	I91V	+										
FO22	Blood	K15	ST11		>2048	I91V		IS^$^								*IS* ^%^	
FO23	Blood	K24	ST15		>2048	I91		ES276									
FO24	Blood	K47	ST11	KPC-2	2048	I91V							A393V				
FO25	Blood	K47	ST11	KPC-2	>2048	I91V		LF^@^					ES446				
FO26	Urine	K47	ST11	KPC-2	>2048	I91V		LF^#^			D.		D.		D.	D.	
FO27	Sputum	K47	ST11	KPC-2	>2048	I91V		D274V								ES69	
FO28	Sputum	K47	ST11	KPC-2	>2048	I91V		D274V								ES69	
FO29	Sputum	K47	ST11	KPC-2	>2048	I91V		D274V								ES69	
FO30	Sputum	K47	ST11	KPC-2	>2048	I91V		D274V								ES69	
FO31	Sputum	K47	ST11	KPC-2	>2048	I91V		D274V								ES69	
FO32	Sputum	K47	ST11	KPC-2	>2048	I91V		D274V								ES69	
FO33	Sputum	K62	ST378		>2048	I91V		ES293									
FO34	Sputum	K62	ST378		>2048	I91V		ES293									
FO35	Sputum	K62	ST378		>2048	I91V		ES293									
FO36	Urine	K64	ST11		>2048	I91V		ES290									
FO37	Blood	K64	ST11		>2048	I91V		E299D									
FO38	Urine	K64	ST11		>2048	I91V		E299D									
FO39	Urine	K64	ST11		>2048	I91V					ES86						
FO40	Urine	K64	ST11		>2048	I91V					D.		D.		D.	D.	

*MIC, Minimal inhibitory concentration; G3P, glycerol-3-phosphate; G6P, glucose-6-phosphate; ES, early stop condon; D., Deletion; fosA3, Plasmid-borne fosA3; fosA^KP^, Chromosomal fosA. ^$^: insertion sequence, IS4-like in glpT coding region (+865). ^%^: insertion sequence, IS4-like in uhpT coding region (+ 287). ^@^: Loss of fragment, 1,160th–1,172th nucleotide deletion. ^#^: Loss of fragment, 388th–420th nucleotide deletion.*

**FIGURE 1:**
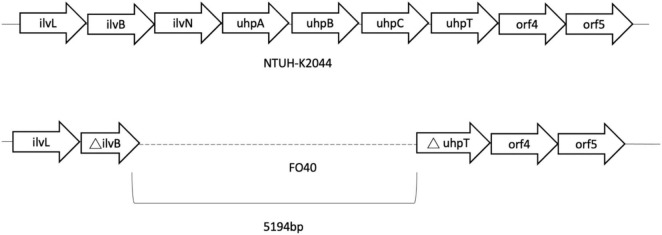
Flanking region of uhpABCT operon of NTUH-2044 and FO40.

**FIGURE 2:**
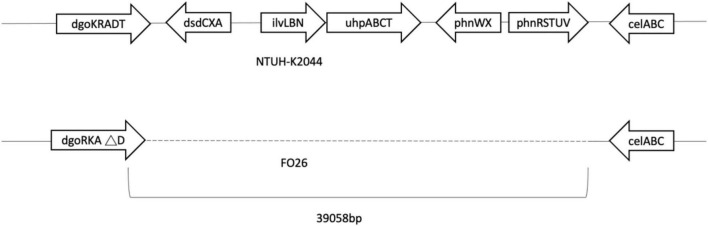
Flanking region of uhpABCT operon of NTUH-2044 and FO26.

Genes of fosfomycin-targeting enzymes and transporters were examined using PCR amplification and sequencing (Table 1). We did not find any sequence changes or amino acid variations in *murA* in all 40 fosfomycin-resistant strains, while transporter gene mutations were observed in 19 strains. As growth impairment using a sole carbohydrate source among the 19 strains indicated the loss of function of transporter genes, we performed a growth assay using G3P and G6P to check the function of these transporters. All 19 strains had growth impairment on either G3P or G6P minimal medium agar, which was compatible with gene mutations (Table 2). In particular, eight strains were not able to grow on G3P minimal medium agar only, three isolates could not grow on G6P minimal medium agar only, and growth impairment was observed on both G3P and G6P minimal medium agar in eight strains. In the 15 strains that showed growth impairment on G3P minimal medium agar, an early stop codon was found in glpT of 5 strains, and amino acid substitutions in glpT (299D, 274V) were detected in 6 strains. Loss of small nucleotide fragment in glpT in 2 strains and an insertion sequence added in the glpT coding region in one strain were also detected. Mutations in glpR were not detected in this study. Among 11 isolates with growth impairment on G6P minimal medium agar, an early stop codon was detected in uhpA and uhpT in one strain and six strains, respectively. Amino acid substitution was detected in uhpB (393V) in one strain. We were not able to amplify *uhpA*, *uhpB*, *uhpC*, and *uhpT* in two strains (FO26, FO40) using the primer pairs. Therefore, we designed primer pairs for the outer area of the uhpABCT operon to amplify and sequence the fragments. Results showed that FO40 had lost a 5,154 bp-long fragment, including most of the uhpABCT operon and ilvNBL operon (Figure 1). Because we were unable to find suitable primer pairs, next generation sequencing revealed that FO26 (GenBank accession: JAHZSM000000000) had lost a large fragment (39,058 bp), including the entire uhpABCT operon (Figure 2).

**TABLE 2:** Deficiency in G3P and G6P transport systems and consequent carbohydrate utilization among the fosfomycin high-level resistant CRKP strains.

Strain	MIC	G3P growth	G3P system deficiency	G6P growth	G6P system deficiency
NTUH-2044	32	+		+	
FO15	>2,048	+		+	
FO16	>2,048	+		+	
FO17	>2,048	+		+	
FO18	>2,048	+		+	
FO19	>2,048	+		+	
FO20	>2,048	+		+	
FO23	>2,048	−	glpT ES276	+	
FO33	>2,048	−	glpT ES293	+	
FO34	>2,048	−	glpT ES293	+	
FO35	>2,048	−	glpT ES293	+	
FO36	>2,048	–	glpT ES290	+	
FO37	>2,048	−	glpT E299D	+	
FO38	>2,048	–	glpT E299D	+	
FO24	2,048	+		−	uhpB 393V
FO39	>2,048	+		−	uhpA ES86
FO40	>2,048	+		−	Loss of uhpA, ubpB, uhpC, uhpT
FO22	>2,048	−	*IS4-like in glpT coding region*	−	*IS4-like in uhpT coding region*
FO25	>2,048	−	glpT 1,160th–1,172th nucleotide loss	+	uhpB ES446
FO26	>2,048	−	glpT 388th–420th nucleotide loss	−	Loss of uhpA, ubpB, uhpC, uhpT
FO27	>2,048	−	glpT 274V	−	uhpT ES69
FO28	>2,048	−	glpT 274V	−	uhpT ES69
FO29	>2,048	−	glpT 274V	−	uhpT ES69
FO30	>2,048	−	glpT 274V	−	uhpT ES69
FO31	>2,048	−	glpT 274V	−	uhpT ES69
FO32	>2,048	−	glpT 274V	−	uhpT ES69

*MIC, Minimal inhibitory concentration; G3P, glycerol-3-phosphate; G6P, glucose-6-phosphate; ES, early stop codon; (+), growth; (−), no growth.*

To confirm whether the transporter-related point mutations contribute to fosfomycin resistance, we performed site-directed mutagenesis. After the mutations were generated in NTUH-2044, the MICs of fosfomycin increased by 32-fold (1,024 mg/dL) (Table 3). Moreover, the NTUH-2004 mutants did not grow on either G3P or G6P minimal medium agar plates. These phenotypic changes were comparable with the gene mutations generated.

**TABLE 3:** Minimum inhibitory concentration of fosfomycin in site-directed mutants of NTUH-K2044.

Strain	MIC to fosfomycin	G3P growth	G6P growth
NTUH-K2044			
Wild type	32	+	+
glpT 299D	>1,024	−	+
glpT 274V	>1,024	−	+
uhpA ES 86	1,024	+	−
uhpC 393V	1,024	+	−
fosA I91V	256	n/a	n/a

*MIC, Minimal inhibitory concentration; G3P, glycerol-3-phosphate; G6P, glucose-6-phosphate; (+), growth; (−), no growth; n/a, not available.*

**FIGURE 3:**
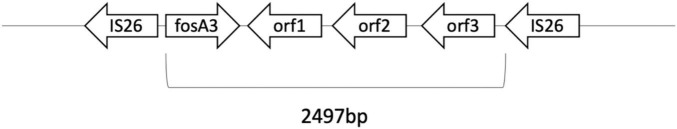
Flanking region of fosA3 of FO15.

Plasmid-mediated *fosA3* was detected in seven isolates (7/40, 24%) (Table 4). The flanking regions of the seven isolates were observed to be identical (Figure 3). Fragments with 2497 bp in total, which contained *fosA3*, *orf1*, *orf2*, and a truncated *orf3*, was located between two IS26 elements. Next generation sequencing was performed for FO15 (Genbank accession: CP073002) to read the plasmids. To further confirm that the fosA3 genes were located on the plasmid, we performed plasmid transformation study (Table 3). All *fosA3* detected in the seven strains was successfully transformed into *E. coli* DH10B, which became resistant to fosfomycin. Three other randomly selected fosfomycin-resistant strains and NTUH-2044 were used to perform the same plasmid transformation procedures. The recipient *E. coli* DH10B did not grow on the fosfomycin plate after plasmid transformation.

**TABLE 4:** Plasmid transformation result for fosA3-carrying strains.

	Detected fosA gene		Growth on fosfomycin plate after plasmid transformation
	fosA	fosA3	
NTUH-2044	+		
FO15	+	+	+
FO16	+	+	+
FO17	+	+	+
FO18	+	+	+
FO19	+	+	+
FO20	+	+	+
FO21	+	+	+
FO22	+		
FO26	+		
FO38	+		

**FIGURE 4:**
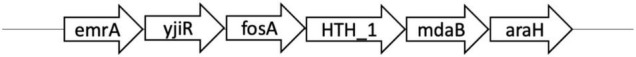
Flanking region of fosA.

Notably, mutations in *murA* or transporter gene or plasmid-mediated *fosA3* were not detected in low-level fosfomycin-resistant strains (Table 1). Therefore, we examined the chromosomal fosA^KP^. The flanking regions of chromosomal fosA^KP^ was shown on Figure 4. As fosA^KP^ was detected in both fosfomycin-susceptible and fosfomycin-resistant strains, we also analyzed the gene sequence and mRNA expression levels of fosA^KP^ in fosfomycin low-level resistant CRKP strains. Chromosomal fosA^KP^ was found in all fosfomycin-resistant strains (40/40, 100%), and no significant difference in fosA^KP^ mRNA expression between fosfomycin-resistant and fosfomycin-resistant strains was observed (Supplementary Figure 1). Compared with the reference strains (NTUH-2044, MGH 78578), mutation I91V was found in 39/40 (97.5%) fosfomycin-resistant CRKP. All 14 low-level resistant strains had I91V mutation in fosA^KP^. A site-directed mutation study was performed to confirm whether this mutation contributed to fosfomycin resistance. The MIC of fosfomycin in the NTUH-2044 fosA^KP^ I91V mutant was 8-fold higher (from 32 to 256 mg/dl) than that in the wild type (Table 3).

In addition, we observed that several strains shared the same resistance mechanism. To distinguish these strains, we analyzed the capsular types using the available nucleotide sequences and performed pulsed-field gel electrophoresis (PFGE) analysis. Among the 14 low-level fosfomycin-resistant strains with the fosA I91V mutation, we detected five different capsular types (K5, K47, K54, K64, and KN2). However, we could not distinguish six strains with capsular type K47 using the available methods. All of the available nucleotide sequences among these six K47 CRKP strains were identical, and the results of MLST showed that all six strains belonged to ST11. Six strains shared the same gene deficiency of glpT D274V and uhpT ES 69 (FO27, 28, 29, 30, 31, 32). FO31 had one nucleotide change in uhpB, and FO29 had two fragments different from other strains of PFGE analysis (Supplementary Figure 2). The MLST results also showed that all six strains belonged to ST11.

## Discussion

Treatment of CRKP remains a challenge, with fosfomycin as one of the limited options available. A recent report showed that the fosfomycin resistance rate (approximately 32–48.5%) is high among CRKP (Kaase et al., 2014; Yang et al., 2019; Liu et al., 2020). Several studies have reported that the fosA family is the main mechanism of CRKP resistance, especially in East Asian countries. Among several variants of the fosA family, fosA3 was the most commonly reported subtype of plasmid-mediated fosA based on reports from Taiwan (Tseng et al., 2017), Korea (Lee et al., 2012), and China (Wang et al., 2020). Huang et al. (2017) and Tseng et al. (2017) reported that fosA5 was also prevalent among CRKP. However, according to a previous literature (Ma et al., 2015), fosA5 showed high similarity to the chromosomal fosA^KP^ of *K. pneumoniae*, which can be found in most of *K. pneumoniae* (Ito et al., 2017). In the present study, we designed suitable primers to distinguish all *K. pneumoniae* strains, including fosfomycin-susceptible strains such as NUTH-K2044. Therefore, the detection of fosA5 by amplification may not contribute to plasmid-mediated high level fosfomycin resistance. The results of analysis of surrounding elements of fosA3 and plasmid transformation support that only fosA3, and not fosA^KP^, was located on the plasmid.

Chromosomal fosA^KP^ is present in most *K. pneumoniae*, and fosA^KP^ I91V has also been reported previously by Ito et al. (2017). However, the relationship between the mutation and MIC of fosfomycin was not studied. A previous study suggested that chromosomally encoded fosA was associated with a higher MIC distribution in CRKP (Elliott et al., 2019). However, most *K. pneumoniae* harboring *fosA* still exhibited MIC below CLSI breakpoints (64 mg/dL), and the difference between resistant and susceptible strains was not clear. To the best of our knowledge, the genetic or functional mechanism underlying this phenomenon is not yet reported. Our study is the first to document that chromosomal fosA^KP^ I91V is related to low-level fosfomycin resistance. Mutagenesis of *fosA* increased the MIC by eight-fold (from 32 to 256 mg/dL) in NTUH-K2044. Furthermore, genetic studies revealed that 39/40 of the fosfomycin-resistant CRKP contained chromosomal fosA^KP^ I91V, and this is the only mechanism observed in low-level fosfomycin-resistant strains. We detected approximately 25% of the fosfomycin-susceptible strains carrying chromosomal fosA^KP^ I91V, suggesting that chromosomal fosA^KP^ I91V might not be sufficient to convert strains with very low intrinsic fosfomycin-resistant strains into resistant strains. The chromosomal fosA^KP^ did not raised concern in previous studies (Yang et al., 2019; Liu et al., 2020) of fosfomycin resistant mechanism. However, the chromosomally encoded fosA may also increase risk of development of drug resistance during treatment, which should be taken into consideration on choosing therapeutic option.

Aside from the chromosomal *fosA* mutation, transporter gene deficiencies were the most common mechanism identified among the high-level resistant strains in our study. Although transporter gene deficiency-related resistance has been well studied in *E. coli*, there are only limited reports on *K. pneumoniae*. Lu et al. (2016) reported that glpT and uhpT were found in 97% of fosfomycin-resistant extended spectrum β-lactamase (ESBL)-producing *K. pneumoniae*. Various amino acid substitutions were found in the study. However, most of the strains in their study exhibited intermediate resistance, and genetic studies were not performed to confirm this relationship. In the study conducted by Liu et al. (2020), only 3 of 48 strains harbored deletion or mutation of glpT. No gene deficiency or functional changes were found in the G6P transporter system. Furthermore, an antimicrobial susceptibility testing above 512 mg/dL was not performed. In our study, deficient transporters were associated with various kinds of gene mutations, including early stop codons, insertion in the coding region, and single amino acid mutations in transporters and their regulatory genes. The entire uhpABCT operon was also deleted in two of the strains. Deficiencies in the transporter genes in these strains contributed to high fosfomycin resistance (MIC > 1,024 mg/dL). As these transporters are not essential for *K. pneumoniae*, it is not surprising that deficient transporters with various gene mutations were frequently observed under fosfomycin selective pressure. Notably, we found that three single amino acid mutations (glpT E299D, glpT D274V, and uhpC A393V) were related to fosfomycin resistance, and mutagenesis of deficiency transporter genes to NTUH-K2044 increased the MIC to 1,024 mg/dL. Although a previous biochemical study revealed that D274 and E299 are conserved sites in *E. coli* glpT essential to its function (Law et al., 2008), these mutations, as well as mutations in uhpC, have not yet been reported in *K. pneumoniae*.

We did not observe any murA mutations in this study. This was different from previous findings (Lu et al., 2016), wherein variant murA modifications accounted for 70% (21/30) of fosfomycin-resistant ESBL-producing *K. pneumoniae*. Liu et al. (2020) detected murA mutations in only one strain among 48 fosfomycin-resistant strains. As murA is an essential gene for cell wall synthesis, the possibility of losing murA function is low. Previous studies have observed that most of the strains with the murA mutation carried transporter gene mutations as well, and the MICs were not very high (Lu et al., 2016; Liu et al., 2020). However, mutagenesis was not performed, and the mechanism underlying it was not elucidated. Therefore, the murA mutation might be polymorphisms, and other mechanisms may be responsible for the resistance.

In our previous study (Pan et al., 2015), we demonstrated that capsular type-specific bacteriophages and capsule depolymerases could be novel therapies for eradicating CRKP. Due to limited options for treating CRKP infections, investigating the capsular type is essential. In the present study, K47 and K64 were the predominant capsular types of fosfomycin-resistant CRKP, but K47 was not frequently seen in our previous study. This finding suggests that novel capsular type-specific therapies, such as bacteriophage therapy or vaccine development, should be elucidated further.

In our study, detailed gene examination was performed, and serial functional assay and mutagenesis study confirmed that the detected gene mutations contribute to the high-level fosfomycin resistance. Three single amino acid mutations (glpT E299D, glpT D274V, and uhpC A393V) were observed for the first time to contribute to *K. pneumoniae* fosfomycin resistance. A series of strains shared same gene deficiency. Although the clinical strains were collected from different patients, it is possible that these strains belonged to a clone. We also performed capsular typing, nucleotide sequences analysis, MLST, and PFGE analysis. Most of the strains could be differentiated by these methods. However, some of them, such as six strains of K47 with fosAI91V (FO02-07), and four strains of K47 with glpT D274V (FOS27, 28, 30, 32), could not be excluded as a same clone.

In summary, we observed that chromosomal fosA^KP^ I91V was present in 39/40 fosfomycin-resistant CRKP and contributed to low level fosfomycin resistance. Chromosomal fosA^KP^ mutation is the only mechanism underlying the low-level resistance in CRKP strains, and most high-level resistance strains harbored this mutation. Transporter deficiency was the second most common resistance mechanism among high-level fosfomycin-resistant CRKP in Taiwan. Various G3P and G6P transporter gene mutations, including three novel single amino acid mutations (glpT E299D, glpT D274V, and uhpC A393V), were identified. Plasmid-mediated *fosA3* only accounted for 27% of the high-level resistant mechanisms. No murA mutations were found. These findings may provide new therapeutic approaches against CRKP infection in Taiwan.

## Data Availability Statement

The datasets presented in this study can be found in online repositories. The names of the repository/repositories and accession number(s) can be found below: https://www.ncbi.nlm.nih.gov/genbank/, JAHZSM000000000; https://www.ncbi.nlm.nih.gov/genbank/, CP073002.

## Author Contributions

Y-PW and Y-HChe: study concept and design. Y-PW, Y-HChe, I-CH, P-HC, and Y-HCha: carrying out the experiments. Y-PW: analysis and interpretation of data. Y-PW and Y-TL: drafting of the manuscript. Y-HChe, I-CH, P-HC, and Y-HCha: statistical analysis. Y-TL, H-CY, and J-TW: administrative, technical, and material support. H-CY and J-TW: study supervision. All authors contributed to the article and approved the submitted version.

## Conflict of Interest

The authors declare that the research was conducted in the absence of any commercial or financial relationships that could be construed as a potential conflict of interest.

## Publisher’s Note

All claims expressed in this article are solely those of the authors and do not necessarily represent those of their affiliated organizations, or those of the publisher, the editors and the reviewers. Any product that may be evaluated in this article, or claim that may be made by its manufacturer, is not guaranteed or endorsed by the publisher.
