# Supplementary material for: Transporter Genes and fosA Associated With Fosfomycin Resistance in Carbapenem-Resistant Klebsiella pneumoniae
Source: Front Microbiol. 2022 Jan 31;13:816806. doi: 10.3389/fmicb.2022.816806 (PMC8841775; doi:10.3389/fmicb.2022.816806)
Supplement: Supplementary file 1 [file Data_Sheet_1.DOCX]

Supplemental Table 1. Primers in the study

| **Primer** | **Sequence** |  |
| --- | --- | --- |
| **pKO3L** | AGGGCAGGGTCGTTAAATAGC | Plasmid construction |
| **KmF inverse** | tcccgttgaatatggctcat | Plasmid construction |
| **murA F** | GCAAACTCAATGGCTTCTAAGC | *murA* gene sequencing |
| **murA R** | GAAGAAGATCGACAAGTGATGTG |  |
| **cyaA F** | GTGACGGTTTTTGTTGAAATACTG | *cyaA* gene sequencing |
| **cyaA R** | CGCCTTATCCGGGCCAAAT |  |
| **cyaA IF** | GCG GTT CGA CCC AGC ATA TC |  |
| **ptsI F** | GGTTAAACTGATGGCTGAACTCG | *ptsI* gene sequencing |
| **ptsI R** | CAAACAAACCCATGATCTTCTCC |  |
| **glpT F** | GCGACCATATTTGAAGTTGGTG | *glpT* gene sequencing |
| **glpT R** | CAGCGCCGAACTGGACAG |  |
| **glpR F** | GCGAAAACGCGCATAAAGAC | *glpR* gene sequencing |
| **glpR R** | GCAGGACGCGGTACAGC |  |
| **uhpT F** | CGGCGCGCTGGTTTTTAC | *uhpT* gene sequencing |
| **uhpT R** | GCGCTTTTTGCATCAGGC |  |
| **uhpA F** | GTGAATCCGTAGGCCGGATAAG | *uhpA* gene sequencing |
| **uhpA R** | CTCCACAGGCAGAACCAGG |  |
| **uhpB F** | CAACCTGCTGGAAAAGCTG | *uhpB* gene sequencing |
| **uhpB R** | GTTTATCGCTAATCGGCGCAG |  |
| **uhpC F** | AATATCGTCAAGCACGCCAG | *uhpC* gene sequencing |
| **uhpC R** | GACGGCGTCCAGGTAAAAC |  |
| **IS6 FF** | AGTGGCAGATCCCGCGATTC | gene sequencing between IS6 |
| **IS6 RR** | GGTTTCATCCATGTGCCACG |  |
| **FosA3 F** | ATGCTGCAGGGATTGAATCATC | *fosA3* gene sequencing |
| **FosA3 R** | TCAATCAAAAAAGACCATCCCC |  |
| **FosA F** | ATGCTGAGTGGACTGAATCACCTG | FosA gene sequencing |
| **FosA R** | TCACTCAGCAAAAAACACCATCC |  |
| **FosA frk F** | CGACGCCATAGGCAAAGCC | FosA gene mutagenesis |
| **FosA frk R** | GACGCAGCCAAGCCTGAAG |  |
| **glpT frk F** | GCGGCCGCCTACTATTTGG | *glpT* gene mutagenesis |
| **glpT frk R** | GCGGCCGCTGCGGGAAAC |  |
| **glpT out F** | CTGCTGGTTATCGTGATGC | *glpT* gene mutagenesis check |
| **glpT out R** | CAGTTCGTACGCCGGGTAAG |  |
| **CP05 F1** | CTCTCATCCATACTCTATGAC | CP05 alignment analysis |
| **CP05 R1** | GTCGGACCTGGCATAATG |  |
| **uhpC frk F** | CCGGTATCGTCAGCGGCCGCTCCG | *uhpC* gene mutagenesis |
| **uhpC frk R** | CGGTCTCTTTGTCCTCTTCG |  |
| **KP-wza-CF2** | GGGTTTTTATCGGGTTGTAC | Capsular typing |
| **KP-wzc-CR2** | GCTTCCATCATTGCAAAATG |  |
|  |  |  |

**Supplemental Figure 1. mRNA expression level between low-level fosfomycin resistant CRKP strains**

**
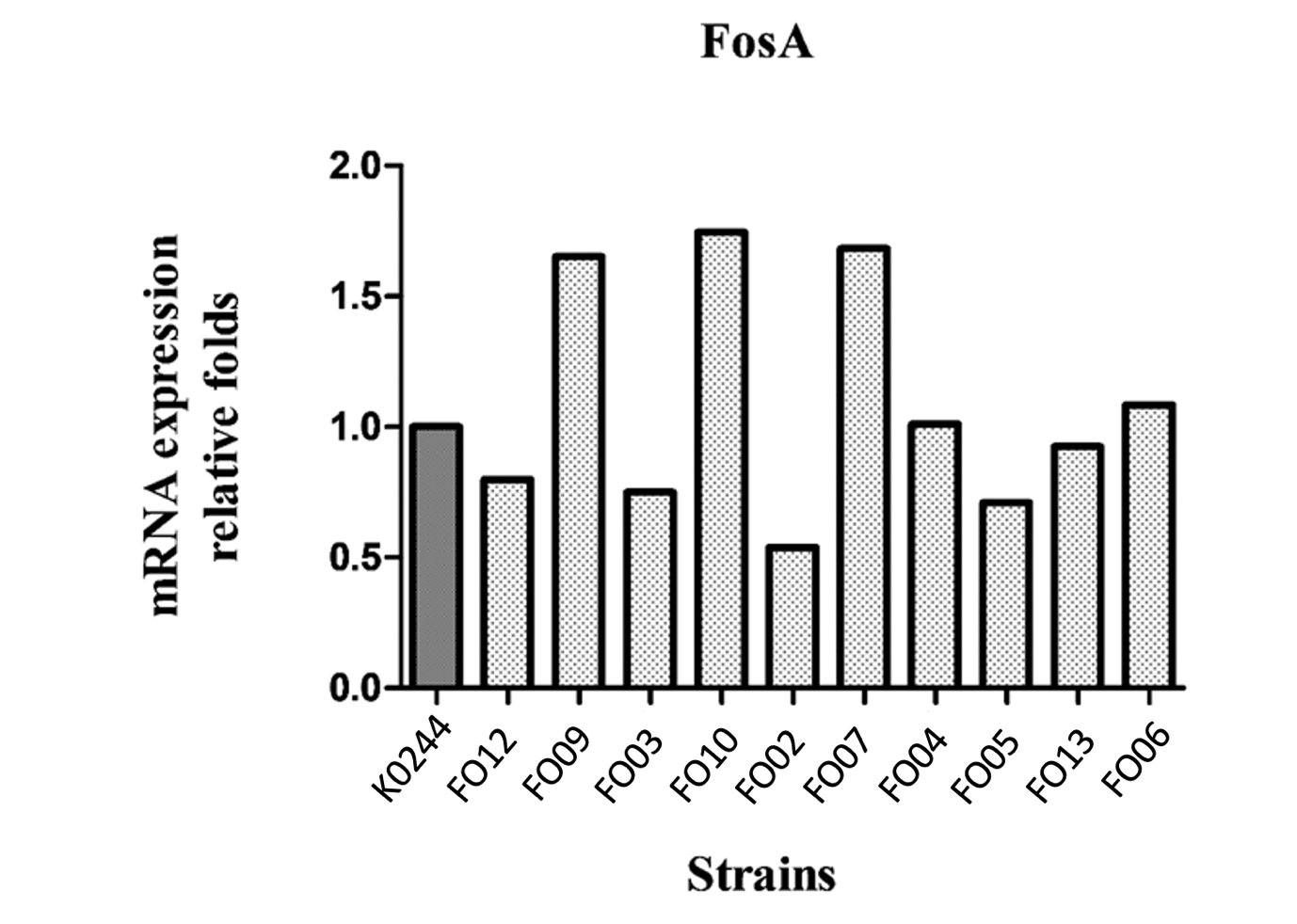
**

**Supplemental Figure 2. PFGE analysis of FO27, FO28, FO29, FO30, FO31
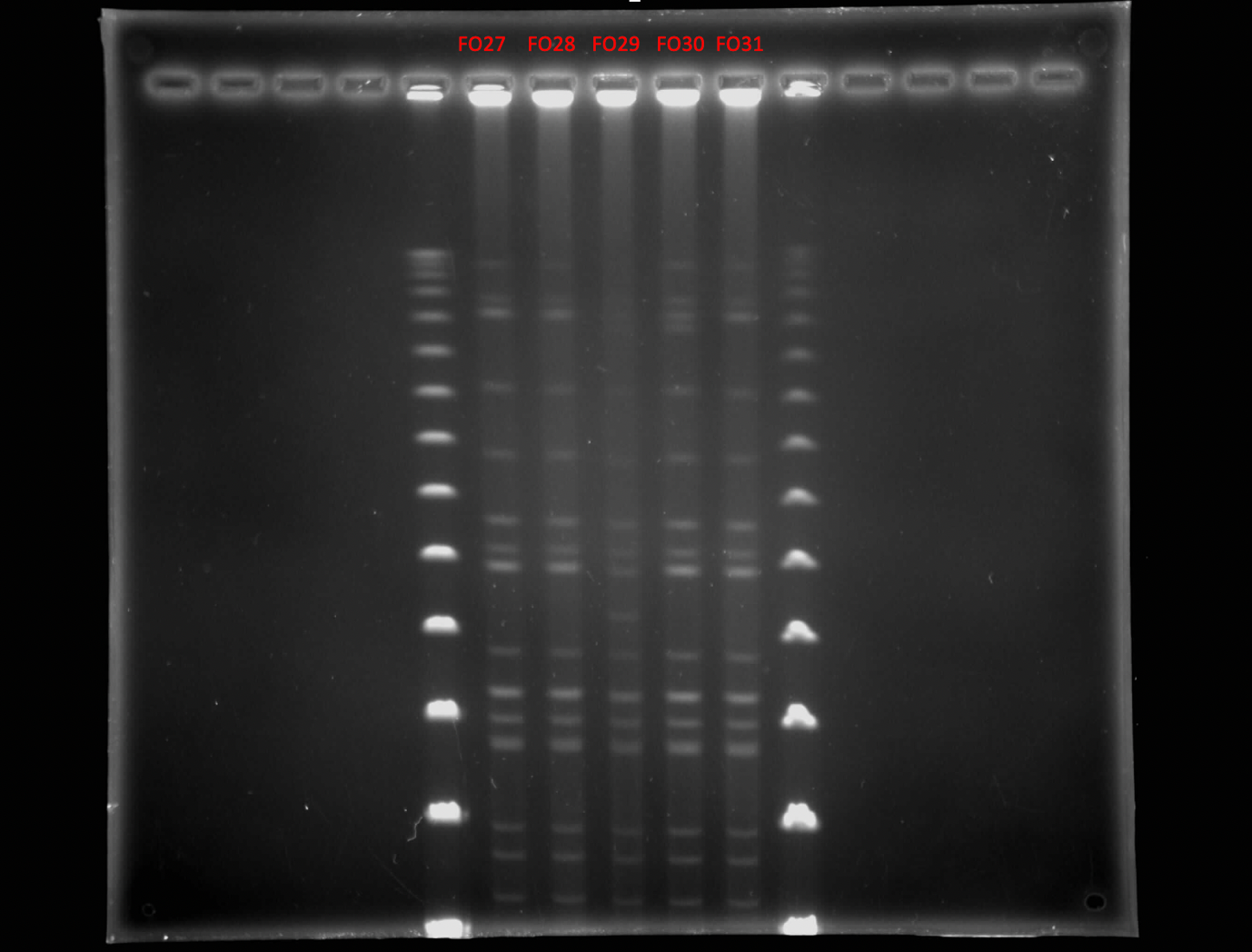
**

**MLST results of fosfomycin resistant CRKP**

**FO01 (ST76)**

>rpoB

TGGCTTGCTGCCGTGTAGAGCGTGCGGTGATGAGCGTCTGTGTGCTTGGCGATCTGGATACCCGTGATGCCTCAGGATATGATCAACGCCAAGCCGATTTCCGCAGCAGTGAAAGAGTTCTTTGGTTCCAGCCAGCTGTCTCAGTTTATGGACCAGAACAACCCGCTGTCTGAGATTACGCACAAACGTCGTATCTCCGCACTCGGCCCAGGCGGTCTGACCCGTGAGCGCGCAGGCTTCGAAGTTCGAGACGTACACCCGACCCACTACGGTCGCGTATGTCCGATCGAAACGCCTGAAGGTCCGAACATCGGTCTGATTAACTCCCTGTCCGTGTACGCGCAGACCAACGAATATGGCTTCCTTGAGACGCCGTATCGTAAAGTGACCAACGGTGTGGTTACTGACGAAATTCACTACCTGTCTGCTATCGAAGAAGGCAACTACGTTATCGCTCAGGCGAACTCCAACCTGGATGAAAACGGCCACTTCGTAGAAGATCTGGTTACCTGCCGTAGCAAAGGCGAATCCAGCTTGTTCAGCCGCGACCAGGTTGACTACATGGACGTATCCACCCAGCAGGTGGTATCCGTCGGTGCGTCCCTGATCCCGTTCCTGGAACACGATGACGCCAACCGTGCATTGATGGGTGCGAACATGCAACGTCAGGCGGTTCCGACTCTGCGCGCTGATAAGCCGCTGGTTGGTACCGGTATGGAACGTGCTGTTGCCGTTGACTCCGGTGTTACTGCCGTGGCTAAACGTGGCGGTACCGTTCAGTACGTGGATGCTTCCCGTATCGTTATCAAAGTTAACGAAGACGAGATGTACCCGGGCGAAGCAGGTATCGACATCTATAACCTGACCAAGTACACCCGTTCTAACCAGAACACCTGCATCAACCAGATGCCTTGCGTGTCCCTGGGCGAACCTATTGAGCGCGGCGACGTGCTGGCAGACGGCCCGTCCACCGACCTCGGTGAGCTGGCGCTGGGTCAGACATGCGTGTAGCGTTCATGCCGTGAACGGTACTTTTTCAAAAAAAAACATCAAAAAGGAAGGTGGAG

>gapA

GGCATCCAGTCGTTGAGTGAGACGGTCATCTGGTCGTTAACGGTAAAAAAATCCGTGTTACCGCTGAACGTGACCCGGCTAACCTGAAGTGGGACGAAGTTGGTGTTGACGTTGTTGCTGAAGCAACCGGTATCTTCCTGACCGACGAAACCGCTCGTAAACACATCACCGCTGGCGCGAAAAAAGTCGTTCTGACTGGCCCGTCCAAAGACAACACTCCGATGTTCGTTCGCGGCGCTAACTTCGACGCTTACGCTGGCCAGGACATCGTTTCCAACGCTTCCTGCACCACCAACTGCCTGGCGCCGCTGGCTAAAGTTATCAACGACAACTTCGGTATCGTTGAAGGCCTGATGACCACCGTCCACGCTACCACCGCTACTCAGAAAACCGTTGATGGCCCGTCTCACAAAGACTGGCGCGGCGGCCGCGGCGCAGCTCAAAACATCATCCCGTCCTCTACCGGCGCTGCTAAAGCAGTAGGTAAAGTACTGCCAGAACTGAACGGCAAACTGACCGGTATGGCGTTCCGCGTTCCAACTCCGAACGTATCTGTTGTTGACCTGACCGTTCGTCTGGAAAAAGCAGCGTCCTACGAAGAAATCAAGAAAGCCATCAAAGCCCTTTTTTGGGAGAAAA

>mdh

CAGGTAGCGATCGCTCGGTTACGCCGGGCGTGGCGGTAGATCTAAGTCATATCCCCACAGATGTAAAAATTAAAGGATTTTCCGGTGAAGACGCTACTCCGGCGCTGGAAGGCGCGGATGTAGTGCTGATCTCCGCGGGCGTGGCGCGTAAGCCCGGCATGGATCGTTCCGACCTGTTTAATGTGAATGCGGGTATCGTGAAGAACCTCGTGCAGCAGATTGCCAAAACCTGCCCGCAGGCCTGCATCGGCATTATCACCAACCCGGTGAATACCACCGTGGCTATCGCCGCCGAAGTACTGAAAAAAGCCGGCGTGTACGATAAAAACAAACTGTTCGGCGTTACCACGCTGGACATCATCCGTTCCAATACCTTTGTGGCGGAGCTGAAAGGTAAATCGGCAACCGAGGTGGAAGTCCCGGTCATTGGTGGTCACTCCGGGGTCACCATTCTGCCTTTACTGTCGCAGATCCCCGGCGTCAGCTTTAGCGATCAGGAAATTGCCGACCTGACTAAACGTATTCAGAACGCCGGTACCGAAGTCGTGGAAGCGAAAGCGGGCGGCGGGTCGGCGACCTTGTCGATGGGCCAGGCGGCTGCCCGTTTTGGTCTCTCTCTGGTTCGCGCCATGCAGGGGGAAAAAGGCGTGGTGGAGTGCGCCTACGTGGAAGGCGACGGCCACTATGCGCGTTTCTTCTCCCAGCCGCTGCTGTGGGAAAAAAAAACGGAGA

>pgi

CTAATATTCCTATCGCTGGTCACACTTCTTCGGTGCGGAACCGAAGCGATTCTGCCGTACGACCAGTACATGCACCGCTTTGCCGCTTACTTCCAGCAGGGCAACATGGAGTCCAACGGTAAGTATGTTGACCGTAACGGCCACGCGGTAGACTACCAGACTGGCCCAATCATCTGGGGTGAGCCGGGCACCAACGGTCAGCACGCGTTCTACCAGCTGATCCACCAGGGCACCAAAATGGTACCGTGCGATTTCATCGCTCCGGCTATCACCCACAACCCGCTGTCTGACCACCATCAGAAACTGCTGTCTAACTTCTTCGCCCAGACCGAGGCCCTGGCCTTTGGTAAATCCCGCGAAGTGGTTGAGCAGGAATATCGCGATCAGGGTAAAGACCCGGCGACCCTGGAGCACGTGGTGCCGTTCAAAGTGTTCGAAGGTAACCGCCCGACTAACTCCATCCTGCTGCGTGAGATCACCCCGTTCAGCCTCGGGGCGCTGATTGCCCTGTACGAGCACAAAATCTTCACCCAGGGCGCGATCCTCAACATCTTCACCTTTGACCAGTGGGGCGTTGAGCTGGGCAAACAGCTGGCTAACCGCATCCTGCCGGAGCTGAAAGACGGCAGCGAAGTTAGCAGCCACGACAGCTCTACTAACGGCCTGATTAACCGCTATAAAGGGGGGGGCGGGGA

>phoE

ATAGCTCTGTGTCTCATCTGACCTGCAGTACCAGGGTAAAAACGAAGGCCGTGAAGCGAAGAAACAGAACGGCGACGGCGTCGGCACCTCGTTAAGCTATGATTTCGGCGGCAGCGACTTCGCCGTCAGCGCGGCCTACACCAGCTCCGACCGTACCAACGATCAGAACCTGCTGGCCCGCGGCCAGGGTTCGAAAGCGGAAGCCTGGGCGACCGGCCTGAAATATGACGCCAACAATATCTACCTGGCGACCATGTACTCTGAAACCCGCAAGATGACCCCGATCAGCGGCGGCTTTGCCAACAAAGCGCAGAACTTTGAAGCGGTGGCGCAGTATCAGTTCGACTTCGGTCTGCGTCCGTCCCTCGGCTATGTGCTGTCGAAAGGGAAGGATATCGAAGGGGTGGGGAGTGAAGATCTGGTTAACTACATCGACGTGGGCCTGACCTATTACTTCAACAAAAACATGAACGCCTTCGTGGATTACAAAATCAACCAGCTGAAAAGCGATAACAAACTCGGCATCAACGATGACGACATCGTCGCGCTGGGTATCACCTACATTTTTTCTGATAACAA

>infB

ATAGGTTGCTCGGCGAGCGGGTGGTATTACCCAGCACATCGGTGCTTACCACGTCGAAACCGACAACGGCATGATCACCTTCCTGGATACCCCGGGCCACGCCGCGTTTACCTCCATGCGTGCTCGTGGCGCGCAGGCGACGGATATCGTGGTTCTGGTGGTGGCGGCAGACGACGGCGTGATGCCGCAGACTATCGAAGCTATCCAGCACGCTAAAGCGGCGCAGGTACCGGTGGTAGTGGCGGTGAACAAGATCGATAAGCCAGAAGCCGATCCGGATCGCGTGAAGAACGAACTGTCCCAGTACGGCATCCTGCCGGAAGAGTGGGGCGGCGAGAGCCAGTTCGTCCACGTTTCCGCGAAAGCGGGTACCGGCATCGACGACCTGCTGGACGCGATCCTGCTGCAGGCTGAAGTTCTTGGCTTTAAAAGCGA

>tonB

GGAAGGACTAGCTTCGTCGACGCAGCCGATAGAGATCACAATGGTGGCGCCGGCCGATCTTGAGCCGCCTCCGGCGGCGCAGCCTGTCGTGGAGCCCGTTGTTGAACCCGAACCTGAGCCGGAGACAGAGGTAGCGCCTGAACCGCCGAAAGAGGCGCCGGTGGTGATCCATAAACCGGAACCTAAGCCGAAGCCCAAACCTAAACCCAAGCCTAAGCCGGCGAAAAAGGTTGAACAGCCGAAGCGGGAAGTGAAGCCGGCAGCAGAGCCGCGTCCGGCCTCGCCGTTTGAAAACAACAATACGGCGCCGGCGCGTACAGCGCCAAGTACCTCGACCGCAGCGGCTAAACCCACCGTTACTGCTCCGAGCGGCCCGCGGGCGATCAGCCGCGTTCAGCCGTCCTATCCGGCGCGCGCTCAGGCGCTGCGCATTGAAGGGACGGTACGGGTGAAGTTTGACGTTTCGCCTGATGGCCGCATTGATAATCTGCAGATCCTCTCTGCTCCACCGGGGCAAATAA

FO02 (ST11)

>rpoB

TAGACTGGCTGTCGTGTAGAGCGTGCGGTGAGAGCGTCTGTCTCTTGGCGATCTGGATACCCTGATGCCTCAGGATATGATCAACGCCAAGCCGATTTCCGCAGCAGTGAAAGAGTTCTTTGGTTCCAGCCAGCTGTCTCAGTTTATGGACCAGAACAACCCGCTGTCTGAGATTACGCACAAACGTCGTATCTCCGCACTCGGCCCAGGCGGTCTGACCCGTGAGCGCGCAGGCTTCGAAGTTCGAGACGTACACCCGACCCACTACGGTCGCGTATGTCCGATCGAAACGCCTGAAGGTCCGAACATCGGTCTGATTAACTCCCTGTCCGTGTACGCGCAGACCAACGAATATGGCTTCCTTGAGACGCCGTATCGTAAAGTGACCAACGGTGTGGTTACTGACGAAATTCACTACCTGTCTGCTATCGAAGAAGGCAACTACGTTATCGCTCAGGCGAACTCCAACCTGGATGAAAACGGCCACTTCGTAGAAGATCTGGTTACCTGCCGTAGCAAAGGCGAATCCAGCTTGTTCAGCCGCGACCAGGTTGACTACATGGACGTATCCACCCAGCAGGTGGTATCCGTCGGTGCGTCCCTGATCCCGTTCCTGGAACACGATGACGCCAACCGTGCATTGATGGGTGCGAACATGCAACGTCAGGCGGTTCCGACTCTGCGCGCTGATAAGCCGCTGGTTGGTACCGGTATGGAACGTGCTGTTGCCGTTGACTCCGGTGTTACTGCCGTGGCTAAACGTGGCGGTACCGTTCAGTACGTGGATGCTTCCCGTATCGTTATCAAAGTTAACGAAGACGAGATGTACCCGGGCGAAGCAGGTATCGACATCTATAACCTGACCAAGTACACCCGTTCTAACCAGAACACCTGCATCAACCAGATGCCTTGCGTGTCCCTGGGCGAACCTATTGAGCGCGGCGACGTGCTGGCAGACGGCCCGTCCACCGACCTCGGTGAGCTGGCGCTGGGTCAGAACATGCGTGTAGCGTTCATGCCGTGGAACGTCTTTTTTTTGAATA

>gapA

CAGATCAGATCGTTGAGTGAAGACGGTCATCTGGTCGTTAACGGTAAAAAAATCCGTGTTACCGCTGAACGTGACCCGGCTAACCTGAAGTGGGACGAAGTTGGTGTTGACGTTGTTGCTGAAGCAACCGGTATCTTCCTGACCGACAAAACCGCTCGTAAACACATCACCGCTGGCGCGAAAAAAGTCGTTCTGACTGGCCCGTCCAAAGACAACACTCCGATGTTCGTTCGCGGCGCTAACTTCGACGCTTACGCTGGCCAGGACATCGTTTCCAACGCTTCCTGCACCACTAACTGCCTGGCGCCGCTGGCTAAAGTTATCAACGACAACTTCGGTATCGTTGAAGGCCTGATGACCACCGTCCACGCTACCACCGCTACTCAGAAAACCGTTGATGGCCCGTCTCACAAAGACTGGCGCGGCGGCCGCGGCGCAGCTCAGAACATCATCCCGTCCTCTACCGGCGCTGCTAAAGCAGTAGGTAAAGTACTGCCAGAACTGAACGGCAAACTGACCGGTATGGCGTTCCGCGTTCCAACTCCGAACGTATCTGTTGTTGACCTGACCGTTCGTCTGGAAAAAGCAGCGTCCTACGAAGAAATCAAGAAAGCCATCAAAGCCGTTTTCTGAAGA

>mdh

AACGAGCAACTCGCTCGGTTACGCCGGGCGTGGCGGTAGATCTAAGTCATATCCCCACAGATGTAAAAATTAAAGGATTTTCCGGTGAAGACGCTACTCCGGCGCTGGAAGGCGCGGATGTAGTGCTGATCTCCGCGGGCGTGGCGCGTAAGCCCGGCATGGATCGTTCCGACCTGTTTAATGTGAATGCGGGTATCGTGAAGAACCTCGTGCAGCAGATTGCCAAAACCTGCCCGCAGGCCTGCATCGGCATTATCACCAACCCGGTGAATACCACCGTGGCTATCGCCGCCGAAGTACTGAAAAAAGCCGGCGTGTACGATAAAAACAAACTGTTCGGCGTTACCACGCTGGACATCATCCGTTCCAATACCTTTGTGGCGGAGCTGAAAGGTAAATCGGCAACCGAGGTGGAAGTCCCGGTCATTGGTGGTCACTCCGGGGTCACCATTCTGCCTTTACTGTCGCAGATCCCCGGCGTCAGCTTTAGCGATCAGGAAATTGCCGACCTGACTAAACGTATTCAGAACGCCGGTACCGAAGTCGTGGAAGCGAAAGCGGGCGGCGGGTCGGCGACCTTGTCGATGGGCCAGGCGGCTGCCCGTTTTGGTCTCTCTCTGGTTCGCGCCATGCAGGGGGAAAAAGGCGTGGTGGAGTGCGCCTACGTGGAAGGCGACGGCCACTATGCGCGTTTCTTCTCCCAGCCGCTGCTGCTGGAAAAAAAAACGGA

>pgi

TCGGACAGACGTCGCTCTGGTCACACTTCTTCGGTGCGGAACCGAAGCGATTCTGCCGTACGACCAGTACATGCACCGCTTTGCCGCTTACTTCCAGCAGGGCAACATGGAGTCCAACGGTAAGTATGTTGACCGTAACGGCCACGCGGTAGACTACCAGACTGGCCCAATCATCTGGGGTGAGCCGGGCACCAACGGTCAGCACGCGTTCTACCAGCTGATCCACCAGGGCACCAAAATGGTACCGTGCGATTTCATCGCTCCGGCTATCACCCACAACCCGCTGTCTGACCACCATCAGAAACTGCTGTCTAACTTCTTCGCCCAGACCGAGGCCCTGGCCTTTGGTAAATCCCGCGAAGTGGTTGAGCAGGAATATCGCGATCAGGGTAAAGACCCGGCGACCCTGGAGCACGTGGTGCCGTTCAAAGTGTTCGAAGGTAACCGCCCGACTAACTCCATCCTGCTGCGTGAGATCACCCCGTTCAGCCTCGGGGCGCTGATTGCCCTGTACGAGCACAAAATCTTCACCCAGGGCGCGATCCTCAACATCTTCACCTTTGACCAGTGGGGCGTTGAGCTGGGCAAACAGCTGGCTAACCGCATCCTGCCGGAGCTGAAAGACGGCAGCGAAGTTAGCAGCCACGACAGCTCTACTAACGGCCTGATTAACCGCTATAAGGGGGGGGGCGCGCGA

>phoE

GCTGATAGCATCGTGGCTGAATCTGACCTGCAGTACCAGGGTAAAACGAAGGCCGTGAAGCGAAGAAACAGAACGGCGACGGCGTCGGCACCTCGTTAAGCTATGATTTCGGCGGCAGCGACTTCGCCGTCAGCGCAGCCTACACCAGCTCCGACCGTACCAACGATCAGAACCTGCTGGCCCGCGGCCAGGGTTCGAAAGCGGAAGCCTGGGCGACCGGCCTGAAATATGACGCCAACAATATCTACCTGGCGACCATGTACTCTGAAACCCGCAAGATGACCCCGATCAGCGGCGGCTTTGCCAACAAAGCGCAGAACTTTGAAGCGGTGGCGCAGTATCAGTTCGACTTCGGTCTGCGTCCGTCCCTCGGCTATGTGCTGTCGAAAGGGAAGGATATCGAAGGGGTGGGGAGTGAAGATCTGGTTAACTACATCGACGTGGGCCTGACCTACTACTTCAACAAAAACATGAACGCCTTCGTGGATTACAAAATCAACCAGCTGAAAAGCGATAACAAACTCGGCATCAACGATGACGACATCGTCGCGCTGGGTATCACCTACGTTTTTTTAATAACAA

>infB

GCAGTTGCCTCGGCGAGCGGGTGGTATTACCCAGCACATCGGTGCTTACCACGTCGAAACCGACAACGGCATGATCACCTTCCTGGATACCCCGGGCCACGCCGCGTTTACCTCCATGCGTGCTCGTGGCGCGCAGGCGACGGATATCGTGGTTCTGGTGGTGGCGGCAGACGACGGCGTGATGCCGCAGACTATCGAAGCTATCCAGCACGCTAAAGCGGCGCAGGTACCGGTGGTAGTGGCGGTGAACAAGATCGATAAGCCAGAAGCCGATCCGGATCGCGTGAAGAACGAACTGTCCCAGTACGGCATCCTGCCGGAAGAGTGGGGCGGCGAGAGCCAGTTTGTCCACGTTTCCGCGAAAGCGGGTACCGGCATCGACGACCTGCTGGACGCGATCCTGCTGCAGGCTGAAGTTCTTGGCTTGAAAACCGA

>tonB

GCGTCCGCGTCTCGACGCAGCCGATAGAGATCACAATGGTGGCGCCGGCCGATCTTGAGCCGCCTCCGGCGGCGCAGCCTGTCGTGGAGCCCGTTGTTGAACCCGAACCTGAGCCGGAGCCAGAGGTAGCGCCTGAACCGCCGAAAGAGGCGCCGGTGGTGATCCATAAACCGGAACCTAAGCCGAAGCCCAAACCTAAACCCAAGCCTAAGCCGGAGAAAAAGGTTGAACAGCCGAAGCGGGAAGTGAAGCCGGCAGCAGAGCCGCGTCCGGCCTCGCCGTTTGAAAACAACAATACGGCGCCGGCGCGTACAGCGCCAAGTACCTCGACCGCAGCGGCTAAACCCACCGTTACTGCTCCGAGCGGCCCGCGGGCGATCAGCCGCGTTCAGCCGTCCTATCCGCCGCGCGCTCAGGCGCTGCGCATTGAAGGGACGGTACGGGTGAAGTTTGACGTTTCGCCTGATGGCCGCATTGATAATCTACAGATCCTCTCTGCTCGCCCGGGGGGAATATA

FO03 (ST11)

>rpoB

TAGACTGGCTGTCGTGTAGAGCGTGCGGTGAGAGCGTCTGTCTCTTGGCGATCTGGATACCCTGATGCCTCAGGATATGATCAACGCCAAGCCGATTTCCGCAGCAGTGAAAGAGTTCTTTGGTTCCAGCCAGCTGTCTCAGTTTATGGACCAGAACAACCCGCTGTCTGAGATTACGCACAAACGTCGTATCTCCGCACTCGGCCCAGGCGGTCTGACCCGTGAGCGCGCAGGCTTCGAAGTTCGAGACGTACACCCGACCCACTACGGTCGCGTATGTCCGATCGAAACGCCTGAAGGTCCGAACATCGGTCTGATTAACTCCCTGTCCGTGTACGCGCAGACCAACGAATATGGCTTCCTTGAGACGCCGTATCGTAAAGTGACCAACGGTGTGGTTACTGACGAAATTCACTACCTGTCTGCTATCGAAGAAGGCAACTACGTTATCGCTCAGGCGAACTCCAACCTGGATGAAAACGGCCACTTCGTAGAAGATCTGGTTACCTGCCGTAGCAAAGGCGAATCCAGCTTGTTCAGCCGCGACCAGGTTGACTACATGGACGTATCCACCCAGCAGGTGGTATCCGTCGGTGCGTCCCTGATCCCGTTCCTGGAACACGATGACGCCAACCGTGCATTGATGGGTGCGAACATGCAACGTCAGGCGGTTCCGACTCTGCGCGCTGATAAGCCGCTGGTTGGTACCGGTATGGAACGTGCTGTTGCCGTTGACTCCGGTGTTACTGCCGTGGCTAAACGTGGCGGTACCGTTCAGTACGTGGATGCTTCCCGTATCGTTATCAAAGTTAACGAAGACGAGATGTACCCGGGCGAAGCAGGTATCGACATCTATAACCTGACCAAGTACACCCGTTCTAACCAGAACACCTGCATCAACCAGATGCCTTGCGTGTCCCTGGGCGAACCTATTGAGCGCGGCGACGTGCTGGCAGACGGCCCGTCCACCGACCTCGGTGAGCTGGCGCTGGGTCAGAACATGCGTGTAGCGTTCATGCCGTGGAACGTCTTTTTTTTGAATA

>gapA

GGGTTCAGATCGTTGAGTGAGACGGTCATCTGGTCGTTAACGGTAAAAAAATCCGTGTTACCGCTGAACGTGACCCGGCTAACCTGAAGTGGGACGAAGTTGGTGTTGACGTTGTTGCTGAAGCAACCGGTATCTTCCTGACCGACAAAACCGCTCGTAAACACATCACCGCTGGCGCGAAAAAAGTCGTTCTGACTGGCCCGTCCAAAGACAACACTCCGATGTTCGTTCGCGGCGCTAACTTCGACGCTTACGCTGGCCAGGACATCGTTTCCAACGCTTCCTGCACCACTAACTGCCTGGCGCCGCTGGCTAAAGTTATCAACGACAACTTCGGTATCGTTGAAGGCCTGATGACCACCGTCCACGCTACCACCGCTACTCAGAAAACCGTTGATGGCCCGTCTCACAAAGACTGGCGCGGCGGCCGCGGCGCAGCTCAGAACATCATCCCGTCCTCTACCGGCGCTGCTAAAGCAGTAGGTAAAGTACTGCCAGAACTGAACGGCAAACTGACCGGTATGGCGTTCCGCGTTCCAACTCCGAACGTATCTGTTGTTGACCTGACCGTTCGTCTGGAAAAAGCAGCGTCCTACGAAGAAATCAAGAAAGCCATCAAAGCGTTTTTTTTAAAAA

>mdh

TCAGGTTCAGAGCTCTCGTTGTACGACATCGCTCCGGTTACGCCGGGCGTGGCGGTAGATCTAAGTCATATCCCCACAGATGTAAAAATTAAAGGATTTTCCGGTGAAGACGCTACTCCGGCGCTGGAAGGCGCGGATGTAGTGCTGATCTCCGCGGGCGTGGCGCGTAAGCCCGGCATGGATCGTTCCGACCTGTTTAATGTGAATGCGGGTATCGTGAAGAACCTCGTGCAGCAGATTGCCAAAACCTGCCCGCAGGCCTGCATCGGCATTATCACCAACCCGGTGAATACCACCGTGGCTATCGCCGCCGAAGTACTGAAAAAAGCCGGCGTGTACGATAAAAACAAACTGTTCGGCGTTACCACGCTGGACATCATCCGTTCCAATACCTTTGTGGCGGAGCTGAAAGGTAAATCGGCAACCGAGGTGGAAGTCCCGGTCATTGGTGGTCACTCCGGGGTCACCATTCTGCCTTTACTGTCGCAGATCCCCGGCGTCAGCTTTAGCGATCAGGAAATTGCCGACCTGACTAAACGTATTCAGAACGCCGGTACCGAAGTCGTGGAAGCGAAAGCGGGCGGCGGGTCGGCGACCTTGTCGATGGGCCAGGCGGCTGCCCGTTTTGGTCTCTCTCTGGTTCGCGCCATGCAGGGGGAAAAAGGCGTGGTGGAGTGCGCCTACGTGGAAGGCGACGGCCACTATGCGCGTTTCTTCTCCCAGCCGCTGCTGCTGGAAAAAAA

>pgi

AAAAAACCTCCTGTACTGCTGGCTCTGATCGGCATCTGGTACAACAACTTCTTCGGTGCGGAAACCGAAGCGATTCTGCCGTACGACCAGTACATGCACCGCTTTGCCGCTTACTTCCAGCAGGGCAACATGGAGTCCAACGGTAAGTATGTTGACCGTAACGGCCACGCGGTAGACTACCAGACTGGCCCAATCATCTGGGGTGAGCCGGGCACCAACGGTCAGCACGCGTTCTACCAGCTGATCCACCAGGGCACCAAAATGGTACCGTGCGATTTCATCGCTCCGGCTATCACCCACAACCCGCTGTCTGACCACCATCAGAAACTGCTGTCTAACTTCTTCGCCCAGACCGAGGCCCTGGCCTTTGGTAAATCCCGCGAAGTGGTTGAGCAGGAATATCGCGATCAGGGTAAAGACCCGGCGACCCTGGAGCACGTGGTGCCGTTCAAAGTGTTCGAAGGTAACCGCCCGACTAACTCCATCCTGCTGCGTGAGATCACCCCGTTCAGCCTCGGGGCGCTGATTGCCCTGTACGAGCACAAAATCTTCACCCAGGGCGCGATCCTCAACATCTTCACCTTTGACCAGTGGGGCGTTGAGCTGGGCAAACAGCTGGCTAACCGCATCCTGCCGGAGCTGAAAGACGGCAGCGAAGTTAGCAGCCACGACAGCTCTACTAACGGCCTGATTAACCGCTATAAGGGGGGGG

>phoE

CCCCCCACACCGACTTCTTCGGTCTGGTGGATGGCCTGGATCTGACCCTGCAGTACCAGGGTAAAAACGAAGGCCGTGAAGCGAAGAAACAGAACGGCGACGGCGTCGGCACCTCGTTAAGCTATGATTTCGGCGGCAGCGACTTCGCCGTCAGCGCAGCCTACACCAGCTCCGACCGTACCAACGATCAGAACCTGCTGGCCCGCGGCCAGGGTTCGAAAGCGGAAGCCTGGGCGACCGGCCTGAAATATGACGCCAACAATATCTACCTGGCGACCATGTACTCTGAAACCCGCAAGATGACCCCGATCAGCGGCGGCTTTGCCAACAAAGCGCAGAACTTTGAAGCGGTGGCGCAGTATCAGTTCGACTTCGGTCTGCGTCCGTCCCTCGGCTATGTGCTGTCGAAAGGGAAGGATATCGAAGGGGTGGGGAGTGAAGATCTGGTTAACTACATCGACGTGGGCCTGACCTACTACTTCAACAAAAACATGAACGCCTTCGTGGATTACAAAATCAACCAGCTGAAAAGCGATAACAAACTCGGCATCAACGATGACGACATCGTCGCGCTGGGTATCACC

>infB

ATATTCGTTCTACCAAGGTTGCCTCCGGCGAAGCGGGTGGTATTACCCAGCACATCGGTGCTTACCACGTCGAAACCGACAACGGCATGATCACCTTCCTGGATACCCCGGGCCACGCCGCGTTTACCTCCATGCGTGCTCGTGGCGCGCAGGCGACGGATATCGTGGTTCTGGTGGTGGCGGCAGACGACGGCGTGATGCCGCAGACTATCGAAGCTATCCAGCACGCTAAAGCGGCGCAGGTACCGGTGGTAGTGGCGGTGAACAAGATCGATAAGCCAGAAGCCGATCCGGATCGCGTGAAGAACGAACTGTCCCAGTACGGCATCCTGCCGGAAGAGTGGGGCGGCGAGAGCCAGTTTGTCCACGTTTCCGCGAAAGCGGGTACCGGCATCGACGACCTGCTGGACGCGATCCTGCTGCAGGCTGAAGTTCTTGACCTTGAAAGCGA

>tonB

GCGTCCGCGTCTCGACGCAGCCGATAGAGATCACAATGGTGGCGCCGGCCGATCTTGAGCCGCCTCCGGCGGCGCAGCCTGTCGTGGAGCCCGTTGTTGAACCCGAACCTGAGCCGGAGCCAGAGGTAGCGCCTGAACCGCCGAAAGAGGCGCCGGTGGTGATCCATAAACCGGAACCTAAGCCGAAGCCCAAACCTAAACCCAAGCCTAAGCCGGAGAAAAAGGTTGAACAGCCGAAGCGGGAAGTGAAGCCGGCAGCAGAGCCGCGTCCGGCCTCGCCGTTTGAAAACAACAATACGGCGCCGGCGCGTACAGCGCCAAGTACCTCGACCGCAGCGGCTAAACCCACCGTTACTGCTCCGAGCGGCCCGCGGGCGATCAGCCGCGTTCAGCCGTCCTATCCGCCGCGCGCTCAGGCGCTGCGCATTGAAGGGACGGTACGGGTGAAGTTTGACGTTTCGCCTGATGGCCGCATTGATAATCTACAGATCCTCTCTGCTCGCCCGGGGGGAATATA

FO04 (ST11)

>rpoB

TAGACTGGCTGTCGTGTAGAGCGTGCGGTGAGAGCGTCTGTCTCTTGGCGATCTGGATACCCTGATGCCTCAGGATATGATCAACGCCAAGCCGATTTCCGCAGCAGTGAAAGAGTTCTTTGGTTCCAGCCAGCTGTCTCAGTTTATGGACCAGAACAACCCGCTGTCTGAGATTACGCACAAACGTCGTATCTCCGCACTCGGCCCAGGCGGTCTGACCCGTGAGCGCGCAGGCTTCGAAGTTCGAGACGTACACCCGACCCACTACGGTCGCGTATGTCCGATCGAAACGCCTGAAGGTCCGAACATCGGTCTGATTAACTCCCTGTCCGTGTACGCGCAGACCAACGAATATGGCTTCCTTGAGACGCCGTATCGTAAAGTGACCAACGGTGTGGTTACTGACGAAATTCACTACCTGTCTGCTATCGAAGAAGGCAACTACGTTATCGCTCAGGCGAACTCCAACCTGGATGAAAACGGCCACTTCGTAGAAGATCTGGTTACCTGCCGTAGCAAAGGCGAATCCAGCTTGTTCAGCCGCGACCAGGTTGACTACATGGACGTATCCACCCAGCAGGTGGTATCCGTCGGTGCGTCCCTGATCCCGTTCCTGGAACACGATGACGCCAACCGTGCATTGATGGGTGCGAACATGCAACGTCAGGCGGTTCCGACTCTGCGCGCTGATAAGCCGCTGGTTGGTACCGGTATGGAACGTGCTGTTGCCGTTGACTCCGGTGTTACTGCCGTGGCTAAACGTGGCGGTACCGTTCAGTACGTGGATGCTTCCCGTATCGTTATCAAAGTTAACGAAGACGAGATGTACCCGGGCGAAGCAGGTATCGACATCTATAACCTGACCAAGTACACCCGTTCTAACCAGAACACCTGCATCAACCAGATGCCTTGCGTGTCCCTGGGCGAACCTATTGAGCGCGGCGACGTGCTGGCAGACGGCCCGTCCACCGACCTCGGTGAGCTGGCGCTGGGTCAGAACATGCGTGTAGCGTTCATGCCGTGGAACGTCTTTTTTTTGAATA

>gapA

GGGTTCAGATCGTTGAGTGAGACGGTCATCTGGTCGTTAACGGTAAAAAAATCCGTGTTACCGCTGAACGTGACCCGGCTAACCTGAAGTGGGACGAAGTTGGTGTTGACGTTGTTGCTGAAGCAACCGGTATCTTCCTGACCGACAAAACCGCTCGTAAACACATCACCGCTGGCGCGAAAAAAGTCGTTCTGACTGGCCCGTCCAAAGACAACACTCCGATGTTCGTTCGCGGCGCTAACTTCGACGCTTACGCTGGCCAGGACATCGTTTCCAACGCTTCCTGCACCACTAACTGCCTGGCGCCGCTGGCTAAAGTTATCAACGACAACTTCGGTATCGTTGAAGGCCTGATGACCACCGTCCACGCTACCACCGCTACTCAGAAAACCGTTGATGGCCCGTCTCACAAAGACTGGCGCGGCGGCCGCGGCGCAGCTCAGAACATCATCCCGTCCTCTACCGGCGCTGCTAAAGCAGTAGGTAAAGTACTGCCAGAACTGAACGGCAAACTGACCGGTATGGCGTTCCGCGTTCCAACTCCGAACGTATCTGTTGTTGACCTGACCGTTCGTCTGGAAAAAGCAGCGTCCTACGAAGAAATCAAGAAAGCCATCAAAGCGTTTTTTTTAAAAA

>mdh

TCAGGTTCAGAGCTCTCGTTGTACGACATCGCTCCGGTTACGCCGGGCGTGGCGGTAGATCTAAGTCATATCCCCACAGATGTAAAAATTAAAGGATTTTCCGGTGAAGACGCTACTCCGGCGCTGGAAGGCGCGGATGTAGTGCTGATCTCCGCGGGCGTGGCGCGTAAGCCCGGCATGGATCGTTCCGACCTGTTTAATGTGAATGCGGGTATCGTGAAGAACCTCGTGCAGCAGATTGCCAAAACCTGCCCGCAGGCCTGCATCGGCATTATCACCAACCCGGTGAATACCACCGTGGCTATCGCCGCCGAAGTACTGAAAAAAGCCGGCGTGTACGATAAAAACAAACTGTTCGGCGTTACCACGCTGGACATCATCCGTTCCAATACCTTTGTGGCGGAGCTGAAAGGTAAATCGGCAACCGAGGTGGAAGTCCCGGTCATTGGTGGTCACTCCGGGGTCACCATTCTGCCTTTACTGTCGCAGATCCCCGGCGTCAGCTTTAGCGATCAGGAAATTGCCGACCTGACTAAACGTATTCAGAACGCCGGTACCGAAGTCGTGGAAGCGAAAGCGGGCGGCGGGTCGGCGACCTTGTCGATGGGCCAGGCGGCTGCCCGTTTTGGTCTCTCTCTGGTTCGCGCCATGCAGGGGGAAAAAGGCGTGGTGGAGTGCGCCTACGTGGAAGGCGACGGCCACTATGCGCGTTTCTTCTCCCAGCCGCTGCTGCTGGAAAAAAA

>pgi

TGTGATGTGCGTTGCTCTGGTCACACTTCTTCGGTGCGGAACCGAAGCGATTCTGCCGTACGACCAGTACATGCACCGCTTTGCCGCTTACTTCCAGCAGGGCAACATGGAGTCCAACGGTAAGTATGTTGACCGTAACGGCCACGCGGTAGACTACCAGACTGGCCCAATCATCTGGGGTGAGCCGGGCACCAACGGTCAGCACGCGTTCTACCAGCTGATCCACCAGGGCACCAAAATGGTACCGTGCGATTTCATCGCTCCGGCTATCACCCACAACCCGCTGTCTGACCACCATCAGAAACTGCTGTCTAACTTCTTCGCCCAGACCGAGGCCCTGGCCTTTGGTAAATCCCGCGAAGTGGTTGAGCAGGAATATCGCGATCAGGGTAAAGACCCGGCGACCCTGGAGCACGTGGTGCCGTTCAAAGTGTTCGAAGGTAACCGCCCGACTAACTCCATCCTGCTGCGTGAGATCACCCCGTTCAGCCTCGGGGCGCTGATTGCCCTGTACGAGCACAAAATCTTCACCCAGGGCGCGATCCTCAACATCTTCACCTTTGACCAGTGGGGCGTTGAGCTGGGCAAACAGCTGGCTAACCGCATCCTGCCGGAGCTGAAAGACGGCAGCGAAGTTAGCAGCCACGACAGCTCTACTAACGGCCTGATTAACCGCTATAACGGGGGGGGCCGGGAA

>phoE

ACTTCTAGCAGGCTGCTCATCTGACCTGCAGTACCAGGGTAAAAACGAAGGCCGTGAAGCGAAGAAACAGAACGGCGACGGCGTCGGCACCTCGTTAAGCTATGATTTCGGCGGCAGCGACTTCGCCGTCAGCGCAGCCTACACCAGCTCCGACCGTACCAACGATCAGAACCTGCTGGCCCGCGGCCAGGGTTCGAAAGCGGAAGCCTGGGCGACCGGCCTGAAATATGACGCCAACAATATCTACCTGGCGACCATGTACTCTGAAACCCGCAAGATGACCCCGATCAGCGGCGGCTTTGCCAACAAAGCGCAGAACTTTGAAGCGGTGGCGCAGTATCAGTTCGACTTCGGTCTGCGTCCGTCCCTCGGCTATGTGCTGTCGAAAGGGAAGGATATCGAAGGGGTGGGGAGTGAAGATCTGGTTAACTACATCGACGTGGGCCTGACCTACTACTTCAACAAAAACATGAACGCCTTCGTGGATTACAAAATCAACCAGCTGAAAAGCGATAACAAACTCGGCATCAACGATGACGACATCGTCGCGCTGGGTATCACCTACCGTTTTCTTGATCACAA

>infB

ATAGTTGCTCGGCGAGCGGGTGGTATTACCCAGCACATCGGTGCTTACCACGTCGAAACCGACAACGGCATGATCACCTTCCTGGATACCCCGGGCCACGCCGCGTTTACCTCCATGCGTGCTCGTGGCGCGCAGGCGACGGATATCGTGGTTCTGGTGGTGGCGGCAGACGACGGCGTGATGCCGCAGACTATCGAAGCTATCCAGCACGCTAAAGCGGCGCAGGTACCGGTGGTAGTGGCGGTGAACAAGATCGATAAGCCAGAAGCCGATCCGGATCGCGTGAAGAACGAACTGTCCCAGTACGGCATCCTGCCGGAAGAGTGGGGCGGCGAGAGCCAGTTTGTCCACGTTTCCGCGAAAGCGGGTACCGGCATCGACGACCTGCTGGACGCGATCCTGCTGCAGGCTGAAGTTCTTGCTTAAAAAGCGA

>tonB

GCGTCCGCGTCTCGACGCAGCCGATAGAGATCACAATGGTGGCGCCGGCCGATCTTGAGCCGCCTCCGGCGGCGCAGCCTGTCGTGGAGCCCGTTGTTGAACCCGAACCTGAGCCGGAGCCAGAGGTAGCGCCTGAACCGCCGAAAGAGGCGCCGGTGGTGATCCATAAACCGGAACCTAAGCCGAAGCCCAAACCTAAACCCAAGCCTAAGCCGGAGAAAAAGGTTGAACAGCCGAAGCGGGAAGTGAAGCCGGCAGCAGAGCCGCGTCCGGCCTCGCCGTTTGAAAACAACAATACGGCGCCGGCGCGTACAGCGCCAAGTACCTCGACCGCAGCGGCTAAACCCACCGTTACTGCTCCGAGCGGCCCGCGGGCGATCAGCCGCGTTCAGCCGTCCTATCCGCCGCGCGCTCAGGCGCTGCGCATTGAAGGGACGGTACGGGTGAAGTTTGACGTTTCGCCTGATGGCCGCATTGATAATCTACAGATCCTCTCTGCTCGCCCGGGGGGAATATA

FO05 (ST11)

>rpoB

ATGCTGCTGGCGTGTAGAGCGTGCGGTGATGAGCGTCTGTCTCTTGGCGATCTGGATACCCTGATGCCTCAGGATATGATCAACGCCAAGCCGATTTCCGCAGCAGTGAAAGAGTTCTTTGGTTCCAGCCAGCTGTCTCAGTTTATGGACCAGAACAACCCGCTGTCTGAGATTACGCACAAACGTCGTATCTCCGCACTCGGCCCAGGCGGTCTGACCCGTGAGCGCGCAGGCTTCGAAGTTCGAGACGTACACCCGACCCACTACGGTCGCGTATGTCCGATCGAAACGCCTGAAGGTCCGAACATCGGTCTGATTAACTCCCTGTCCGTGTACGCGCAGACCAACGAATATGGCTTCCTTGAGACGCCGTATCGTAAAGTGACCAACGGTGTGGTTACTGACGAAATTCACTACCTGTCTGCTATCGAAGAAGGCAACTACGTTATCGCTCAGGCGAACTCCAACCTGGATGAAAACGGCCACTTCGTAGAAGATCTGGTTACCTGCCGTAGCAAAGGCGAATCCAGCTTGTTCAGCCGCGACCAGGTTGACTACATGGACGTATCCACCCAGCAGGTGGTATCCGTCGGTGCGTCCCTGATCCCGTTCCTGGAACACGATGACGCCAACCGTGCATTGATGGGTGCGAACATGCAACGTCAGGCGGTTCCGACTCTGCGCGCTGATAAGCCGCTGGTTGGTACCGGTATGGAACGTGCTGTTGCCGTTGACTCCGGTGTTACTGCCGTGGCTAAACGTGGCGGTACCGTTCAGTACGTGGATGCTTCCCGTATCGTTATCAAAGTTAACGAAGACGAGATGTACCCGGGCGAAGCAGGTATCGACATCTATAACCTGACCAAGTACACCCGTTCTAACCAGAACACCTGCATCAACCAGATGCCTTGCGTGTCCCTGGGCGAACCTATTGAGCGCGGCGACGTGCTGGCAGACGGCCCGTCCACCGACCTCGGTGAGCTGGCGCTGGGTCAGAACATGCGTGTAGCGTTCATGCCGTGGAACCG

>gapA

CACGATCCAGATCGTTGAGTGAGACGGTCATCTGGTCGTTAACGGTAAAAAAATCCGTGTTACCGCTGAACGTGACCCGGCTAACCTGAAGTGGGACGAAGTTGGTGTTGACGTTGTTGCTGAAGCAACCGGTATCTTCCTGACCGACAAAACCGCTCGTAAACACATCACCGCTGGCGCGAAAAAAGTCGTTCTGACTGGCCCGTCCAAAGACAACACTCCGATGTTCGTTCGCGGCGCTAACTTCGACGCTTACGCTGGCCAGGACATCGTTTCCAACGCTTCCTGCACCACTAACTGCCTGGCGCCGCTGGCTAAAGTTATCAACGACAACTTCGGTATCGTTGAAGGCCTGATGACCACCGTCCACGCTACCACCGCTACTCAGAAAACCGTTGATGGCCCGTCTCACAAAGACTGGCGCGGCGGCCGCGGCGCAGCTCAGAACATCATCCCGTCCTCTACCGGCGCTGCTAAAGCAGTAGGTAAAGTACTGCCAGAACTGAACGGCAAACTGACCGGTATGGCGTTCCGCGTTCCAACTCCGAACGTATCTGTTGTTGACCTGACCGTTCGTCTGGAAAAAGCAGCGTCCTACGAAGAAATCAAGAAAGCCATCAAAGCCGTTTTTCGAAAGA

>mdh

CGCGTAGGCGCTCGCTCGGTTACGCCGGGCGTGGCGGTAGATCTAAGTCATATCCCCACAGATGTAAAAATTAAAGGATTTTCCGGTGAAGACGCTACTCCGGCGCTGGAAGGCGCGGATGTAGTGCTGATCTCCGCGGGCGTGGCGCGTAAGCCCGGCATGGATCGTTCCGACCTGTTTAATGTGAATGCGGGTATCGTGAAGAACCTCGTGCAGCAGATTGCCAAAACCTGCCCGCAGGCCTGCATCGGCATTATCACCAACCCGGTGAATACCACCGTGGCTATCGCCGCCGAAGTACTGAAAAAAGCCGGCGTGTACGATAAAAACAAACTGTTCGGCGTTACCACGCTGGACATCATCCGTTCCAATACCTTTGTGGCGGAGCTGAAAGGTAAATCGGCAACCGAGGTGGAAGTCCCGGTCATTGGTGGTCACTCCGGGGTCACCATTCTGCCTTTACTGTCGCAGATCCCCGGCGTCAGCTTTAGCGATCAGGAAATTGCCGACCTGACTAAACGTATTCAGAACGCCGGTACCGAAGTCGTGGAAGCGAAAGCGGGCGGCGGGTCGGCGACCTTGTCGATGGGCCAGGCGGCTGCCCGTTTTGGTCTCTCTCTGGTTCGCGCCATGCAGGGGGAAAAAGGCGTGGTGGAGTGCGCCTACGTGGAAGGCGACGGCCACTATGCGCGTTTCTTCTCCCAGCCGCTGCTGTGGAAAAAAAAACCGAGA

>pgi

TCATAGTTGCGGGCGGCTCTGGTCACACTTCTTCGGTGCGGAACCGAAGCGATTCTGCCGTACGACCAGTACATGCACCGCTTTGCCGCTTACTTCCAGCAGGGCAACATGGAGTCCAACGGTAAGTATGTTGACCGTAACGGCCACGCGGTAGACTACCAGACTGGCCCAATCATCTGGGGTGAGCCGGGCACCAACGGTCAGCACGCGTTCTACCAGCTGATCCACCAGGGCACCAAAATGGTACCGTGCGATTTCATCGCTCCGGCTATCACCCACAACCCGCTGTCTGACCACCATCAGAAACTGCTGTCTAACTTCTTCGCCCAGACCGAGGCCCTGGCCTTTGGTAAATCCCGCGAAGTGGTTGAGCAGGAATATCGCGATCAGGGTAAAGACCCGGCGACCCTGGAGCACGTGGTGCCGTTCAAAGTGTTCGAAGGTAACCGCCCGACTAACTCCATCCTGCTGCGTGAGATCACCCCGTTCAGCCTCGGGGCGCTGATTGCCCTGTACGAGCACAAAATCTTCACCCAGGGCGCGATCCTCAACATCTTCACCTTTGACCAGTGGGGCGTTGAGCTGGGCAAACAGCTGGCTAACCGCATCCTGCCGGAGCTGAAAGACGGCAGCGAAGTTAGCAGCCACGACAGCTCTACTAACGGCCTGATTAACCGCTTAGGGGGGGGGGCGGGGGA

>phoE

AGGACATAGCTCTGTACTGAATCTGACCTGCAGTACCAGGGTAAAACGAAGGCCGTGAAGCGAAGAAACAGAACGGCGACGGCGTCGGCACCTCGTTAAGCTATGATTTCGGCGGCAGCGACTTCGCCGTCAGCGCAGCCTACACCAGCTCCGACCGTACCAACGATCAGAACCTGCTGGCCCGCGGCCAGGGTTCGAAAGCGGAAGCCTGGGCGACCGGCCTGAAATATGACGCCAACAATATCTACCTGGCGACCATGTACTCTGAAACCCGCAAGATGACCCCGATCAGCGGCGGCTTTGCCAACAAAGCGCAGAACTTTGAAGCGGTGGCGCAGTATCAGTTCGACTTCGGTCTGCGTCCGTCCCTCGGCTATGTGCTGTCGAAAGGGAAGGATATCGAAGGGGTGGGGAGTGAAGATCTGGTTAACTACATCGACGTGGGCCTGACCTACTACTTCAACAAAAACATGAACGCCTTCGTGGATTACAAAATCAACCAGCTGAAAAGCGATAACAAACTCGGCATCAACGATGACGACATCGTCGCGCTGGGTATCACCTACCGTTTTTTGGATCAA

>infB

ATAGGTTGCTCAGCGAGCGGGTGGTATTACCCAGCACATCGGTGCTTACCACGTCGAAACCGACAACGGCATGATCACCTTCCTGGATACCCCGGGCCACGCCGCGTTTACCTCCATGCGTGCTCGTGGCGCGCAGGCGACGGATATCGTGGTTCTGGTGGTGGCGGCAGACGACGGCGTGATGCCGCAGACTATCGAAGCTATCCAGCACGCTAAAGCGGCGCAGGTACCGGTGGTAGTGGCGGTGAACAAGATCGATAAGCCAGAAGCCGATCCGGATCGCGTGAAGAACGAACTGTCCCAGTACGGCATCCTGCCGGAAGAGTGGGGCGGCGAGAGCCAGTTTGTCCACGTTTCCGCGAAAGCGGGTACCGGCATCGACGACCTGCTGGACGCGATCCTGCTGCAGGCTGAAGTTCTTGGCTTTGAAAGCGA

>tonB

GCGTCCGCGTCTCGACGCAGCCGATAGAGATCACAATGGTGGCGCCGGCCGATCTTGAGCCGCCTCCGGCGGCGCAGCCTGTCGTGGAGCCCGTTGTTGAACCCGAACCTGAGCCGGAGCCAGAGGTAGCGCCTGAACCGCCGAAAGAGGCGCCGGTGGTGATCCATAAACCGGAACCTAAGCCGAAGCCCAAACCTAAACCCAAGCCTAAGCCGGAGAAAAAGGTTGAACAGCCGAAGCGGGAAGTGAAGCCGGCAGCAGAGCCGCGTCCGGCCTCGCCGTTTGAAAACAACAATACGGCGCCGGCGCGTACAGCGCCAAGTACCTCGACCGCAGCGGCTAAACCCACCGTTACTGCTCCGAGCGGCCCGCGGGCGATCAGCCGCGTTCAGCCGTCCTATCCGCCGCGCGCTCAGGCGCTGCGCATTGAAGGGACGGTACGGGTGAAGTTTGACGTTTCGCCTGATGGCCGCATTGATAATCTACAGATCCTCTCTGCTCGCCCGGGGGGAATATA

FO06 (ST11)

>rpoB

ATGCTGCTGGCGTGTAGAGCGTGCGGTGATGAGCGTCTGTCTCTTGGCGATCTGGATACCCTGATGCCTCAGGATATGATCAACGCCAAGCCGATTTCCGCAGCAGTGAAAGAGTTCTTTGGTTCCAGCCAGCTGTCTCAGTTTATGGACCAGAACAACCCGCTGTCTGAGATTACGCACAAACGTCGTATCTCCGCACTCGGCCCAGGCGGTCTGACCCGTGAGCGCGCAGGCTTCGAAGTTCGAGACGTACACCCGACCCACTACGGTCGCGTATGTCCGATCGAAACGCCTGAAGGTCCGAACATCGGTCTGATTAACTCCCTGTCCGTGTACGCGCAGACCAACGAATATGGCTTCCTTGAGACGCCGTATCGTAAAGTGACCAACGGTGTGGTTACTGACGAAATTCACTACCTGTCTGCTATCGAAGAAGGCAACTACGTTATCGCTCAGGCGAACTCCAACCTGGATGAAAACGGCCACTTCGTAGAAGATCTGGTTACCTGCCGTAGCAAAGGCGAATCCAGCTTGTTCAGCCGCGACCAGGTTGACTACATGGACGTATCCACCCAGCAGGTGGTATCCGTCGGTGCGTCCCTGATCCCGTTCCTGGAACACGATGACGCCAACCGTGCATTGATGGGTGCGAACATGCAACGTCAGGCGGTTCCGACTCTGCGCGCTGATAAGCCGCTGGTTGGTACCGGTATGGAACGTGCTGTTGCCGTTGACTCCGGTGTTACTGCCGTGGCTAAACGTGGCGGTACCGTTCAGTACGTGGATGCTTCCCGTATCGTTATCAAAGTTAACGAAGACGAGATGTACCCGGGCGAAGCAGGTATCGACATCTATAACCTGACCAAGTACACCCGTTCTAACCAGAACACCTGCATCAACCAGATGCCTTGCGTGTCCCTGGGCGAACCTATTGAGCGCGGCGACGTGCTGGCAGACGGCCCGTCCACCGACCTCGGTGAGCTGGCGCTGGGTCAGAACATGCGTGTAGCGTTCATGCCGTGGAACCG

>gapA

CACGATCCAGATCGTTGAGTGAGACGGTCATCTGGTCGTTAACGGTAAAAAAATCCGTGTTACCGCTGAACGTGACCCGGCTAACCTGAAGTGGGACGAAGTTGGTGTTGACGTTGTTGCTGAAGCAACCGGTATCTTCCTGACCGACAAAACCGCTCGTAAACACATCACCGCTGGCGCGAAAAAAGTCGTTCTGACTGGCCCGTCCAAAGACAACACTCCGATGTTCGTTCGCGGCGCTAACTTCGACGCTTACGCTGGCCAGGACATCGTTTCCAACGCTTCCTGCACCACTAACTGCCTGGCGCCGCTGGCTAAAGTTATCAACGACAACTTCGGTATCGTTGAAGGCCTGATGACCACCGTCCACGCTACCACCGCTACTCAGAAAACCGTTGATGGCCCGTCTCACAAAGACTGGCGCGGCGGCCGCGGCGCAGCTCAGAACATCATCCCGTCCTCTACCGGCGCTGCTAAAGCAGTAGGTAAAGTACTGCCAGAACTGAACGGCAAACTGACCGGTATGGCGTTCCGCGTTCCAACTCCGAACGTATCTGTTGTTGACCTGACCGTTCGTCTGGAAAAAGCAGCGTCCTACGAAGAAATCAAGAAAGCCATCAAAGCCGTTTTTCGAAAGA

>mdh

CGCGTAGGCGCTCGCTCGGTTACGCCGGGCGTGGCGGTAGATCTAAGTCATATCCCCACAGATGTAAAAATTAAAGGATTTTCCGGTGAAGACGCTACTCCGGCGCTGGAAGGCGCGGATGTAGTGCTGATCTCCGCGGGCGTGGCGCGTAAGCCCGGCATGGATCGTTCCGACCTGTTTAATGTGAATGCGGGTATCGTGAAGAACCTCGTGCAGCAGATTGCCAAAACCTGCCCGCAGGCCTGCATCGGCATTATCACCAACCCGGTGAATACCACCGTGGCTATCGCCGCCGAAGTACTGAAAAAAGCCGGCGTGTACGATAAAAACAAACTGTTCGGCGTTACCACGCTGGACATCATCCGTTCCAATACCTTTGTGGCGGAGCTGAAAGGTAAATCGGCAACCGAGGTGGAAGTCCCGGTCATTGGTGGTCACTCCGGGGTCACCATTCTGCCTTTACTGTCGCAGATCCCCGGCGTCAGCTTTAGCGATCAGGAAATTGCCGACCTGACTAAACGTATTCAGAACGCCGGTACCGAAGTCGTGGAAGCGAAAGCGGGCGGCGGGTCGGCGACCTTGTCGATGGGCCAGGCGGCTGCCCGTTTTGGTCTCTCTCTGGTTCGCGCCATGCAGGGGGAAAAAGGCGTGGTGGAGTGCGCCTACGTGGAAGGCGACGGCCACTATGCGCGTTTCTTCTCCCAGCCGCTGCTGTGGAAAAAAAAACCGAGA

>pgi

TCATAGTTGCGGGCGGCTCTGGTCACACTTCTTCGGTGCGGAACCGAAGCGATTCTGCCGTACGACCAGTACATGCACCGCTTTGCCGCTTACTTCCAGCAGGGCAACATGGAGTCCAACGGTAAGTATGTTGACCGTAACGGCCACGCGGTAGACTACCAGACTGGCCCAATCATCTGGGGTGAGCCGGGCACCAACGGTCAGCACGCGTTCTACCAGCTGATCCACCAGGGCACCAAAATGGTACCGTGCGATTTCATCGCTCCGGCTATCACCCACAACCCGCTGTCTGACCACCATCAGAAACTGCTGTCTAACTTCTTCGCCCAGACCGAGGCCCTGGCCTTTGGTAAATCCCGCGAAGTGGTTGAGCAGGAATATCGCGATCAGGGTAAAGACCCGGCGACCCTGGAGCACGTGGTGCCGTTCAAAGTGTTCGAAGGTAACCGCCCGACTAACTCCATCCTGCTGCGTGAGATCACCCCGTTCAGCCTCGGGGCGCTGATTGCCCTGTACGAGCACAAAATCTTCACCCAGGGCGCGATCCTCAACATCTTCACCTTTGACCAGTGGGGCGTTGAGCTGGGCAAACAGCTGGCTAACCGCATCCTGCCGGAGCTGAAAGACGGCAGCGAAGTTAGCAGCCACGACAGCTCTACTAACGGCCTGATTAACCGCTTAGGGGGGGGGGCGGGGGA

>phoE

AGGACATAGCTCTGTACTGAATCTGACCTGCAGTACCAGGGTAAAACGAAGGCCGTGAAGCGAAGAAACAGAACGGCGACGGCGTCGGCACCTCGTTAAGCTATGATTTCGGCGGCAGCGACTTCGCCGTCAGCGCAGCCTACACCAGCTCCGACCGTACCAACGATCAGAACCTGCTGGCCCGCGGCCAGGGTTCGAAAGCGGAAGCCTGGGCGACCGGCCTGAAATATGACGCCAACAATATCTACCTGGCGACCATGTACTCTGAAACCCGCAAGATGACCCCGATCAGCGGCGGCTTTGCCAACAAAGCGCAGAACTTTGAAGCGGTGGCGCAGTATCAGTTCGACTTCGGTCTGCGTCCGTCCCTCGGCTATGTGCTGTCGAAAGGGAAGGATATCGAAGGGGTGGGGAGTGAAGATCTGGTTAACTACATCGACGTGGGCCTGACCTACTACTTCAACAAAAACATGAACGCCTTCGTGGATTACAAAATCAACCAGCTGAAAAGCGATAACAAACTCGGCATCAACGATGACGACATCGTCGCGCTGGGTATCACCTACCGTTTTTTGGATCAA

>infB

ATAGGTTGCTCAGCGAGCGGGTGGTATTACCCAGCACATCGGTGCTTACCACGTCGAAACCGACAACGGCATGATCACCTTCCTGGATACCCCGGGCCACGCCGCGTTTACCTCCATGCGTGCTCGTGGCGCGCAGGCGACGGATATCGTGGTTCTGGTGGTGGCGGCAGACGACGGCGTGATGCCGCAGACTATCGAAGCTATCCAGCACGCTAAAGCGGCGCAGGTACCGGTGGTAGTGGCGGTGAACAAGATCGATAAGCCAGAAGCCGATCCGGATCGCGTGAAGAACGAACTGTCCCAGTACGGCATCCTGCCGGAAGAGTGGGGCGGCGAGAGCCAGTTTGTCCACGTTTCCGCGAAAGCGGGTACCGGCATCGACGACCTGCTGGACGCGATCCTGCTGCAGGCTGAAGTTCTTGGCTTTGAAAGCGA

>tonB

GCGTCCGCGTCTCGACGCAGCCGATAGAGATCACAATGGTGGCGCCGGCCGATCTTGAGCCGCCTCCGGCGGCGCAGCCTGTCGTGGAGCCCGTTGTTGAACCCGAACCTGAGCCGGAGCCAGAGGTAGCGCCTGAACCGCCGAAAGAGGCGCCGGTGGTGATCCATAAACCGGAACCTAAGCCGAAGCCCAAACCTAAACCCAAGCCTAAGCCGGAGAAAAAGGTTGAACAGCCGAAGCGGGAAGTGAAGCCGGCAGCAGAGCCGCGTCCGGCCTCGCCGTTTGAAAACAACAATACGGCGCCGGCGCGTACAGCGCCAAGTACCTCGACCGCAGCGGCTAAACCCACCGTTACTGCTCCGAGCGGCCCGCGGGCGATCAGCCGCGTTCAGCCGTCCTATCCGCCGCGCGCTCAGGCGCTGCGCATTGAAGGGACGGTACGGGTGAAGTTTGACGTTTCGCCTGATGGCCGCATTGATAATCTACAGATCCTCTCTGCTCGCCCGGGGGGAATATA

FO08 (ST29)

>rpoB

ATGCTTGCTGTCGTGTAGAGCGTGCGGTGATGAGCGTCTGTCTCTTGGCGATCTGGATACCCTGATGCCTCAGGATATGATCAACGCCAAGCCGATTTCCGCAGCAGTGAAAGAGTTCTTTGGTTCCAGCCAGCTGTCTCAGTTTATGGACCAGAACAACCCGCTGTCTGAGATTACGCACAAACGTCGTATCTCCGCACTCGGCCCAGGCGGTCTGACCCGTGAGCGCGCAGGCTTCGAAGTTCGAGACGTACACCCGACCCACTACGGTCGCGTATGTCCGATCGAAACGCCTGAAGGTCCGAACATCGGTCTGATTAACTCCCTGTCCGTGTACGCGCAGACCAACGAATATGGCTTCCTTGAGACGCCGTATCGTAAAGTGACCGACGGTGTGGTTACTGACGAAATTCACTACCTGTCTGCTATCGAAGAAGGCAACTACGTTATCGCTCAGGCGAACTCCAACCTGGATGAAAACGGCCACTTCGTAGAAGATCTGGTTACCTGCCGTAGCAAAGGCGAATCCAGCTTGTTCAGCCGCGACCAGGTTGACTACATGGACGTATCCACCCAGCAGGTGGTATCCGTCGGTGCGTCCCTGATCCCGTTCCTGGAACACGATGACGCCAACCGTGCATTGATGGGTGCGAACATGCAACGTCAGGCGGTTCCGACTCTGCGCGCTGATAAGCCGCTGGTTGGTACCGGTATGGAACGTGCTGTTGCCGTTGACTCCGGTGTTACTGCCGTGGCTAAACGTGGCGGTACCGTTCAGTACGTGGATGCTTCCCGTATCGTTATCAAAGTTAACGAAGACGAGATGTACCCGGGCGAAGCAGTATCGACATCTATAACCTGACCAAGTACACCCGTTCTAACCAGAACACCTGCATCAACCAGATGCCTTGCGTGTCCCTGGGCGAACCTATTGAGCGCGGCGACGTGCTGGCAGACGGCCCGTCCACCGACCTCGGTGAGCTGGCGCTGGGTCAGACATGCGTGTAGCGTTCATGCCGTGAACGTCTCTTCTTTAAAAACACACACACAAAAGTGCGC

>gapA

CGGGTCCAGAAACGTTGAGTGAAGACGGTCATCTGGTCGTTAACGGTAAAAAAATCCGTGTTACCGCTGAACGTGACCCGGCTAACCTGAAGTGGGACGAAGTTGGTGTTGACGTTGTTGCTGAAGCAACCGGTATCTTCCTGACCGACGAAACCGCTCGTAAACACATCACCGCTGGCGCGAAAAAAGTCGTTCTGACTGGCCCGTCCAAAGACAACACTCCGATGTTCGTTCGCGGCGCTAACTTCGACGCTTACGCTGGCCAGGACATCGTTTCCAACGCTTCCTGCACCACCAACTGCCTGGCGCCGCTGGCTAAAGTTATCAACGACAACTTCGGTATCGTTGAAGGCCTGATGACCACCGTCCACGCTACCACCGCTACTCAGAAAACCGTTGATGGCCCGTCTCACAAAGACTGGCGCGGCGGCCGCGGCGCAGCTCAGAACATCATCCCGTCCTCTACCGGCGCTGCTAAAGCAGTAGGTAAAGTACTGCCAGAACTGAACGGCAAACTGACCGGTATGGCGTTCCGCGTTCCGACTCCGAACGTATCTGTTGTTGACCTGACCGTTCGTCTGGAAAAAGCAGCGTCCTACGAAGAAATCAAGAAAGCCATCAAGGTTTTTTTTAAAAAGAA

>mdh

CGCGAGAGATCGCTCGGTTACGCCGGGCGTGGCGGTAGATCTAAGTCATATCCCCACAGATGTAAAAATTAAAGGATTTTCCGGTGAAGACGCTACTCCGGCGCTGGAAGGCGCGGATGTAGTGCTGATCTCCGCGGGCGTGGCGCGTAAGCCCGGCATGGATCGTTCCGACCTGTTTAATGTGAATGCGGGTATCGTGAAGAACCTCGTGCAGCAGATTGCCAAAACCTGCCCGCAGGCCTGCATCGGCATTATCACCAACCCGGTGAATACCACCGTGGCTATCGCCGCCGAAGTACTGAAAAAAGCCGGCGTGTACGATAAAAACAAACTGTTCGGCGTTACCACGCTGGACATCATCCGTTCCAATACCTTTGTGGCGGAGCTGAAAGGTAAATCGGCAACCGAGGTGGAAGTCCCGGTCATTGGTGGTCACTCCGGGGTCACCATTCTGCCTTTACTGTCGCAGATCCCCGGCGTCAGCTTTAGCGATCAGGAAATTGCCGACCTGACTAAACGTATTCAGAACGCCGGTACTGAAGTCGTGGAAGCGAAAGCGGGCGGCGGGTCGGCGACCTTGTCGATGGGCCAGGCGGCTGCCCGTTTTGGTCTCTCTCTGGTTCGCGCCATGCAGGGGGAAAAAGGCGTGGTGGAGTGCGCCTACGTGGAAGGCGACGGCCACTATGCGCGTTTCTTCTCCCAGCCGCTGTGGAAAGAAAAAAACAGCGAAAA

>pgi

CACTGACATAGTCTCTGGTCACACTTCTTCGGTGCGGAACCGAAGCGATTCTGCCGTACGACCAGTACATGCACCGCTTTGCCGCTTACTTCCAGCAGGGCAACATGGAGTCCAACGGTAAGTATGTTGACCGTAACGGCCACGCGGTAGACTACCAGACTGGCCCAATCATCTGGGGTGAGCCGGGAACCAACGGTCAGCACGCGTTCTACCAGCTGATCCACCAGGGCACCAAAATGGTACCGTGCGATTTCATCGCTCCGGCTATCACCCACAACCCGCTGTCTGACCACCATCAGAAACTGCTGTCTAACTTCTTCGCCCAGACCGAGGCCCTGGCCTTTGGTAAATCCCGCGAAGTGGTTGAGCAGGAATATCGCGATCAGGGTAAAGACCCGGCGACCCTGGAGCACGTGGTGCCGTTCAAAGTGTTCGAAGGTAACCGCCCGACTAACTCCATCCTGCTGCGTGAGATCACCCCGTTCAGCCTCGGGGCGCTGATTGCCCTGTACGAGCACAAAATCTTCACCCAGGGCGCGATCCTCAACATCTTCACCTTTGACCAGTGGGGCGTTGAGCTGGGCAAACAGCTGGCTAACCGCATCCTGCCGGAGCTGAAAGACGGCAGCGAAGTTAGCAGCCACGACAGCTCTACTAACGGCCTGATTAACCGCTTACGGGGGGGGGCGGGAGA

>phoE

TAGCTTACTCAGGTCTCATCTGACCTGCAGTACCAGGGTAAAAACGAAGGCCGTGAAGCGAAGAAACAGAACGGCGACGGCGTCGGCACCTCGTTAAGCTATGATTTCGGCGGCAGCGACTTCGCCGTCAGCGCAGCCTACACCAGCTCCGACCGTACCAACGATCAGAACCTGCTGGCCCGCGGGCAGGGTTCGAAAGCGGAAGCCTGGGCGACCGGCCTGAAATATGACGCCAACAATATCTACCTGGCGACCATGTACTCTGAAACCCGCAAAATGACCCCGATCAGCGGCGGCTTTGCCAACAAAGCGCAGAACTTTGAAGCGGTGGCGCAGTATCAGTTCGACTTCGGTCTGCGTCCGTCCCTCGGCTATGTGCTGTCGAAAGGGAAGGATATCGAAGGGGTGGGGAGTGAGGATCTGGTTAACTACATTGACGTGGGCCTGACCTACTACTTCAACAAAAACATGAACGCCTTCGTGGATTACAAAATCAACCAGCTGAAAAGCGATAACAAACTCGGCATCAACGATGACGACATCGTCGCGCTGGGTATCACCTACGTTTTTGAAAAAAAACTC

>infB

GTAGTTGCCTCGGCGAGCGGGTGGTATTACCCAGCACATCGGTGCTTACCACGTCGAAACCGACAACGGCATGATCACCTTCCTGGATACCCCGGGCCACGCCGCGTTTACCTCCATGCGTGCTCGTGGCGCGCAGGCGACGGATATCGTGGTTCTGGTGGTGGCGGCAGACGACGGCGTGATGCCGCAGACTATCGAAGCTATCCAGCACGCTAAAGCGGCGCAGGTACCGGTGGTAGTGGCGGTGAACAAGATCGATAAGCCAGAAGCCGATCCGGATCGCGTGAAGAACGAACTGTCCCAGTACGGCATCCTGCCGGAAGAGTGGGGCGGCGAGAGCCAGTTTGTCCACGTTTCCGCGAAAGCGGGTACCGGCATCGACGACCTGCTGGACGCGATCCTGCTGCAGGCTGAAGTTCTTGGGTGAAAAGCGAA

>tonB

GGGCAGAGCGTCGTCGACGCAGCCGATAGAGATCACAATGGTGGCGCCGGCCGATCTTGAGCCGCCTCCGGCGGCGCAGCCTGTCGTGGAGCCCGTTGTTGAACCCGAACCTGAGCCGGAGCCAGAGGTAGCGCCTGAACCGCCGAAAGAGGCGCCGGTGGTGATCCATAAACCGGAACCTAAGCCGAAGCCCAAACCTAAACCCAAGCCTAAGCCGGAGAAAAAGGTTGAACAGCCGAAGCGGGAAGTGAAGCCGGCAGCAGAGCCGCGTCCGGCCTCGCCGTTTGAAAACAACAATACGGCGCCGGCGCGTACAGCGCCAAGTACCTCGACCGCAGCGGCTAAACCCACCGTTACTGCTCCGAGCGGCCCGCGGGCGATCAGCCGCGTTCAGCCGTCCTATCCGCCGCGCGCTCAGGCGCTGCGCATTGAAGGGACGGTACGGGTGAAGTTTGACGTTTCGCCTGATGGCCGCATTGATAATCTGCAGATCCTCTCTGCTCGCAGGGGGGAATTAA

FO09 (ST11)

>rpoB

CAGTCCGCGTGGCTGGTACGTGTAGAGCGTGCGGTGAAAGAGCGTCTGTCTCTTGGCGATCTGGATACCCTGATGCCTCAGGATATGATCAACGCCAAGCCGATTTCCGCAGCAGTGAAAGAGTTCTTTGGTTCCAGCCAGCTGTCTCAGTTTATGGACCAGAACAACCCGCTGTCTGAGATTACGCACAAACGTCGTATCTCCGCACTCGGCCCAGGCGGTCTGACCCGTGAGCGCGCAGGCTTCGAAGTTCGAGACGTACACCCGACCCACTACGGTCGCGTATGTCCGATCGAAACGCCTGAAGGTCCGAACATCGGTCTGATTAACTCCCTGTCCGTGTACGCGCAGACCAACGAATATGGCTTCCTTGAGACGCCGTATCGTAAAGTGACCAACGGTGTGGTTACTGACGAAATTCACTACCTGTCTGCTATCGAAGAAGGCAACTACGTTATCGCTCAGGCGAACTCCAACCTGGATGAAAACGGCCACTTCGTAGAAGATCTGGTTACCTGCCGTAGCAAAGGCGAATCCAGCTTGTTCAGCCGCGACCAGGTTGACTACATGGACGTATCCACCCAGCAGGTGGTATCCGTCGGTGCGTCCCTGATCCCGTTCCTGGAACACGATGACGCCAACCGTGCATTGATGGGTGCGAACATGCAACGTCAGGCGGTTCCGACTCTGCGCGCTGATAAGCCGCTGGTTGGTACCGGTATGGAACGTGCTGTTGCCGTTGACTCCGGTGTTACTGCCGTGGCTAAACGTGGCGGTACCGTTCAGTACGTGGATGCTTCCCGTATCGTTATCAAAGTTAACGAAGACGAGATGTACCCGGGCGAAGCAGGTATCGACATCTATAACCTGACCAAGTACACCCGTTCTAACCAGAACACCTGCATCAACCAGATGCCTTGCGTGTCCCTGGGCGAACCTATTGAGCGCGGCGACGTGCTGGCAGACGGCCCGTCCACCGACCTCGGTGAGCTGGCGCTGGGTCAGAACATGCGTGTAGCGTTCATGCCG

>gapA

TTTGAAATTATGGCTCCACTCACGGTCGTTTCGACGGTACCGTTGAAGTGAAAGACGGTCATCTGGTCGTTAACGGTAAAAAAATCCGTGTTACCGCTGAACGTGACCCGGCTAACCTGAAGTGGGACGAAGTTGGTGTTGACGTTGTTGCTGAAGCAACCGGTATCTTCCTGACCGACGAAACCGCTCGTAAACACATCACCGCTGGCGCGAAAAAAGTCGTTCTGACTGGCCCGTCCAAAGACAACACTCCGATGTTCGTTCGCGGCGCTAACTTCGACGCTTACGCTGGCCAGGACATCGTTTCCAACGCTTCCTGCACCACTAACTGCCTGGCGCCGCTGGCTAAAGTTATCAACGACAACTTCGGTATCGTTGAAGGCCTGATGACCACCGTCCACGCTACCACCGCTACTCAGAAAACCGTTGATGGCCCGTCTCACAAAGACTGGCGCGGCGGCCGCGGCGCAGCTCAGAACATCATCCCGTCCTCTACCGGCGCTGCTAAAGCAGTAGGTAAAGTACTGCCAGAACTGAACGGCAAACTGACCGGTATGGCGTTCCGCGTTCCAACTCCGAACGTATCTGTTGTTGACCTGACCGTTCGTCTGGAAAAAGCAGCGTCCTACGAAGAAATCAAGAAAGCCATCAAAGCCCTTTTCTGAAGA

>mdh

TCAGGTTCAGAGCTCTCGTTGTACGACATCGCTCCGGTTACGCCGGGCGTGGCGGTAGATCTAAGTCATATCCCCACAGATGTAAAAATTAAAGGATTTTCCGGTGAAGACGCTACTCCGGCGCTGGAAGGCGCGGATGTAGTGCTGATCTCCGCGGGCGTGGCGCGTAAGCCCGGCATGGATCGTTCCGACCTGTTTAATGTGAATGCGGGTATCGTGAAGAACCTCGTGCAGCAGATTGCCAAAACCTGCCCGCAGGCCTGCATCGGCATTATCACCAACCCGGTGAATACCACCGTGGCTATCGCCGCCGAAGTACTGAAAAAAGCCGGCGTGTACGATAAAAACAAACTGTTCGGCGTTACCACGCTGGACATCATCCGTTCCAATACCTTTGTGGCGGAGCTGAAAGGTAAATCGGCAACCGAGGTGGAAGTCCCGGTCATTGGTGGTCACTCCGGGGTCACCATTCTGCCTTTACTGTCGCAGATCCCCGGCGTCAGCTTTAGCGATCAGGAAATTGCCGACCTGACTAAACGTATTCAGAACGCCGGTACCGAAGTCGTGGAAGCGAAAGCGGGCGGCGGGTCGGCGACCTTGTCGATGGGCCAGGCGGCTGCCCGTTTTGGTCTCTCTCTGGTTCGCGCCATGCAGGGGGAAAAAGGCGTGGTGGAGTGCGCCTACGTGGAAGGCGACGGCCACTATGCGCGTTTCTTCTCCCAGCCGCTGCTGCTGGAAAAAAA

>pgi

AAAAAACCTCCTGTACTGCTGGCTCTGATCGGCATCTGGTACAACAACTTCTTCGGTGCGGAAACCGAAGCGATTCTGCCGTACGACCAGTACATGCACCGCTTTGCCGCTTACTTCCAGCAGGGCAACATGGAGTCCAACGGTAAGTATGTTGACCGTAACGGCCACGCGGTAGACTACCAGACTGGCCCAATCATCTGGGGTGAGCCGGGCACCAACGGTCAGCACGCGTTCTACCAGCTGATCCACCAGGGCACCAAAATGGTACCGTGCGATTTCATCGCTCCGGCTATCACCCACAACCCGCTGTCTGACCACCATCAGAAACTGCTGTCTAACTTCTTCGCCCAGACCGAGGCCCTGGCCTTTGGTAAATCCCGCGAAGTGGTTGAGCAGGAATATCGCGATCAGGGTAAAGACCCGGCGACCCTGGAGCACGTGGTGCCGTTCAAAGTGTTCGAAGGTAACCGCCCGACTAACTCCATCCTGCTGCGTGAGATCACCCCGTTCAGCCTCGGGGCGCTGATTGCCCTGTACGAGCACAAAATCTTCACCCAGGGCGCGATCCTCAACATCTTCACCTTTGACCAGTGGGGCGTTGAGCTGGGCAAACAGCTGGCTAACCGCATCCTGCCGGAGCTGAAAGACGGCAGCGAAGTTAGCAGCCACGACAGCTCTACTAACGGCCTGATTAACCGCTATAAGGGGGGGG

>phoE

CCCCCCACACCGACTTCTTCGGTCTGGTGGATGGCCTGGATCTGACCCTGCAGTACCAGGGTAAAAACGAAGGCCGTGAAGCGAAGAAACAGAACGGCGACGGCGTCGGCACCTCGTTAAGCTATGATTTCGGCGGCAGCGACTTCGCCGTCAGCGCAGCCTACACCAGCTCCGACCGTACCAACGATCAGAACCTGCTGGCCCGCGGCCAGGGTTCGAAAGCGGAAGCCTGGGCGACCGGCCTGAAATATGACGCCAACAATATCTACCTGGCGACCATGTACTCTGAAACCCGCAAGATGACCCCGATCAGCGGCGGCTTTGCCAACAAAGCGCAGAACTTTGAAGCGGTGGCGCAGTATCAGTTCGACTTCGGTCTGCGTCCGTCCCTCGGCTATGTGCTGTCGAAAGGGAAGGATATCGAAGGGGTGGGGAGTGAAGATCTGGTTAACTACATCGACGTGGGCCTGACCTACTACTTCAACAAAAACATGAACGCCTTCGTGGATTACAAAATCAACCAGCTGAAAAGCGATAACAAACTCGGCATCAACGATGACGACATCGTCGCGCTGGGTATCACC

>infB

ATATTCGTTCTACCAAGGTTGCCTCCGGCGAAGCGGGTGGTATTACCCAGCACATCGGTGCTTACCACGTCGAAACCGACAACGGCATGATCACCTTCCTGGATACCCCGGGCCACGCCGCGTTTACCTCCATGCGTGCTCGTGGCGCGCAGGCGACGGATATCGTGGTTCTGGTGGTGGCGGCAGACGACGGCGTGATGCCGCAGACTATCGAAGCTATCCAGCACGCTAAAGCGGCGCAGGTACCGGTGGTAGTGGCGGTGAACAAGATCGATAAGCCAGAAGCCGATCCGGATCGCGTGAAGAACGAACTGTCCCAGTACGGCATCCTGCCGGAAGAGTGGGGCGGCGAGAGCCAGTTTGTCCACGTTTCCGCGAAAGCGGGTACCGGCATCGACGACCTGCTGGACGCGATCCTGCTGCAGGCTGAAGTTCTTGACCTTGAAAGCGA

>tonB

ACATCAGGTTATTGAACAGCCTTCTCCGACGCAGCCGATAGAGATCACAATGGTGGCGCCGGCCGATCTTGAGCCGCCTCCGGCGGCGCAGCCTGTCGTGGAGCCCGTTGTTGAACCCGAACCTGAGCCGGAGCCAGAGGTAGCGCCTGAACCGCCGAAAGAGGCGCCGGTGGTGATCCATAAACCGGAACCTAAGCCGAAGCCCAAACCTAAACCCAAGCCTAAGCCGGAGAAAAAGGTTGAACAGCCGAAGCGGGAAGTGAAGCCGGCAGCAGAGCCGCGTCCGGCCTCGCCGTTTGAAAACAACAATACGGCGCCGGCGCGTACAGCGCCAAGTACCTCGACCGCAGCGGCTAAACCCACCGTTACTGCTCCGAGCGGCCCGCGGGCGATCAGCCGCGTTCAGCCGTCCTATCCGCCGCGCGCTCAGGCGCTGCGCATTGAAGGGACGGTACGGGTGAAGTTTGACGTTTCGCCTGATGGCCGCATTGATAATCTGCAGATCCTCTCTGC

FO10 (ST11)

>rpoB

ATGCTGGCTGGCGTGTAGAGCGTGCGGTGAGAGCGTCTGTCTCTTGGCGATCTGGATACCCTGATGCCTCAGGATATGATCAACGCCAAGCCGATTTCCGCAGCAGTGAAAGAGTTCTTTGGTTCCAGCCAGCTGTCTCAGTTTATGGACCAGAACAACCCGCTGTCTGAGATTACGCACAAACGTCGTATCTCCGCACTCGGCCCAGGCGGTCTGACCCGTGAGCGCGCAGGCTTCGAAGTTCGAGACGTACACCCGACCCACTACGGTCGCGTATGTCCGATCGAAACGCCTGAAGGTCCGAACATCGGTCTGATTAACTCCCTGTCCGTGTACGCGCAGACCAACGAATATGGCTTCCTTGAGACGCCGTATCGTAAAGTGACCAACGGTGTGGTTACTGACGAAATTCACTACCTGTCTGCTATCGAAGAAGGCAACTACGTTATCGCTCAGGCGAACTCCAACCTGGATGAAAACGGCCACTTCGTAGAAGATCTGGTTACCTGCCGTAGCAAAGGCGAATCCAGCTTGTTCAGCCGCGACCAGGTTGACTACATGGACGTATCCACCCAGCAGGTGGTATCCGTCGGTGCGTCCCTGATCCCGTTCCTGGAACACGATGACGCCAACCGTGCATTGATGGGTGCGAACATGCAACGTCAGGCGGTTCCGACTCTGCGCGCTGATAAGCCGCTGGTTGGTACCGGTATGGAACGTGCTGTTGCCGTTGACTCCGGTGTTACTGCCGTGGCTAAACGTGGCGGTACCGTTCAGTACGTGGATGCTTCCCGTATCGTTATCAAAGTTAACGAAGACGAGATGTACCCGGGCGAAGCAGGTATCGACATCTATAACCTGACCAAGTACACCCGTTCTAACCAGAACACCTGCATCAACCAGATGCCTTGCGTGTCCCTGGGCGAACCTATTGAGCGCGGCGACGTGCTGGCAGACGGCCCGTCCACCGACCTCGGTGAGCTGGCGCTGGGTCAGAACATGCGTGTAGCGTTCATGCCGTGGAACGTCTTTTTTTAACCATA

>gapA

GGGATCCAGTCGTTGAGTGAGACGGTCATCTGGTCGTTAACGGTAAAAAAATCCGTGTTACCGCTGAACGTGACCCGGCTAACCTGAAGTGGGACGAAGTTGGTGTTGACGTTGTTGCTGAAGCAACCGGTATCTTCCTGACCGACGAAACCGCTCGTAAACACATCACCGCTGGCGCGAAAAAAGTCGTTCTGACTGGCCCGTCCAAAGACAACACTCCGATGTTCGTTCGCGGCGCTAACTTCGACGCTTACGCTGGCCAGGACATCGTTTCCAACGCTTCCTGCACCACTAACTGCCTGGCGCCGCTGGCTAAAGTTATCAACGACAACTTCGGTATCGTTGAAGGCCTGATGACCACCGTCCACGCTACCACCGCTACTCAGAAAACCGTTGATGGCCCGTCTCACAAAGACTGGCGCGGCGGCCGCGGCGCAGCTCAGAACATCATCCCGTCCTCTACCGGCGCTGCTAAAGCAGTAGGTAAAGTACTGCCAGAACTGAACGGCAAACTGACCGGTATGGCGTTCCGCGTTCCAACTCCGAACGTATCTGTTGTTGACCTGACCGTTCGTCTGGAAAAAGCAGCGTCCTACGAAGAAATCAAGAAAGCCATCAAGCTTTTTCTCTAGAAGAGA

>mdh

GCGAGCGACTCGCTCGGTTACGCCGGGCGTGGCGGTAGATCTAAGTCATATCCCCACAGATGTAAAAATTAAAGGATTTTCCGGTGAAGACGCTACTCCGGCGCTGGAAGGCGCGGATGTAGTGCTGATCTCCGCGGGCGTGGCGCGTAAGCCCGGCATGGATCGTTCCGACCTGTTTAATGTGAATGCGGGTATCGTGAAGAACCTCGTGCAGCAGATTGCCAAAACCTGCCCGCAGGCCTGCATCGGCATTATCACCAACCCGGTGAATACCACCGTGGCTATCGCCGCCGAAGTACTGAAAAAAGCCGGCGTGTACGATAAAAACAAACTGTTCGGCGTTACCACGCTGGACATCATCCGTTCCAATACCTTTGTGGCGGAGCTGAAAGGTAAATCGGCAACCGAGGTGGAAGTCCCGGTCATTGGTGGTCACTCCGGGGTCACCATTCTGCCTTTACTGTCGCAGATCCCCGGCGTCAGCTTTAGCGATCAGGAAATTGCCGACCTGACTAAACGTATTCAGAACGCCGGTACCGAAGTCGTGGAAGCGAAAGCGGGCGGCGGGTCGGCGACCTTGTCGATGGGCCAGGCGGCTGCCCGTTTTGGTCTCTCTCTGGTTCGCGCCATGCAGGGGGAAAAAGGCGTGGTGGAGTGCGCCTACGTGGAAGGCGACGGCCACTATGCGCGTTTCTTCTCCCAGCCGCTGCTGGGAAAAAAAAAAACGAAAA

>pgi

CCGTACGGACGCATCTGGTCACACTTCTTCGGTGCGGAACCGAAGCGATTCTGCCGTACGACCAGTACATGCACCGCTTTGCCGCTTACTTCCAGCAGGGCAACATGGAGTCCAACGGTAAGTATGTTGACCGTAACGGCCACGCGGTAGACTACCAGACTGGCCCAATCATCTGGGGTGAGCCGGGCACCAACGGTCAGCACGCGTTCTACCAGCTGATCCACCAGGGCACCAAAATGGTACCGTGCGATTTCATCGCTCCGGCTATCACCCACAACCCGCTGTCTGACCACCATCAGAAACTGCTGTCTAACTTCTTCGCCCAGACCGAGGCCCTGGCCTTTGGTAAATCCCGCGAAGTGGTTGAGCAGGAATATCGCGATCAGGGTAAAGACCCGGCGACCCTGGAGCACGTGGTGCCGTTCAAAGTGTTCGAAGGTAACCGCCCGACTAACTCCATCCTGCTGCGTGAGATCACCCCGTTCAGCCTCGGGGCGCTGATTGCCCTGTACGAGCACAAAATCTTCACCCAGGGCGCGATCCTCAACATCTTCACCTTTGACCAGTGGGGCGTTGAGCTGGGCAAACAGCTGGCTAACCGCATCCTGCCGGAGCTGAAAGACGGCAGCGAAGTTAGCAGCCACGACAGCTCTACTAACGGCCTGATTAACCGCTTAGGGGGGGGGGGGCGCACAGA

>phoE

GAGGCATACTCTGCTAATCTGACCTGCAGTACCAGGGTAAAAACGAAGGCCGTGAAGCGAAGAAACAGAACGGCGACGGCGTCGGCACCTCGTTAAGCTATGATTTCGGCGGCAGCGACTTCGCCGTCAGCGCAGCCTACACCAGCTCCGACCGTACCAACGATCAGAACCTGCTGGCCCGCGGCCAGGGTTCGAAAGCGGAAGCCTGGGCGACCGGCCTGAAATATGACGCCAACAATATCTACCTGGCGACCATGTACTCTGAAACCCGCAAGATGACCCCGATCAGCGGCGGCTTTGCCAACAAAGCGCAGAACTTTGAAGCGGTGGCGCAGTATCAGTTCGACTTCGGTCTGCGTCCGTCCCTCGGCTATGTGCTGTCGAAAGGGAAGGATATCGAAGGGGTGGGGAGTGAAGATCTGGTTAACTACATCGACGTGGGCCTGACCTACTACTTCAACAAAAACATGAACGCCTTCGTGGATTACAAAATCAACCAGCTGAAAAGCGATAACAAACTCGGCATCAACGATGACGACATCGTCGCGCTGGGTATCACCTACGTTTTTAAAAAAAA

>infB

ATACACGTTCCTCCGGCGAGCGGGTGGTATTACCCAGCACATCGGTGCTTACCACGTCGAAACCGACAACGGCATGATCACCTTCCTGGATACCCCGGGCCACGCCGCGTTTACCTCCATGCGTGCTCGTGGCGCGCAGGCGACGGATATCGTGGTTCTGGTGGTGGCGGCAGACGACGGCGTGATGCCGCAGACTATCGAAGCTATCCAGCACGCTAAAGCGGCGCAGGTACCGGTGGTAGTGGCGGTGAACAAGATCGATAAGCCAGAAGCCGATCCGGATCGCGTGAAGAACGAACTGTCCCAGTACGGCATCCTGCCGGAAGAGTGGGGCGGCGAGAGCCAGTTTGTCCACGTTTCCGCGAAAGCGGGTACCGGCATCGACGACCTGCTGGACGCGATCCTGCTGCAGGCTGAAGTTCTTAGATGAAAAAGGCGA

>tonB

AGGTAGGCGTCTCGACGCAGCCGATAGAGATCACAATGGTGGCGCCGGCCGATCTTGAGCCGCCTCCGGCGGCGCAGCCTGTCGTGGAGCCCGTTGTTGAACCCGAACCTGAGCCGGAGCCAGAGGTAGCGCCTGAACCGCCGAAAGAGGCGCCGGTGGTGATCCATAAACCGGAACCTAAGCCGAAGCCCAAACCTAAACCCAAGCCTAAGCCGGAGAAAAAGGTTGAACAGCCGAAGCGGGAAGTGAAGCCGGCAGCAGAGCCGCGTCCGGCCTCGCCGTTTGAAAACAACAATACGGCGCCGGCGCGTACAGCGCCAAGTACCTCGACCGCAGCGGCTAAACCCACCGTTACTGCTCCGAGCGGCCCGCGGGCGATCAGCCGCGTTCAGCCGTCCTATCCGCCGCGCGCTCAGGCGCTGCGCATTGAAGGGACGGTACGGGTGAAGTTTGACGTTTCGCCTGATGGCCGCATTGATAATCTACAGATCCTCTCTGCTCCCCCGGGGGAAATAA

FO11 (ST11)

>rpoB

ATACTGCTGTCGTGTAGAGCGTGCGGTGaATGAGCGTCTGTCTCTTGGCGATCTGGATACCCTGATGCCTCAGGATATGATCAACGCCAAGCCGATTTCCGCAGCAGTGAAAGAGTTCTTTGGTTCCAGCCAGCTGTCTCAGTTTATGGACCAGAACAACCCGCTGTCTGAGATTACGCACAAACGTCGTATCTCCGCACTCGGCCCAGGCGGTCTGACCCGTGAGCGCGCAGGCTTCGAAGTTCGAGACGTACACCCGACCCACTACGGTCGCGTATGTCCGATCGAAACGCCTGAAGGTCCGAACATCGGTCTGATTAACTCCCTGTCCGTGTACGCGCAGACCAACGAATATGGCTTCCTTGAGACGCCGTATCGTAAAGTGACCAACGGTGTGGTTACTGACGAAATTCACTACCTGTCTGCTATCGAAGAAGGCAACTACGTTATCGCTCAGGCGAACTCCAACCTGGATGAAAACGGCCACTTCGTAGAAGATCTGGTTACCTGCCGTAGCAAAGGCGAATCCAGCTTGTTCAGCCGCGACCAGGTTGACTACATGGACGTATCCACCCAGCAGGTGGTATCCGTCGGTGCGTCCCTGATCCCGTTCCTGGAACACGATGACGCCAACCGTGCATTGATGGGTGCGAACATGCAACGTCAGGCGGTTCCGACTCTGCGCGCTGATAAGCCGCTGGTTGGTACCGGTATGGAACGTGCTGTTGCCGTTGACTCCGGTGTTACTGCCGTGGCTAAACGTGGCGGTACCGTTCAGTACGTGGATGCTTCCCGTATCGTTATCAAAGTTAACGAAGACGAGATGTACCCGGGCGAAGCAgGTATCGACATCTATAACCTGACCAAGTACACCCGTTCTAACCAGAACACCTGCATCAACCAGATGCCTTGCGTGTCCCTGGGCGAACCTATTGAGCGCGGCGACGTGCTGGCAGACGGCCCGTCCACCGACCTCGGTGAGCTGGCGCTGGGTCAGAACATGCGTGTAGCGTTCATGCCGTGAACG

>gapA

GGGATCAGATCGATGAGTGAaGACGGTCATCTGGTCGTTAACGGTAAAAAAATCCGTGTTACCGCTGAACGTGACCCGGCTAACCTGAAGTGGGACGAAGTTGGTGTTGACGTTGTTGCTGAAGCAACCGGTATCTTCCTGACCGACGAAACCGCTCGTAAACACATCACCGCTGGCGCGAAAAAAGTCGTTCTGACTGGCCCGTCCAAAGACAACACTCCGATGTTCGTTCGCGGCGCTAACTTCGACGCTTACGCTGGCCAGGACATCGTTTCCAACGCTTCCTGCACCACTAACTGCCTGGCGCCGCTGGCTAAAGTTATCAACGACAACTTCGGTATCGTTGAAGGCCTGATGACCACCGTCCACGCTACCACCGCTACTCAGAAAACCGTTGATGGCCCGTCTCACAAAGACTGGCGCGGCGGCCGCGGCGCAGCTCAGAACATCATCCCGTCCTCTACCGGCGCTGCTAAAGCAGTAGGTAAAGTACTGCCAGAACTGAACGGCAAACTGACCGGTATGGCGTTCCGCGTTCCAACTCCGAACGTATCTGTTGTTGACCTGACCGTTCGTCTGGAAAAAGCAGCGTCCTACGAAGAAATCAAGAAAGCCATCAAAGGCTTTTTTTTTAAGAGAG

>mdh

GACGTAGAGATCGCTCGGTTACGCCGGGCGTGGCGGTAGATCTAAGTCATATCCCCACAGATGTAAAAATTAAAGGATTTTCCGGTGAAGACGCTACTCCGGCGCTGGAAGGCGCGGATGTAGTGCTGATCTCCGCGGGCGTGGCGCGTAAGCCCGGCATGGATCGTTCCGACCTGTTTAATGTGAATGCGGGTATCGTGAAGAACCTCGTGCAGCAGATTGCCAAAACCTGCCCGCAGGCCTGCATCGGCATTATCACCAACCCGGTGAATACCACCGTGGCTATCGCCGCCGAAGTACTGAAAAAAGCCGGCGTGTACGATAAAAACAAACTGTTCGGCGTTACCACGCTGGACATCATCCGTTCCAATACCTTTGTGGCGGAGCTGAAAGGTAAATCGGCAACCGAGGTGGAAGTCCCGGTCATTGGTGGTCACTCCGGGGTCACCATTCTGCCTTTACTGTCGCAGATCCCCGGCGTCAGCTTTAGCGATCAGGAAATTGCCGACCTGACTAAACGTATTCAGAACGCCGGTACCGAAGTCGTGGAAGCGAAAGCGGGCGGCGGGTCGGCGACCTTGTCGATGGGCCAGGCGGCTGCCCGTTTTGGTCTCTCTCTGGTTCGCGCCATGCAGGGGGAAAAAGGCGTGGTGGAGTGCGCCTACGTGGAAGGCGACGGCCACTATGCGCGTTTCTTCTCCCAGCCGCTGCTGTGGAAAAAAAAAGCAAAA

>pgi

CATCGTGGGTGGGTCTGGTCACACTTCTTCGGTGCGGAACCGAAGCGATTCTGCCGTACGACCAGTACATGCACCGCTTTGCCGCTTACTTCCAGCAGGGCAACATGGAGTCCAACGGTAAGTATGTTGACCGTAACGGCCACGCGGTAGACTACCAGACTGGCCCAATCATCTGGGGTGAGCCGGGCACCAACGGTCAGCACGCGTTCTACCAGCTGATCCACCAGGGCACCAAAATGGTACCGTGCGATTTCATCGCTCCGGCTATCACCCACAACCCGCTGTCTGACCACCATCAGAAACTGCTGTCTAACTTCTTCGCCCAGACCGAGGCCCTGGCCTTTGGTAAATCCCGCGAAGTGGTTGAGCAGGAATATCGCGATCAGGGTAAAGACCCGGCGACCCTGGAGCACGTGGTGCCGTTCAAAGTGTTCGAAGGTAACCGCCCGACTAACTCCATCCTGCTGCGTGAGATCACCCCGTTCAGCCTCGGGGCGCTGATTGCCCTGTACGAGCACAAAATCTTCACCCAGGGCGCGATCCTCAACATCTTCACCTTTGACCAGTGGGGCGTTGAGCTGGGCAAACAGCTGGCTAACCGCATCCTGCCGGAGCTGAAAGACGGCAGCGAAGTTAGCAGCCACGACAGCTCTACTAACGGCCTGATTAACCGCTTAGGGGGGGGGGGCGGGGGA

>phoE

GAAACATGCGGCAGTACTGCATCTGACCTGCAGTACCAGGGTAAAAACGAAGGCCGTGAAGCGAAGAAACAGAACGGCGACGGCGTCGGCACCTCGTTAAGCTATGATTTCGGCGGCAGCGACTTCGCCGTCAGCGCAGCCTACACCAGCTCCGACCGTACCAACGATCAGAACCTGCTGGCCCGCGGCCAGGGTTCGAAAGCGGAAGCCTGGGCGACCGGCCTGAAATATGACGCCAACAATATCTACCTGGCGACCATGTACTCTGAAACCCGCAAGATGACCCCGATCAGCGGCGGCTTTGCCAACAAAGCGCAGAACTTTGAAGCGGTGGCGCAGTATCAGTTCGACTTCGGTCTGCGTCCGTCCCTCGGCTATGTGCTGTCGAAAGGGAAGGATATCGAAGGGGTGGGGAGTGAAGATCTGGTTAACTACATCGACGTGGGCCTGACCTACTACTTCAACAAAAACATGAACGCCTTCGTGGATTACAAAATCAACCAGCTGAAAAGCGATAACAAACTCGGCATCAACGATGACGACATCGTCGCGCTGGGTATCACCTACGTTTTTTTAGATCAA

>infB

TACGTTGCTCGGCGAGCGGGTGGTATTACCCAGCACATCGGTGCTTACCACGTCGAAACCGACAACGGCATGATCACCTTCCTGGATACCCCGGGCCACGCCGCGTTTACCTCCATGCGTGCTCGTGGCGCGCAGGCGACGGATATCGTGGTTCTGGTGGTGGCGGCAGACGACGGCGTGATGCCGCAGACTATCGAAGCTATCCAGCACGCTAAAGCGGCGCAGGTACCGGTGGTAGTGGCGGTGAACAAGATCGATAAGCCAGAAGCCGATCCGGATCGCGTGAAGAACGAACTGTCCCAGTACGGCATCCTGCCGGAAGAGTGGGGCGGCGAGAGCCAGTTTGTCCACGTTTCCGCGAAAGCGGGTACCGGCATCGACGACCTGCTGGACGCGATCCTGCTGCAGGCTGAAGTTCTTGCCTTTGAAAGCGA

>tonB

GGGCCCTCTCGACGCAGCCGATAGAGATCACAATGGTGGCGCCGGCCGATCTTGAGCCGCCTCCGGCGGCGCAGCCTGTCGTGGAGCCCGTTGTTGAACCCGAACCTGAGCCGGAGCCAGAGGTAGCGCCTGAACCGCCGAAAGAGGCGCCGGTGGTGATCCATAAACCGGAACCTAAGCCGAAGCCCAAACCTAAACCCAAGCCTAAGCCGGAGAAAAAGGTTGAACAGCCGAAGCGGGAAGTGAAGCCGGCAGCAGAGCCGCGTCCGGCCTCGCCGTTTGAAAACAACAATACGGCGCCGGCGCGTACAGCGCCAAGTACCTCGACCGCAGCGGCTAAACCCACCGTTACTGCTCCGAGCGGCCCGCGGGCGATCAGCCGCGTTCAGCCGTCCTATCCGCCGCGCGCTCAGGCGCTGCGCATTGAAGGGACGGTACGGGTGAAGTTTGACGTTTCGCCTGATGGCCGCATTGATAATCTGCAGATCCTCTCTGCTCGCCCGGGGGCAATATA

FO12 (ST11)

>rpoB

TGCCTGCTGGCGTGTAGAGCGTGCGGTGaATGAGCGTCTGTCTCTTGGCGATCTGGATACCCTGATGCCTCAGGATATGATCAACGCCAAGCCGATTTCCGCAGCAGTGAAAGAGTTCTTTGGTTCCAGCCAGCTGTCTCAGTTTATGGACCAGAACAACCCGCTGTCTGAGATTACGCACAAACGTCGTATCTCCGCACTCGGCCCAGGCGGTCTGACCCGTGAGCGCGCAGGCTTCGAAGTTCGAGACGTACACCCGACCCACTACGGTCGCGTATGTCCGATCGAAACGCCTGAAGGTCCGAACATCGGTCTGATTAACTCCCTGTCCGTGTACGCGCAGACCAACGAATATGGCTTCCTTGAGACGCCGTATCGTAAAGTGACCAACGGTGTGGTTACTGACGAAATTCACTACCTGTCTGCTATCGAAGAAGGCAACTACGTTATCGCTCAGGCGAACTCCAACCTGGATGAAAACGGCCACTTCGTAGAAGATCTGGTTACCTGCCGTAGCAAAGGCGAATCCAGCTTGTTCAGCCGCGACCAGGTTGACTACATGGACGTATCCACCCAGCAGGTGGTATCCGTCGGTGCGTCCCTGATCCCGTTCCTGGAACACGATGACGCCAACCGTGCATTGATGGGTGCGAACATGCAACGTCAGGCGGTTCCGACTCTGCGCGCTGATAAGCCGCTGGTTGGTACCGGTATGGAACGTGCTGTTGCCGTTGACTCCGGTGTTACTGCCGTGGCTAAACGTGGCGGTACCGTTCAGTACGTGGATGCTTCCCGTATCGTTATCAAAGTTAACGAAGACGAGATGTACCCGGGCGAAGCAGGTATCGACATCTATAACCTGACCAAGTACACCCGTTCTAACCAGAACACCTGCATCAACCAGATGCCTTGCGTGTCCCTGGGCGAACCTATTGAGCGCGGCGACGTGCTGGCAGACGGCCCGTCCACCGACCTCGGTGAGCTGGCGCTGGGTCAGACATGCGTGTAGCGTTCATGCCGTGAACGGTCTTTTTCTAAAAAAACACTACTACAAAAAGGGTACT

>gapA

GCATCCAGATCGTTGAGTGaAGACGGTCATCTGGTCGTTAACGGTAAAAAAATCCGTGTTACCGCTGAACGTGACCCGGCTAACCTGAAGTGGGACGAAGTTGGTGTTGACGTTGTTGCTGAAGCAACCGGTATCTTCCTGACCGACGAAACCGCTCGTAAACACATCACCGCTGGCGCGAAAAAAGTCGTTCTGACTGGCCCGTCCAAAGACAACACTCCGATGTTCGTTCGCGGCGCTAACTTCGACGCTTACGCTGGCCAGGACATCGTTTCCAACGCTTCCTGCACCACTAACTGCCTGGCGCCGCTGGCTAAAGTTATCAACGACAACTTCGGTATCGTTGAAGGCCTGATGACCACCGTCCACGCTACCACCGCTACTCAGAAAACCGTTGATGGCCCGTCTCACAAAGACTGGCGCGGCGGCCGCGGCGCAGCTCAGAACATCATCCCGTCCTCTACCGGCGCTGCTAAAGCAGTAGGTAAAGTACTGCCAGAACTGAACGGCAAACTGACCGGTATGGCGTTCCGCGTTCCAACTCCGAACGTATCTGTTGTTGACCTGACCGTTCGTCTGGAAAAAGCAGCGTCCTACGAAGAAATCAAGAAAGCCATCAAAGGTTTTTTTTAAAAAAA

>mdh

AGCGAGTCGATCGCTCGGTTACGCCGGGCGTGGCGGTAGATCTAAGTCATATCCCCACAGATGTAAAAATTAAAGGATTTTCCGGTGAAGACGCTACTCCGGCGCTGGAAGGCGCGGATGTAGTGCTGATCTCCGCGGGCGTGGCGCGTAAGCCCGGCATGGATCGTTCCGACCTGTTTAATGTGAATGCGGGTATCGTGAAGAACCTCGTGCAGCAGATTGCCAAAACCTGCCCGCAGGCCTGCATCGGCATTATCACCAACCCGGTGAATACCACCGTGGCTATCGCCGCCGAAGTACTGAAAAAAGCCGGCGTGTACGATAAAAACAAACTGTTCGGCGTTACCACGCTGGACATCATCCGTTCCAATACCTTTGTGGCGGAGCTGAAAGGTAAATCGGCAACCGAGGTGGAAGTCCCGGTCATTGGTGGTCACTCCGGGGTCACCATTCTGCCTTTACTGTCGCAGATCCCCGGCGTCAGCTTTAGCGATCAGGAAATTGCCGACCTGACTAAACGTATTCAGAACGCCGGTACCGAAGTCGTGGAAGCGAAAGCGGGCGGCGGGTCGGCGACCTTGTCGATGGGCCAGGCGGCTGCCCGTTTTGGTCTCTCTCTGGTTCGCGCCATGCAGGGGGAAAAAGGCGTGGTGGAGTGCGCCTACGTGGAAGGCGACGGCCACTATGCGCGTTTCTTCTCCCAGCCGCTGCTGTGGAAAAAAAAAACCGAAA

>pgi

TCACAGTTGCGTGATTCGTCTGGTCACACTTCTTCGGTGCGGAACCGAAGCGATTCTGCCGTACGACCAGTACATGCACCGCTTTGCCGCTTACTTCCAGCAGGGCAACATGGAGTCCAACGGTAAGTATGTTGACCGTAACGGCCACGCGGTAGACTACCAGACTGGCCCAATCATCTGGGGTGAGCCGGGCACCAACGGTCAGCACGCGTTCTACCAGCTGATCCACCAGGGCACCAAAATGGTACCGTGCGATTTCATCGCTCCGGCTATCACCCACAACCCGCTGTCTGACCACCATCAGAAACTGCTGTCTAACTTCTTCGCCCAGACCGAGGCCCTGGCCTTTGGTAAATCCCGCGAAGTGGTTGAGCAGGAATATCGCGATCAGGGTAAAGACCCGGCGACCCTGGAGCACGTGGTGCCGTTCAAAGTGTTCGAAGGTAACCGCCCGACTAACTCCATCCTGCTGCGTGAGATCACCCCGTTCAGCCTCGGGGCGCTGATTGCCCTGTACGAGCACAAAATCTTCACCCAGGGCGCGATCCTCAACATCTTCACCTTTGACCAGTGGGGCGTTGAGCTGGGCAAACAGCTGGCTAACCGCATCCTGCCGGAGCTGAAAGACGGCAGCGAAGTTAGCAGCCACGACAGCTCTACTAACGGCCTGATTAACCGCTTACGGGGGGGGGGGGACGA

>phoE

ATAAGCGTCTCAGGTCTGATCTGACCTGCAGTACCAGGGTAAAAACGAAGGCCGTGAAGCGAAGAAACAGAACGGCGACGGCGTCGGCACCTCGTTAAGCTATGATTTCGGCGGCAGCGACTTCGCCGTCAGCGCAGCCTACACCAGCTCCGACCGTACCAACGATCAGAACCTGCTGGCCCGCGGCCAGGGTTCGAAAGCGGAAGCCTGGGCGACCGGCCTGAAATATGACGCCAACAATATCTACCTGGCGACCATGTACTCTGAAACCCGCAAGATGACCCCGATCAGCGGCGGCTTTGCCAACAAAGCGCAGAACTTTGAAGCGGTGGCGCAGTATCAGTTCGACTTCGGTCTGCGTCCGTCCCTCGGCTATGTGCTGTCGAAAGGGAAGGATATCGAAGGGGTGGGGAGTGAAGATCTGGTTAACTACATCGACGTGGGCCTGACCTACTACTTCAACAAAAACATGAACGCCTTCGTGGATTACAAAATCAACCAGCTGAAAAGCGATAACAAACTCGGCATCAACGATGACGACATCGTCGCGCTGGGTATCACCTACGTTTTTTAAAAAAAA

>infB

ATACGCGTAGCCTCCGGCGAGCGGGTGGTATTACCCAGCACATCGGTGCTTACCACGTCGAAACCGACAACGGCATGATCACCTTCCTGGATACCCCGGGCCACGCCGCGTTTACCTCCATGCGTGCTCGTGGCGCGCAGGCGACGGATATCGTGGTTCTGGTGGTGGCGGCAGACGACGGCGTGATGCCGCAGACTATCGAAGCTATCCAGCACGCTAAAGCGGCGCAGGTACCGGTGGTAGTGGCGGTGAACAAGATCGATAAGCCAGAAGCCGATCCGGATCGCGTGAAGAACGAACTGTCCCAGTACGGCATCCTGCCGGAAGAGTGGGGCGGCGAGAGCCAGTTTGTCCACGTTTCCGCGAAAGCGGGTACCGGCATCGACGACCTGCTGGACGCGATCCTGCTGCAGGCTGAAGTTTTGGCTGAAAAGCGCGACT

>tonB

GATAACGCGACGTCGACGCAGCCGATAGAGATCACAATGGTGGCGCCGGCCGATCTTGAGCCGCCTCCGGCGGCGCAGCCTGTCGTGGAGCCCGTTGTTGAACCCGAACCTGAGCCGGAGCCAGAGGTAGCGCCTGAACCGCCGAAAGAGGCGCCGGTGGTGATCCATAAACCGGAACCTAAGCCGAAGCCCAAACCTAAACCCAAGCCTAAGCCGGAGAAAAAGGTTGAACAGCCGAAGCGGGAAGTGAAGCCGGCAGCAGAGCCGCGTCCGGCCTCGCCGTTTGAAAACAACAATACGGCGCCGGCGCGTACAGCGCCAAGTACCTCGACCGCAGCGGCTAAACCCACCGTTACTGCTCCGAGCGGCCCGCGGGCGATCAGCCGCGTTCAGCCGTCCTATCCGCCGCGCGCTCAGGCGCTGCGCATTGAAGGGACGGTACGGGTGAAGTTTGACGTTTCGCCTGATGGCCGCATTGATAATCTGCAGATCCTCTCTGCTCGCCCGGGGGCGAATAAA

FO13 (ST11)

>rpoB

ATAAGACTGCTGGCGTGTAGAGCGTGCGGTGATGAGCGTCTGTCTCTTGGCGATCTGGATACCCTGATGCCTCAGGATATGATCAACGCCAAGCCGATTTCCGCAGCAGTGAAAGAGTTCTTTGGTTCCAGCCAGCTGTCTCAGTTTATGGACCAGAACAACCCGCTGTCTGAGATTACGCACAAACGTCGTATCTCCGCACTCGGCCCAGGCGGTCTGACCCGTGAGCGCGCAGGCTTCGAAGTTCGAGACGTACACCCGACCCACTACGGTCGCGTATGTCCGATCGAAACGCCTGAAGGTCCGAACATCGGTCTGATTAACTCCCTGTCCGTGTACGCGCAGACCAACGAATATGGCTTCCTTGAGACGCCGTATCGTAAAGTGACCAACGGTGTGGTTACTGACGAAATTCACTACCTGTCTGCTATCGAAGAAGGCAACTACGTTATCGCTCAGGCGAACTCCAACCTGGATGAAAACGGCCACTTCGTAGAAGATCTGGTTACCTGCCGTAGCAAAGGCGAATCCAGCTTGTTCAGCCGCGACCAGGTTGACTACATGGACGTATCCACCCAGCAGGTGGTATCCGTCGGTGCGTCCCTGATCCCGTTCCTGGAACACGATGACGCCAACCGTGCATTGATGGGTGCGAACATGCAACGTCAGGCGGTTCCGACTCTGCGCGCTGATAAGCCGCTGGTTGGTACCGGTATGGAACGTGCTGTTGCCGTTGACTCCGGTGTTACTGCCGTGGCTAAACGTGGCGGTACCGTTCAGTACGTGGATGCTTCCCGTATCGTTATCAAAGTTAACGAAGACGAGATGTACCCGGGCGAAGCAGTATCGACATCTATAACCTGACCAAGTACACCCGTTCTAACCAGAACACCTGCATCAACCAGATGCCTTGCGTGTCCCTGGGCGAACCTATTGAGCGCGGCGACGTGCTGGCAGACGGCCCGTCCACCGACCTCGGTGAGCTGGCGCTGGGTCAGAACATGCGTGTAGCG

>gapA

GACATCCAGTCGTTGAGTGAGACGGTCATCTGGTCGTTAACGGTAAAAAAATCCGTGTTACCGCTGAACGTGACCCGGCTAACCTGAAGTGGGACGAAGTTGGTGTTGACGTTGTTGCTGAAGCAACCGGTATCTTCCTGACCGACGAAACCGCTCGTAAACACATCACCGCTGGCGCGAAAAAAGTCGTTCTGACTGGCCCGTCCAAAGACAACACTCCGATGTTCGTTCGCGGCGCTAACTTCGACGCTTACGCTGGCCAGGACATCGTTTCCAACGCTTCCTGCACCACTAACTGCCTGGCGCCGCTGGCTAAAGTTATCAACGACAACTTCGGTATCGTTGAAGGCCTGATGACCACCGTCCACGCTACCACCGCTACTCAGAAAACCGTTGATGGCCCGTCTCACAAAGACTGGCGCGGCGGCCGCGGCGCAGCTCAGAACATCATCCCGTCCTCTACCGGCGCTGCTAAAGCAGTAGGTAAAGTACTGCCAGAACTGAACGGCAAACTGACCGGTATGGCGTTCCGCGTTCCAACTCCGAACGTATCTGTTGTTGACCTGACCGTTCGTCTGGAAAAAGCAGCGTCCTACGAAGAAATCAAGAAAGCCATCAAGCTCTCTCTCTGTATGAGAGAA

>mdh

TACGAGGCGACTCGCTCGGTTACGCCGGGCGTGGCGGTAGATCTAAGTCATATCCCCACAGATGTAAAAATTAAAGGATTTTCCGGTGAAGACGCTACTCCGGCGCTGGAAGGCGCGGATGTAGTGCTGATCTCCGCGGGCGTGGCGCGTAAGCCCGGCATGGATCGTTCCGACCTGTTTAATGTGAATGCGGGTATCGTGAAGAACCTCGTGCAGCAGATTGCCAAAACCTGCCCGCAGGCCTGCATCGGCATTATCACCAACCCGGTGAATACCACCGTGGCTATCGCCGCCGAAGTACTGAAAAAAGCCGGCGTGTACGATAAAAACAAACTGTTCGGCGTTACCACGCTGGACATCATCCGTTCCAATACCTTTGTGGCGGAGCTGAAAGGTAAATCGGCAACCGAGGTGGAAGTCCCGGTCATTGGTGGTCACTCCGGGGTCACCATTCTGCCTTTACTGTCGCAGATCCCCGGCGTCAGCTTTAGCGATCAGGAAATTGCCGACCTGACTAAACGTATTCAGAACGCCGGTACCGAAGTCGTGGAAGCGAAAGCGGGCGGCGGGTCGGCGACCTTGTCGATGGGCCAGGCGGCTGCCCGTTTTGGTCTCTCTCTGGTTCGCGCCATGCAGGGGGAAAAAGGCGTGGTGGAGTGCGCCTACGTGGAAGGCGACGGCCACTATGCGCGTTTCTTCTCCCAGCCGCTGCTGTGGAGAAAAAAAAAACGAAA

>pgi

GACGGCGTGCGGTCCGCTCTGGTAACACTTCTTCGGTGCGGAACCGAAGCGATTCTGCCGTACGACCAGTACATGCACCGCTTTGCCGCTTACTTCCAGCAGGGCAACATGGAGTCCAACGGTAAGTATGTTGACCGTAACGGCCACGCGGTAGACTACCAGACTGGCCCAATCATCTGGGGTGAGCCGGGCACCAACGGTCAGCACGCGTTCTACCAGCTGATCCACCAGGGCACCAAAATGGTACCGTGCGATTTCATCGCTCCGGCTATCACCCACAACCCGCTGTCTGACCACCATCAGAAACTGCTGTCTAACTTCTTCGCCCAGACCGAGGCCCTGGCCTTTGGTAAATCCCGCGAAGTGGTTGAGCAGGAATATCGCGATCAGGGTAAAGACCCGGCGACCCTGGAGCACGTGGTGCCGTTCAAAGTGTTCGAAGGTAACCGCCCGACTAACTCCATCCTGCTGCGTGAGATCACCCCGTTCAGCCTCGGGGCGCTGATTGCCCTGTACGAGCACAAAATCTTCACCCAGGGCGCGATCCTCAACATCTTCACCTTTGACCAGTGGGGCGTTGAGCTGGGCAAACAGCTGGCTAACCGCATCCTGCCGGAGCTGAAAGACGGCAGCGAAGTTAGCAGCCACGACAGCTCTACTAACGGCCTGATTAACCGCTTAGGGGGGGGGGGCGGGGGA

>phoE

TAAAGACAGATCTGCTGCATCTGACCTGCAGTACCAGGGTAAAACGAAGGCCGTGAAGCGAAGAAACAGAACGGCGACGGCGTCGGCACCTCGTTAAGCTATGATTTCGGCGGCAGCGACTTCGCCGTCAGCGCAGCCTACACCAGCTCCGACCGTACCAACGATCAGAACCTGCTGGCCCGCGGCCAGGGTTCGAAAGCGGAAGCCTGGGCGACCGGCCTGAAATATGACGCCAACAATATCTACCTGGCGACCATGTACTCTGAAACCCGCAAGATGACCCCGATCAGCGGCGGCTTTGCCAACAAAGCGCAGAACTTTGAAGCGGTGGCGCAGTATCAGTTCGACTTCGGTCTGCGTCCGTCCCTCGGCTATGTGCTGTCGAAAGGGAAGGATATCGAAGGGGTGGGGAGTGAAGATCTGGTTAACTACATCGACGTGGGCCTGACCTACTACTTCAACAAAAACATGAACGCCTTCGTGGATTACAAAATCAACCAGCTGAAAAGCGATAACAAACTCGGCATCAACGATGACGACATCGTCGCGCTGGGTATCACCTACCGTTTTTCTGATCACAAA

>infB

ATAGTTGCTCGGCGAGCGGGTGGTATTACCCAGCACATCGGTGCTTACCACGTCGAAACCGACAACGGCATGATCACCTTCCTGGATACCCCGGGCCACGCCGCGTTTACCTCCATGCGTGCTCGTGGCGCGCAGGCGACGGATATCGTGGTTCTGGTGGTGGCGGCAGACGACGGCGTGATGCCGCAGACTATCGAAGCTATCCAGCACGCTAAAGCGGCGCAGGTACCGGTGGTAGTGGCGGTGAACAAGATCGATAAGCCAGAAGCCGATCCGGATCGCGTGAAGAACGAACTGTCCCAGTACGGCATCCTGCCGGAAGAGTGGGGCGGCGAGAGCCAGTTTGTCCACGTTTCCGCGAAAGCGGGTACCGGCATCGACGACCTGCTGGACGCGATCCTGCTGCAGGCTGAAGTTCTTGGCTTTTGAAAGCGA

>tonB

GCATAAGCATCTCGACGCAGCCGATAGAGATCACAATGGTGGCGCCGGCCGATCTTGAGCCGCCTCCGGCGGCGCAGCCTGTCGTGGAGCCCGTTGTTGAACCCGAACCTGAGCCGGAGCCAGAGGTAGCGCCTGAACCGCCGAAAGAGGCGCCGGTGGTGATCCATAAACCGGAACCTAAGCCGAAGCCCAAACCTAAACCCAAGCCTAAGCCGGAGAAAAAGGTTGAACAGCCGAAGCGGGAAGTGAAGCCGGCAGCAGAGCCGCGTCCGGCCTCGCCGTTTGAAAACAACAATACGGCGCCGGCGCGTACAGCGCCAAGTACCTCGACCGCAGCGGCTAAACCCACCGTTACTGCTCCGAGCGGCCCGCGGGCGATCAGCCGCGTTCAGCCGTCCTATCCGCCGCGCGCTCAGGCGCTGCGCATTGAAGGGACGGTACGGGTGAAGTTTGACGTTTCGCCTGATGGCCGCATTGATAATCTGCAGATCCTCTCTGCTCGCCGGGGGCAATATAA

FO14 (ST11)

>rpoB

CTAAATGCTGGCGTGTAGAGCGTGCGGTGaATGAGCGTCTGTCTCTTGGCGATCTGGATACCCTGATGCCTCAGGATATGATCAACGCCAAGCCGATTTCCGCAGCAGTGAAAGAGTTCTTTGGTTCCAGCCAGCTGTCTCAGTTTATGGACCAGAACAACCCGCTGTCTGAGATTACGCACAAACGTCGTATCTCCGCACTCGGCCCAGGCGGTCTGACCCGTGAGCGCGCAGGCTTCGAAGTTCGAGACGTACACCCGACCCACTACGGTCGCGTATGTCCGATCGAAACGCCTGAAGGTCCGAACATCGGTCTGATTAACTCCCTGTCCGTGTACGCGCAGACCAACGAATATGGCTTCCTTGAGACGCCGTATCGTAAAGTGACCAACGGTGTGGTTACTGACGAAATTCACTACCTGTCTGCTATCGAAGAAGGCAACTACGTTATCGCTCAGGCGAACTCCAACCTGGATGAAAACGGCCACTTCGTAGAAGATCTGGTTACCTGCCGTAGCAAAGGCGAATCCAGCTTGTTCAGCCGCGACCAGGTTGACTACATGGACGTATCCACCCAGCAGGTGGTATCCGTCGGTGCGTCCCTGATCCCGTTCCTGGAACACGATGACGCCAACCGTGCATTGATGGGTGCGAACATGCAACGTCAGGCGGTTCCGACTCTGCGCGCTGATAAGCCGCTGGTTGGTACCGGTATGGAACGTGCTGTTGCCGTTGACTCCGGTGTTACTGCCGTGGCTAAACGTGGCGGTACCGTTCAGTACGTGGATGCTTCCCGTATCGTTATCAAAGTTAACGAAGACGAGATGTACCCGGGCGAAGCAGTATCGACATCTATAACCTGACCAAGTACACCCGTTCTAACCAGAACACCTGCATCAACCAGATGCCTTGCGTGTCCCTGGGCGAACCTATTGAGCGCGGCGACGTGCTGGCAGACGGCCCGTCCACCGACCTCGGTGAGCTGGCGCTGGGTCAGAACATGCGTGTAGCGTTCATGCCGTGGAACGGTCTCTTTTTGGGAAATA

>gapA

GCGATCCAGATCGTTGAGTGAGACGGTCATCTGGTCGTTAACGGTAAAAAAATCCGTGTTACCGCTGAACGTGACCCGGCTAACCTGAAGTGGGACGAAGTTGGTGTTGACGTTGTTGCTGAAGCAACCGGTATCTTCCTGACCGACAAAACCGCTCGTAAACACATCACCGCTGGCGCGAAAAAAGTCGTTCTGACTGGCCCGTCCAAAGACAACACTCCGATGTTCGTTCGCGGCGCTAACTTCGACGCTTACGCTGGCCAGGACATCGTTTCCAACGCTTCCTGCACCACTAACTGCCTGGCGCCGCTGGCTAAAGTTATCAACGACAACTTCGGTATCGTTGAAGGCCTGATGACCACCGTCCACGCTACCACCGCTACTCAGAAAACCGTTGATGGCCCGTCTCACAAAGACTGGCGCGGCGGCCGCGGCGCAGCTCAGAACATCATCCCGTCCTCTACCGGCGCTGCTAAAGCAGTAGGTAAAGTACTGCCAGAACTGAACGGCAAACTGACCGGTATGGCGTTCCGCGTTCCAACTCCGAACGTATCTGTTGTTGACCTGACCGTTCGTCTGGAAAAAGCAGCGTCCTACGAAGAAATCAAGAAAGCCATCAAAGGTTTTTTTTGAAAGAA

>mdh

GCGAAGGCGACTCGCTCGGTTACGCCGGGCGTGGCGGTAGATCTAAGTCATATCCCCACAGATGTAAAAATTAAAGGATTTTCCGGTGAAGACGCTACTCCGGCGCTGGAAGGCGCGGATGTAGTGCTGATCTCCGCGGGCGTGGCGCGTAAGCCCGGCATGGATCGTTCCGACCTGTTTAATGTGAATGCGGGTATCGTGAAGAACCTCGTGCAGCAGATTGCCAAAACCTGCCCGCAGGCCTGCATCGGCATTATCACCAACCCGGTGAATACCACCGTGGCTATCGCCGCCGAAGTACTGAAAAAAGCCGGCGTGTACGATAAAAACAAACTGTTCGGCGTTACCACGCTGGACATCATCCGTTCCAATACCTTTGTGGCGGAGCTGAAAGGTAAATCGGCAACCGAGGTGGAAGTCCCGGTCATTGGTGGTCACTCCGGGGTCACCATTCTGCCTTTACTGTCGCAGATCCCCGGCGTCAGCTTTAGCGATCAGGAAATTGCCGACCTGACTAAACGTATTCAGAACGCCGGTACCGAAGTCGTGGAAGCGAAAGCGGGCGGCGGGTCGGCGACCTTGTCGATGGGCCAGGCGGCTGCCCGTTTTGGTCTCTCTCTGGTTCGCGCCATGCAGGGGGAAAAAGGCGTGGTGGAGTGCGCCTACGTGGAAGGCGACGGCCACTATGCGCGTTTCTTCTCCCAGCCGCTGCTGTGGAAAAAAAAAACCGAGA

>pgi

TCATAGTCCGGAGCTCTGTCCACTTCTTCGGTGCGGAACCGAAGCGATTCTGCCGTACGACCAGTACATGCACCGCTTTGCCGCTTACTTCCAGCAGGGCAACATGGAGTCCAACGGTAAGTATGTTGACCGTAACGGCCACGCGGTAGACTACCAGACTGGCCCAATCATCTGGGGTGAGCCGGGCACCAACGGTCAGCACGCGTTCTACCAGCTGATCCACCAGGGCACCAAAATGGTACCGTGCGATTTCATCGCTCCGGCTATCACCCACAACCCGCTGTCTGACCACCATCAGAAACTGCTGTCTAACTTCTTCGCCCAGACCGAGGCCCTGGCCTTTGGTAAATCCCGCGAAGTGGTTGAGCAGGAATATCGCGATCAGGGTAAAGACCCGGCGACCCTGGAGCACGTGGTGCCGTTCAAAGTGTTCGAAGGTAACCGCCCGACTAACTCCATCCTGCTGCGTGAGATCACCCCGTTCAGCCTCGGGGCGCTGATTGCCCTGTACGAGCACAAAATCTTCACCCAGGGCGCGATCCTCAACATCTTCACCTTTGACCAGTGGGGCGTTGAGCTGGGCAAACAGCTGGCTAACCGCATCCTGCCGGAGCTGAAAGACGGCAGCGAAGTTAGCAGCCACGACAGCTCTACTAACGGCCTGATTAACCGCTATAAGGGGGGGGGCGGGGA

>phoE

GGATCAGCAGCGGGTGGCTCAATCTGACCTGCAGTACCAGGGTAAAAACGAAGGCCGTGAAGCGAAGAAACAGAACGGCGACGGCGTCGGCACCTCGTTAAGCTATGATTTCGGCGGCAGCGACTTCGCCGTCAGCGCAGCCTACACCAGCTCCGACCGTACCAACGATCAGAACCTGCTGGCCCGCGGCCAGGGTTCGAAAGCGGAAGCCTGGGCGACCGGCCTGAAATATGACGCCAACAATATCTACCTGGCGACCATGTACTCTGAAACCCGCAAGATGACCCCGATCAGCGGCGGCTTTGCCAACAAAGCGCAGAACTTTGAAGCGGTGGCGCAGTATCAGTTCGACTTCGGTCTGCGTCCGTCCCTCGGCTATGTGCTGTCGAAAGGGAAGGATATCGAAGGGGTGGGGAGTGAAGATCTGGTTAACTACATCGACGTGGGCCTGACCTACTACTTCAACAAAAACATGAACGCCTTCGTGGATTACAAAATCAACCAGCTGAAAAGCGATAACAAACTCGGCATCAACGATGACGACATCGTCGCGCTGGGTATCACCTACCTTTTCTTGGATCAAA

>infB

ACGTGCTCGGCGAGCGGGTGGTATTACCCAGCACATCGGTGCTTACCACGTCGAAACCGACAACGGCATGATCACCTTCCTGGATACCCCGGGCCACGCCGCGTTTACCTCCATGCGTGCTCGTGGCGCGCAGGCGACGGATATCGTGGTTCTGGTGGTGGCGGCAGACGACGGCGTGATGCCGCAGACTATCGAAGCTATCCAGCACGCTAAAGCGGCGCAGGTACCGGTGGTAGTGGCGGTGAACAAGATCGATAAGCCAGAAGCCGATCCGGATCGCGTGAAGAACGAACTGTCCCAGTACGGCATCCTGCCGGAAGAGTGGGGCGGCGAGAGCCAGTTTGTCCACGTTTCCGCGAAAGCGGGTACCGGCATCGACGACCTGCTGGACGCGATCCTGCTGCAGGCTGAAGTTCTTGGCTTGGAAAGCGA

>tonB

GGGTTCGGTCTCTCGACGCAGCCGATAGAGATCACAATGGTGGCGCCGGCCGATCTTGAGCCGCCTCCGGCGGCGCAGCCTGTCGTGGAGCCCGTTGTTGAACCCGAACCTGAGCCGGAGCCAGAGGTAGCGCCTGAACCGCCGAAAGAGGCGCCGGTGGTGATCCATAAACCGGAACCTAAGCCGAAGCCCAAACCTAAACCCAAGCCTAAGCCGGAGAAAAAGGTTGAACAGCCGAAGCGGGAAGTGAAGCCGGCAGCAGAGCCGCGTCCGGCCTCGCCGTTTGAAAACAACAATACGGCGCCGGCGCGTACAGCGCCAAGTACCTCGACCGCAGCGGCTAAACCCACCGTTACTGCTCCGAGCGGCCCGCGGGCGATCAGCCGCGTTCAGCCGTCCTATCCGCCGCGCGCTCAGGCGCTGCGCATTGAAGGGACGGTACGGGTGAAGTTTGACGTTTCGCCTGATGGCCGCATTGATAATCTGCAGATCCTCTCTGCTCACCCGGGGCGAATAA

FO15 (ST11)

>rpoB

ATAACTCGCTGTCGTGTAGAGCGTGCGGTGATGAGCGTCTGTCTCTTGGCGATCTGGATACCCTGATGCCTCAGGATATGATCAACGCCAAGCCGATTTCCGCAGCAGTGAAAGAGTTCTTTGGTTCCAGCCAGCTGTCTCAGTTTATGGACCAGAACAACCCGCTGTCTGAGATTACGCACAAACGTCGTATCTCCGCACTCGGCCCAGGCGGTCTGACCCGTGAGCGCGCAGGCTTCGAAGTTCGAGACGTACACCCGACCCACTACGGTCGCGTATGTCCGATCGAAACGCCTGAAGGTCCGAACATCGGTCTGATTAACTCCCTGTCCGTGTACGCGCAGACCAACGAATATGGCTTCCTTGAGACGCCGTATCGTAAAGTGACCAACGGTGTGGTTACTGACGAAATTCACTACCTGTCTGCTATCGAAGAAGGCAACTACGTTATCGCTCAGGCGAACTCCAACCTGGATGAAAACGGCCACTTCGTAGAAGATCTGGTTACCTGCCGTAGCAAAGGCGAATCCAGCTTGTTCAGCCGCGACCAGGTTGACTACATGGACGTATCCACCCAGCAGGTGGTATCCGTCGGTGCGTCCCTGATCCCGTTCCTGGAACACGATGACGCCAACCGTGCATTGATGGGTGCGAACATGCAACGTCAGGCGGTTCCGACTCTGCGCGCTGATAAGCCGCTGGTTGGTACCGGTATGGAACGTGCTGTTGCCGTTGACTCCGGTGTTACTGCCGTGGCTAAACGTGGCGGTACCGTTCAGTACGTGGATGCTTCCCGTATCGTTATCAAAGTTAACGAAGACGAGATGTACCCGGGCGAAGCAGTATCGACATCTATAACCTGACCAAGTACACCCGTTCTAACCAGAACACCTGCATCAACCAGATGCCTTGCGTGTCCCTGGGCGAACCTATTGAGCGCGGCGACGTGCTGGCAGACGGCCCGTCCACCGACCTCGTGAGCTGCGCTGGGTCAGACATGCGTGTAGCGTTCATGCCGTGACGTATTTTCTCTTAAAGACCATCATCA

>gapA

CCGATGAGATCGTTGAATGAAGACGGTCATCTGGTCGTTAACGGTAAAAAAATCCGTGTTACCGCTGAACGTGACCCGGCTAACCTGAAGTGGGACGAAGTTGGTGTTGACGTTGTTGCTGAAGCAACCGGTATCTTCCTGACCGACAAAACCGCTCGTAAACACATCACCGCTGGCGCGAAAAAAGTCGTTCTGACTGGCCCGTCCAAAGACAACACTCCGATGTTCGTTCGCGGCGCTAACTTCGACGCTTACGCTGGCCAGGACATCGTTTCCAACGCTTCCTGCACCACTAACTGCCTGGCGCCGCTGGCTAAAGTTATCAACGACAACTTCGGTATCGTTGAAGGCCTGATGACCACCGTCCACGCTACCACCGCTACTCAGAAAACCGTTGATGGCCCGTCTCACAAAGACTGGCGCGGCGGCCGCGGCGCAGCTCAGAACATCATCCCGTCCTCTACCGGCGCTGCTAAAGCAGTAGGTAAAGTACTGCCAGAACTGAACGGCAAACTGACCGGTATGGCGTTCCGCGTTCCAACTCCGAACGTATCTGTTGTTGACCTGACCGTTCGTCTGGAAAAAGCAGCGTCCTACGAAGAAATCAAGAAAGCCATCAAAGCGTTTTTTTTAAGGA

>mdh

AGCGTTGACTCGCTCGGTTACGCCGGGCGTGGCGGTAGATCTAAGTCATATCCCCACAGATGTAAAAATTAAAGGATTTTCCGGTGAAGACGCTACTCCGGCGCTGGAAGGCGCGGATGTAGTGCTGATCTCCGCGGGCGTGGCGCGTAAGCCCGGCATGGATCGTTCCGACCTGTTTAATGTGAATGCGGGTATCGTGAAGAACCTCGTGCAGCAGATTGCCAAAACCTGCCCGCAGGCCTGCATCGGCATTATCACCAACCCGGTGAATACCACCGTGGCTATCGCCGCCGAAGTACTGAAAAAAGCCGGCGTGTACGATAAAAACAAACTGTTCGGCGTTACCACGCTGGACATCATCCGTTCCAATACCTTTGTGGCGGAGCTGAAAGGTAAATCGGCAACCGAGGTGGAAGTCCCGGTCATTGGTGGTCACTCCGGGGTCACCATTCTGCCTTTACTGTCGCAGATCCCCGGCGTCAGCTTTAGCGATCAGGAAATTGCCGACCTGACTAAACGTATTCAGAACGCCGGTACCGAAGTCGTGGAAGCGAAAGCGGGCGGCGGGTCGGCGACCTTGTCGATGGGCCAGGCGGCTGCCCGTTTTGGTCTCTCTCTGGTTCGCGCCATGCAGGGGGAAAAAGGCGTGGTGGAGTGCGCCTACGTGGAAGGCGACGGCCACTATGCGCGTTTCTTCTCCCAGCCGCTGCTGTGGAAAAAAAACGGGA

>pgi

ACAAGTCTATTCGGACCTCTCTGGTCACACTTCTTCGGTGCGGAACCGAAGCGATTCTGCCGTACGACCAGTACATGCACCGCTTTGCCGCTTACTTCCAGCAGGGCAACATGGAGTCCAACGGTAAGTATGTTGACCGTAACGGCCACGCGGTAGACTACCAGACTGGCCCAATCATCTGGGGTGAGCCGGGCACCAACGGTCAGCACGCGTTCTACCAGCTGATCCACCAGGGCACCAAAATGGTACCGTGCGATTTCATCGCTCCGGCTATCACCCACAACCCGCTGTCTGACCACCATCAGAAACTGCTGTCTAACTTCTTCGCCCAGACCGAGGCCCTGGCCTTTGGTAAATCCCGCGAAGTGGTTGAGCAGGAATATCGCGATCAGGGTAAAGACCCGGCGACCCTGGAGCACGTGGTGCCGTTCAAAGTGTTCGAAGGTAACCGCCCGACTAACTCCATCCTGCTGCGTGAGATCACCCCGTTCAGCCTCGGGGCGCTGATTGCCCTGTACGAGCACAAAATCTTCACCCAGGGCGCGATCCTCAACATCTTCACCTTTGACCAGTGGGGCGTTGAGCTGGGCAAACAGCTGGCTAACCGCATCCTGCCGGAGCTGAAAGACGGCAGCGAAGTTAGCAGCCACGACAGCTCTACTAACGGCCTGATTAACCGCTATAAGGGGGGGGGGGCGGGGA

>phoE

GCTACGAGTAACTGCTGAGTCTGGATCTGACCCTGCAGTACCAGGGTAAAAACGAAGGCCGTGAAGCGAAGAAACAGAACGGCGACGGCGTCGGCACCTCGTTAAGCTATGATTTCGGCGGCAGCGACTTCGCCGTCAGCGCAGCCTACACCAGCTCCGACCGTACCAACGATCAGAACCTGCTGGCCCGCGGCCAGGGTTCGAAAGCGGAAGCCTGGGCGACCGGCCTGAAATATGACGCCAACAATATCTACCTGGCGACCATGTACTCTGAAACCCGCAAGATGACCCCGATCAGCGGCGGCTTTGCCAACAAAGCGCAGAACTTTGAAGCGGTGGCGCAGTATCAGTTCGACTTCGGTCTGCGTCCGTCCCTCGGCTATGTGCTGTCGAAAGGGAAGGATATCGAAGGGGTGGGGAGTGAAGATCTGGTTAACTACATCGACGTGGGCCTGACCTACTACTTCAACAAAAACATGAACGCCTTCGTGGATTACAAAATCAACCAGCTGAAAAGCGATAACAAACTCGGCATCAACGATGACGACATCGTCGCGCTGGGTATCACCTACCGTTTTTTGATTACAA

>infB

GTACGGATGCTCGGCGAGCGGGTGGTATTACCCAGCACATCGGTGCTTACCACGTCGAAACCGACAACGGCATGATCACCTTCCTGGATACCCCGGGCCACGCCGCGTTTACCTCCATGCGTGCTCGTGGCGCGCAGGCGACGGATATCGTGGTTCTGGTGGTGGCGGCAGACGACGGCGTGATGCCGCAGACTATCGAAGCTATCCAGCACGCTAAAGCGGCGCAGGTACCGGTGGTAGTGGCGGTGAACAAGATCGATAAGCCAGAAGCCGATCCGGATCGCGTGAAGAACGAACTGTCCCAGTACGGCATCCTGCCGGAAGAGTGGGGCGGCGAGAGCCAGTTTGTCCACGTTTCCGCGAAAGCGGGTACCGGCATCGACGACCTGCTGGACGCGATCCTGCTGCAGGCTGAAGTTCTTGCTTTGAAAAACCGA

>tonB

ACAGAGCATCTCGACGCAGCCGATAGAGATCACAATGGTGGCGCCGGCCGATCTTGAGCCGCCTCCGGCGGCGCAGCCTGTCGTGGAGCCCGTTGTTGAACCCGAACCTGAGCCGGAGCCAGAGGTAGCGCCTGAACCGCCGAAAGAGGCGCCGGTGGTGATCCATAAACCGGAACCTAAGCCGAAGCCCAAACCTAAACCCAAGCCTAAGCCGGAGAAAAAGGTTGAACAGCCGAAGCGGGAAGTGAAGCCGGCAGCAGAGCCGCGTCCGGCCTCGCCGTTTGAAAACAACAATACGGCGCCGGCGCGTACAGCGCCAAGTACCTCGACCGCAGCGGCTAAACCCACCGTTACTGCTCCGAGCGGCCCGCGGGCGATCAGCCGCGTTCAGCCGTCCTATCCGCCGCGCGCTCAGGCGCTGCGCATTGAAGGGACGGTACGGGTGAAGTTTGACGTTTCGCCTGATGGCCGCATTGATAATCTGCAGATCCTCTCTGCTAGCCCGGGGCAAATAA

FO16 (ST11)

>rpoB

CAGTCCGCGTGGCTGGTACGTGTAGAGCGTGCGGTGAAAGAGCGTCTGTCTCTTGGCGATCTGGATACCCTGATGCCTCAGGATATGATCAACGCCAAGCCGATTTCCGCAGCAGTGAAAGAGTTCTTTGGTTCCAGCCAGCTGTCTCAGTTTATGGACCAGAACAACCCGCTGTCTGAGATTACGCACAAACGTCGTATCTCCGCACTCGGCCCAGGCGGTCTGACCCGTGAGCGCGCAGGCTTCGAAGTTCGAGACGTACACCCGACCCACTACGGTCGCGTATGTCCGATCGAAACGCCTGAAGGTCCGAACATCGGTCTGATTAACTCCCTGTCCGTGTACGCGCAGACCAACGAATATGGCTTCCTTGAGACGCCGTATCGTAAAGTGACCAACGGTGTGGTTACTGACGAAATTCACTACCTGTCTGCTATCGAAGAAGGCAACTACGTTATCGCTCAGGCGAACTCCAACCTGGATGAAAACGGCCACTTCGTAGAAGATCTGGTTACCTGCCGTAGCAAAGGCGAATCCAGCTTGTTCAGCCGCGACCAGGTTGACTACATGGACGTATCCACCCAGCAGGTGGTATCCGTCGGTGCGTCCCTGATCCCGTTCCTGGAACACGATGACGCCAACCGTGCATTGATGGGTGCGAACATGCAACGTCAGGCGGTTCCGACTCTGCGCGCTGATAAGCCGCTGGTTGGTACCGGTATGGAACGTGCTGTTGCCGTTGACTCCGGTGTTACTGCCGTGGCTAAACGTGGCGGTACCGTTCAGTACGTGGATGCTTCCCGTATCGTTATCAAAGTTAACGAAGACGAGATGTACCCGGGCGAAGCAGGTATCGACATCTATAACCTGACCAAGTACACCCGTTCTAACCAGAACACCTGCATCAACCAGATGCCTTGCGTGTCCCTGGGCGAACCTATTGAGCGCGGCGACGTGCTGGCAGACGGCCCGTCCACCGACCTCGGTGAGCTGGCGCTGGGTCAGAACATGCGTGTAGCGTTCATGCCG

>gapA

TTTGAAATTATGGCTCCACTCACGGTCGTTTCGACGGTACCGTTGAAGTGAAAGACGGTCATCTGGTCGTTAACGGTAAAAAAATCCGTGTTACCGCTGAACGTGACCCGGCTAACCTGAAGTGGGACGAAGTTGGTGTTGACGTTGTTGCTGAAGCAACCGGTATCTTCCTGACCGACGAAACCGCTCGTAAACACATCACCGCTGGCGCGAAAAAAGTCGTTCTGACTGGCCCGTCCAAAGACAACACTCCGATGTTCGTTCGCGGCGCTAACTTCGACGCTTACGCTGGCCAGGACATCGTTTCCAACGCTTCCTGCACCACTAACTGCCTGGCGCCGCTGGCTAAAGTTATCAACGACAACTTCGGTATCGTTGAAGGCCTGATGACCACCGTCCACGCTACCACCGCTACTCAGAAAACCGTTGATGGCCCGTCTCACAAAGACTGGCGCGGCGGCCGCGGCGCAGCTCAGAACATCATCCCGTCCTCTACCGGCGCTGCTAAAGCAGTAGGTAAAGTACTGCCAGAACTGAACGGCAAACTGACCGGTATGGCGTTCCGCGTTCCAACTCCGAACGTATCTGTTGTTGACCTGACCGTTCGTCTGGAAAAAGCAGCGTCCTACGAAGAAATCAAGAAAGCCATCAAAGCCCTTTTCTGAAGA

>mdh

TCAGGTTCAGAGCTCTCGTTGTACGACATCGCTCCGGTTACGCCGGGCGTGGCGGTAGATCTAAGTCATATCCCCACAGATGTAAAAATTAAAGGATTTTCCGGTGAAGACGCTACTCCGGCGCTGGAAGGCGCGGATGTAGTGCTGATCTCCGCGGGCGTGGCGCGTAAGCCCGGCATGGATCGTTCCGACCTGTTTAATGTGAATGCGGGTATCGTGAAGAACCTCGTGCAGCAGATTGCCAAAACCTGCCCGCAGGCCTGCATCGGCATTATCACCAACCCGGTGAATACCACCGTGGCTATCGCCGCCGAAGTACTGAAAAAAGCCGGCGTGTACGATAAAAACAAACTGTTCGGCGTTACCACGCTGGACATCATCCGTTCCAATACCTTTGTGGCGGAGCTGAAAGGTAAATCGGCAACCGAGGTGGAAGTCCCGGTCATTGGTGGTCACTCCGGGGTCACCATTCTGCCTTTACTGTCGCAGATCCCCGGCGTCAGCTTTAGCGATCAGGAAATTGCCGACCTGACTAAACGTATTCAGAACGCCGGTACCGAAGTCGTGGAAGCGAAAGCGGGCGGCGGGTCGGCGACCTTGTCGATGGGCCAGGCGGCTGCCCGTTTTGGTCTCTCTCTGGTTCGCGCCATGCAGGGGGAAAAAGGCGTGGTGGAGTGCGCCTACGTGGAAGGCGACGGCCACTATGCGCGTTTCTTCTCCCAGCCGCTGCTGCTGGAAAAAAA

>pgi

AAAAAACCTCCTGTACTGCTGGCTCTGATCGGCATCTGGTACAACAACTTCTTCGGTGCGGAAACCGAAGCGATTCTGCCGTACGACCAGTACATGCACCGCTTTGCCGCTTACTTCCAGCAGGGCAACATGGAGTCCAACGGTAAGTATGTTGACCGTAACGGCCACGCGGTAGACTACCAGACTGGCCCAATCATCTGGGGTGAGCCGGGCACCAACGGTCAGCACGCGTTCTACCAGCTGATCCACCAGGGCACCAAAATGGTACCGTGCGATTTCATCGCTCCGGCTATCACCCACAACCCGCTGTCTGACCACCATCAGAAACTGCTGTCTAACTTCTTCGCCCAGACCGAGGCCCTGGCCTTTGGTAAATCCCGCGAAGTGGTTGAGCAGGAATATCGCGATCAGGGTAAAGACCCGGCGACCCTGGAGCACGTGGTGCCGTTCAAAGTGTTCGAAGGTAACCGCCCGACTAACTCCATCCTGCTGCGTGAGATCACCCCGTTCAGCCTCGGGGCGCTGATTGCCCTGTACGAGCACAAAATCTTCACCCAGGGCGCGATCCTCAACATCTTCACCTTTGACCAGTGGGGCGTTGAGCTGGGCAAACAGCTGGCTAACCGCATCCTGCCGGAGCTGAAAGACGGCAGCGAAGTTAGCAGCCACGACAGCTCTACTAACGGCCTGATTAACCGCTATAAGGGGGGGG

>phoE

CCCCCCACACCGACTTCTTCGGTCTGGTGGATGGCCTGGATCTGACCCTGCAGTACCAGGGTAAAAACGAAGGCCGTGAAGCGAAGAAACAGAACGGCGACGGCGTCGGCACCTCGTTAAGCTATGATTTCGGCGGCAGCGACTTCGCCGTCAGCGCAGCCTACACCAGCTCCGACCGTACCAACGATCAGAACCTGCTGGCCCGCGGCCAGGGTTCGAAAGCGGAAGCCTGGGCGACCGGCCTGAAATATGACGCCAACAATATCTACCTGGCGACCATGTACTCTGAAACCCGCAAGATGACCCCGATCAGCGGCGGCTTTGCCAACAAAGCGCAGAACTTTGAAGCGGTGGCGCAGTATCAGTTCGACTTCGGTCTGCGTCCGTCCCTCGGCTATGTGCTGTCGAAAGGGAAGGATATCGAAGGGGTGGGGAGTGAAGATCTGGTTAACTACATCGACGTGGGCCTGACCTACTACTTCAACAAAAACATGAACGCCTTCGTGGATTACAAAATCAACCAGCTGAAAAGCGATAACAAACTCGGCATCAACGATGACGACATCGTCGCGCTGGGTATCACC

>infB

ATATTCGTTCTACCAAGGTTGCCTCCGGCGAAGCGGGTGGTATTACCCAGCACATCGGTGCTTACCACGTCGAAACCGACAACGGCATGATCACCTTCCTGGATACCCCGGGCCACGCCGCGTTTACCTCCATGCGTGCTCGTGGCGCGCAGGCGACGGATATCGTGGTTCTGGTGGTGGCGGCAGACGACGGCGTGATGCCGCAGACTATCGAAGCTATCCAGCACGCTAAAGCGGCGCAGGTACCGGTGGTAGTGGCGGTGAACAAGATCGATAAGCCAGAAGCCGATCCGGATCGCGTGAAGAACGAACTGTCCCAGTACGGCATCCTGCCGGAAGAGTGGGGCGGCGAGAGCCAGTTTGTCCACGTTTCCGCGAAAGCGGGTACCGGCATCGACGACCTGCTGGACGCGATCCTGCTGCAGGCTGAAGTTCTTGACCTTGAAAGCGA

>tonB

ACATCAGGTTATTGAACAGCCTTCTCCGACGCAGCCGATAGAGATCACAATGGTGGCGCCGGCCGATCTTGAGCCGCCTCCGGCGGCGCAGCCTGTCGTGGAGCCCGTTGTTGAACCCGAACCTGAGCCGGAGCCAGAGGTAGCGCCTGAACCGCCGAAAGAGGCGCCGGTGGTGATCCATAAACCGGAACCTAAGCCGAAGCCCAAACCTAAACCCAAGCCTAAGCCGGAGAAAAAGGTTGAACAGCCGAAGCGGGAAGTGAAGCCGGCAGCAGAGCCGCGTCCGGCCTCGCCGTTTGAAAACAACAATACGGCGCCGGCGCGTACAGCGCCAAGTACCTCGACCGCAGCGGCTAAACCCACCGTTACTGCTCCGAGCGGCCCGCGGGCGATCAGCCGCGTTCAGCCGTCCTATCCGCCGCGCGCTCAGGCGCTGCGCATTGAAGGGACGGTACGGGTGAAGTTTGACGTTTCGCCTGATGGCCGCATTGATAATCTGCAGATCCTCTCTGC

FO17 (ST11)

>rpoB

TGTGTTAGATGGTAGGAGCGTGCGGTGAGAGCGTCTGTCTCTTGGCGATCTGGATACCCTGATGCCTCAGGATATGATCAACGCCAAGCCGATTTCCGCAGCAGTGAAAGAGTTCTTTGGTTCCAGCCAGCTGTCTCAGTTTATGGACCAGAACAACCCGCTGTCTGAGATTACGCACAAACGTCGTATCTCCGCACTCGGCCCAGGCGGTCTGACCCGTGAGCGCGCAGGCTTCGAAGTTCGAGACGTACACCCGACCCACTACGGTCGCGTATGTCCGATCGAAACGCCTGAAGGTCCGAACATCGGTCTGATTAACTCCCTGTCCGTGTACGCGCAGACCAACGAATATGGCTTCCTTGAGACGCCGTATCGTAAAGTGACCAACGGTGTGGTTACTGACGAAATTCACTACCTGTCTGCTATCGAAGAAGGCAACTACGTTATCGCTCAGGCGAACTCCAACCTGGATGAAAACGGCCACTTCGTAGAAGATCTGGTTACCTGCCGTAGCAAAGGCGAATCCAGCTTGTTCAGCCGCGACCAGGTTGACTACATGGACGTATCCACCCAGCAGGTGGTATCCGTCGGTGCGTCCCTGATCCCGTTCCTGGAACACGATGACGCCAACCGTGCATTGATGGGTGCGAACATGCAACGTCAGGCGGTTCCGACTCTGCGCGCTGATAAGCCGCTGGTTGGTACCGGTATGGAACGTGCTGTTGCCGTTGACTCCGGTGTTACTGCCGTGGCTAAACGTGGCGGTACCGTTCAGTACGTGGATGCTTCCCGTATCGTTATCAAAGTTAACGAAGACGAGATGTACCCGGGCGAAGCAGGTATCGACATCTATAACCTGACCAAGTACACCCGTTCTAACCAGAACACCTGCATCAACCAGATGCCTTGCGTGTCCCTGGGCGAACCTATTGAGCGCGGCGACGTGCTGGGCAGACGGCCCGTCCACCGACCTCGGTGAGCTGGCGCTGGGTCAGAACATGCGTGTAGCGTTCATGCC

>gapA

CTGGGTTCCAGATCGTTGAGTGAGACGGTCATCTGGTCGTTAACGGTAAAAAAATCCGTGTTACCGCTGAACGTGACCCGGCTAACCTGAAGTGGGACGAAGTTGGTGTTGACGTTGTTGCTGAAGCAACCGGTATCTTCCTGACCGACGAAACCGCTCGTAAACACATCACCGCTGGCGCGAAAAAAGTCGTTCTGACTGGCCCGTCCAAAGACAACACTCCGATGTTCGTTCGCGGCGCTAACTTCGACGCTTACGCTGGCCAGGACATCGTTTCCAACGCTTCCTGCACCACTAACTGCCTGGCGCCGCTGGCTAAAGTTATCAACGACAACTTCGGTATCGTTGAAGGCCTGATGACCACCGTCCACGCTACCACCGCTACTCAGAAAACCGTTGATGGCCCGTCTCACAAAGACTGGCGCGGCGGCCGCGGCGCAGCTCAGAACATCATCCCGTCCTCTACCGGCGCTGCTAAAGCAGTAGGTAAAGTACTGCCAGAACTGAACGGCAAACTGACCGGTATGGCGTTCCGCGTTCCAACTCCGAACGTATCTGTTGTTGACCTGACCGTTCGTCTGGAAAAAGCAGCGTCCTACGAAGAAATCAAGAAAGCCATCAAAGCCCTTTTCTGAAGA

>mdh

GAGGGAGCGATCGCTCGGTTACGCCGGGCGTGGCGGTAGATCTAAGTCATATCCCCACAGATGTAAAAATTAAAGGATTTTCCGGTGAAGACGCTACTCCGGCGCTGGAAGGCGCGGATGTAGTGCTGATCTCCGCGGGCGTGGCGCGTAAGCCCGGCATGGATCGTTCCGACCTGTTTAATGTGAATGCGGGTATCGTGAAGAACCTCGTGCAGCAGATTGCCAAAACCTGCCCGCAGGCCTGCATCGGCATTATCACCAACCCGGTGAATACCACCGTGGCTATCGCCGCCGAAGTACTGAAAAAAGCCGGCGTGTACGATAAAAACAAACTGTTCGGCGTTACCACGCTGGACATCATCCGTTCCAATACCTTTGTGGCGGAGCTGAAAGGTAAATCGGCAACCGAGGTGGAAGTCCCGGTCATTGGTGGTCACTCCGGGGTCACCATTCTGCCTTTACTGTCGCAGATCCCCGGCGTCAGCTTTAGCGATCAGGAAATTGCCGACCTGACTAAACGTATTCAGAACGCCGGTACCGAAGTCGTGGAAGCGAAAGCGGGCGGCGGGTCGGCGACCTTGTCGATGGGCCAGGCGGCTGCCCGTTTTGGTCTCTCTCTGGTTCGCGCCATGCAGGGGGAAAAAGGCGTGGTGGAGTGCGCCTACGTGGAAGGCGACGGCCACTATGCGCGTTTCTTCTCCCAGCCGCTGCTGCTGGAAAAAAAACGGA

>pgi

CTGGGATTCACAACTTCTTCGGTGCGGAACCGAAGCGATTCTGCCGTACGACCAGTACATGCACCGCTTTGCCGCTTACTTCCAGCAGGGCAACATGGAGTCCAACGGTAAGTATGTTGACCGTAACGGCCACGCGGTAGACTACCAGACTGGCCCAATCATCTGGGGTGAGCCGGGCACCAACGGTCAGCACGCGTTCTACCAGCTGATCCACCAGGGCACCAAAATGGTACCGTGCGATTTCATCGCTCCGGCTATCACCCACAACCCGCTGTCTGACCACCATCAGAAACTGCTGTCTAACTTCTTCGCCCAGACCGAGGCCCTGGCCTTTGGTAAATCCCGCGAAGTGGTTGAGCAGGAATATCGCGATCAGGGTAAAGACCCGGCGACCCTGGAGCACGTGGTGCCGTTCAAAGTGTTCGAAGGTAACCGCCCGACTAACTCCATCCTGCTGCGTGAGATCACCCCGTTCAGCCTCGGGGCGCTGATTGCCCTGTACGAGCACAAAATCTTCACCCAGGGCGCGATCCTCAACATCTTCACCTTTGACCAGTGGGGCGTTGAGCTGGGCAAACAGCTGGCTAACCGCATCCTGCCGGAGCTGAAAGACGGCAGCGAAGTTAGCAGCCACGACAGCTCTACTAACGGCCTGATTAACCGCTATAAACGGGGGGGCGGCAAA

>phoE

GTCACTAGGCTGCTTGCTGAACTGACCTGCAGTACCAGGGTAAAACGAAGGCCGTGAAGCGAAGAAACAGAACGGCGACGGCGTCGGCACCTCGTTAAGCTATGATTTCGGCGGCAGCGACTTCGCCGTCAGCGCAGCCTACACCAGCTCCGACCGTACCAACGATCAGAACCTGCTGGCCCGCGGCCAGGGTTCGAAAGCGGAAGCCTGGGCGACCGGCCTGAAATATGACGCCAACAATATCTACCTGGCGACCATGTACTCTGAAACCCGCAAGATGACCCCGATCAGCGGCGGCTTTGCCAACAAAGCGCAGAACTTTGAAGCGGTGGCGCAGTATCAGTTCGACTTCGGTCTGCGTCCGTCCCTCGGCTATGTGCTGTCGAAAGGGAAGGATATCGAAGGGGTGGGGAGTGAAGATCTGGTTAACTACATCGACGTGGGCCTGACCTACTACTTCAACAAAAACATGAACGCCTTCGTGGATTACAAAATCAACCAGCTGAAAAGCGATAACAAACTCGGCATCAACGATGACGACATCGTCGCGCTGGGTATCACCTACCGTTTTCGGGATCAA

>infB

TAAAGTGCCTCGGCGAGCGGGTGGTATTACCCAGCACATCGGTGCTTACCACGTCGAAACCGACAACGGCATGATCACCTTCCTGGATACCCCGGGCCACGCCGCGTTTACCTCCATGCGTGCTCGTGGCGCGCAGGCGACGGATATCGTGGTTCTGGTGGTGGCGGCAGACGACGGCGTGATGCCGCAGACTATCGAAGCTATCCAGCACGCTAAAGCGGCGCAGGTACCGGTGGTAGTGGCGGTGAACAAGATCGATAAGCCAGAAGCCGATCCGGATCGCGTGAAGAACGAACTGTCCCAGTACGGCATCCTGCCGGAAGAGTGGGGCGGCGAGAGCCAGTTTGTCCACGTTTCCGCGAAAGCGGGTACCGGCATCGACGACCTGCTGGACGCGATCCTGCTGCAGGCTGAAGTTCTTGGCTTTTGAAAGCGA

>tonB

TCGGTTCGGCTTCTCGACGCAGCCGATAGAGATCACAATGGTGGCGCCGGCCGATCTTGAGCCGCCTCCGGCGGCGCAGCCTGTCGTGGAGCCCGTTGTTGAACCCGAACCTGAGCCGGAGCCAGAGGTAGCGCCTGAACCGCCGAAAGAGGCGCCGGTGGTGATCCATAAACCGGAACCTAAGCCGAAGCCCAAACCTAAACCCAAGCCTAAGCCGGAGAAAAAGGTTGAACAGCCGAAGCGGGAAGTGAAGCCGGCAGCAGAGCCGCGTCCGGCCTCGCCGTTTGAAAACAACAATACGGCGCCGGCGCGTACAGCGCCAAGTACCTCGACCGCAGCGGCTAAACCCACCGTTACTGCTCCGAGCGGCCCGCGGGCGATCAGCCGCGTTCAGCCGTCCTATCCGCCGCGCGCTCAGGCGCTGCGCATTGAAGGGACGGTACGGGTGAAGTTTGACGTTTCGCCTGATGGCCGCATTGATAATCTGCAGATCCTCTCTGCTCAGCCCGGCGAATAA

FO18 (ST11)

>rpoB

AAAGAAAACATGCGGTCGTGTAGAGCGTGCGGTGAAGAGCGTCTGTCTCTTGGCGATCTGGATACCCTGATGCCTCAGGATATGATCAACGCCAAGCCGATTTCCGCAGCAGTGAAAGAGTTCTTTGGTTCCAGCCAGCTGTCTCAGTTTATGGACCAGAACAACCCGCTGTCTGAGATTACGCACAAACGTCGTATCTCCGCACTCGGCCCAGGCGGTCTGACCCGTGAGCGCGCAGGCTTCGAAGTTCGAGACGTACACCCGACCCACTACGGTCGCGTATGTCCGATCGAAACGCCTGAAGGTCCGAACATCGGTCTGATTAACTCCCTGTCCGTGTACGCGCAGACCAACGAATATGGCTTCCTTGAGACGCCGTATCGTAAAGTGACCAACGGTGTGGTTACTGACGAAATTCACTACCTGTCTGCTATCGAAGAAGGCAACTACGTTATCGCTCAGGCGAACTCCAACCTGGATGAAAACGGCCACTTCGTAGAAGATCTGGTTACCTGCCGTAGCAAAGGCGAATCCAGCTTGTTCAGCCGCGACCAGGTTGACTACATGGACGTATCCACCCAGCAGGTGGTATCCGTCGGTGCGTCCCTGATCCCGTTCCTGGAACACGATGACGCCAACCGTGCATTGATGGGTGCGAACATGCAACGTCAGGCGGTTCCGACTCTGCGCGCTGATAAGCCGCTGGTTGGTACCGGTATGGAACGTGCTGTTGCCGTTGACTCCGGTGTTACTGCCGTGGCTAAACGTGGCGGTACCGTTCAGTACGTGGATGCTTCCCGTATCGTTATCAAAGTTAACGAAGACGAGATGTACCCGGGCGAAGCAGTATCGACATCTATAACCTGACCAAGTACACCCGTTCTAACCAGAACACCTGCATCAACCAGATGCCTTGCGTGTCCCTGGGCGAACCTATTGAGCGCGGCGACGTGCTGGCAGACGGCCCGTCCACCGACCTCGGTGAGCTGGCGCTGGGTCAGACATGCGTGTAGCGTTCATGCCGTGAACGGTTACATTTCTAAAAAACATCTAAAAACAATCGGGGGGGG

>gapA

GTCGTACGACGCTGCTTTTTCAGACGAACGGTCAGGTCAACAACAGATACGTTCGGAGTTGGAACGCGGAACGCCATACCGGTCAGTTTGCCGTTCAGTTCTGGCAGTACTTTACCTACTGCTTTAGCAGCGCCGGTAGAGGACGGGATGATGTTCTGAGCTGCGCCGCGGCCGCCGCGCCAGTCTTTGTGAGACGGGCCATCAACGGTTTTCTGAGTAGCGGTGGTAGCGTGGACGGTGGTCATCAGGCCTTCAACGATACCGAAGTTGTCGTTGATAACTTTAGCCAGCGGCGCCAGGCAGTTAGTGGTGCAGGAAGCGTTGGAAACGATGTCCTGGCCAGCGTAAGCGTCGAAGTTAGCGCCGCGAACGAACATCGGAGTGTTGTCTTTGGACGGGCCAGTCAGAACGACTTTTTTCGCGCCAGCGGTGATGTGTTTACGAGCGGTTTCGTCGGTCAGGAAGATACCGGTTGCTTCAGCAACAACGTCAACACCAACTTCGTCCCACTTCAGGTTAGCCGGGTCACGTTCAGCGGTAACACGGATTTTTTTACCGTTAACGACCAGATGACCGTCTTTCACTTCAACGGTACCGTCGAAACGACCGTGAGTGGAGCCATTAATTTTCAAA

>mdh

CTGCTATGTCGACTCGCTCGGTTACGCCGGGCGTGGCGGTAGATCTAAGTCATATCCCCACAGATGTAAAAATTAAAGGATTTTCCGGTGAAGACGCTACTCCGGCGCTGGAAGGCGCGGATGTAGTGCTGATCTCCGCGGGCGTGGCGCGTAAGCCCGGCATGGATCGTTCCGACCTGTTTAATGTGAATGCGGGTATCGTGAAGAACCTCGTGCAGCAGATTGCCAAAACCTGCCCGCAGGCCTGCATCGGCATTATCACCAACCCGGTGAATACCACCGTGGCTATCGCCGCCGAAGTACTGAAAAAAGCCGGCGTGTACGATAAAAACAAACTGTTCGGCGTTACCACGCTGGACATCATCCGTTCCAATACCTTTGTGGCGGAGCTGAAAGGTAAATCGGCAACCGAGGTGGAAGTCCCGGTCATTGGTGGTCACTCCGGGGTCACCATTCTGCCTTTACTGTCGCAGATCCCCGGCGTCAGCTTTAGCGATCAGGAAATTGCCGACCTGACTAAACGTATTCAGAACGCCGGTACCGAAGTCGTGGAAGCGAAAGCGGGCGGCGGGTCGGCGACCTTGTCGATGGGCCAGGCGGCTGCCCGTTTTGGTCTCTCTCTGGTTCGCGCCATGCAGGGGGAAAAAGGCGTGGTGGAGTGCGCCTACGTGGAAGGCGACGGCCACTATGCGCGTTTCTTCTCCCAGCCGCTGCTGCTGGGAAAAAAAACGGAA

>pgi

CCTTGGGTCACAACTTCTTCGGTGCGGAACCGAAGCGATTCTGCCGTACGACCAGTACATGCACCGCTTTGCCGCTTACTTCCAGCAGGGCAACATGGAGTCCAACGGTAAGTATGTTGACCGTAACGGCCACGCGGTAGACTACCAGACTGGCCCAATCATCTGGGGTGAGCCGGGCACCAACGGTCAGCACGCGTTCTACCAGCTGATCCACCAGGGCACCAAAATGGTACCGTGCGATTTCATCGCTCCGGCTATCACCCACAACCCGCTGTCTGACCACCATCAGAAACTGCTGTCTAACTTCTTCGCCCAGACCGAGGCCCTGGCCTTTGGTAAATCCCGCGAAGTGGTTGAGCAGGAATATCGCGATCAGGGTAAAGACCCGGCGACCCTGGAGCACGTGGTGCCGTTCAAAGTGTTCGAAGGTAACCGCCCGACTAACTCCATCCTGCTGCGTGAGATCACCCCGTTCAGCCTCGGGGCGCTGATTGCCCTGTACGAGCACAAAATCTTCACCCAGGGCGCGATCCTCAACATCTTCACCTTTGACCAGTGGGGCGTTGAGCTGGGCAAACAGCTGGCTAACCGCATCCTGCCGGAGCTGAAAGACGGCAGCGAAGTTAGCAGCCACGACAGCTCTACTAACGGCCTGATTAACCGCTATAAACGGGGGGGCGCGGA

>phoE

TCGCCGCCTAGCTGTTGCTGATCTGACCTGCAGTACCAGGGTAAAAACGAAGGCCGTGAAGCGAAGAAACAGAACGGCGACGGCGTCGGCACCTCGTTAAGCTATGATTTCGGCGGCAGCGACTTCGCCGTCAGCGCAGCCTACACCAGCTCCGACCGTACCAACGATCAGAACCTGCTGGCCCGCGGCCAGGGTTCGAAAGCGGAAGCCTGGGCGACCGGCCTGAAATATGACGCCAACAATATCTACCTGGCGACCATGTACTCTGAAACCCGCAAGATGACCCCGATCAGCGGCGGCTTTGCCAACAAAGCGCAGAACTTTGAAGCGGTGGCGCAGTATCAGTTCGACTTCGGTCTGCGTCCGTCCCTCGGCTATGTGCTGTCGAAAGGGAAGGATATCGAAGGGGTGGGGAGTGAAGATCTGGTTAACTACATCGACGTGGGCCTGACCTACTACTTCAACAAAAACATGAACGCCTTCGTGGATTACAAAATCAACCAGCTGAAAAGCGATAACAAACTCGGCATCAACGATGACGACATCGTCGCGCTGGGTATCACCTACCAGTTTCTGATCAA

>infB

ATACGTTGCTCGGCGAGCGGGTGGTATTACCCAGCACATCGGTGCTTACCACGTCGAAACCGACAACGGCATGATCACCTTCCTGGATACCCCGGGCCACGCCGCGTTTACCTCCATGCGTGCTCGTGGCGCGCAGGCGACGGATATCGTGGTTCTGGTGGTGGCGGCAGACGACGGCGTGATGCCGCAGACTATCGAAGCTATCCAGCACGCTAAAGCGGCGCAGGTACCGGTGGTAGTGGCGGTGAACAAGATCGATAAGCCAGAAGCCGATCCGGATCGCGTGAAGAACGAACTGTCCCAGTACGGCATCCTGCCGGAAGAGTGGGGCGGCGAGAGCCAGTTTGTCCACGTTTCCGCGAAAGCGGGTACCGGCATCGACGACCTGCTGGACGCGATCCTGCTGCAGGCTGAAGTTCTTGACCTGAAAGCGA

>tonB

TCGGTTCGGCTTCTCGACGCAGCCGATAGAGATCACAATGGTGGCGCCGGCCGATCTTGAGCCGCCTCCGGCGGCGCAGCCTGTCGTGGAGCCCGTTGTTGAACCCGAACCTGAGCCGGAGCCAGAGGTAGCGCCTGAACCGCCGAAAGAGGCGCCGGTGGTGATCCATAAACCGGAACCTAAGCCGAAGCCCAAACCTAAACCCAAGCCTAAGCCGGAGAAAAAGGTTGAACAGCCGAAGCGGGAAGTGAAGCCGGCAGCAGAGCCGCGTCCGGCCTCGCCGTTTGAAAACAACAATACGGCGCCGGCGCGTACAGCGCCAAGTACCTCGACCGCAGCGGCTAAACCCACCGTTACTGCTCCGAGCGGCCCGCGGGCGATCAGCCGCGTTCAGCCGTCCTATCCGCCGCGCGCTCAGGCGCTGCGCATTGAAGGGACGGTACGGGTGAAGTTTGACGTTTCGCCTGATGGCCGCATTGATAATCTGCAGATCCTCTCTGCTCAGCCCGGCGAATAA

FO19 (ST11)

>rpoB

TCAGAAAACATGCGGTCGTGTAGAGCGTGCGGTGAAGAGCGTCTGTCTCTTGGCGATCTGGATACCCTGATGCCTCAGGATATGATCAACGCCAAGCCGATTTCCGCAGCAGTGAAAGAGTTCTTTGGTTCCAGCCAGCTGTCTCAGTTTATGGACCAGAACAACCCGCTGTCTGAGATTACGCACAAACGTCGTATCTCCGCACTCGGCCCAGGCGGTCTGACCCGTGAGCGCGCAGGCTTCGAAGTTCGAGACGTACACCCGACCCACTACGGTCGCGTATGTCCGATCGAAACGCCTGAAGGTCCGAACATCGGTCTGATTAACTCCCTGTCCGTGTACGCGCAGACCAACGAATATGGCTTCCTTGAGACGCCGTATCGTAAAGTGACCAACGGTGTGGTTACTGACGAAATTCACTACCTGTCTGCTATCGAAGAAGGCAACTACGTTATCGCTCAGGCGAACTCCAACCTGGATGAAAACGGCCACTTCGTAGAAGATCTGGTTACCTGCCGTAGCAAAGGCGAATCCAGCTTGTTCAGCCGCGACCAGGTTGACTACATGGACGTATCCACCCAGCAGGTGGTATCCGTCGGTGCGTCCCTGATCCCGTTCCTGGAACACGATGACGCCAACCGTGCATTGATGGGTGCGAACATGCAACGTCAGGCGGTTCCGACTCTGCGCGCTGATAAGCCGCTGGTTGGTACCGGTATGGAACGTGCTGTTGCCGTTGACTCCGGTGTTACTGCCGTGGCTAAACGTGGCGGTACCGTTCAGTACGTGGATGCTTCCCGTATCGTTATCAAAGTTAACGAAGACGAGATGTACCCGGGCGAAGCAGTATCGACATCTATAACCTGACCAAGTACACCCGTTCTAACCAGAACACCTGCATCAACCAGATGCCTTGCGTGTCCCTGGGCGAACCTATTGAGCGCGGCGACGTGCTGGCAGACGGCCCGTCCACCGACCTCGGTGAGCTGGCGCTGGGTCAGACATGCGTGTAGCGTTCATGCCGTGAACGGTTACATTTCTAAAAAACATCTAAAAACAATCGGGGGCC

>gapA

GTCGTACGACGCTGCTTTTTCAGACGAACGGTCAGGTCAACAACAGATACGTTCGGAGTTGGAACGCGGAACGCCATACCGGTCAGTTTGCCGTTCAGTTCTGGCAGTACTTTACCTACTGCTTTAGCAGCGCCGGTAGAGGACGGGATGATGTTCTGAGCTGCGCCGCGGCCGCCGCGCCAGTCTTTGTGAGACGGGCCATCAACGGTTTTCTGAGTAGCGGTGGTAGCGTGGACGGTGGTCATCAGGCCTTCAACGATACCGAAGTTGTCGTTGATAACTTTAGCCAGCGGCGCCAGGCAGTTAGTGGTGCAGGAAGCGTTGGAAACGATGTCCTGGCCAGCGTAAGCGTCGAAGTTAGCGCCGCGAACGAACATCGGAGTGTTGTCTTTGGACGGGCCAGTCAGAACGACTTTTTTCGCGCCAGCGGTGATGTGTTTACGAGCGGTTTCGTCGGTCAGGAAGATACCGGTTGCTTCAGCAACAACGTCAACACCAACTTCGTCCCACTTCAGGTTAGCCGGGTCACGTTCAGCGGTAACACGGATTTTTTTACCGTTAACGACCAGATGACCGTCTTTCACTTCAACGGTACCGTCGAAACGACCGTGAGTGGAGCCATTAATTTTCATA

>mdh

AGCGCGTGACTCGCTCGGTTACGCCGGGCGTGGCGGTAGATCTAAGTCATATCCCCACAGATGTAAAAATTAAAGGATTTTCCGGTGAAGACGCTACTCCGGCGCTGGAAGGCGCGGATGTAGTGCTGATCTCCGCGGGCGTGGCGCGTAAGCCCGGCATGGATCGTTCCGACCTGTTTAATGTGAATGCGGGTATCGTGAAGAACCTCGTGCAGCAGATTGCCAAAACCTGCCCGCAGGCCTGCATCGGCATTATCACCAACCCGGTGAATACCACCGTGGCTATCGCCGCCGAAGTACTGAAAAAAGCCGGCGTGTACGATAAAAACAAACTGTTCGGCGTTACCACGCTGGACATCATCCGTTCCAATACCTTTGTGGCGGAGCTGAAAGGTAAATCGGCAACCGAGGTGGAAGTCCCGGTCATTGGTGGTCACTCCGGGGTCACCATTCTGCCTTTACTGTCGCAGATCCCCGGCGTCAGCTTTAGCGATCAGGAAATTGCCGACCTGACTAAACGTATTCAGAACGCCGGTACCGAAGTCGTGGAAGCGAAAGCGGGCGGCGGGTCGGCGACCTTGTCGATGGGCCAGGCGGCTGCCCGTTTTGGTCTCTCTCTGGTTCGCGCCATGCAGGGGGAAAAAGGCGTGGTGGAGTGCGCCTACGTGGAAGGCGACGGCCACTATGCGCGTTTCTTCTCCCAGCCGCTGCTGTGGAAAAAAAAAAACGGA

>pgi

CCTTGGGTCACAACTTCTTCGGTGCGGAACCGAAGCGATTCTGCCGTACGACCAGTACATGCACCGCTTTGCCGCTTACTTCCAGCAGGGCAACATGGAGTCCAACGGTAAGTATGTTGACCGTAACGGCCACGCGGTAGACTACCAGACTGGCCCAATCATCTGGGGTGAGCCGGGCACCAACGGTCAGCACGCGTTCTACCAGCTGATCCACCAGGGCACCAAAATGGTACCGTGCGATTTCATCGCTCCGGCTATCACCCACAACCCGCTGTCTGACCACCATCAGAAACTGCTGTCTAACTTCTTCGCCCAGACCGAGGCCCTGGCCTTTGGTAAATCCCGCGAAGTGGTTGAGCAGGAATATCGCGATCAGGGTAAAGACCCGGCGACCCTGGAGCACGTGGTGCCGTTCAAAGTGTTCGAAGGTAACCGCCCGACTAACTCCATCCTGCTGCGTGAGATCACCCCGTTCAGCCTCGGGGCGCTGATTGCCCTGTACGAGCACAAAATCTTCACCCAGGGCGCGATCCTCAACATCTTCACCTTTGACCAGTGGGGCGTTGAGCTGGGCAAACAGCTGGCTAACCGCATCCTGCCGGAGCTGAAAGACGGCAGCGAAGTTAGCAGCCACGACAGCTCTACTAACGGCCTGATTAACCGCTATAAACGGGGGGGCGCGGA

>phoE

TCCGCTAGCTGCTGAGGTCTGGATCTGACCCTGCAGTACCAGGGTAAAAACGAAGGCCGTGAAGCGAAGAAACAGAACGGCGACGGCGTCGGCACCTCGTTAAGCTATGATTTCGGCGGCAGCGACTTCGCCGTCAGCGCAGCCTACACCAGCTCCGACCGTACCAACGATCAGAACCTGCTGGCCCGCGGCCAGGGTTCGAAAGCGGAAGCCTGGGCGACCGGCCTGAAATATGACGCCAACAATATCTACCTGGCGACCATGTACTCTGAAACCCGCAAGATGACCCCGATCAGCGGCGGCTTTGCCAACAAAGCGCAGAACTTTGAAGCGGTGGCGCAGTATCAGTTCGACTTCGGTCTGCGTCCGTCCCTCGGCTATGTGCTGTCGAAAGGGAAGGATATCGAAGGGGTGGGGAGTGAAGATCTGGTTAACTACATCGACGTGGGCCTGACCTACTACTTCAACAAAAACATGAACGCCTTCGTGGATTACAAAATCAACCAGCTGAAAAGCGATAACAAACTCGGCATCAACGATGACGACATCGTCGCGCTGGGTATCACCTACCGTTTTTTGAGATACAA

>infB

ATACGTTGCTCGGCGAGCGGGTGGTATTACCCAGCACATCGGTGCTTACCACGTCGAAACCGACAACGGCATGATCACCTTCCTGGATACCCCGGGCCACGCCGCGTTTACCTCCATGCGTGCTCGTGGCGCGCAGGCGACGGATATCGTGGTTCTGGTGGTGGCGGCAGACGACGGCGTGATGCCGCAGACTATCGAAGCTATCCAGCACGCTAAAGCGGCGCAGGTACCGGTGGTAGTGGCGGTGAACAAGATCGATAAGCCAGAAGCCGATCCGGATCGCGTGAAGAACGAACTGTCCCAGTACGGCATCCTGCCGGAAGAGTGGGGCGGCGAGAGCCAGTTTGTCCACGTTTCCGCGAAAGCGGGTACCGGCATCGACGACCTGCTGGACGCGATCCTGCTGCAGGCTGAAGTTCTTGACCTGAAAGC

>tonB

TCGGTTCGGCTTCTCGACGCAGCCGATAGAGATCACAATGGTGGCGCCGGCCGATCTTGAGCCGCCTCCGGCGGCGCAGCCTGTCGTGGAGCCCGTTGTTGAACCCGAACCTGAGCCGGAGCCAGAGGTAGCGCCTGAACCGCCGAAAGAGGCGCCGGTGGTGATCCATAAACCGGAACCTAAGCCGAAGCCCAAACCTAAACCCAAGCCTAAGCCGGAGAAAAAGGTTGAACAGCCGAAGCGGGAAGTGAAGCCGGCAGCAGAGCCGCGTCCGGCCTCGCCGTTTGAAAACAACAATACGGCGCCGGCGCGTACAGCGCCAAGTACCTCGACCGCAGCGGCTAAACCCACCGTTACTGCTCCGAGCGGCCCGCGGGCGATCAGCCGCGTTCAGCCGTCCTATCCGCCGCGCGCTCAGGCGCTGCGCATTGAAGGGACGGTACGGGTGAAGTTTGACGTTTCGCCTGATGGCCGCATTGATAATCTGCAGATCCTCTCTGCTCAGCCCGGCGAACC

FO20 (ST11)

>rpoB

ACTAAAACATGCGGTCGTGTAGAGCGTGCGGTGAAGAGCGTCTGTCTCTTGGCGATCTGGATACCCTGATGCCTCAGGATATGATCAACGCCAAGCCGATTTCCGCAGCAGTGAAAGAGTTCTTTGGTTCCAGCCAGCTGTCTCAGTTTATGGACCAGAACAACCCGCTGTCTGAGATTACGCACAAACGTCGTATCTCCGCACTCGGCCCAGGCGGTCTGACCCGTGAGCGCGCAGGCTTCGAAGTTCGAGACGTACACCCGACCCACTACGGTCGCGTATGTCCGATCGAAACGCCTGAAGGTCCGAACATCGGTCTGATTAACTCCCTGTCCGTGTACGCGCAGACCAACGAATATGGCTTCCTTGAGACGCCGTATCGTAAAGTGACCAACGGTGTGGTTACTGACGAAATTCACTACCTGTCTGCTATCGAAGAAGGCAACTACGTTATCGCTCAGGCGAACTCCAACCTGGATGAAAACGGCCACTTCGTAGAAGATCTGGTTACCTGCCGTAGCAAAGGCGAATCCAGCTTGTTCAGCCGCGACCAGGTTGACTACATGGACGTATCCACCCAGCAGGTGGTATCCGTCGGTGCGTCCCTGATCCCGTTCCTGGAACACGATGACGCCAACCGTGCATTGATGGGTGCGAACATGCAACGTCAGGCGGTTCCGACTCTGCGCGCTGATAAGCCGCTGGTTGGTACCGGTATGGAACGTGCTGTTGCCGTTGACTCCGGTGTTACTGCCGTGGCTAAACGTGGCGGTACCGTTCAGTACGTGGATGCTTCCCGTATCGTTATCAAAGTTAACGAAGACGAGATGTACCCGGGCGAAGCAGTATCGACATCTATAACCTGACCAAGTACACCCGTTCTAACCAGAACACCTGCATCAACCAGATGCCTTGCGTGTCCCTGGGCGAACCTATTGAGCGCGGCGACGTGCTGGCAGACGGCCCGTCCACCGACCTCGGTGAGCTGGCGCTGGGTCAGACATGCGTGTAGCGTTCATGCCGTGAACGGTTACATTTCTAAAAAACATCTAAAAACAATCGGGGGCC

>gapA

GGGTTCAGATCGTTGAGTGAGACGGTCATCTGGTCGTTAACGGTAAAAAAATCCGTGTTACCGCTGAACGTGACCCGGCTAACCTGAAGTGGGACGAAGTTGGTGTTGACGTTGTTGCTGAAGCAACCGGTATCTTCCTGACCGACAAAACCGCTCGTAAACACATCACCGCTGGCGCGAAAAAAGTCGTTCTGACTGGCCCGTCCAAAGACAACACTCCGATGTTCGTTCGCGGCGCTAACTTCGACGCTTACGCTGGCCAGGACATCGTTTCCAACGCTTCCTGCACCACTAACTGCCTGGCGCCGCTGGCTAAAGTTATCAACGACAACTTCGGTATCGTTGAAGGCCTGATGACCACCGTCCACGCTACCACCGCTACTCAGAAAACCGTTGATGGCCCGTCTCACAAAGACTGGCGCGGCGGCCGCGGCGCAGCTCAGAACATCATCCCGTCCTCTACCGGCGCTGCTAAAGCAGTAGGTAAAGTACTGCCAGAACTGAACGGCAAACTGACCGGTATGGCGTTCCGCGTTCCAACTCCGAACGTATCTGTTGTTGACCTGACCGTTCGTCTGGAAAAAGCAGCGTCCTACGAAGAAATCAAGAAAGCCATCAAAGCGTTTTCCA

>mdh

ATCAGGTTCAGAGCTCTCGTTGTACGACATCGCTCCGGTTACGCCGGGCGTGGCGGTAGATCTAAGTCATATCCCCACAGATGTAAAAATTAAAGGATTTTCCGGTGAAGACGCTACTCCGGCGCTGGAAGGCGCGGATGTAGTGCTGATCTCCGCGGGCGTGGCGCGTAAGCCCGGCATGGATCGTTCCGACCTGTTTAATGTGAATGCGGGTATCGTGAAGAACCTCGTGCAGCAGATTGCCAAAACCTGCCCGCAGGCCTGCATCGGCATTATCACCAACCCGGTGAATACCACCGTGGCTATCGCCGCCGAAGTACTGAAAAAAGCCGGCGTGTACGATAAAAACAAACTGTTCGGCGTTACCACGCTGGACATCATCCGTTCCAATACCTTTGTGGCGGAGCTGAAAGGTAAATCGGCAACCGAGGTGGAAGTCCCGGTCATTGGTGGTCACTCCGGGGTCACCATTCTGCCTTTACTGTCGCAGATCCCCGGCGTCAGCTTTAGCGATCAGGAAATTGCCGACCTGACTAAACGTATTCAGAACGCCGGTACCGAAGTCGTGGAAGCGAAAGCGGGCGGCGGGTCGGCGACCTTGTCGATGGGCCAGGCGGCTGCCCGTTTTGGTCTCTCTCTGGTTCGCGCCATGCAGGGGGAAAAAGGCGTGGTGGAGTGCGCCTACGTGGAAGGCGACGGCCACTATGCGCGTTTCTTCTCCCAGCCGCTGCTGCTGGAAAAAC

>pgi

CTAAAAAACCTCCTGTACTGCTGGCTCTGATCGGCATCTGGTACAACAACTTCTTCGGTGCGGAAACCGAAGCGATTCTGCCGTACGACCAGTACATGCACCGCTTTGCCGCTTACTTCCAGCAGGGCAACATGGAGTCCAACGGTAAGTATGTTGACCGTAACGGCCACGCGGTAGACTACCAGACTGGCCCAATCATCTGGGGTGAGCCGGGCACCAACGGTCAGCACGCGTTCTACCAGCTGATCCACCAGGGCACCAAAATGGTACCGTGCGATTTCATCGCTCCGGCTATCACCCACAACCCGCTGTCTGACCACCATCAGAAACTGCTGTCTAACTTCTTCGCCCAGACCGAGGCCCTGGCCTTTGGTAAATCCCGCGAAGTGGTTGAGCAGGAATATCGCGATCAGGGTAAAGACCCGGCGACCCTGGAGCACGTGGTGCCGTTCAAAGTGTTCGAAGGTAACCGCCCGACTAACTCCATCCTGCTGCGTGAGATCACCCCGTTCAGCCTCGGGGCGCTGATTGCCCTGTACGAGCACAAAATCTTCACCCAGGGCGCGATCCTCAACATCTTCACCTTTGACCAGTGGGGCGTTGAGCTGGGCAAACAGCTGGCTAACCGCATCCTGCCGGAGCTGAAAGACGGCAGCGAAGTTAGCAGCCACGACAGCTCTACTAACGGCCTGATTAACCGCTATAAGGGGGGGGAC

>phoE

TCCGCTAGCTGCTGAGGTCTGGATCTGACCCTGCAGTACCAGGGTAAAAACGAAGGCCGTGAAGCGAAGAAACAGAACGGCGACGGCGTCGGCACCTCGTTAAGCTATGATTTCGGCGGCAGCGACTTCGCCGTCAGCGCAGCCTACACCAGCTCCGACCGTACCAACGATCAGAACCTGCTGGCCCGCGGCCAGGGTTCGAAAGCGGAAGCCTGGGCGACCGGCCTGAAATATGACGCCAACAATATCTACCTGGCGACCATGTACTCTGAAACCCGCAAGATGACCCCGATCAGCGGCGGCTTTGCCAACAAAGCGCAGAACTTTGAAGCGGTGGCGCAGTATCAGTTCGACTTCGGTCTGCGTCCGTCCCTCGGCTATGTGCTGTCGAAAGGGAAGGATATCGAAGGGGTGGGGAGTGAAGATCTGGTTAACTACATCGACGTGGGCCTGACCTACTACTTCAACAAAAACATGAACGCCTTCGTGGATTACAAAATCAACCAGCTGAAAAGCGATAACAAACTCGGCATCAACGATGACGACATCGTCGCGCTGGGTATCACCTACCGTTTTTTGAGATACAA

>infB

ATACGTTGCTCGGCGAGCGGGTGGTATTACCCAGCACATCGGTGCTTACCACGTCGAAACCGACAACGGCATGATCACCTTCCTGGATACCCCGGGCCACGCCGCGTTTACCTCCATGCGTGCTCGTGGCGCGCAGGCGACGGATATCGTGGTTCTGGTGGTGGCGGCAGACGACGGCGTGATGCCGCAGACTATCGAAGCTATCCAGCACGCTAAAGCGGCGCAGGTACCGGTGGTAGTGGCGGTGAACAAGATCGATAAGCCAGAAGCCGATCCGGATCGCGTGAAGAACGAACTGTCCCAGTACGGCATCCTGCCGGAAGAGTGGGGCGGCGAGAGCCAGTTTGTCCACGTTTCCGCGAAAGCGGGTACCGGCATCGACGACCTGCTGGACGCGATCCTGCTGCAGGCTGAAGTTCTTGACCTGAAAGC

>tonB

ACGTCCGCGTCTCGACGCAGCCGATAGAGATCACAATGGTGGCGCCGGCCGATCTTGAGCCGCCTCCGGCGGCGCAGCCTGTCGTGGAGCCCGTTGTTGAACCCGAACCTGAGCCGGAGCCAGAGGTAGCGCCTGAACCGCCGAAAGAGGCGCCGGTGGTGATCCATAAACCGGAACCTAAGCCGAAGCCCAAACCTAAACCCAAGCCTAAGCCGGAGAAAAAGGTTGAACAGCCGAAGCGGGAAGTGAAGCCGGCAGCAGAGCCGCGTCCGGCCTCGCCGTTTGAAAACAACAATACGGCGCCGGCGCGTACAGCGCCAAGTACCTCGACCGCAGCGGCTAAACCCACCGTTACTGCTCCGAGCGGCCCGCGGGCGATCAGCCGCGTTCAGCCGTCCTATCCGCCGCGCGCTCAGGCGCTGCGCATTGAAGGGACGGTACGGGTGAAGTTTGACGTTTCGCCTGATGGCCGCATTGATAATCTACAGATCCTCTCTGCTCGCCCGGGGGGAATACT

FO21 (ST11)

>rpoB

ATACTCGCTGTCGTGTAGAGCGTGCGGTGATGAGCGTCTGTCTCTTGGCGATCTGGATACCCTGATGCCTCAGGATATGATCAACGCCAAGCCGATTTCCGCAGCAGTGAAAGAGTTCTTTGGTTCCAGCCAGCTGTCTCAGTTTATGGACCAGAACAACCCGCTGTCTGAGATTACGCACAAACGTCGTATCTCCGCACTCGGCCCAGGCGGTCTGACCCGTGAGCGCGCAGGCTTCGAAGTTCGAGACGTACACCCGACCCACTACGGTCGCGTATGTCCGATCGAAACGCCTGAAGGTCCGAACATCGGTCTGATTAACTCCCTGTCCGTGTACGCGCAGACCAACGAATATGGCTTCCTTGAGACGCCGTATCGTAAAGTGACCAACGGTGTGGTTACTGACGAAATTCACTACCTGTCTGCTATCGAAGAAGGCAACTACGTTATCGCTCAGGCGAACTCCAACCTGGATGAAAACGGCCACTTCGTAGAAGATCTGGTTACCTGCCGTAGCAAAGGCGAATCCAGCTTGTTCAGCCGCGACCAGGTTGACTACATGGACGTATCCACCCAGCAGGTGGTATCCGTCGGTGCGTCCCTGATCCCGTTCCTGGAACACGATGACGCCAACCGTGCATTGATGGGTGCGAACATGCAACGTCAGGCGGTTCCGACTCTGCGCGCTGATAAGCCGCTGGTTGGTACCGGTATGGAACGTGCTGTTGCCGTTGACTCCGGTGTTACTGCCGTGGCTAAACGTGGCGGTACCGTTCAGTACGTGGATGCTTCCCGTATCGTTATCAAAGTTAACGAAGACGAGATGTACCCGGGCGAAGCAGTATCGACATCTATAACCTGACCAAGTACACCCGTTCTAACCAGAACACCTGCATCAACCAGATGCCTTGCGTGTCCCTGGGCGAACCTATTGAGCGCGGCGACGTGCTGGCAGACGCCCGTCCACCGACCTCGGTGAGCTGGCGCTGGGTCAGACATGCGTGTAGCGTTCATGCGTGACGCTTTTTTTTTCATACCCCCCTGAGGTAAATCG

>gapA

CCAATGAGTCGTTGAGTGAAGACGGTCATCTGGTCGTTAACGGTAAAAAAATCCGTGTTACCGCTGAACGTGACCCGGCTAACCTGAAGTGGGACGAAGTTGGTGTTGACGTTGTTGCTGAAGCAACCGGTATCTTCCTGACCGACGAAACCGCTCGTAAACACATCACCGCTGGCGCGAAAAAAGTCGTTCTGACTGGCCCGTCCAAAGACAACACTCCGATGTTCGTTCGCGGCGCTAACTTCGACGCTTACGCTGGCCAGGACATCGTTTCCAACGCTTCCTGCACCACTAACTGCCTGGCGCCGCTGGCTAAAGTTATCAACGACAACTTCGGTATCGTTGAAGGCCTGATGACCACCGTCCACGCTACCACCGCTACTCAGAAAACCGTTGATGGCCCGTCTCACAAAGACTGGCGCGGCGGCCGCGGCGCAGCTCAGAACATCATCCCGTCCTCTACCGGCGCTGCTAAAGCAGTAGGTAAAGTACTGCCAGAACTGAACGGCAAACTGACCGGTATGGCGTTCCGCGTTCCAACTCCGAACGTATCTGTTGTTGACCTGACCGTTCGTCTGGAAAAAGCAGCGTCCTACGAAGAAATCAAGAAAGCCATCAAGGCTTTTTTTAAAAAAAA

>mdh

CGCATTTGACTCGCTCGGTTACGCCGGGCGTGGCGGTAGATCTAAGTCATATCCCCACAGATGTAAAAATTAAAGGATTTTCCGGTGAAGACGCTACTCCGGCGCTGGAAGGCGCGGATGTAGTGCTGATCTCCGCGGGCGTGGCGCGTAAGCCCGGCATGGATCGTTCCGACCTGTTTAATGTGAATGCGGGTATCGTGAAGAACCTCGTGCAGCAGATTGCCAAAACCTGCCCGCAGGCCTGCATCGGCATTATCACCAACCCGGTGAATACCACCGTGGCTATCGCCGCCGAAGTACTGAAAAAAGCCGGCGTGTACGATAAAAACAAACTGTTCGGCGTTACCACGCTGGACATCATCCGTTCCAATACCTTTGTGGCGGAGCTGAAAGGTAAATCGGCAACCGAGGTGGAAGTCCCGGTCATTGGTGGTCACTCCGGGGTCACCATTCTGCCTTTACTGTCGCAGATCCCCGGCGTCAGCTTTAGCGATCAGGAAATTGCCGACCTGACTAAACGTATTCAGAACGCCGGTACCGAAGTCGTGGAAGCGAAAGCGGGCGGCGGGTCGGCGACCTTGTCGATGGGCCAGGCGGCTGCCCGTTTTGGTCTCTCTCTGGTTCGCGCCATGCAGGGGGAAAAAGGCGTGGTGGAGTGCGCCTACGTGGAAGGCGACGGCCACTATGCGCGTTTCTTCTCCCAGCCGCTGTTGGGGAGAAAAAAAAGAGCGGGA

>pgi

AGTCTATTAGGGACGCTCTCTGGTCACACTTCTTCGGTGCGGAACCGAAGCGATTCTGCCGTACGACCAGTACATGCACCGCTTTGCCGCTTACTTCCAGCAGGGCAACATGGAGTCCAACGGTAAGTATGTTGACCGTAACGGCCACGCGGTAGACTACCAGACTGGCCCAATCATCTGGGGTGAGCCGGGCACCAACGGTCAGCACGCGTTCTACCAGCTGATCCACCAGGGCACCAAAATGGTACCGTGCGATTTCATCGCTCCGGCTATCACCCACAACCCGCTGTCTGACCACCATCAGAAACTGCTGTCTAACTTCTTCGCCCAGACCGAGGCCCTGGCCTTTGGTAAATCCCGCGAAGTGGTTGAGCAGGAATATCGCGATCAGGGTAAAGACCCGGCGACCCTGGAGCACGTGGTGCCGTTCAAAGTGTTCGAAGGTAACCGCCCGACTAACTCCATCCTGCTGCGTGAGATCACCCCGTTCAGCCTCGGGGCGCTGATTGCCCTGTACGAGCACAAAATCTTCACCCAGGGCGCGATCCTCAACATCTTCACCTTTGACCAGTGGGGCGTTGAGCTGGGCAAACAGCTGGCTAACCGCATCCTGCCGGAGCTGAAAGACGGCAGCGAAGTTAGCAGCCACGACAGCTCTACTAACGGCCTGATTAACCGCTTAGGGGGGGGGGGGGGGGGA

>phoE

AGCCGCTAGCATCTGAGTCTGGATCTGACCCTGCAGTACCAGGGTAAAAACGAAGGCCGTGAAGCGAAGAAACAGAACGGCGACGGCGTCGGCACCTCGTTAAGCTATGATTTCGGCGGCAGCGACTTCGCCGTCAGCGCAGCCTACACCAGCTCCGACCGTACCAACGATCAGAACCTGCTGGCCCGCGGCCAGGGTTCGAAAGCGGAAGCCTGGGCGACCGGCCTGAAATATGACGCCAACAATATCTACCTGGCGACCATGTACTCTGAAACCCGCAAGATGACCCCGATCAGCGGCGGCTTTGCCAACAAAGCGCAGAACTTTGAAGCGGTGGCGCAGTATCAGTTCGACTTCGGTCTGCGTCCGTCCCTCGGCTATGTGCTGTCGAAAGGGAAGGATATCGAAGGGGTGGGGAGTGAAGATCTGGTTAACTACATCGACGTGGGCCTGACCTACTACTTCAACAAAAACATGAACGCCTTCGTGGATTACAAAATCAACCAGCTGAAAAGCGATAACAAACTCGGCATCAACGATGACGACATCGTCGCGCTGGGTATCACCTACGTTTTTTAAAAAAAAA

>infB

TTAAGGATGCTCGGCGAGCGGGTGGTATTACCCAGCACATCGGTGCTTACCACGTCGAAACCGACAACGGCATGATCACCTTCCTGGATACCCCGGGCCACGCCGCGTTTACCTCCATGCGTGCTCGTGGCGCGCAGGCGACGGATATCGTGGTTCTGGTGGTGGCGGCAGACGACGGCGTGATGCCGCAGACTATCGAAGCTATCCAGCACGCTAAAGCGGCGCAGGTACCGGTGGTAGTGGCGGTGAACAAGATCGATAAGCCAGAAGCCGATCCGGATCGCGTGAAGAACGAACTGTCCCAGTACGGCATCCTGCCGGAAGAGTGGGGCGGCGAGAGCCAGTTTGTCCACGTTTCCGCGAAAGCGGGTACCGGCATCGACGACCTGCTGGACGCGATCCTGCTGCAGGCTGAAGTTCTAACGGAAAAAGCGCGA

>tonB

GATGAGCGTCTCGACGCAGCCGATAGAGATCACAATGGTGGCGCCGGCCGATCTTGAGCCGCCTCCGGCGGCGCAGCCTGTCGTGGAGCCCGTTGTTGAACCCGAACCTGAGCCGGAGCCAGAGGTAGCGCCTGAACCGCCGAAAGAGGCGCCGGTGGTGATCCATAAACCGGAACCTAAGCCGAAGCCCAAACCTAAACCCAAGCCTAAGCCGGAGAAAAAGGTTGAACAGCCGAAGCGGGAAGTGAAGCCGGCAGCAGAGCCGCGTCCGGCCTCGCCGTTTGAAAACAACAATACGGCGCCGGCGCGTACAGCGCCAAGTACCTCGACCGCAGCGGCTAAACCCACCGTTACTGCTCCGAGCGGCCCGCGGGCGATCAGCCGCGTTCAGCCGTCCTATCCGCCGCGCGCTCAGGCGCTGCGCATTGAAGGGACGGTACGGGTGAAGTTTGACGTTTCGCCTGATGGCCGCATTGATAATCTGCAGATCCTCTCTGTAGCCGGGAGGAAAAAA

FO22 (ST11)

>rpoB

TGGGTCCGTTGTAGAGCGTGCGGTGATGAGCGTCTGTCTCTTGGCGATCTGGATACCCTGATGCCTCAGGATATGATCAACGCCAAGCCGATTTCCGCAGCAGTGAAAGAGTTCTTTGGTTCCAGCCAGCTGTCTCAGTTTATGGACCAGAACAACCCGCTGTCTGAGATTACGCACAAACGTCGTATCTCCGCACTCGGCCCAGGCGGTCTGACCCGTGAGCGCGCAGGCTTCGAAGTTCGAGACGTACACCCGACCCACTACGGTCGCGTATGTCCGATCGAAACGCCTGAAGGTCCGAACATCGGTCTGATTAACTCCCTGTCCGTGTACGCGCAGACCAACGAATATGGCTTCCTTGAGACGCCGTATCGTAAAGTGACCAACGGTGTGGTTACTGACGAAATTCACTACCTGTCTGCTATCGAAGAAGGCAACTACGTTATCGCTCAGGCGAACTCCAACCTGGATGAAAACGGCCACTTCGTAGAAGATCTGGTTACCTGCCGTAGCAAAGGCGAATCCAGCTTGTTCAGCCGCGACCAGGTTGACTACATGGACGTATCCACCCAGCAGGTGGTATCCGTCGGTGCGTCCCTGATCCCGTTCCTGGAACACGATGACGCCAACCGTGCATTGATGGGTGCGAACATGCAACGTCAGGCGGTTCCGACTCTGCGCGCTGATAAGCCGCTGGTTGGTACCGGTATGGAACGTGCTGTTGCCGTTGACTCCGGTGTTACTGCCGTGGCTAAACGTGGCGGTACCGTTCAGTACGTGGATGCTTCCCGTATCGTTATCAAAGTTAACGAAGACGAGATGTACCCGGGCGAAGCAGGTATCGACATCTATAACCTGACCAAGTACACCCGTTCTAACCAGAACACCTGCATCAACCAGATGCCTTGCGTGTCCCTGGGCGAACCTATTGAGCGCGGCGACGTGCTGGCAGACGGCCCGTCCACCGACCTCGGTGAGCTGGCGCTGGGTCAGAACATGCGTGTAGCGTTCATGCCGTGGAACGGGTC

>gapA

GGATCAGTCGTTGAGTGAGACGGTCATCTGGTCGTTAACGGTAAAAAAATCCGTGTTACCGCTGAACGTGACCCGGCTAACCTGAAGTGGGACGAAGTTGGTGTTGACGTTGTTGCTGAAGCAACCGGTATCTTCCTGACCGACGAAACCGCTCGTAAACACATCACCGCTGGCGCGAAAAAAGTCGTTCTGACTGGCCCGTCCAAAGACAACACTCCGATGTTCGTTCGCGGCGCTAACTTCGACGCTTACGCTGGCCAGGACATCGTTTCCAACGCTTCCTGCACCACTAACTGCCTGGCGCCGCTGGCTAAAGTTATCAACGACAACTTCGGTATCGTTGAAGGCCTGATGACCACCGTCCACGCTACCACCGCTACTCAGAAAACCGTTGATGGCCCGTCTCACAAAGACTGGCGCGGCGGCCGCGGCGCAGCTCAGAACATCATCCCGTCCTCTACCGGCGCTGCTAAAGCAGTAGGTAAAGTACTGCCAGAACTGAACGGCAAACTGACCGGTATGGCGTTCCGCGTTCCAACTCCGAACGTATCTGTTGTTGACCTGACCGTTCGTCTGGAAAAAGCAGCGTCCTACGAAGAAATCAAGAAAGCCATCAAAGCGTCTTTTCTCTGAGAGA

>mdh

CGAAATCAATCGCTCGGTTACGCCGGGCGTGGCGGTAGATCTAAGTCATATCCCCACAGATGTAAAAATTAAAGGATTTTCCGGTGAAGACGCTACTCCGGCGCTGGAAGGCGCGGATGTAGTGCTGATCTCCGCGGGCGTGGCGCGTAAGCCCGGCATGGATCGTTCCGACCTGTTTAATGTGAATGCGGGTATCGTGAAGAACCTCGTGCAGCAGATTGCCAAAACCTGCCCGCAGGCCTGCATCGGCATTATCACCAACCCGGTGAATACCACCGTGGCTATCGCCGCCGAAGTACTGAAAAAAGCCGGCGTGTACGATAAAAACAAACTGTTCGGCGTTACCACGCTGGACATCATCCGTTCCAATACCTTTGTGGCGGAGCTGAAAGGTAAATCGGCAACCGAGGTGGAAGTCCCGGTCATTGGTGGTCACTCCGGGGTCACCATTCTGCCTTTACTGTCGCAGATCCCCGGCGTCAGCTTTAGCGATCAGGAAATTGCCGACCTGACTAAACGTATTCAGAACGCCGGTACCGAAGTCGTGGAAGCGAAAGCGGGCGGCGGGTCGGCGACCTTGTCGATGGGCCAGGCGGCTGCCCGTTTTGGTCTCTCTCTGGTTCGCGCCATGCAGGGGGAAAAAGGCGTGGTGGAGTGCGCCTACGTGGAAGGCGACGGCCACTATGCGCGTTTCTTCTCCCAGCCGCTGCTGCTGGGAAAAAAACGGAA

>pgi

TCTTGCGTAACAACTTCTTCGGTGCGGAACCGAAGCGATTCTGCCGTACGACCAGTACATGCACCGCTTTGCCGCTTACTTCCAGCAGGGCAACATGGAGTCCAACGGTAAGTATGTTGACCGTAACGGCCACGCGGTAGACTACCAGACTGGCCCAATCATCTGGGGTGAGCCGGGCACCAACGGTCAGCACGCGTTCTACCAGCTGATCCACCAGGGCACCAAAATGGTACCGTGCGATTTCATCGCTCCGGCTATCACCCACAACCCGCTGTCTGACCACCATCAGAAACTGCTGTCTAACTTCTTCGCCCAGACCGAGGCCCTGGCCTTTGGTAAATCCCGCGAAGTGGTTGAGCAGGAATATCGCGATCAGGGTAAAGACCCGGCGACCCTGGAGCACGTGGTGCCGTTCAAAGTGTTCGAAGGTAACCGCCCGACTAACTCCATCCTGCTGCGTGAGATCACCCCGTTCAGCCTCGGGGCGCTGATTGCCCTGTACGAGCACAAAATCTTCACCCAGGGCGCGATCCTCAACATCTTCACCTTTGACCAGTGGGGCGTTGAGCTGGGCAAACAGCTGGCTAACCGCATCCTGCCGGAGCTGAAAGACGGCAGCGAAGTTAGCAGCCACGACAGCTCTACTAACGGCCTGATTAACCGCTATAAGGGGGGGGGCGGGAA

>phoE

TGGAGTTACAGTAGCTGCGATGCTGATCTCTGACTGCAGTACCAGGGTAAAACGAAGGCCGTGAAGCGAAGAAACAGAACGGCGACGGCGTCGGCACCTCGTTAAGCTATGATTTCGGCGGCAGCGACTTCGCCGTCAGCGCAGCCTACACCAGCTCCGACCGTACCAACGATCAGAACCTGCTGGCCCGCGGCCAGGGTTCGAAAGCGGAAGCCTGGGCGACCGGCCTGAAATATGACGCCAACAATATCTACCTGGCGACCATGTACTCTGAAACCCGCAAGATGACCCCGATCAGCGGCGGCTTTGCCAACAAAGCGCAGAACTTTGAAGCGGTGGCGCAGTATCAGTTCGACTTCGGTCTGCGTCCGTCCCTCGGCTATGTGCTGTCGAAAGGGAAGGATATCGAAGGGGTGGGGAGTGAAGATCTGGTTAACTACATCGACGTGGGCCTGACCTACTACTTCAACAAAAACATGAACGCCTTCGTGGATTACAAAATCAACCAGCTGAAAAGCGATAACAAACTCGGCATCAACGATGACGACATCGTCGCGCTGGGTATCACCTACCATTTTCTGATCAA

>infB

ATAGTGCTCGGCGAGCGGGTGGTATTACCCAGCACATCGGTGCTTACCACGTCGAAACCGACAACGGCATGATCACCTTCCTGGATACCCCGGGCCACGCCGCGTTTACCTCCATGCGTGCTCGTGGCGCGCAGGCGACGGATATCGTGGTTCTGGTGGTGGCGGCAGACGACGGCGTGATGCCGCAGACTATCGAAGCTATCCAGCACGCTAAAGCGGCGCAGGTACCGGTGGTAGTGGCGGTGAACAAGATCGATAAGCCAGAAGCCGATCCGGATCGCGTGAAGAACGAACTGTCCCAGTACGGCATCCTGCCGGAAGAGTGGGGCGGCGAGAGCCAGTTTGTCCACGTTTCCGCGAAAGCGGGTACCGGCATCGACGACCTGCTGGACGCGATCCTGCTGCAGGCTGAAGTTCTTGCCTGTGAAAGCGA

>tonB

GGGTACCCCGTCTCGACGCAGCCGATAGAGATCACAATGGTGGCGCCGGCCGATCTTGAGCCGCCTCCGGCGGCGCAGCCTGTCGTGGAGCCCGTTGTTGAACCCGAACCTGAGCCGGAGCCAGAGGTAGCGCCTGAACCGCCGAAAGAGGCGCCGGTGGTGATCCATAAACCGGAACCTAAGCCGAAGCCCAAACCTAAACCCAAGCCTAAGCCGGAGAAAAAGGTTGAACAGCCGAAGCGGGAAGTGAAGCCGGCAGCAGAGCCGCGTCCGGCCTCGCCGTTTGAAAACAACAATACGGCGCCGGCGCGTACAGCGCCAAGTACCTCGACCGCAGCGGCTAAACCCACCGTTACTGCTCCGAGCGGCCCGCGGGCGATCAGCCGCGTTCAGCCGTCCTATCCGCCGCGCGCTCAGGCGCTGCGCATTGAAGGGACGGTACGGGTGAAGTTTGACGTTTCGCCTGATGGCCGCATTGATAATCTGCAGATCCTCTCTGCTCACCCCGGGCGAATAA

FO23 (ST11)

>rpoB

CAACATTGGCTGCTCGTGTAGAGCGTGCGGTGAaGAGCGTCTGTCTCTTGGCGATCTGGATACCCTGATGCCTCAGGATATGATCAACGCCAAGCCGATTTCCGCAGCAGTGAAAGAGTTCTTTGGTTCCAGTCAGCTGTCTCAGTTTATGGACCAGAACAACCCGCTGTCTGAGATTACGCACAAACGTCGTATCTCCGCACTCGGCCCAGGCGGTCTGACCCGTGAGCGCGCAGGCTTCGAAGTTCGAGACGTACACCCGACCCACTACGGTCGCGTATGTCCGATCGAAACGCCTGAAGGTCCGAACATCGGTCTGATTAACTCCCTGTCCGTGTACGCGCAGACCAACGAATATGGCTTCCTTGAGACGCCGTATCGTAAAGTGACCAACGGTGTGGTTACTGACGAAATTCACTACCTGTCTGCTATCGAAGAAGGCAACTACGTTATCGCTCAGGCGAACTCCAACCTGGATGAAAACGGCCACTTCGTAGAAGATCTGGTTACCTGCCGTAGCAAAGGCGAATCCAGCTTGTTCAGCCGCGACCAGGTTGACTACATGGACGTATCCACCCAGCAGGTGGTATCCGTCGGTGCGTCCCTGATCCCGTTCCTGGAACACGATGACGCCAACCGTGCATTGATGGGTGCGAACATGCAACGTCAGGCGGTTCCGACTCTGCGCGCTGATAAGCCGCTGGTTGGTACCGGTATGGAACGTGCTGTTGCCGTTGACTCCGGTGTTACTGCCGTGGCTAAACGTGGCGGTACCGTTCAGTACGTGGATGCTTCCCGTATCGTTATCAAAGTTAACGAAGACGAGATGTACCCGGGCGAAGCAGGTATCGACATCTATAACCTGACCAAGTACACCCGTTCTAACCAGAACACCTGCATCAACCAGATGCCTTGCGTGTCCCTGGGCGAACCTATTGAGCGCGGCGACGTGCTGGCAGACGGCCCGTCCACCGACCTCGGTGAGCTGGCGCTGGGTCAGAACATGCGTGTAGCGTTCATGCCGTGGAACGGGTTACATTTCTCTCAAAAGATCTCAC

>gapA

GCGATTCAGATCGTTGAGTGAGACGGTCATCTGGTCGTTAACGGTAAAAAAATCCGTGTTACCGCTGAACGTGACCCGGCTAACCTGAAGTGGGACGAAGTTGGTGTTGACGTTGTTGCTGAAGCAACCGGTATCTTCCTGACCGACAAAACCGCTCGTAAACACATCACCGCTGGCGCGAAAAAAGTCGTTCTGACTGGCCCGTCCAAAGACAACACTCCGATGTTCGTTCGCGGCGCTAACTTCGACGCTTACGCAGGCCAGGACATCGTTTCCAACGCTTCCTGCACCACCAACTGCCTGGCGCCGCTGGCTAAAGTTATCAACGACAACTTCGGTATCGTTGAAGGCCTGATGACCACCGTCCACGCTACCACCGCTACTCAGAAAACCGTTGATGGCCCGTCTCACAAAGACTGGCGCGGCGGCCGCGGCGCAGCTCAGAACATCATCCCGTCCTCTACCGGCGCTGCTAAAGCAGTAGGTAAAGTACTGCCAGAACTGAACGGCAAACTGACCGGTATGGCGTTCCGCGTTCCGACTCCGAACGTATCTGTTGTTGACCTGACCGTTCGTCTGGAAAAAGCAGCGTCCTACGAAGAAATCAAGAAAGCCATCAAAGGCTTTTTTTAAAAAAA

>mdh

GGGTAGCGATCGCTCGGTTAACCGGGCGTGGCGGTAGATCTAAGTCATATCCCCACAGATGTAAAAATTAAAGGATTTTCCGGTGAAGACGCTACTCCGGCGCTGGAAGGCGCGGATGTAGTGCTGATCTCCGCGGGCGTGGCGCGTAAGCCCGGCATGGATCGTTCCGACCTGTTTAATGTGAATGCGGGTATCGTGAAGAACCTCGTGCAGCAGATTGCCAAAACCTGCCCGCAGGCCTGCATCGGCATTATCACCAACCCGGTGAATACCACCGTGGCTATCGCCGCCGAAGTACTGAAAAAAGCCGGCGTGTACGATAAAAACAAACTGTTCGGCGTTACCACGCTGGACATCATCCGTTCCAATACCTTTGTGGCGGAGCTGAAAGGTAAATCGGCAACCGAGGTGGAAGTCCCGGTCATTGGTGGTCACTCCGGGGTCACCATTCTGCCTTTACTGTCGCAGATCCCCGGCGTCAGCTTTAGCGATCAGGAAATTGCCGACCTGACTAAACGTATTCAGAACGCCGGTACCGAAGTCGTGGAAGCGAAAGCGGGCGGCGGGTCGGCGACCTTGTCGATGGGCCAGGCGGCTGCCCGTTTTGGTCTCTCTCTGGTTCGCGCCATGCAGGGGGAAAAAGGCGTGGTGGAGTGCGCCTACGTGGAAGGCGACGGCCACTATGCGCGTTTCTTCTCCCAGCCGCTGCTGTGGAGAAAAAAAAAACGGAA

>pgi

TGTGGAAGGCTCTGGTAACACTTCTTCGGTGCGGAACCGAAGCGATTCTGCCGTACGACCAGTACATGCACCGCTTTGCCGCTTACTTCCAGCAGGGCAACATGGAGTCCAACGGTAAGTATGTTGACCGTAACGGCCACGCGGTAGACTACCAGACTGGCCCAATCATCTGGGGTGAGCCGGGCACCAACGGTCAGCACGCGTTCTACCAGCTGATCCACCAGGGCACCAAAATGGTACCGTGCGATTTCATCGCTCCGGCTATCACCCACAACCCGCTGTCTGACCACCATCAGAAACTGCTGTCTAACTTCTTCGCCCAGACCGAGGCCCTGGCCTTTGGTAAATCCCGCGAAGTGGTTGAGCAGGAATATCGCGATCAGGGTAAAGACCCGGCGACCCTGGAGCACGTGGTGCCGTTCAAAGTGTTCGAAGGTAACCGCCCGACTAACTCCATCCTGCTGCGTGAGATCACCCCGTTCAGCCTCGGGGCGCTGATTGCCCTGTACGAGCACAAAATCTTCACCCAGGGCGCGATCCTCAACATCTTCACCTTTGACCAGTGGGGCGTTGAGCTGGGCAAACAGCTGGCTAACCGCATCCTGCCGGAGCTGAAAGACGGCAGCGAAGTTAGCAGCCACGACAGCTCTACTAACGGCCTGATTAACCGCTATAACGGGGGGGGGCCGCAAA

>phoE

ATAAGAATACCGAGTCTCATCTGACCTGCAGTACCAGGGTAAAAACGAAGGCCGTGAAGCGAAGAAACAGAACGGCGACGGCGTCGGCACCTCGTTAAGCTATGATTTCGGCGGCAGCGACTTCGCCGTCAGCGCAGCCTACACCAGCTCCGACCGTACCAACGATCAGAACCTGCTGGCCCGCGGCCAGGGTTCGAAAGCGGAAGCCTGGGCGACCGGCCTGAAATATGACGCCAACAATATCTACCTGGCGACCATGTACTCTGAAACCCGCAAGATGACCCCGATCAGCGGCGGCTTTGCCAACAAAGCGCAGAACTTTGAAGCGGTGGCGCAGTATCAGTTCGACTTCGGTCTGCGTCCGTCCCTCGGCTATGTGCTGTCGAAAGGGAAGGATATCGAAGGGGTGGGGAGTGAAGATCTGGTTAACTACATCGACGTGGGCCTGACCTACTACTTCAACAAAAACATGAACGCCTTCGTGGATTACAAAATCAACCAGCTGAAAAGCGATAACAAACTCGGCATCAACGATGACGACATCGTCGCGCTGGGTATCACCTACTTTTTTTTTGAACCAA

>infB

ATAAGTTGCTCGGCGAGCGGGTGGTATTACCCAGCACATCGGTGCTTACCACGTCGAAACCGACAACGGCATGATCACCTTCCTGGATACCCCGGGCCACGCCGCGTTTACCTCCATGCGTGCTCGTGGCGCGCAGGCGACGGATATCGTGGTTCTGGTGGTGGCGGCAGACGACGGCGTGATGCCGCAGACTATCGAAGCTATCCAGCACGCTAAAGCGGCGCAGGTACCGGTGGTAGTGGCGGTGAACAAGATCGATAAGCCAGAAGCCGATCCGGATCGCGTGAAGAACGAACTGTCCCAGTACGGCATCCTGCCGGAAGAGTGGGGCGGCGAGAGCCAGTTCGTCCACGTTTCCGCGAAAGCGGGTACCGGCATCGACGACCTGCTGGACGCGATCCTGCTGCAGGCTGAAGTTCTTGGGTGTGAAAGCGAA

>tonB

GGACTCGTCTCACTCGACGCAGCCGATAGAGATCACAATGGTGGCGCCGGCCGATCTTGAGCCGCCTCCGGCGGCGCAGCCTGTCGTGGAGCCCGTTGTTGAACCCGAACCTGAGCCGGAGCCAGAGGTAGTGCCTGAACCGCCGAAAGAGGCGCCGGTGGTGATCCATAAACCGGAACCTAAGCCGAAGCCCAAACCTAAACCCAAGCCTAAGCCGGAGAAAAAGGTTGAACAGCCGAAGCGGGAAGTGAAGCCGGCAGCAGAGCCGCGTCCGGCCTCGCCGTTTGAAAACAACAATACGGCGCCGGCGCGTACAGCGCCAAGCACCTCGACAGCAGCGGCTAAACCCACCGTTACTGCTCCAAGCGGCCCGCGGGCGATCAGCCGCGTTCAGCCGTCCTATCCAGCGCGCGCTCAGGCGCTGCGCATTGAAGGTACGGTACGGGTGAAGTTTGACGTTTCGCCTGATGGCCGTATTGATAATCTGCAGATCCTCTCTGCTCCCCCGGGCAATTAAA

FO24 (ST11)

>rpoB

GTTCGGTGTTAGAGCGTGCGGTGAAGAGCGTCTGTCTCTTGGCGATCTGGATACCCTGATGCCTCAGGATATGATCAACGCCAAGCCGATTTCCGCAGCAGTGAAAGAGTTCTTTGGTTCCAGCCAGCTGTCTCAGTTTATGGACCAGAACAACCCGCTGTCTGAGATTACGCACAAACGTCGTATCTCCGCACTCGGCCCAGGCGGTCTGACCCGTGAGCGCGCAGGCTTCGAAGTTCGAGACGTACACCCGACCCACTACGGTCGCGTATGTCCGATCGAAACGCCTGAAGGTCCGAACATCGGTCTGATTAACTCCCTGTCCGTGTACGCGCAGACCAACGAATATGGCTTCCTTGAGACGCCGTATCGTAAAGTGACCAACGGTGTGGTTACTGACGAAATTCACTACCTGTCTGCTATCGAAGAAGGCAACTACGTTATCGCTCAGGCGAACTCCAACCTGGATGAAAACGGCCACTTCGTAGAAGATCTGGTTACCTGCCGTAGCAAAGGCGAATCCAGCTTGTTCAGCCGCGACCAGGTTGACTACATGGACGTATCCACCCAGCAGGTGGTATCCGTCGGTGCGTCCCTGATCCCGTTCCTGGAACACGATGACGCCAACCGTGCATTGATGGGTGCGAACATGCAACGTCAGGCGGTTCCGACTCTGCGCGCTGATAAGCCGCTGGTTGGTACCGGTATGGAACGTGCTGTTGCCGTTGACTCCGGTGTTACTGCCGTGGCTAAACGTGGCGGTACCGTTCAGTACGTGGATGCTTCCCGTATCGTTATCAAAGTTAACGAAGACGAGATGTACCCGGGCGAAGCAGGTATCGACATCTATAACCTGACCAAGTACACCCGTTCTAACCAGAACACCTGCATCAACCAGATGCCTTGCGTGTCCCTGGGCGAACCTATTGAGCGCGGCGACGTGCTGGCAGACGGCCCGTCCACCGACCTCGGTGAGCTGGCGCTGGGTCAGAACATGCGTGTAGCGTTCATGCCGTGGAACGGGTCTTCT

>gapA

GGGTTTCAGATACGTTGAGTGAGACGGTCATCTGGTCGTTAACGGTAAAAAAATCCGTGTTACCGCTGAACGTGACCCGGCTAACCTGAAGTGGGACGAAGTTGGTGTTGACGTTGTTGCTGAAGCAACCGGTATCTTCCTGACCGACGAAACCGCTCGTAAACACATCACCGCTGGCGCGAAAAAAGTCGTTCTGACTGGCCCGTCCAAAGACAACACTCCGATGTTCGTTCGCGGCGCTAACTTCGACGCTTACGCTGGCCAGGACATCGTTTCCAACGCTTCCTGCACCACTAACTGCCTGGCGCCGCTGGCTAAAGTTATCAACGACAACTTCGGTATCGTTGAAGGCCTGATGACCACCGTCCACGCTACCACCGCTACTCAGAAAACCGTTGATGGCCCGTCTCACAAAGACTGGCGCGGCGGCCGCGGCGCAGCTCAGAACATCATCCCGTCCTCTACCGGCGCTGCTAAAGCAGTAGGTAAAGTACTGCCAGAACTGAACGGCAAACTGACCGGTATGGCGTTCCGCGTTCCAACTCCGAACGTATCTGTTGTTGACCTGACCGTTCGTCTGGAAAAAGCAGCGTCCTACGAAGAAATCAAGAAAGCCATCAAAGCCCTTTTCTGAAGA

>mdh

CCGGAGTGATCGCTCGGTTACGCCGGGCGTGGCGGTAGATCTAAGTCATATCCCCACAGATGTAAAAATTAAAGGATTTTCCGGTGAAGACGCTACTCCGGCGCTGGAAGGCGCGGATGTAGTGCTGATCTCCGCGGGCGTGGCGCGTAAGCCCGGCATGGATCGTTCCGACCTGTTTAATGTGAATGCGGGTATCGTGAAGAACCTCGTGCAGCAGATTGCCAAAACCTGCCCGCAGGCCTGCATCGGCATTATCACCAACCCGGTGAATACCACCGTGGCTATCGCCGCCGAAGTACTGAAAAAAGCCGGCGTGTACGATAAAAACAAACTGTTCGGCGTTACCACGCTGGACATCATCCGTTCCAATACCTTTGTGGCGGAGCTGAAAGGTAAATCGGCAACCGAGGTGGAAGTCCCGGTCATTGGTGGTCACTCCGGGGTCACCATTCTGCCTTTACTGTCGCAGATCCCCGGCGTCAGCTTTAGCGATCAGGAAATTGCCGACCTGACTAAACGTATTCAGAACGCCGGTACCGAAGTCGTGGAAGCGAAAGCGGGCGGCGGGTCGGCGACCTTGTCGATGGGCCAGGCGGCTGCCCGTTTTGGTCTCTCTCTGGTTCGCGCCATGCAGGGGGAAAAAGGCGTGGTGGAGTGCGCCTACGTGGAAGGCGACGGCCACTATGCGCGTTTCTTCTCCCAGCCGCTGCTGCTGGAAAAAAAACGGA

>pgi

TGATTAACAACTTCTTCGGTGCGGAACCGAAGCGATTCTGCCGTACGACCAGTACATGCACCGCTTTGCCGCTTACTTCCAGCAGGGCAACATGGAGTCCAACGGTAAGTATGTTGACCGTAACGGCCACGCGGTAGACTACCAGACTGGCCCAATCATCTGGGGTGAGCCGGGCACCAACGGTCAGCACGCGTTCTACCAGCTGATCCACCAGGGCACCAAAATGGTACCGTGCGATTTCATCGCTCCGGCTATCACCCACAACCCGCTGTCTGACCACCATCAGAAACTGCTGTCTAACTTCTTCGCCCAGACCGAGGCCCTGGCCTTTGGTAAATCCCGCGAAGTGGTTGAGCAGGAATATCGCGATCAGGGTAAAGACCCGGCGACCCTGGAGCACGTGGTGCCGTTCAAAGTGTTCGAAGGTAACCGCCCGACTAACTCCATCCTGCTGCGTGAGATCACCCCGTTCAGCCTCGGGGCGCTGATTGCCCTGTACGAGCACAAAATCTTCACCCAGGGCGCGATCCTCAACATCTTCACCTTTGACCAGTGGGGCGTTGAGCTGGGCAAACAGCTGGCTAACCGCATCCTGCCGGAGCTGAAAGACGGCAGCGAAGTTAGCAGCCACGACAGCTCTACTAACGGCCTGATTAACCGCTATAAACGGGGGGGGGCCAAA

>phoE

GGTAAGGTTACTGCATCTGACCCTGCAGTACCAGGGTAAAAACGAAGGCCGTGAAGCGAAGAAACAGAACGGCGACGGCGTCGGCACCTCGTTAAGCTATGATTTCGGCGGCAGCGACTTCGCCGTCAGCGCAGCCTACACCAGCTCCGACCGTACCAACGATCAGAACCTGCTGGCCCGCGGCCAGGGTTCGAAAGCGGAAGCCTGGGCGACCGGCCTGAAATATGACGCCAACAATATCTACCTGGCGACCATGTACTCTGAAACCCGCAAGATGACCCCGATCAGCGGCGGCTTTGCCAACAAAGCGCAGAACTTTGAAGCGGTGGCGCAGTATCAGTTCGACTTCGGTCTGCGTCCGTCCCTCGGCTATGTGCTGTCGAAAGGGAAGGATATCGAAGGGGTGGGGAGTGAAGATCTGGTTAACTACATCGACGTGGGCCTGACCTACTACTTCAACAAAAACATGAACGCCTTCGTGGATTACAAAATCAACCAGCTGAAAAGCGATAACAAACTCGGCATCAACGATGACGACATCGTCGCGCTGGGTATCACCTACCATTTTCCTGATCAA

>infB

GCGGTGCTCGGCGAGCGGGTGGTATTACCCAGCACATCGGTGCTTACCACGTCGAAACCGACAACGGCATGATCACCTTCCTGGATACCCCGGGCCACGCCGCGTTTACCTCCATGCGTGCTCGTGGCGCGCAGGCGACGGATATCGTGGTTCTGGTGGTGGCGGCAGACGACGGCGTGATGCCGCAGACTATCGAAGCTATCCAGCACGCTAAAGCGGCGCAGGTACCGGTGGTAGTGGCGGTGAACAAGATCGATAAGCCAGAAGCCGATCCGGATCGCGTGAAGAACGAACTGTCCCAGTACGGCATCCTGCCGGAAGAGTGGGGCGGCGAGAGCCAGTTTGTCCACGTTTCCGCGAAAGCGGGTACCGGCATCGACGACCTGCTGGACGCGATCCTGCTGCAGGCTGAAGTTCTTGACCCTGAAAGCGA

>tonB

GCGTACCTCTCGACGCAGCCGATAGAGATCACAATGGTGGCGCCGGCCGATCTTGAGCCGCCTCCGGCGGCGCAGCCTGTCGTGGAGCCCGTTGTTGAACCCGAACCTGAGCCGGAGCCAGAGGTAGCGCCTGAACCGCCGAAAGAGGCGCCGGTGGTGATCCATAAACCGGAACCTAAGCCGAAGCCCAAACCTAAACCCAAGCCTAAGCCGGAGAAAAAGGTTGAACAGCCGAAGCGGGAAGTGAAGCCGGCAGCAGAGCCGCGTCCGGCCTCGCCGTTTGAAAACAACAATACGGCGCCGGCGCGTACAGCGCCAAGTACCTCGACCGCAGCGGCTAAACCCACCGTTACTGCTCCGAGCGGCCCGCGGGCGATCAGCCGCGTTCAGCCGTCCTATCCGCCGCGCGCTCAGGCGCTGCGCATTGAAGGGACGGTACGGGTGAAGTTTGACGTTTCGCCTGATGGCCGCATTGATAATCTGCAGATCCTCTCTGCTCGCCGGGGGCGAATAA

FO25 (ST11)

>rpoB

CGCGGTAGGTTGTAGGAGCGTGCGGTGAGAGCGTCTGTCTCTTGGCGATCTGGATACCCTGATGCCTCAGGATATGATCAACGCCAAGCCGATTTCCGCAGCAGTGAAAGAGTTCTTTGGTTCCAGCCAGCTGTCTCAGTTTATGGACCAGAACAACCCGCTGTCTGAGATTACGCACAAACGTCGTATCTCCGCACTCGGCCCAGGCGGTCTGACCCGTGAGCGCGCAGGCTTCGAAGTTCGAGACGTACACCCGACCCACTACGGTCGCGTATGTCCGATCGAAACGCCTGAAGGTCCGAACATCGGTCTGATTAACTCCCTGTCCGTGTACGCGCAGACCAACGAATATGGCTTCCTTGAGACGCCGTATCGTAAAGTGACCAACGGTGTGGTTACTGACGAAATTCACTACCTGTCTGCTATCGAAGAAGGCAACTACGTTATCGCTCAGGCGAACTCCAACCTGGATGAAAACGGCCACTTCGTAGAAGATCTGGTTACCTGCCGTAGCAAAGGCGAATCCAGCTTGTTCAGCCGCGACCAGGTTGACTACATGGACGTATCCACCCAGCAGGTGGTATCCGTCGGTGCGTCCCTGATCCCGTTCCTGGAACACGATGACGCCAACCGTGCATTGATGGGTGCGAACATGCAACGTCAGGCGGTTCCGACTCTGCGCGCTGATAAGCCGCTGGTTGGTACCGGTATGGAACGTGCTGTTGCCGTTGACTCCGGTGTTACTGCCGTGGCTAAACGTGGCGGTACCGTTCAGTACGTGGATGCTTCCCGTATCGTTATCAAAGTTAACGAAGACGAGATGTACCCGGGCGAAGCAGGTATCGACATCTATAACCTGACCAAGTACACCCGTTCTAACCAGAACACCTGCATCAACCAGATGCCTTGCGTGTCCCTGGGCGAACCTATTGAGCGCGGCGACGTGCTGGCAGACGGCCCGTCCACCGACCTCGGTGAGCTGGCGCTGGGTCAGAACATGCGTGTAGCGTTCATGCCGTGA

>gapA

CGGTTTAAGATCGTTGAGTGAAGACGGTCATCTGGTCGTTAACGGTAAAAAAATCCGTGTTACCGCTGAACGTGACCCGGCTAACCTGAAGTGGGACGAAGTTGGTGTTGACGTTGTTGCTGAAGCAACCGGTATCTTCCTGACCGACGAAACCGCTCGTAAACACATCACCGCTGGCGCGAAAAAAGTCGTTCTGACTGGCCCGTCCAAAGACAACACTCCGATGTTCGTTCGCGGCGCTAACTTCGACGCTTACGCTGGCCAGGACATCGTTTCCAACGCTTCCTGCACCACTAACTGCCTGGCGCCGCTGGCTAAAGTTATCAACGACAACTTCGGTATCGTTGAAGGCCTGATGACCACCGTCCACGCTACCACCGCTACTCAGAAAACCGTTGATGGCCCGTCTCACAAAGACTGGCGCGGCGGCCGCGGCGCAGCTCAGAACATCATCCCGTCCTCTACCGGCGCTGCTAAAGCAGTAGGTAAAGTACTGCCAGAACTGAACGGCAAACTGACCGGTATGGCGTTCCGCGTTCCAACTCCGAACGTATCTGTTGTTGACCTGACCGTTCGTCTGGAAAAAGCAGCGTCCTACGAAGAAATCAAGAAAGCCATCAAAGCCCTTTTCTGAAG

>mdh

GCGAAGTCGACTCGCTCGGTTACGCCGGGCGTGGCGGTAGATCTAAGTCATATCCCCACAGATGTAAAAATTAAAGGATTTTCCGGTGAAGACGCTACTCCGGCGCTGGAAGGCGCGGATGTAGTGCTGATCTCCGCGGGCGTGGCGCGTAAGCCCGGCATGGATCGTTCCGACCTGTTTAATGTGAATGCGGGTATCGTGAAGAACCTCGTGCAGCAGATTGCCAAAACCTGCCCGCAGGCCTGCATCGGCATTATCACCAACCCGGTGAATACCACCGTGGCTATCGCCGCCGAAGTACTGAAAAAAGCCGGCGTGTACGATAAAAACAAACTGTTCGGCGTTACCACGCTGGACATCATCCGTTCCAATACCTTTGTGGCGGAGCTGAAAGGTAAATCGGCAACCGAGGTGGAAGTCCCGGTCATTGGTGGTCACTCCGGGGTCACCATTCTGCCTTTACTGTCGCAGATCCCCGGCGTCAGCTTTAGCGATCAGGAAATTGCCGACCTGACTAAACGTATTCAGAACGCCGGTACCGAAGTCGTGGAAGCGAAAGCGGGCGGCGGGTCGGCGACCTTGTCGATGGGCCAGGCGGCTGCCCGTTTTGGTCTCTCTCTGGTTCGCGCCATGCAGGGGGAAAAAGGCGTGGTGGAGTGCGCCTACGTGGAAGGCGACGGCCACTATGCGCGTTTCTTCTCCCAGCCGCTGCTGCTGGAAAAAAAAACGGA

>pgi

GACACCAGCTTCTTCGGTGCGGAACCGAAGCGATTCTGCCGTACGACCAGTACATGCACCGCTTTGCCGCTTACTTCCAGCAGGGCAACATGGAGTCCAACGGTAAGTATGTTGACCGTAACGGCCACGCGGTAGACTACCAGACTGGCCCAATCATCTGGGGTGAGCCGGGCACCAACGGTCAGCACGCGTTCTACCAGCTGATCCACCAGGGCACCAAAATGGTACCGTGCGATTTCATCGCTCCGGCTATCACCCACAACCCGCTGTCTGACCACCATCAGAAACTGCTGTCTAACTTCTTCGCCCAGACCGAGGCCCTGGCCTTTGGTAAATCCCGCGAAGTGGTTGAGCAGGAATATCGCGATCAGGGTAAAGACCCGGCGACCCTGGAGCACGTGGTGCCGTTCAAAGTGTTCGAAGGTAACCGCCCGACTAACTCCATCCTGCTGCGTGAGATCACCCCGTTCAGCCTCGGGGCGCTGATTGCCCTGTACGAGCACAAAATCTTCACCCAGGGCGCGATCCTCAACATCTTCACCTTTGACCAGTGGGGCGTTGAGCTGGGCAAACAGCTGGCTAACCGCATCCTGCCGGAGCTGAAAGACGGCAGCGAAGTTAGCAGCCACGACAGCTCTACTAACGGCCTGATTAACCGCTATAACGGGGGGGGCGGGGA

>phoE

CCTGAACATCTGACCCTGCAGTACCAGGGTAAAACGAAGGCCGTGAAGCGAAGAAACAGAACGGCGACGGCGTCGGCACCTCGTTAAGCTATGATTTCGGCGGCAGCGACTTCGCCGTCAGCGCAGCCTACACCAGCTCCGACCGTACCAACGATCAGAACCTGCTGGCCCGCGGCCAGGGTTCGAAAGCGGAAGCCTGGGCGACCGGCCTGAAATATGACGCCAACAATATCTACCTGGCGACCATGTACTCTGAAACCCGCAAGATGACCCCGATCAGCGGCGGCTTTGCCAACAAAGCGCAGAACTTTGAAGCGGTGGCGCAGTATCAGTTCGACTTCGGTCTGCGTCCGTCCCTCGGCTATGTGCTGTCGAAAGGGAAGGATATCGAAGGGGTGGGGAGTGAAGATCTGGTTAACTACATCGACGTGGGCCTGACCTACTACTTCAACAAAAACATGAACGCCTTCGTGGATTACAAAATCAACCAGCTGAAAAGCGATAACAAACTCGGCATCAACGATGACGACATCGTCGCGCTGGGTATCACCTACCAGTTTTCTGATCAA

>infB

ATCCAAAAAGTGCTCGGCGAGCGGGTGGTATTACCCAGCACATCGGTGCTTACCACGTCGAAACCGACAACGGCATGATCACCTTCCTGGATACCCCGGGCCACGCCGCGTTTACCTCCATGCGTGCTCGTGGCGCGCAGGCGACGGATATCGTGGTTCTGGTGGTGGCGGCAGACGACGGCGTGATGCCGCAGACTATCGAAGCTATCCAGCACGCTAAAGCGGCGCAGGTACCGGTGGTAGTGGCGGTGAACAAGATCGATAAGCCAGAAGCCGATCCGGATCGCGTGAAGAACGAACTGTCCCAGTACGGCATCCTGCCGGAAGAGTGGGGCGGCGAGAGCCAGTTTGTCCACGTTTCCGCGAAAGCGGGTACCGGCATCGACGACCTGCTGGACGCGATCCTGCTGCAGGCTGAAGTTCTTGACCTGAAAGCGA

>tonB

AGGTTGGGCATCTCGACGCAGCCGATAGAGATCACAATGGTGGCGCCGGCCGATCTTGAGCCGCCTCCGGCGGCGCAGCCTGTCGTGGAGCCCGTTGTTGAACCCGAACCTGAGCCGGAGCCAGAGGTAGCGCCTGAACCGCCGAAAGAGGCGCCGGTGGTGATCCATAAACCGGAACCTAAGCCGAAGCCCAAACCTAAACCCAAGCCTAAGCCGGAGAAAAAGGTTGAACAGCCGAAGCGGGAAGTGAAGCCGGCAGCAGAGCCGCGTCCGGCCTCGCCGTTTGAAAACAACAATACGGCGCCGGCGCGTACAGCGCCAAGTACCTCGACCGCAGCGGCTAAACCCACCGTTACTGCTCCGAGCGGCCCGCGGGCGATCAGCCGCGTTCAGCCGTCCTATCCGCCGCGCGCTCAGGCGCTGCGCATTGAAGGGACGGTACGGGTGAAGTTTGACGTTTCGCCTGATGGCCGCATTGATAATCTGCAGATCCTCTCTGCTCAGCCGGGGCGAATAA

FO26 (ST11)

>rpoB

CGCGGTAGGTTGTAGGAGCGTGCGGTGAGAGCGTCTGTCTCTTGGCGATCTGGATACCCTGATGCCTCAGGATATGATCAACGCCAAGCCGATTTCCGCAGCAGTGAAAGAGTTCTTTGGTTCCAGCCAGCTGTCTCAGTTTATGGACCAGAACAACCCGCTGTCTGAGATTACGCACAAACGTCGTATCTCCGCACTCGGCCCAGGCGGTCTGACCCGTGAGCGCGCAGGCTTCGAAGTTCGAGACGTACACCCGACCCACTACGGTCGCGTATGTCCGATCGAAACGCCTGAAGGTCCGAACATCGGTCTGATTAACTCCCTGTCCGTGTACGCGCAGACCAACGAATATGGCTTCCTTGAGACGCCGTATCGTAAAGTGACCAACGGTGTGGTTACTGACGAAATTCACTACCTGTCTGCTATCGAAGAAGGCAACTACGTTATCGCTCAGGCGAACTCCAACCTGGATGAAAACGGCCACTTCGTAGAAGATCTGGTTACCTGCCGTAGCAAAGGCGAATCCAGCTTGTTCAGCCGCGACCAGGTTGACTACATGGACGTATCCACCCAGCAGGTGGTATCCGTCGGTGCGTCCCTGATCCCGTTCCTGGAACACGATGACGCCAACCGTGCATTGATGGGTGCGAACATGCAACGTCAGGCGGTTCCGACTCTGCGCGCTGATAAGCCGCTGGTTGGTACCGGTATGGAACGTGCTGTTGCCGTTGACTCCGGTGTTACTGCCGTGGCTAAACGTGGCGGTACCGTTCAGTACGTGGATGCTTCCCGTATCGTTATCAAAGTTAACGAAGACGAGATGTACCCGGGCGAAGCAGGTATCGACATCTATAACCTGACCAAGTACACCCGTTCTAACCAGAACACCTGCATCAACCAGATGCCTTGCGTGTCCCTGGGCGAACCTATTGAGCGCGGCGACGTGCTGGCAGACGGCCCGTCCACCGACCTCGGTGAGCTGGCGCTGGGTCAGAACATGCGTGTAGCGTTCATGCCGCG

>gapA

CGGTTTAAGATCGTTGAGTGAAGACGGTCATCTGGTCGTTAACGGTAAAAAAATCCGTGTTACCGCTGAACGTGACCCGGCTAACCTGAAGTGGGACGAAGTTGGTGTTGACGTTGTTGCTGAAGCAACCGGTATCTTCCTGACCGACGAAACCGCTCGTAAACACATCACCGCTGGCGCGAAAAAAGTCGTTCTGACTGGCCCGTCCAAAGACAACACTCCGATGTTCGTTCGCGGCGCTAACTTCGACGCTTACGCTGGCCAGGACATCGTTTCCAACGCTTCCTGCACCACTAACTGCCTGGCGCCGCTGGCTAAAGTTATCAACGACAACTTCGGTATCGTTGAAGGCCTGATGACCACCGTCCACGCTACCACCGCTACTCAGAAAACCGTTGATGGCCCGTCTCACAAAGACTGGCGCGGCGGCCGCGGCGCAGCTCAGAACATCATCCCGTCCTCTACCGGCGCTGCTAAAGCAGTAGGTAAAGTACTGCCAGAACTGAACGGCAAACTGACCGGTATGGCGTTCCGCGTTCCAACTCCGAACGTATCTGTTGTTGACCTGACCGTTCGTCTGGAAAAAGCAGCGTCCTACGAAGAAATCAAGAAAGCCATCAAAGCCCTTTTCTGAAG

>mdh

GCCGTAGACTCGCTCGGTTACGCCGGGCGTGGCGGTAGATCTAAGTCATATCCCCACAGATGTAAAAATTAAAGGATTTTCCGGTGAAGACGCTACTCCGGCGCTGGAAGGCGCGGATGTAGTGCTGATCTCCGCGGGCGTGGCGCGTAAGCCCGGCATGGATCGTTCCGACCTGTTTAATGTGAATGCGGGTATCGTGAAGAACCTCGTGCAGCAGATTGCCAAAACCTGCCCGCAGGCCTGCATCGGCATTATCACCAACCCGGTGAATACCACCGTGGCTATCGCCGCCGAAGTACTGAAAAAAGCCGGCGTGTACGATAAAAACAAACTGTTCGGCGTTACCACGCTGGACATCATCCGTTCCAATACCTTTGTGGCGGAGCTGAAAGGTAAATCGGCAACCGAGGTGGAAGTCCCGGTCATTGGTGGTCACTCCGGGGTCACCATTCTGCCTTTACTGTCGCAGATCCCCGGCGTCAGCTTTAGCGATCAGGAAATTGCCGACCTGACTAAACGTATTCAGAACGCCGGTACCGAAGTCGTGGAAGCGAAAGCGGGCGGCGGGTCGGCGACCTTGTCGATGGGCCAGGCGGCTGCCCGTTTTGGTCTCTCTCTGGTTCGCGCCATGCAGGGGGAAAAAGGCGTGGTGGAGTGCGCCTACGTGGAAGGCGACGGCCACTATGCGCGTTTCTTCTCCCAGCCGCTGCTGTGGGAAAAAAAAACGGGGA

>pgi

GACACCAGCTTCTTCGGTGCGGAACCGAAGCGATTCTGCCGTACGACCAGTACATGCACCGCTTTGCCGCTTACTTCCAGCAGGGCAACATGGAGTCCAACGGTAAGTATGTTGACCGTAACGGCCACGCGGTAGACTACCAGACTGGCCCAATCATCTGGGGTGAGCCGGGCACCAACGGTCAGCACGCGTTCTACCAGCTGATCCACCAGGGCACCAAAATGGTACCGTGCGATTTCATCGCTCCGGCTATCACCCACAACCCGCTGTCTGACCACCATCAGAAACTGCTGTCTAACTTCTTCGCCCAGACCGAGGCCCTGGCCTTTGGTAAATCCCGCGAAGTGGTTGAGCAGGAATATCGCGATCAGGGTAAAGACCCGGCGACCCTGGAGCACGTGGTGCCGTTCAAAGTGTTCGAAGGTAACCGCCCGACTAACTCCATCCTGCTGCGTGAGATCACCCCGTTCAGCCTCGGGGCGCTGATTGCCCTGTACGAGCACAAAATCTTCACCCAGGGCGCGATCCTCAACATCTTCACCTTTGACCAGTGGGGCGTTGAGCTGGGCAAACAGCTGGCTAACCGCATCCTGCCGGAGCTGAAAGACGGCAGCGAAGTTAGCAGCCACGACAGCTCTACTAACGGCCTGATTAACCGCTATAACGGGGGGGGCGGG

>phoE

GCAGCTAGCTGCTGAGGTCTGGATCTGACCCTGCAGTACCAGGGTAAAAACGAAGGCCGTGAAGCGAAGAAACAGAACGGCGACGGCGTCGGCACCTCGTTAAGCTATGATTTCGGCGGCAGCGACTTCGCCGTCAGCGCAGCCTACACCAGCTCCGACCGTACCAACGATCAGAACCTGCTGGCCCGCGGCCAGGGTTCGAAAGCGGAAGCCTGGGCGACCGGCCTGAAATATGACGCCAACAATATCTACCTGGCGACCATGTACTCTGAAACCCGCAAGATGACCCCGATCAGCGGCGGCTTTGCCAACAAAGCGCAGAACTTTGAAGCGGTGGCGCAGTATCAGTTCGACTTCGGTCTGCGTCCGTCCCTCGGCTATGTGCTGTCGAAAGGGAAGGATATCGAAGGGGTGGGGAGTGAAGATCTGGTTAACTACATCGACGTGGGCCTGACCTACTACTTCAACAAAAACATGAACGCCTTCGTGGATTACAAAATCAACCAGCTGAAAAGCGATAACAAACTCGGCATCAACGATGACGACATCGTCGCGCTGGGTATCACCTACCGTTTTTGGAATCAA

>infB

ATCCAAAAAGTGCTCGGCGAGCGGGTGGTATTACCCAGCACATCGGTGCTTACCACGTCGAAACCGACAACGGCATGATCACCTTCCTGGATACCCCGGGCCACGCCGCGTTTACCTCCATGCGTGCTCGTGGCGCGCAGGCGACGGATATCGTGGTTCTGGTGGTGGCGGCAGACGACGGCGTGATGCCGCAGACTATCGAAGCTATCCAGCACGCTAAAGCGGCGCAGGTACCGGTGGTAGTGGCGGTGAACAAGATCGATAAGCCAGAAGCCGATCCGGATCGCGTGAAGAACGAACTGTCCCAGTACGGCATCCTGCCGGAAGAGTGGGGCGGCGAGAGCCAGTTTGTCCACGTTTCCGCGAAAGCGGGTACCGGCATCGACGACCTGCTGGACGCGATCCTGCTGCAGGCTGAAGTTCTTGACCTGAAAGCGCC

>tonB

AGGTTGGGCATCTCGACGCAGCCGATAGAGATCACAATGGTGGCGCCGGCCGATCTTGAGCCGCCTCCGGCGGCGCAGCCTGTCGTGGAGCCCGTTGTTGAACCCGAACCTGAGCCGGAGCCAGAGGTAGCGCCTGAACCGCCGAAAGAGGCGCCGGTGGTGATCCATAAACCGGAACCTAAGCCGAAGCCCAAACCTAAACCCAAGCCTAAGCCGGAGAAAAAGGTTGAACAGCCGAAGCGGGAAGTGAAGCCGGCAGCAGAGCCGCGTCCGGCCTCGCCGTTTGAAAACAACAATACGGCGCCGGCGCGTACAGCGCCAAGTACCTCGACCGCAGCGGCTAAACCCACCGTTACTGCTCCGAGCGGCCCGCGGGCGATCAGCCGCGTTCAGCCGTCCTATCCGCCGCGCGCTCAGGCGCTGCGCATTGAAGGGACGGTACGGGTGAAGTTTGACGTTTCGCCTGATGGCCGCATTGATAATCTGCAGATCCTCTCTGCTCAGCCGGGGCGAATCT

FO27 (ST11)

>rpoB

ATCGGTAGGTTGTAGGAGCGTGCGGTGAGAGCGTCTGTCTCTTGGCGATCTGGATACCCTGATGCCTCAGGATATGATCAACGCCAAGCCGATTTCCGCAGCAGTGAAAGAGTTCTTTGGTTCCAGCCAGCTGTCTCAGTTTATGGACCAGAACAACCCGCTGTCTGAGATTACGCACAAACGTCGTATCTCCGCACTCGGCCCAGGCGGTCTGACCCGTGAGCGCGCAGGCTTCGAAGTTCGAGACGTACACCCGACCCACTACGGTCGCGTATGTCCGATCGAAACGCCTGAAGGTCCGAACATCGGTCTGATTAACTCCCTGTCCGTGTACGCGCAGACCAACGAATATGGCTTCCTTGAGACGCCGTATCGTAAAGTGACCAACGGTGTGGTTACTGACGAAATTCACTACCTGTCTGCTATCGAAGAAGGCAACTACGTTATCGCTCAGGCGAACTCCAACCTGGATGAAAACGGCCACTTCGTAGAAGATCTGGTTACCTGCCGTAGCAAAGGCGAATCCAGCTTGTTCAGCCGCGACCAGGTTGACTACATGGACGTATCCACCCAGCAGGTGGTATCCGTCGGTGCGTCCCTGATCCCGTTCCTGGAACACGATGACGCCAACCGTGCATTGATGGGTGCGAACATGCAACGTCAGGCGGTTCCGACTCTGCGCGCTGATAAGCCGCTGGTTGGTACCGGTATGGAACGTGCTGTTGCCGTTGACTCCGGTGTTACTGCCGTGGCTAAACGTGGCGGTACCGTTCAGTACGTGGATGCTTCCCGTATCGTTATCAAAGTTAACGAAGACGAGATGTACCCGGGCGAAGCAGGTATCGACATCTATAACCTGACCAAGTACACCCGTTCTAACCAGAACACCTGCATCAACCAGATGCCTTGCGTGTCCCTGGGCGAACCTATTGAGCGCGGCGACGTGCTGGCAGACGGCCCGTCCACCGACCTCGGTGAGCTGGCGCTGGGTCAGAACATGCGTGTAGCGTTCATGCCGCG

>gapA

CGGTTTAAGATCGTTGAGTGAAGACGGTCATCTGGTCGTTAACGGTAAAAAAATCCGTGTTACCGCTGAACGTGACCCGGCTAACCTGAAGTGGGACGAAGTTGGTGTTGACGTTGTTGCTGAAGCAACCGGTATCTTCCTGACCGACGAAACCGCTCGTAAACACATCACCGCTGGCGCGAAAAAAGTCGTTCTGACTGGCCCGTCCAAAGACAACACTCCGATGTTCGTTCGCGGCGCTAACTTCGACGCTTACGCTGGCCAGGACATCGTTTCCAACGCTTCCTGCACCACTAACTGCCTGGCGCCGCTGGCTAAAGTTATCAACGACAACTTCGGTATCGTTGAAGGCCTGATGACCACCGTCCACGCTACCACCGCTACTCAGAAAACCGTTGATGGCCCGTCTCACAAAGACTGGCGCGGCGGCCGCGGCGCAGCTCAGAACATCATCCCGTCCTCTACCGGCGCTGCTAAAGCAGTAGGTAAAGTACTGCCAGAACTGAACGGCAAACTGACCGGTATGGCGTTCCGCGTTCCAACTCCGAACGTATCTGTTGTTGACCTGACCGTTCGTCTGGAAAAAGCAGCGTCCTACGAAGAAATCAAGAAAGCCATCAAAGCCCTTTTCTGAAG

>mdh

CGCATTTGACTCGCTCGGTTACGCCGGGCGTGGCGGTAGATCTAAGTCATATCCCCACAGATGTAAAAATTAAAGGATTTTCCGGTGAAGACGCTACTCCGGCGCTGGAAGGCGCGGATGTAGTGCTGATCTCCGCGGGCGTGGCGCGTAAGCCCGGCATGGATCGTTCCGACCTGTTTAATGTGAATGCGGGTATCGTGAAGAACCTCGTGCAGCAGATTGCCAAAACCTGCCCGCAGGCCTGCATCGGCATTATCACCAACCCGGTGAATACCACCGTGGCTATCGCCGCCGAAGTACTGAAAAAAGCCGGCGTGTACGATAAAAACAAACTGTTCGGCGTTACCACGCTGGACATCATCCGTTCCAATACCTTTGTGGCGGAGCTGAAAGGTAAATCGGCAACCGAGGTGGAAGTCCCGGTCATTGGTGGTCACTCCGGGGTCACCATTCTGCCTTTACTGTCGCAGATCCCCGGCGTCAGCTTTAGCGATCAGGAAATTGCCGACCTGACTAAACGTATTCAGAACGCCGGTACCGAAGTCGTGGAAGCGAAAGCGGGCGGCGGGTCGGCGACCTTGTCGATGGGCCAGGCGGCTGCCCGTTTTGGTCTCTCTCTGGTTCGCGCCATGCAGGGGGAAAAAGGCGTGGTGGAGTGCGCCTACGTGGAAGGCGACGGCCACTATGCGCGTTTCTTCTCCCAGCCGCTGTGGGGAGGAAAAAACAGCACGGA

>pgi

GACACCAGCTTCTTCGGTGCGGAACCGAAGCGATTCTGCCGTACGACCAGTACATGCACCGCTTTGCCGCTTACTTCCAGCAGGGCAACATGGAGTCCAACGGTAAGTATGTTGACCGTAACGGCCACGCGGTAGACTACCAGACTGGCCCAATCATCTGGGGTGAGCCGGGCACCAACGGTCAGCACGCGTTCTACCAGCTGATCCACCAGGGCACCAAAATGGTACCGTGCGATTTCATCGCTCCGGCTATCACCCACAACCCGCTGTCTGACCACCATCAGAAACTGCTGTCTAACTTCTTCGCCCAGACCGAGGCCCTGGCCTTTGGTAAATCCCGCGAAGTGGTTGAGCAGGAATATCGCGATCAGGGTAAAGACCCGGCGACCCTGGAGCACGTGGTGCCGTTCAAAGTGTTCGAAGGTAACCGCCCGACTAACTCCATCCTGCTGCGTGAGATCACCCCGTTCAGCCTCGGGGCGCTGATTGCCCTGTACGAGCACAAAATCTTCACCCAGGGCGCGATCCTCAACATCTTCACCTTTGACCAGTGGGGCGTTGAGCTGGGCAAACAGCTGGCTAACCGCATCCTGCCGGAGCTGAAAGACGGCAGCGAAGTTAGCAGCCACGACAGCTCTACTAACGGCCTGATTAACCGCTATAACGGGGGGGGCGGG

>phoE

AAAGCTTGCTGCTGTCTGGATCTGACCCTGCAGTACCAGGGTAAAAACGAAGGCCGTGAAGCGAAGAAACAGAACGGCGACGGCGTCGGCACCTCGTTAAGCTATGATTTCGGCGGCAGCGACTTCGCCGTCAGCGCAGCCTACACCAGCTCCGACCGTACCAACGATCAGAACCTGCTGGCCCGCGGCCAGGGTTCGAAAGCGGAAGCCTGGGCGACCGGCCTGAAATATGACGCCAACAATATCTACCTGGCGACCATGTACTCTGAAACCCGCAAGATGACCCCGATCAGCGGCGGCTTTGCCAACAAAGCGCAGAACTTTGAAGCGGTGGCGCAGTATCAGTTCGACTTCGGTCTGCGTCCGTCCCTCGGCTATGTGCTGTCGAAAGGGAAGGATATCGAAGGGGTGGGGAGTGAAGATCTGGTTAACTACATCGACGTGGGCCTGACCTACTACTTCAACAAAAACATGAACGCCTTCGTGGATTACAAAATCAACCAGCTGAAAAGCGATAACAAACTCGGCATCAACGATGACGACATCGTCGCGCTGGGTATCACCTACGTTTTTTAAAAACAAGT

>infB

GACCAAAAAGTGCTCGGCGAGCGGGTGGTATTACCCAGCACATCGGTGCTTACCACGTCGAAACCGACAACGGCATGATCACCTTCCTGGATACCCCGGGCCACGCCGCGTTTACCTCCATGCGTGCTCGTGGCGCGCAGGCGACGGATATCGTGGTTCTGGTGGTGGCGGCAGACGACGGCGTGATGCCGCAGACTATCGAAGCTATCCAGCACGCTAAAGCGGCGCAGGTACCGGTGGTAGTGGCGGTGAACAAGATCGATAAGCCAGAAGCCGATCCGGATCGCGTGAAGAACGAACTGTCCCAGTACGGCATCCTGCCGGAAGAGTGGGGCGGCGAGAGCCAGTTTGTCCACGTTTCCGCGAAAGCGGGTACCGGCATCGACGACCTGCTGGACGCGATCCTGCTGCAGGCTGAAGTTCTTGACCTGAAAGCGTT

>tonB

AGGTTGGGCATCTCGACGCAGCCGATAGAGATCACAATGGTGGCGCCGGCCGATCTTGAGCCGCCTCCGGCGGCGCAGCCTGTCGTGGAGCCCGTTGTTGAACCCGAACCTGAGCCGGAGCCAGAGGTAGCGCCTGAACCGCCGAAAGAGGCGCCGGTGGTGATCCATAAACCGGAACCTAAGCCGAAGCCCAAACCTAAACCCAAGCCTAAGCCGGAGAAAAAGGTTGAACAGCCGAAGCGGGAAGTGAAGCCGGCAGCAGAGCCGCGTCCGGCCTCGCCGTTTGAAAACAACAATACGGCGCCGGCGCGTACAGCGCCAAGTACCTCGACCGCAGCGGCTAAACCCACCGTTACTGCTCCGAGCGGCCCGCGGGCGATCAGCCGCGTTCAGCCGTCCTATCCGCCGCGCGCTCAGGCGCTGCGCATTGAAGGGACGGTACGGGTGAAGTTTGACGTTTCGCCTGATGGCCGCATTGATAATCTGCAGATCCTCTCTGCTCAGCCGGGGCGAATCT

FO28 (ST11)

>rpoB

ATCGGTAGGTTGTAGGAGCGTGCGGTGAGAGCGTCTGTCTCTTGGCGATCTGGATACCCTGATGCCTCAGGATATGATCAACGCCAAGCCGATTTCCGCAGCAGTGAAAGAGTTCTTTGGTTCCAGCCAGCTGTCTCAGTTTATGGACCAGAACAACCCGCTGTCTGAGATTACGCACAAACGTCGTATCTCCGCACTCGGCCCAGGCGGTCTGACCCGTGAGCGCGCAGGCTTCGAAGTTCGAGACGTACACCCGACCCACTACGGTCGCGTATGTCCGATCGAAACGCCTGAAGGTCCGAACATCGGTCTGATTAACTCCCTGTCCGTGTACGCGCAGACCAACGAATATGGCTTCCTTGAGACGCCGTATCGTAAAGTGACCAACGGTGTGGTTACTGACGAAATTCACTACCTGTCTGCTATCGAAGAAGGCAACTACGTTATCGCTCAGGCGAACTCCAACCTGGATGAAAACGGCCACTTCGTAGAAGATCTGGTTACCTGCCGTAGCAAAGGCGAATCCAGCTTGTTCAGCCGCGACCAGGTTGACTACATGGACGTATCCACCCAGCAGGTGGTATCCGTCGGTGCGTCCCTGATCCCGTTCCTGGAACACGATGACGCCAACCGTGCATTGATGGGTGCGAACATGCAACGTCAGGCGGTTCCGACTCTGCGCGCTGATAAGCCGCTGGTTGGTACCGGTATGGAACGTGCTGTTGCCGTTGACTCCGGTGTTACTGCCGTGGCTAAACGTGGCGGTACCGTTCAGTACGTGGATGCTTCCCGTATCGTTATCAAAGTTAACGAAGACGAGATGTACCCGGGCGAAGCAGGTATCGACATCTATAACCTGACCAAGTACACCCGTTCTAACCAGAACACCTGCATCAACCAGATGCCTTGCGTGTCCCTGGGCGAACCTATTGAGCGCGGCGACGTGCTGGCAGACGGCCCGTCCACCGACCTCGGTGAGCTGGCGCTGGGTCAGAACATGCGTGTAGCGTTCATGCCGCG

>gapA

CGGTTTAAGATCGTTGAGTGAAGACGGTCATCTGGTCGTTAACGGTAAAAAAATCCGTGTTACCGCTGAACGTGACCCGGCTAACCTGAAGTGGGACGAAGTTGGTGTTGACGTTGTTGCTGAAGCAACCGGTATCTTCCTGACCGACGAAACCGCTCGTAAACACATCACCGCTGGCGCGAAAAAAGTCGTTCTGACTGGCCCGTCCAAAGACAACACTCCGATGTTCGTTCGCGGCGCTAACTTCGACGCTTACGCTGGCCAGGACATCGTTTCCAACGCTTCCTGCACCACTAACTGCCTGGCGCCGCTGGCTAAAGTTATCAACGACAACTTCGGTATCGTTGAAGGCCTGATGACCACCGTCCACGCTACCACCGCTACTCAGAAAACCGTTGATGGCCCGTCTCACAAAGACTGGCGCGGCGGCCGCGGCGCAGCTCAGAACATCATCCCGTCCTCTACCGGCGCTGCTAAAGCAGTAGGTAAAGTACTGCCAGAACTGAACGGCAAACTGACCGGTATGGCGTTCCGCGTTCCAACTCCGAACGTATCTGTTGTTGACCTGACCGTTCGTCTGGAAAAAGCAGCGTCCTACGAAGAAATCAAGAAAGCCATCAAAGCCCTTTTCTGAAG

>mdh

GCATTTGACTCGCTCGGTTACGCCGGGCGTGGCGGTAGATCTAAGTCATATCCCCACAGATGTAAAAATTAAAGGATTTTCCGGTGAAGACGCTACTCCGGCGCTGGAAGGCGCGGATGTAGTGCTGATCTCCGCGGGCGTGGCGCGTAAGCCCGGCATGGATCGTTCCGACCTGTTTAATGTGAATGCGGGTATCGTGAAGAACCTCGTGCAGCAGATTGCCAAAACCTGCCCGCAGGCCTGCATCGGCATTATCACCAACCCGGTGAATACCACCGTGGCTATCGCCGCCGAAGTACTGAAAAAAGCCGGCGTGTACGATAAAAACAAACTGTTCGGCGTTACCACGCTGGACATCATCCGTTCCAATACCTTTGTGGCGGAGCTGAAAGGTAAATCGGCAACCGAGGTGGAAGTCCCGGTCATTGGTGGTCACTCCGGGGTCACCATTCTGCCTTTACTGTCGCAGATCCCCGGCGTCAGCTTTAGCGATCAGGAAATTGCCGACCTGACTAAACGTATTCAGAACGCCGGTACCGAAGTCGTGGAAGCGAAAGCGGGCGGCGGGTCGGCGACCTTGTCGATGGGCCAGGCGGCTGCCCGTTTTGGTCTCTCTCTGGTTCGCGCCATGCAGGGGGAAAAAGGCGTGGTGGAGTGCGCCTACGTGGAAGGCGACGGCCACTATGCGCGTTTCTTCTCCCAGCCGCTGCTGTGGAGGGAAAAAAAACGAGA

>pgi

GACACCAGCTTCTTCGGTGCGGAACCGAAGCGATTCTGCCGTACGACCAGTACATGCACCGCTTTGCCGCTTACTTCCAGCAGGGCAACATGGAGTCCAACGGTAAGTATGTTGACCGTAACGGCCACGCGGTAGACTACCAGACTGGCCCAATCATCTGGGGTGAGCCGGGCACCAACGGTCAGCACGCGTTCTACCAGCTGATCCACCAGGGCACCAAAATGGTACCGTGCGATTTCATCGCTCCGGCTATCACCCACAACCCGCTGTCTGACCACCATCAGAAACTGCTGTCTAACTTCTTCGCCCAGACCGAGGCCCTGGCCTTTGGTAAATCCCGCGAAGTGGTTGAGCAGGAATATCGCGATCAGGGTAAAGACCCGGCGACCCTGGAGCACGTGGTGCCGTTCAAAGTGTTCGAAGGTAACCGCCCGACTAACTCCATCCTGCTGCGTGAGATCACCCCGTTCAGCCTCGGGGCGCTGATTGCCCTGTACGAGCACAAAATCTTCACCCAGGGCGCGATCCTCAACATCTTCACCTTTGACCAGTGGGGCGTTGAGCTGGGCAAACAGCTGGCTAACCGCATCCTGCCGGAGCTGAAAGACGGCAGCGAAGTTAGCAGCCACGACAGCTCTACTAACGGCCTGATTAACCGCTATAACGGGGGGGGCGGG

>phoE

ACCGTAGCTCTCAGTATGAGATCTGACCCTGCAGTACCAGGGTAAAAACGAAGGCCGTGAAGCGAAGAAACAGAACGGCGACGGCGTCGGCACCTCGTTAAGCTATGATTTCGGCGGCAGCGACTTCGCCGTCAGCGCAGCCTACACCAGCTCCGACCGTACCAACGATCAGAACCTGCTGGCCCGCGGCCAGGGTTCGAAAGCGGAAGCCTGGGCGACCGGCCTGAAATATGACGCCAACAATATCTACCTGGCGACCATGTACTCTGAAACCCGCAAGATGACCCCGATCAGCGGCGGCTTTGCCAACAAAGCGCAGAACTTTGAAGCGGTGGCGCAGTATCAGTTCGACTTCGGTCTGCGTCCGTCCCTCGGCTATGTGCTGTCGAAAGGGAAGGATATCGAAGGGGTGGGGAGTGAAGATCTGGTTAACTACATCGACGTGGGCCTGACCTACTACTTCAACAAAAACATGAACGCCTTCGTGGATTACAAAATCAACCAGCTGAAAAGCGATAACAAACTCGGCATCAACGATGACGACATCGTCGCGCTGGGTATCACCTACCTTTTTCTGGATCAAA

>infB

GACCAAAAAGTGCTCGGCGAGCGGGTGGTATTACCCAGCACATCGGTGCTTACCACGTCGAAACCGACAACGGCATGATCACCTTCCTGGATACCCCGGGCCACGCCGCGTTTACCTCCATGCGTGCTCGTGGCGCGCAGGCGACGGATATCGTGGTTCTGGTGGTGGCGGCAGACGACGGCGTGATGCCGCAGACTATCGAAGCTATCCAGCACGCTAAAGCGGCGCAGGTACCGGTGGTAGTGGCGGTGAACAAGATCGATAAGCCAGAAGCCGATCCGGATCGCGTGAAGAACGAACTGTCCCAGTACGGCATCCTGCCGGAAGAGTGGGGCGGCGAGAGCCAGTTTGTCCACGTTTCCGCGAAAGCGGGTACCGGCATCGACGACCTGCTGGACGCGATCCTGCTGCAGGCTGAAGTTCTTGACCTGAAAGCGTT

>tonB

AGGTTGGGCATCTCGACGCAGCCGATAGAGATCACAATGGTGGCGCCGGCCGATCTTGAGCCGCCTCCGGCGGCGCAGCCTGTCGTGGAGCCCGTTGTTGAACCCGAACCTGAGCCGGAGCCAGAGGTAGCGCCTGAACCGCCGAAAGAGGCGCCGGTGGTGATCCATAAACCGGAACCTAAGCCGAAGCCCAAACCTAAACCCAAGCCTAAGCCGGAGAAAAAGGTTGAACAGCCGAAGCGGGAAGTGAAGCCGGCAGCAGAGCCGCGTCCGGCCTCGCCGTTTGAAAACAACAATACGGCGCCGGCGCGTACAGCGCCAAGTACCTCGACCGCAGCGGCTAAACCCACCGTTACTGCTCCGAGCGGCCCGCGGGCGATCAGCCGCGTTCAGCCGTCCTATCCGCCGCGCGCTCAGGCGCTGCGCATTGAAGGGACGGTACGGGTGAAGTTTGACGTTTCGCCTGATGGCCGCATTGATAATCTGCAGATCCTCTCTGCTCAGCCGGGGCGAATCT

FO29 (ST11)

>rpoB

ATCGGTAGGTTGTAGGAGCGTGCGGTGAGAGCGTCTGTCTCTTGGCGATCTGGATACCCTGATGCCTCAGGATATGATCAACGCCAAGCCGATTTCCGCAGCAGTGAAAGAGTTCTTTGGTTCCAGCCAGCTGTCTCAGTTTATGGACCAGAACAACCCGCTGTCTGAGATTACGCACAAACGTCGTATCTCCGCACTCGGCCCAGGCGGTCTGACCCGTGAGCGCGCAGGCTTCGAAGTTCGAGACGTACACCCGACCCACTACGGTCGCGTATGTCCGATCGAAACGCCTGAAGGTCCGAACATCGGTCTGATTAACTCCCTGTCCGTGTACGCGCAGACCAACGAATATGGCTTCCTTGAGACGCCGTATCGTAAAGTGACCAACGGTGTGGTTACTGACGAAATTCACTACCTGTCTGCTATCGAAGAAGGCAACTACGTTATCGCTCAGGCGAACTCCAACCTGGATGAAAACGGCCACTTCGTAGAAGATCTGGTTACCTGCCGTAGCAAAGGCGAATCCAGCTTGTTCAGCCGCGACCAGGTTGACTACATGGACGTATCCACCCAGCAGGTGGTATCCGTCGGTGCGTCCCTGATCCCGTTCCTGGAACACGATGACGCCAACCGTGCATTGATGGGTGCGAACATGCAACGTCAGGCGGTTCCGACTCTGCGCGCTGATAAGCCGCTGGTTGGTACCGGTATGGAACGTGCTGTTGCCGTTGACTCCGGTGTTACTGCCGTGGCTAAACGTGGCGGTACCGTTCAGTACGTGGATGCTTCCCGTATCGTTATCAAAGTTAACGAAGACGAGATGTACCCGGGCGAAGCAGGTATCGACATCTATAACCTGACCAAGTACACCCGTTCTAACCAGAACACCTGCATCAACCAGATGCCTTGCGTGTCCCTGGGCGAACCTATTGAGCGCGGCGACGTGCTGGCAGACGGCCCGTCCACCGACCTCGGTGAGCTGGCGCTGGGTCAGAACATGCGTGTAGCGTTCATGCCGCG

>gapA

CGGTTTAAGATCGTTGAGTGAAGACGGTCATCTGGTCGTTAACGGTAAAAAAATCCGTGTTACCGCTGAACGTGACCCGGCTAACCTGAAGTGGGACGAAGTTGGTGTTGACGTTGTTGCTGAAGCAACCGGTATCTTCCTGACCGACGAAACCGCTCGTAAACACATCACCGCTGGCGCGAAAAAAGTCGTTCTGACTGGCCCGTCCAAAGACAACACTCCGATGTTCGTTCGCGGCGCTAACTTCGACGCTTACGCTGGCCAGGACATCGTTTCCAACGCTTCCTGCACCACTAACTGCCTGGCGCCGCTGGCTAAAGTTATCAACGACAACTTCGGTATCGTTGAAGGCCTGATGACCACCGTCCACGCTACCACCGCTACTCAGAAAACCGTTGATGGCCCGTCTCACAAAGACTGGCGCGGCGGCCGCGGCGCAGCTCAGAACATCATCCCGTCCTCTACCGGCGCTGCTAAAGCAGTAGGTAAAGTACTGCCAGAACTGAACGGCAAACTGACCGGTATGGCGTTCCGCGTTCCAACTCCGAACGTATCTGTTGTTGACCTGACCGTTCGTCTGGAAAAAGCAGCGTCCTACGAAGAAATCAAGAAAGCCATCAAAGCCCTTTTCTGAAG

>mdh

GAGCGCGAGACTCGCTCGGTTACGCCGGGCGTGGCGGTAGATCTAAGTCATATCCCCACAGATGTAAAAATTAAAGGATTTTCCGGTGAAGACGCTACTCCGGCGCTGGAAGGCGCGGATGTAGTGCTGATCTCCGCGGGCGTGGCGCGTAAGCCCGGCATGGATCGTTCCGACCTGTTTAATGTGAATGCGGGTATCGTGAAGAACCTCGTGCAGCAGATTGCCAAAACCTGCCCGCAGGCCTGCATCGGCATTATCACCAACCCGGTGAATACCACCGTGGCTATCGCCGCCGAAGTACTGAAAAAAGCCGGCGTGTACGATAAAAACAAACTGTTCGGCGTTACCACGCTGGACATCATCCGTTCCAATACCTTTGTGGCGGAGCTGAAAGGTAAATCGGCAACCGAGGTGGAAGTCCCGGTCATTGGTGGTCACTCCGGGGTCACCATTCTGCCTTTACTGTCGCAGATCCCCGGCGTCAGCTTTAGCGATCAGGAAATTGCCGACCTGACTAAACGTATTCAGAACGCCGGTACCGAAGTCGTGGAAGCGAAAGCGGGCGGCGGGTCGGCGACCTTGTCGATGGGCCAGGCGGCTGCCCGTTTTGGTCTCTCTCTGGTTCGCGCCATGCAGGGGGAAAAAGGCGTGGTGGAGTGCGCCTACGTGGAAGGCGACGGCCACTATGCGCGTTTCTTCTCCCAGCCGCTGCTGAGGAGAAAAAAACGACGAAAA

>pgi

GACACCAGCTTCTTCGGTGCGGAACCGAAGCGATTCTGCCGTACGACCAGTACATGCACCGCTTTGCCGCTTACTTCCAGCAGGGCAACATGGAGTCCAACGGTAAGTATGTTGACCGTAACGGCCACGCGGTAGACTACCAGACTGGCCCAATCATCTGGGGTGAGCCGGGCACCAACGGTCAGCACGCGTTCTACCAGCTGATCCACCAGGGCACCAAAATGGTACCGTGCGATTTCATCGCTCCGGCTATCACCCACAACCCGCTGTCTGACCACCATCAGAAACTGCTGTCTAACTTCTTCGCCCAGACCGAGGCCCTGGCCTTTGGTAAATCCCGCGAAGTGGTTGAGCAGGAATATCGCGATCAGGGTAAAGACCCGGCGACCCTGGAGCACGTGGTGCCGTTCAAAGTGTTCGAAGGTAACCGCCCGACTAACTCCATCCTGCTGCGTGAGATCACCCCGTTCAGCCTCGGGGCGCTGATTGCCCTGTACGAGCACAAAATCTTCACCCAGGGCGCGATCCTCAACATCTTCACCTTTGACCAGTGGGGCGTTGAGCTGGGCAAACAGCTGGCTAACCGCATCCTGCCGGAGCTGAAAGACGGCAGCGAAGTTAGCAGCCACGACAGCTCTACTAACGGCCTGATTAACCGCTATAACGGGGGGGGCGGG

>phoE

GTACGCCGTAACTGCTGAGTCTGGATCTGACCTGCAGTACCAGGGTAAAAACGAAGGCCGTGAAGCGAAGAAACAGAACGGCGACGGCGTCGGCACCTCGTTAAGCTATGATTTCGGCGGCAGCGACTTCGCCGTCAGCGCAGCCTACACCAGCTCCGACCGTACCAACGATCAGAACCTGCTGGCCCGCGGCCAGGGTTCGAAAGCGGAAGCCTGGGCGACCGGCCTGAAATATGACGCCAACAATATCTACCTGGCGACCATGTACTCTGAAACCCGCAAGATGACCCCGATCAGCGGCGGCTTTGCCAACAAAGCGCAGAACTTTGAAGCGGTGGCGCAGTATCAGTTCGACTTCGGTCTGCGTCCGTCCCTCGGCTATGTGCTGTCGAAAGGGAAGGATATCGAAGGGGTGGGGAGTGAAGATCTGGTTAACTACATCGACGTGGGCCTGACCTACTACTTCAACAAAAACATGAACGCCTTCGTGGATTACAAAATCAACCAGCTGAAAAGCGATAACAAACTCGGCATCAACGATGACGACATCGTCGCGCTGGGTATCACCTACCGTTTTTGGACCAA

>infB

GACCAAAAAGTGCTCGGCGAGCGGGTGGTATTACCCAGCACATCGGTGCTTACCACGTCGAAACCGACAACGGCATGATCACCTTCCTGGATACCCCGGGCCACGCCGCGTTTACCTCCATGCGTGCTCGTGGCGCGCAGGCGACGGATATCGTGGTTCTGGTGGTGGCGGCAGACGACGGCGTGATGCCGCAGACTATCGAAGCTATCCAGCACGCTAAAGCGGCGCAGGTACCGGTGGTAGTGGCGGTGAACAAGATCGATAAGCCAGAAGCCGATCCGGATCGCGTGAAGAACGAACTGTCCCAGTACGGCATCCTGCCGGAAGAGTGGGGCGGCGAGAGCCAGTTTGTCCACGTTTCCGCGAAAGCGGGTACCGGCATCGACGACCTGCTGGACGCGATCCTGCTGCAGGCTGAAGTTCTTGACCTGAAAGCGTT

>tonB

AGGTTGGGCATCTCGACGCAGCCGATAGAGATCACAATGGTGGCGCCGGCCGATCTTGAGCCGCCTCCGGCGGCGCAGCCTGTCGTGGAGCCCGTTGTTGAACCCGAACCTGAGCCGGAGCCAGAGGTAGCGCCTGAACCGCCGAAAGAGGCGCCGGTGGTGATCCATAAACCGGAACCTAAGCCGAAGCCCAAACCTAAACCCAAGCCTAAGCCGGAGAAAAAGGTTGAACAGCCGAAGCGGGAAGTGAAGCCGGCAGCAGAGCCGCGTCCGGCCTCGCCGTTTGAAAACAACAATACGGCGCCGGCGCGTACAGCGCCAAGTACCTCGACCGCAGCGGCTAAACCCACCGTTACTGCTCCGAGCGGCCCGCGGGCGATCAGCCGCGTTCAGCCGTCCTATCCGCCGCGCGCTCAGGCGCTGCGCATTGAAGGGACGGTACGGGTGAAGTTTGACGTTTCGCCTGATGGCCGCATTGATAATCTGCAGATCCTCTCTGCTCAGCCGGGGCGAATCT

FO30 (ST11)

>rpoB

ATCGGTAGGTTGTAGGAGCGTGCGGTGAGAGCGTCTGTCTCTTGGCGATCTGGATACCCTGATGCCTCAGGATATGATCAACGCCAAGCCGATTTCCGCAGCAGTGAAAGAGTTCTTTGGTTCCAGCCAGCTGTCTCAGTTTATGGACCAGAACAACCCGCTGTCTGAGATTACGCACAAACGTCGTATCTCCGCACTCGGCCCAGGCGGTCTGACCCGTGAGCGCGCAGGCTTCGAAGTTCGAGACGTACACCCGACCCACTACGGTCGCGTATGTCCGATCGAAACGCCTGAAGGTCCGAACATCGGTCTGATTAACTCCCTGTCCGTGTACGCGCAGACCAACGAATATGGCTTCCTTGAGACGCCGTATCGTAAAGTGACCAACGGTGTGGTTACTGACGAAATTCACTACCTGTCTGCTATCGAAGAAGGCAACTACGTTATCGCTCAGGCGAACTCCAACCTGGATGAAAACGGCCACTTCGTAGAAGATCTGGTTACCTGCCGTAGCAAAGGCGAATCCAGCTTGTTCAGCCGCGACCAGGTTGACTACATGGACGTATCCACCCAGCAGGTGGTATCCGTCGGTGCGTCCCTGATCCCGTTCCTGGAACACGATGACGCCAACCGTGCATTGATGGGTGCGAACATGCAACGTCAGGCGGTTCCGACTCTGCGCGCTGATAAGCCGCTGGTTGGTACCGGTATGGAACGTGCTGTTGCCGTTGACTCCGGTGTTACTGCCGTGGCTAAACGTGGCGGTACCGTTCAGTACGTGGATGCTTCCCGTATCGTTATCAAAGTTAACGAAGACGAGATGTACCCGGGCGAAGCAGGTATCGACATCTATAACCTGACCAAGTACACCCGTTCTAACCAGAACACCTGCATCAACCAGATGCCTTGCGTGTCCCTGGGCGAACCTATTGAGCGCGGCGACGTGCTGGCAGACGGCCCGTCCACCGACCTCGGTGAGCTGGCGCTGGGTCAGAACATGCGTGTAGCGTTCATGCCGCG

>gapA

CGGTTTAAGATCGTTGAGTGAAGACGGTCATCTGGTCGTTAACGGTAAAAAAATCCGTGTTACCGCTGAACGTGACCCGGCTAACCTGAAGTGGGACGAAGTTGGTGTTGACGTTGTTGCTGAAGCAACCGGTATCTTCCTGACCGACGAAACCGCTCGTAAACACATCACCGCTGGCGCGAAAAAAGTCGTTCTGACTGGCCCGTCCAAAGACAACACTCCGATGTTCGTTCGCGGCGCTAACTTCGACGCTTACGCTGGCCAGGACATCGTTTCCAACGCTTCCTGCACCACTAACTGCCTGGCGCCGCTGGCTAAAGTTATCAACGACAACTTCGGTATCGTTGAAGGCCTGATGACCACCGTCCACGCTACCACCGCTACTCAGAAAACCGTTGATGGCCCGTCTCACAAAGACTGGCGCGGCGGCCGCGGCGCAGCTCAGAACATCATCCCGTCCTCTACCGGCGCTGCTAAAGCAGTAGGTAAAGTACTGCCAGAACTGAACGGCAAACTGACCGGTATGGCGTTCCGCGTTCCAACTCCGAACGTATCTGTTGTTGACCTGACCGTTCGTCTGGAAAAAGCAGCGTCCTACGAAGAAATCAAGAAAGCCATCAAAGCCCTTTTCTGAAG

>mdh

GCATCGTGACTCGCTCGGTTACGCCGGGCGTGGCGGTAGATCTAAGTCATATCCCCACAGATGTAAAAATTAAAGGATTTTCCGGTGAAGACGCTACTCCGGCGCTGGAAGGCGCGGATGTAGTGCTGATCTCCGCGGGCGTGGCGCGTAAGCCCGGCATGGATCGTTCCGACCTGTTTAATGTGAATGCGGGTATCGTGAAGAACCTCGTGCAGCAGATTGCCAAAACCTGCCCGCAGGCCTGCATCGGCATTATCACCAACCCGGTGAATACCACCGTGGCTATCGCCGCCGAAGTACTGAAAAAAGCCGGCGTGTACGATAAAAACAAACTGTTCGGCGTTACCACGCTGGACATCATCCGTTCCAATACCTTTGTGGCGGAGCTGAAAGGTAAATCGGCAACCGAGGTGGAAGTCCCGGTCATTGGTGGTCACTCCGGGGTCACCATTCTGCCTTTACTGTCGCAGATCCCCGGCGTCAGCTTTAGCGATCAGGAAATTGCCGACCTGACTAAACGTATTCAGAACGCCGGTACCGAAGTCGTGGAAGCGAAAGCGGGCGGCGGGTCGGCGACCTTGTCGATGGGCCAGGCGGCTGCCCGTTTTGGTCTCTCTCTGGTTCGCGCCATGCAGGGGGAAAAAGGCGTGGTGGAGTGCGCCTACGTGGAAGGCGACGGCCACTATGCGCGTTTCTTCTCCCAGCCGCTGCTCGGAAAAAAAAAACAGCGAAAA

>pgi

ACAGACGACAGTAGAGCTGTCGTGGCTGCTAACTTCGCTGCCGTCTTTCAGCTCCGGCAGGATGCGGTTAGCCAGCTGTTTGCCCAGCTCAACGCCCCACTGGTCAAAGGTGAAGATGTTGAGGATCGCGCCCTGGGTGAAGATTTTGTGCTCGTACAGGGCAATCAGCGCCCCGAGGCTGAACGGGGTGATCTCACGCAGCAGGATGGAGTTAGTCGGGCGGTTACCTTCGAACACTTTGAACGGCACCACGTGCTCCAGGGTCGCCGGGTCTTTACCCTGATCGCGATATTCCTGCTCAACCACTTCGCGGGATTTACCAAAGGCCAGGGCCTCGGTCTGGGCGAAGAAGTTAGACAGCAGTTTCTGATGGTGGTCAGACAGCGGGTTGTGGGTGATAGCCGGAGCGATGAAATCGCACGGTACCATTTTGGTGCCCTGGTGGATCAGCTGGTAGAACGCGTGCTGACCGTTGGTGCCCGGCTCACCCCAGATGATTGGGCCAGTCTGGTAGTCTACCGCGTGGCCGTTACGGTCAACATACTTACCGTTGGACTCCATGTTGCCCTGCTGGAAGTAAGCGGCAAAGCGGTGCATGTACTGGTCGTACGGCAGAATCGCTTCGGTTTCCGCACCGAAGAAGTTGTTGTACCAGATGCCGATCAGAGCCAGCAGTACAGGAGGTTTTTTTTTTCATCA

>phoE

GTACGCCGTAACTGCTGAGTCTGGATCTGACCTGCAGTACCAGGGTAAAAACGAAGGCCGTGAAGCGAAGAAACAGAACGGCGACGGCGTCGGCACCTCGTTAAGCTATGATTTCGGCGGCAGCGACTTCGCCGTCAGCGCAGCCTACACCAGCTCCGACCGTACCAACGATCAGAACCTGCTGGCCCGCGGCCAGGGTTCGAAAGCGGAAGCCTGGGCGACCGGCCTGAAATATGACGCCAACAATATCTACCTGGCGACCATGTACTCTGAAACCCGCAAGATGACCCCGATCAGCGGCGGCTTTGCCAACAAAGCGCAGAACTTTGAAGCGGTGGCGCAGTATCAGTTCGACTTCGGTCTGCGTCCGTCCCTCGGCTATGTGCTGTCGAAAGGGAAGGATATCGAAGGGGTGGGGAGTGAAGATCTGGTTAACTACATCGACGTGGGCCTGACCTACTACTTCAACAAAAACATGAACGCCTTCGTGGATTACAAAATCAACCAGCTGAAAAGCGATAACAAACTCGGCATCAACGATGACGACATCGTCGCGCTGGGTATCACCTACCGTTTTTGGACCAA

>infB

GACCAAAAAGTGCTCGGCGAGCGGGTGGTATTACCCAGCACATCGGTGCTTACCACGTCGAAACCGACAACGGCATGATCACCTTCCTGGATACCCCGGGCCACGCCGCGTTTACCTCCATGCGTGCTCGTGGCGCGCAGGCGACGGATATCGTGGTTCTGGTGGTGGCGGCAGACGACGGCGTGATGCCGCAGACTATCGAAGCTATCCAGCACGCTAAAGCGGCGCAGGTACCGGTGGTAGTGGCGGTGAACAAGATCGATAAGCCAGAAGCCGATCCGGATCGCGTGAAGAACGAACTGTCCCAGTACGGCATCCTGCCGGAAGAGTGGGGCGGCGAGAGCCAGTTTGTCCACGTTTCCGCGAAAGCGGGTACCGGCATCGACGACCTGCTGGACGCGATCCTGCTGCAGGCTGAAGTTCTTGACCTGAAAGCGTT

>tonB

AGGTTGGGCATCTCGACGCAGCCGATAGAGATCACAATGGTGGCGCCGGCCGATCTTGAGCCGCCTCCGGCGGCGCAGCCTGTCGTGGAGCCCGTTGTTGAACCCGAACCTGAGCCGGAGCCAGAGGTAGCGCCTGAACCGCCGAAAGAGGCGCCGGTGGTGATCCATAAACCGGAACCTAAGCCGAAGCCCAAACCTAAACCCAAGCCTAAGCCGGAGAAAAAGGTTGAACAGCCGAAGCGGGAAGTGAAGCCGGCAGCAGAGCCGCGTCCGGCCTCGCCGTTTGAAAACAACAATACGGCGCCGGCGCGTACAGCGCCAAGTACCTCGACCGCAGCGGCTAAACCCACCGTTACTGCTCCGAGCGGCCCGCGGGCGATCAGCCGCGTTCAGCCGTCCTATCCGCCGCGCGCTCAGGCGCTGCGCATTGAAGGGACGGTACGGGTGAAGTTTGACGTTTCGCCTGATGGCCGCATTGATAATCTGCAGATCCTCTCTGCTCAGCCGGGGCGAATCT

FO31 (ST11)

>rpoB

CGTGACTATGGCTATCGTGTAGAGCGTGCGGTGAAGAGCGTCTGTCTCTTGGCGATCTGGATACCCTGATGCCTCAGGATATGATCAACGCCAAGCCGATTTCCGCAGCAGTGAAAGAGTTCTTTGGTTCCAGCCAGCTGTCTCAGTTTATGGACCAGAACAACCCGCTGTCTGAGATTACGCACAAACGTCGTATCTCCGCACTCGGCCCAGGCGGTCTGACCCGTGAGCGCGCAGGCTTCGAAGTTCGAGACGTACACCCGACCCACTACGGTCGCGTATGTCCGATCGAAACGCCTGAAGGTCCGAACATCGGTCTGATTAACTCCCTGTCCGTGTACGCGCAGACCAACGAATATGGCTTCCTTGAGACGCCGTATCGTAAAGTGACCAACGGTGTGGTTACTGACGAAATTCACTACCTGTCTGCTATCGAAGAAGGCAACTACGTTATCGCTCAGGCGAACTCCAACCTGGATGAAAACGGCCACTTCGTAGAAGATCTGGTTACCTGCCGTAGCAAAGGCGAATCCAGCTTGTTCAGCCGCGACCAGGTTGACTACATGGACGTATCCACCCAGCAGGTGGTATCCGTCGGTGCGTCCCTGATCCCGTTCCTGGAACACGATGACGCCAACCGTGCATTGATGGGTGCGAACATGCAACGTCAGGCGGTTCCGACTCTGCGCGCTGATAAGCCGCTGGTTGGTACCGGTATGGAACGTGCTGTTGCCGTTGACTCCGGTGTTACTGCCGTGGCTAAACGTGGCGGTACCGTTCAGTACGTGGATGCTTCCCGTATCGTTATCAAAGTTAACGAAGACGAGATGTACCCGGGCGAAGCAGTATCGACATCTATAACCTGACCAAGTACACCCGTTCTAACCAGAACACCTGCATCAACCAGATGCCTTGCGTGTCCCTGGGCGAACCTATTGAGCGCGGCGACGTGCTGGCAGACGCCCGTCCACCGACCTCGGTGAGCTGGCGCTGGGTCAGAACATGCGTGTAGCGTTCATGCCGTGACGTCTTACTCTCTATATACAATCTACTACAAA

>gapA

CCGATTCGAGATCGTTGAGTGAAGACGGTCATCTGGTCGTTAACGGTAAAAAAATCCGTGTTACCGCTGAACGTGACCCGGCTAACCTGAAGTGGGACGAAGTTGGTGTTGACGTTGTTGCTGAAGCAACCGGTATCTTCCTGACCGACAAAACCGCTCGTAAACACATCACCGCTGGCGCGAAAAAAGTCGTTCTGACTGGCCCGTCCAAAGACAACACTCCGATGTTCGTTCGCGGCGCTAACTTCGACGCTTACGCTGGCCAGGACATCGTTTCCAACGCTTCCTGCACCACTAACTGCCTGGCGCCGCTGGCTAAAGTTATCAACGACAACTTCGGTATCGTTGAAGGCCTGATGACCACCGTCCACGCTACCACCGCTACTCAGAAAACCGTTGATGGCCCGTCTCACAAAGACTGGCGCGGCGGCCGCGGCGCAGCTCAGAACATCATCCCGTCCTCTACCGGCGCTGCTAAAGCAGTAGGTAAAGTACTGCCAGAACTGAACGGCAAACTGACCGGTATGGCGTTCCGCGTTCCAACTCCGAACGTATCTGTTGTTGACCTGACCGTTCGTCTGGAAAAAGCAGCGTCCTACGAAGAAATCAAGAAAGCCATCAAAGCGTTTTTTCTAAAAGA

>mdh

CGCATCGTGACTCGCTCGGTTACGCCGGGCGTGGCGGTAGATCTAAGTCATATCCCCACAGATGTAAAAATTAAAGGATTTTCCGGTGAAGACGCTACTCCGGCGCTGGAAGGCGCGGATGTAGTGCTGATCTCCGCGGGCGTGGCGCGTAAGCCCGGCATGGATCGTTCCGACCTGTTTAATGTGAATGCGGGTATCGTGAAGAACCTCGTGCAGCAGATTGCCAAAACCTGCCCGCAGGCCTGCATCGGCATTATCACCAACCCGGTGAATACCACCGTGGCTATCGCCGCCGAAGTACTGAAAAAAGCCGGCGTGTACGATAAAAACAAACTGTTCGGCGTTACCACGCTGGACATCATCCGTTCCAATACCTTTGTGGCGGAGCTGAAAGGTAAATCGGCAACCGAGGTGGAAGTCCCGGTCATTGGTGGTCACTCCGGGGTCACCATTCTGCCTTTACTGTCGCAGATCCCCGGCGTCAGCTTTAGCGATCAGGAAATTGCCGACCTGACTAAACGTATTCAGAACGCCGGTACCGAAGTCGTGGAAGCGAAAGCGGGCGGCGGGTCGGCGACCTTGTCGATGGGCCAGGCGGCTGCCCGTTTTGGTCTCTCTCTGGTTCGCGCCATGCAGGGGGAAAAAGGCGTGGTGGAGTGCGCCTACGTGGAAGGCGACGGCCACTATGCGCGTTTCTTCTCCCAGCCGCTGCTGTGGAGAAAAAAAAACGAAA

>pgi

CTATTACGGTACGCTCTCTGGTCACACTTCTTCGGTGCGGAACCGAAGCGATTCTGCCGTACGACCAGTACATGCACCGCTTTGCCGCTTACTTCCAGCAGGGCAACATGGAGTCCAACGGTAAGTATGTTGACCGTAACGGCCACGCGGTAGACTACCAGACTGGCCCAATCATCTGGGGTGAGCCGGGCACCAACGGTCAGCACGCGTTCTACCAGCTGATCCACCAGGGCACCAAAATGGTACCGTGCGATTTCATCGCTCCGGCTATCACCCACAACCCGCTGTCTGACCACCATCAGAAACTGCTGTCTAACTTCTTCGCCCAGACCGAGGCCCTGGCCTTTGGTAAATCCCGCGAAGTGGTTGAGCAGGAATATCGCGATCAGGGTAAAGACCCGGCGACCCTGGAGCACGTGGTGCCGTTCAAAGTGTTCGAAGGTAACCGCCCGACTAACTCCATCCTGCTGCGTGAGATCACCCCGTTCAGCCTCGGGGCGCTGATTGCCCTGTACGAGCACAAAATCTTCACCCAGGGCGCGATCCTCAACATCTTCACCTTTGACCAGTGGGGCGTTGAGCTGGGCAAACAGCTGGCTAACCGCATCCTGCCGGAGCTGAAAGACGGCAGCGAAGTTAGCAGCCACGACAGCTCTACTAACGGCCTGATTAACCGCTTAGGGGGGGGGGGGGGGGGA

>phoE

GGTAAGTGCAACTTATCCTCTCTGACCCTGCAGTACCAGGGTAAAAACGAAGGCCGTGAAGCGAAGAAACAGAACGGCGACGGCGTCGGCACCTCGTTAAGCTATGATTTCGGCGGCAGCGACTTCGCCGTCAGCGCAGCCTACACCAGCTCCGACCGTACCAACGATCAGAACCTGCTGGCCCGCGGCCAGGGTTCGAAAGCGGAAGCCTGGGCGACCGGCCTGAAATATGACGCCAACAATATCTACCTGGCGACCATGTACTCTGAAACCCGCAAGATGACCCCGATCAGCGGCGGCTTTGCCAACAAAGCGCAGAACTTTGAAGCGGTGGCGCAGTATCAGTTCGACTTCGGTCTGCGTCCGTCCCTCGGCTATGTGCTGTCGAAAGGGAAGGATATCGAAGGGGTGGGGAGTGAAGATCTGGTTAACTACATCGACGTGGGCCTGACCTACTACTTCAACAAAAACATGAACGCCTTCGTGGATTACAAAATCAACCAGCTGAAAAGCGATAACAAACTCGGCATCAACGATGACGACATCGTCGCGCTGGGTATCACCTACGTTTTTTTAAAAAAAA

>infB

AAAGGAGCTCGGCGAGCGGGTGGTATTACCCAGCACATCGGTGCTTACCACGTCGAAACCGACAACGGCATGATCACCTTCCTGGATACCCCGGGCCACGCCGCGTTTACCTCCATGCGTGCTCGTGGCGCGCAGGCGACGGATATCGTGGTTCTGGTGGTGGCGGCAGACGACGGCGTGATGCCGCAGACTATCGAAGCTATCCAGCACGCTAAAGCGGCGCAGGTACCGGTGGTAGTGGCGGTGAACAAGATCGATAAGCCAGAAGCCGATCCGGATCGCGTGAAGAACGAACTGTCCCAGTACGGCATCCTGCCGGAAGAGTGGGGCGGCGAGAGCCAGTTTGTCCACGTTTCCGCGAAAGCGGGTACCGGCATCGACGACCTGCTGGACGCGATCCTGCTGCAGGCTGAAGTTCTGATGGAAAAGAGCGGACA

>tonB

GCAGGAGACGTCTCGACGCAGCCGATAGAGATCACAATGGTGGCGCCGGCCGATCTTGAGCCGCCTCCGGCGGCGCAGCCTGTCGTGGAGCCCGTTGTTGAACCCGAACCTGAGCCGGAGCCAGAGGTAGCGCCTGAACCGCCGAAAGAGGCGCCGGTGGTGATCCATAAACCGGAACCTAAGCCGAAGCCCAAACCTAAACCCAAGCCTAAGCCGGAGAAAAAGGTTGAACAGCCGAAGCGGGAAGTGAAGCCGGCAGCAGAGCCGCGTCCGGCCTCGCCGTTTGAAAACAACAATACGGCGCCGGCGCGTACAGCGCCAAGTACCTCGACCGCAGCGGCTAAACCCACCGTTACTGCTCCGAGCGGCCCGCGGGCGATCAGCCGCGTTCAGCCGTCCTATCCGCCGCGCGCTCAGGCGCTGCGCATTGAAGGGACGGTACGGGTGAAGTTTGACGTTTCGCCTGATGGCCGCATTGATAATCTGCAGATCCTCTCTGCTAGCCGGGGGCAAATA

FO32 (ST11)

>rpoB

ATATACTGCTGTCGTGTAGAGCGTGCGGTGAGAGCGTCTGTCTCTTGGCGATCTGGATACCCTGATGCCTCAGGATATGATCAACGCCAAGCCGATTTCCGCAGCAGTGAAAGAGTTCTTTGGTTCCAGCCAGCTGTCTCAGTTTATGGACCAGAACAACCCGCTGTCTGAGATTACGCACAAACGTCGTATCTCCGCACTCGGCCCAGGCGGTCTGACCCGTGAGCGCGCAGGCTTCGAAGTTCGAGACGTACACCCGACCCACTACGGTCGCGTATGTCCGATCGAAACGCCTGAAGGTCCGAACATCGGTCTGATTAACTCCCTGTCCGTGTACGCGCAGACCAACGAATATGGCTTCCTTGAGACGCCGTATCGTAAAGTGACCAACGGTGTGGTTACTGACGAAATTCACTACCTGTCTGCTATCGAAGAAGGCAACTACGTTATCGCTCAGGCGAACTCCAACCTGGATGAAAACGGCCACTTCGTAGAAGATCTGGTTACCTGCCGTAGCAAAGGCGAATCCAGCTTGTTCAGCCGCGACCAGGTTGACTACATGGACGTATCCACCCAGCAGGTGGTATCCGTCGGTGCGTCCCTGATCCCGTTCCTGGAACACGATGACGCCAACCGTGCATTGATGGGTGCGAACATGCAACGTCAGGCGGTTCCGACTCTGCGCGCTGATAAGCCGCTGGTTGGTACCGGTATGGAACGTGCTGTTGCCGTTGACTCCGGTGTTACTGCCGTGGCTAAACGTGGCGGTACCGTTCAGTACGTGGATGCTTCCCGTATCGTTATCAAAGTTAACGAAGACGAGATGTACCCGGGCGAAGCAGGTATCGACATCTATAACCTGACCAAGTACACCCGTTCTAACCAGAACACCTGCATCAACCAGATGCCTTGCGTGTCCCTGGGCGAACCTATTGAGCGCGGCGACGTGCTGGCAGACGGCCCGTCCACCGACCTCGGTGAGCTGGCGCTGGGTCAGACATGCGTGTAGCGTTCATGCCGTGGAACGGTAATCTCTCTCTAAAAACTACTAAAAAAGAA

>gapA

CGTGTTACCGCTGAACGTGACCCGGCTAACCTGAAGTGGGACGAAGTTGGTGTTGACGTTGTTGCTGAAGCAACCGGTATCTTCCTGACCGACAAAACCGCTCGTAAACACATCACCGCTGGCGCGAAAAAAGTCGTTCTGACTGGCCCGTCCAAAGACAACACTCCGATGTTCGTTCGCGGCGCTAACTTCGACGCTTACGCTGGCCAGGACATCGTTTCCAACGCTTCCTGCACCACTAACTGCCTGGCGCCGCTGGCTAAAGTTATCAACGACAACTTCGGTATCGTTGAAGGCCTGATGACCACCGTCCACGCTACCACCGCTACTCAGAAAACCGTTGATGGCCCGTCTCACAAAGACTGGCGCGGCGGCCGCGGCGCAGCTCAGAACATCATCCCGTCCTCTACCGGCGCTGCTAAAGCAGTAGGTAAAGTACTGCCAGAACTGAACGGCAAACTGACCGGTATGGCGTTCCGCGTTCCAACTCCGAACGTATCTGTTGTTGACCTGACCGTTCGTCT

>mdh

GTCATATCCCCACAGATGTAAAAATTAAAGGATTTTCCGGTGAAGACGCTACTCCGGCGCTGGAAGGCGCGGATGTAGTGCTGATCTCCGCGGGCGTGGCGCGTAAGCCCGGCATGGATCGTTCCGACCTGTTTAATGTGAATGCGGGTATCGTGAAGAACCTCGTGCAGCAGATTGCCAAAACCTGCCCGCAGGCCTGCATCGGCATTATCACCAACCCGGTGAATACCACCGTGGCTATCGCCGCCGAAGTACTGAAAAAAGCCGGCGTGTACGATAAAAACAAACTGTTCGGCGTTACCACGCTGGACATCATCCGTTCCAATACCTTTGTGGCGGAGCTGAAAGGTAAATCGGCAACCGAGGTGGAAGTCCCGGTCATTGGTGGTCACTCCGGGGTCACCATTCTGCCTTTACTGTCGCAGATCCCCGGCGTCAGCTTTAGCGATCAGGAAATTGCCGACCTGACTAAACGTATTCAGAACGCCGGTACCGAAGTCGTGGAAGCGAAAGCGGGCGGCGGGTCGGCGACCTTGTCGATGGGCCAGGCGGCTGCCCGTTTTGGTCTCTCTCTGGTTCGCGCCATGCAGGGGGAAAAAGGCGTGGTGGAGTGCGCCTACGTGGAAGGCGACGGCCACTATGCGCGTTTCTTCTCCCAGCCGCTGCT

>pgi

CGATTCTGCCGTACGACCAGTACATGCACCGCTTTGCCGCTTACTTCCAGCAGGGCAACATGGAGTCCAACGGTAAGTATGTTGACCGTAACGGCCACGCGGTAGACTACCAGACTGGCCCAATCATCTGGGGTGAGCCGGGCACCAACGGTCAGCACGCGTTCTACCAGCTGATCCACCAGGGCACCAAAATGGTACCGTGCGATTTCATCGCTCCGGCTATCACCCACAACCCGCTGTCTGACCACCATCAGAAACTGCTGTCTAACTTCTTCGCCCAGACCGAGGCCCTGGCCTTTGGTAAATCCCGCGAAGTGGTTGAGCAGGAATATCGCGATCAGGGTAAAGACCCGGCGACCCTGGAGCACGTGGTGCCGTTCAAAGTGTTCGAAGGTAACCGCCCGACTAACTCCATCCTGCTGCGTGAGATCACCCCGTTCAGCCTCGGGGCGCTGATTGCCCTGTACGAGCACAAAATCTTCACCCAGGGCGCGATCCTCAACATCTTCA

>phoE

CGTGAAGCGAAGAAACAGAACGGCGACGGCGTCGGCACCTCGTTAAGCTATGATTTCGGCGGCAGCGACTTCGCCGTCAGCGCAGCCTACACCAGCTCCGACCGTACCAACGATCAGAACCTGCTGGCCCGCGGCCAGGGTTCGAAAGCGGAAGCCTGGGCGACCGGCCTGAAATATGACGCCAACAATATCTACCTGGCGACCATGTACTCTGAAACCCGCAAGATGACCCCGATCAGCGGCGGCTTTGCCAACAAAGCGCAGAACTTTGAAGCGGTGGCGCAGTATCAGTTCGACTTCGGTCTGCGTCCGTCCCTCGGCTATGTGCTGTCGAAAGGGAAGGATATCGAAGGGGTGGGGAGTGAAGATCTGGTTAACTACATCGACGTGGGCCTGACCTACTACTTCAACAAAAACATGAACGCCTTCGTGGATTACAAAATCAACCAGCTGAAAAGCGATAACAAACTCGGCATCAACGATGACGACATCGTCGCGCTGGGTATCACC

>infB

CGGCATGATCACCTTCCTGGATACCCCGGGCCACGCCGCGTTTACCTCCATGCGTGCTCGTGGCGCGCAGGCGACGGATATCGTGGTTCTGGTGGTGGCGGCAGACGACGGCGTGATGCCGCAGACTATCGAAGCTATCCAGCACGCTAAAGCGGCGCAGGTACCGGTGGTAGTGGCGGTGAACAAGATCGATAAGCCAGAAGCCGATCCGGATCGCGTGAAGAACGAACTGTCCCAGTACGGCATCCTGCCGGAAGAGTGGGGCGGCGAGAGCCAGTTTGTCCACGTTTCCGCGAAAGCGGGTACCGGCATCGACGACCTGCTGGACGCGATCCTGCTGCAGGCTGAAGTTCT

>tonB

GATGAGCGTCTCGACGCAGCCGATAGAGATCACAATGGTGGCGCCGGCCGATCTTGAGCCGCCTCCGGCGGCGCAGCCTGTCGTGGAGCCCGTTGTTGAACCCGAACCTGAGCCGGAGCCAGAGGTAGCGCCTGAACCGCCGAAAGAGGCGCCGGTGGTGATCCATAAACCGGAACCTAAGCCGAAGCCCAAACCTAAACCCAAGCCTAAGCCGGAGAAAAAGGTTGAACAGCCGAAGCGGGAAGTGAAGCCGGCAGCAGAGCCGCGTCCGGCCTCGCCGTTTGAAAACAACAATACGGCGCCGGCGCGTACAGCGCCAAGTACCTCGACCGCAGCGGCTAAACCCACCGTTACTGCTCCGAGCGGCCCGCGGGCGATCAGCCGCGTTCAGCCGTCCTATCCGCCGCGCGCTCAGGCGCTGCGCATTGAAGGGACGGTACGGGTGAAGTTTGACGTTTCGCCTGATGGCCGCATTGATAATCTGCAGATCCTCTCTGTAGCCGGGAGGAAAAAA

FO33 (ST378)

>rpoB

CTACTATGCTGTCGTGTAGAGCGTGCGGTGATGAGCGTCTGTCTCTTGGCGATCTGGATACCCTGATGCCTCAGGATATGATCAACGCCAAGCCGATTTCCGCAGCAGTGAAAGAGTTCTTTGGTTCCAGCCAGCTGTCTCAGTTTATGGACCAGAACAACCCGCTGTCTGAGATTACGCACAAACGTCGTATCTCCGCACTCGGCCCAGGCGGTCTGACCCGTGAGCGCGCAGGCTTCGAAGTTCGAGACGTACACCCGACCCACTACGGTCGCGTATGTCCGATCGAAACGCCTGAAGGTCCGAACATCGGTCTGATTAACTCCCTGTCCGTGTACGCGCAGACCAACGAATATGGCTTCCTTGAGACGCCGTATCGTAAAGTGACCGACGGTGTGGTTACTGACGAAATTCACTACCTGTCTGCTATCGAAGAAGGCAACTACGTTATCGCTCAGGCGAACTCCAACCTGGATGAAAACGGCCACTTCGTAGAAGATCTGGTTACCTGCCGTAGCAAAGGCGAATCCAGCTTGTTCAGCCGCGACCAGGTTGACTACATGGACGTATCCACCCAGCAGGTGGTATCCGTCGGTGCGTCCCTGATCCCGTTCCTGGAACACGATGACGCCAACCGTGCATTGATGGGTGCGAACATGCAACGTCAGGCGGTTCCGACTCTGCGCGCTGATAAGCCGCTGGTTGGTACCGGTATGGAACGTGCTGTTGCCGTTGACTCCGGTGTTACTGCCGTGGCTAAACGTGGCGGTACCGTTCAGTACGTGGATGCTTCCCGTATCGTTATCAAAGTTAACGAAGACGAGATGTACCCGGGCGAAGCAGTATCGACATCTATAACCTGACCAAGTACACCCGTTCTAACCAGAACACCTGCATCAACCAGATGCCTTGCGTGTCCCTGGGCGAACCTATTGAGCGCGGCGACGTGCTGGCAGACGGCCCGTCCACCGACCTCGGTGAGCTGGCGCTGGGTCAGAACATGCGTGTAGCGTTCATGCCGTGACGTCTCACCAAAAAAAAAAAAAAAAAGATGAAAG

>gapA

GCGATCAGTCGTTGAGTGAGACGGTCATCTGGTCGTTAACGGTAAAAAAATCCGTGTTACCGCTGAACGTGACCCGGCTAACCTGAAGTGGGACGAAGTTGGTGTTGACGTTGTTGCTGAAGCAACCGGTATCTTCCTGACCGACGAAACCGCTCGTAAACACATCACCGCTGGCGCGAAAAAAGTCGTTCTGACTGGCCCGTCCAAAGACAACACTCCGATGTTCGTTCGCGGCGCTAACTTCGACGCTTACGCTGGCCAGGACATCGTTTCCAACGCTTCCTGCACCACCAACTGCCTGGCGCCGCTGGCTAAAGTTATCAACGACAACTTCGGTATCGTTGAAGGCCTGATGACCACCGTCCACGCTACCACCGCTACTCAGAAAACCGTTGATGGCCCGTCTCACAAAGACTGGCGCGGCGGCCGCGGCGCAGCTCAGAACATCATCCCGTCCTCTACCGGCGCTGCTAAAGCAGTAGGTAAAGTACTGCCAGAACTGAACGGCAAACTGACCGGTATGGCGTTCCGCGTTCCGACTCCGAACGTATCTGTTGTTGACCTGACCGTTCGTCTGGAAAAAGCAGCGTCCTACGAAGAAATCAAGAAAGCCATCAAGGCTCTTTTTTATAGAAGAGA

>mdh

GCATTAGACTCGCTCGGTTACGCCGGGCGTGGCGGTAGATCTAAGTCATATCCCCACAGATGTAAAAATTAAAGGATTTTCCGGTGAAGACGCTACTCCGGCGCTGGAAGGCGCGGATGTAGTGCTGATCTCCGCGGGCGTGGCGCGTAAGCCCGGCATGGATCGTTCCGACCTGTTTAATGTGAATGCGGGTATCGTGAAGAACCTCGTGCAGCAGATTGCCAAAACCTGCCCGCAGGCCTGCATCGGCATTATCACCAACCCGGTGAATACCACCGTGGCTATCGCCGCCGAAGTACTGAAAAAAGCCGGCGTGTACGATAAAAACAAACTGTTCGGCGTTACCACGCTGGACATCATCCGTTCCAATACCTTTGTGGCGGAGCTGAAAGGTAAATCGGCAACCGAGGTGGAAGTCCCGGTCATTGGTGGTCACTCCGGGGTCACCATTCTGCCTTTACTGTCGCAGATCCCCGGCGTCAGCTTTAGCGATCAGGAAATTGCCGACCTGACTAAACGTATTCAGAACGCCGGTACCGAAGTCGTGGAAGCGAAAGCGGGCGGCGGGTCGGCGACCTTGTCGATGGGCCAGGCGGCTGCCCGTTTTGGTCTCTCTCTGGTTCGCGCCATGCAGGGGGAAAAAGGCGTGGTGGAGTGCGCCTACGTGGAAGGCGACGGCCACTATGCGCGTTTCTTCTCCCAGCCGCTGTCGGGAAAAAAAAACAGCGAGGA

>pgi

ATTCCATACGCTCTCTGGTCACACTTCTTCGGTGCGGAACCGAAGCGATTCTGCCGTACGACCAGTACATGCACCGCTTTGCCGCTTACTTCCAGCAGGGCAACATGGAGTCCAACGGTAAGTATGTTGACCGTAACGGCCACGCGGTAGACTACCAGACTGGCCCAATCATCTGGGGTGAGCCGGGCACCAACGGTCAGCACGCGTTCTACCAGCTGATCCACCAGGGCACCAAAATGGTACCGTGCGATTTCATCGCTCCGGCTATCACCCACAACCCGCTGTCTGACCACCATCAGAAACTGCTGTCTAACTTCTTCGCCCAGACCGAGGCCCTGGCCTTTGGTAAATCCCGCGAAGTGGTTGAGCAGGAATATCGCGATCAGGGTAAAGACCCGGCGACCCTGGAGCACGTGGTGCCGTTCAAAGTGTTCGAAGGTAACCGCCCGACTAACTCCATCCTGCTGCGTGAGATCACCCCGTTCAGCCTCGGGGCGCTGATTGCCCTGTACGAGCACAAAATCTTCACCCAGGGCGCGATCCTCAACATCTTCACCTTTGACCAGTGGGGCGTTGAGCTGGGCAAACAGCTGGCTAACCGCATCCTGCCGGAGCTGAAAGACGGCAGCGAAGTTAGCAGCCACGACAGCTCTACTAACGGCCTGATTAACCGTTAGGGGAGGGGGGGCGCGGGA

>phoE

GTAGTGTAACATCTGAGTCTGGATCTGACCTGCAGTACCAGGGTAAAAACGAAGGCCGTGAAGCGAAGAAACAGAACGGCGACGGCGTCGGCACCTCGTTAAGCTATGATTTCGGCGGCAGCGACTTCGCCGTCAGCGCAGCCTACACCAGCTCCGACCGTACCAACGATCAGAACCTGCTGGCCCGCGGCCAGGGTTCGAAAGCGGAAGCCTGGGCGACCGGCCTGAAATATGACGCCAACAATATCTACCTGGCGACCATGTACTCTGAAACCCGCAAAATGACCCCGATCAGCGGCGGCTTTGCCAACAAAGCGCAGAACTTTGAAGCGGTGGCGCAGTATCAGTTCGACTTCGGTCTGCGTCCGTCCCTCGGCTATGTGCTGTCGAAAGGGAAGGATATCGAAGGGGTGGGGAGTGAGGATCTGGTTAACTACATTGACGTGGGCCTGACCTACTACTTCAACAAAAACATGAACGCCTTCGTGGATTACAAAATCAACCAGCTGAAAAGCGATAACAAACTCGGCATCAACGATGACGACATCGTCGCGCTGGGTATCACCTACGTTTTTTTAAAAAAA

>infB

ATCTTGCTCGGCGAGCGGGTGGTATTACCCAGCACATCGGTGCTTACCACGTCGAAACCGACAACGGCATGATCACCTTCCTGGATACCCCGGGCCACGCCGCGTTTACCTCCATGCGTGCTCGTGGCGCGCAGGCGACGGATATCGTGGTTCTGGTGGTGGCGGCAGACGACGGCGTGATGCCGCAGACTATCGAAGCTATCCAGCACGCTAAAGCGGCGCAGGTACCGGTGGTAGTGGCGGTGAACAAGATCGATAAGCCAGAAGCCGATCCGGATCGCGTGAAGAACGAACTGTCCCAGTACGGCATCCTGCCGGAAGAGTGGGGCGGCGAGAGCCAGTTCGTCCACGTTTCCGCGAAAGCGGGTACCGGCATCGACGACCTGCTGGACGCGATCCTGCTGCAGGCTGAAGTTCTTGGCGGTAAAAGCGA

>tonB

AATGAGCATCTCGACGCAGCCGATAGAGATCACAATGGTGGCGCCGGCCGATCTTGAGCCGCCTCCGGCGGCGCAGCCTGTCGTGGAGCCCGTTGTTGAACCCGAACCTGAGCCGGAGCCAGAGGTAGTGCCTGAACCGCCGAAAGAGGCGCCGGTGGTGATCCATAAACCGGAACCTAAGCCGAAGCCCAAACCTAAACTCAAGCCTAAGCCGGAGAAAAAGGTTGAACAGCCGAAGCGGGAAGTGAAGCCGGCTGCAGAGCCGCGTCCGGCCTCGCCGTTTGAAAACAACAATACGGCGCCGGCGCGTACAGCGCCAAGCACCTCGACAGCAGCGGCTAAACCCACCGTTACTGCTCCAAGCGGCCCGCGGGCGATCAGCCGCGTTCAGCCGTCCTATCCGGCGCGCGCTCAGGCGCTGCGCATTGAAGGGACGGTACGGGTGAAGTTTGACGTTTCGCCTGATGGCCGCATTGATAATCTGCAGATCCTCTCTGTAGCCGGGGGGAAATAA

FO34 (ST378)

>rpoB

TAGACTGCTATCGTGTAGAGCGTGCGGTGATGAGCGTCTGTCTCTTGGCGATCTGGATACCCTGATGCCTCAGGATATGATCAACGCCAAGCCGATTTCCGCAGCAGTGAAAGAGTTCTTTGGTTCCAGCCAGCTGTCTCAGTTTATGGACCAGAACAACCCGCTGTCTGAGATTACGCACAAACGTCGTATCTCCGCACTCGGCCCAGGCGGTCTGACCCGTGAGCGCGCAGGCTTCGAAGTTCGAGACGTACACCCGACCCACTACGGTCGCGTATGTCCGATCGAAACGCCTGAAGGTCCGAACATCGGTCTGATTAACTCCCTGTCCGTGTACGCGCAGACCAACGAATATGGCTTCCTTGAGACGCCGTATCGTAAAGTGACCGACGGTGTGGTTACTGACGAAATTCACTACCTGTCTGCTATCGAAGAAGGCAACTACGTTATCGCTCAGGCGAACTCCAACCTGGATGAAAACGGCCACTTCGTAGAAGATCTGGTTACCTGCCGTAGCAAAGGCGAATCCAGCTTGTTCAGCCGCGACCAGGTTGACTACATGGACGTATCCACCCAGCAGGTGGTATCCGTCGGTGCGTCCCTGATCCCGTTCCTGGAACACGATGACGCCAACCGTGCATTGATGGGTGCGAACATGCAACGTCAGGCGGTTCCGACTCTGCGCGCTGATAAGCCGCTGGTTGGTACCGGTATGGAACGTGCTGTTGCCGTTGACTCCGGTGTTACTGCCGTGGCTAAACGTGGCGGTACCGTTCAGTACGTGGATGCTTCCCGTATCGTTATCAAAGTTAACGAAGACGAGATGTACCCGGGCGAAGCAGTATCGACATCTATAACCTGACCAAGTACACCCGTTCTAACCAGAACACCTGCATCAACCAGATGCCTTGCGTGTCCCTGGGCGAACCTATTGAGCGCGGCGACGTGCTGGCAGACGCCCGTCCACCGACCTCGGTGAGCTGGCGCTGGGTCAGACATGCGTGTAGCGTCATGCGTGACGCTCTTTTTTATAAAAAATAAACAAAAAAAAGTACGG

>gapA

GGGGGTCCAGTCGTTGAGTGAGACGGTCATCTGGTCGTTAACGGTAAAAAAATCCGTGTTACCGCTGAACGTGACCCGGCTAACCTGAAGTGGGACGAAGTTGGTGTTGACGTTGTTGCTGAAGCAACCGGTATCTTCCTGACCGACGAAACCGCTCGTAAACACATCACCGCTGGCGCGAAAAAAGTCGTTCTGACTGGCCCGTCCAAAGACAACACTCCGATGTTCGTTCGCGGCGCTAACTTCGACGCTTACGCTGGCCAGGACATCGTTTCCAACGCTTCCTGCACCACCAACTGCCTGGCGCCGCTGGCTAAAGTTATCAACGACAACTTCGGTATCGTTGAAGGCCTGATGACCACCGTCCACGCTACCACCGCTACTCAGAAAACCGTTGATGGCCCGTCTCACAAAGACTGGCGCGGCGGCCGCGGCGCAGCTCAGAACATCATCCCGTCCTCTACCGGCGCTGCTAAAGCAGTAGGTAAAGTACTGCCAGAACTGAACGGCAAACTGACCGGTATGGCGTTCCGCGTTCCGACTCCGAACGTATCTGTTGTTGACCTGACCGTTCGTCTGGAAAAAGCAGCGTCCTACGAAGAAATCAAGAAAGCCATCAAAGCCCTTTTTCTTGAAG

>mdh

ATACCGATCACTCGCTCGGTTACGCCGGGCGTGGCGGTAGATCTAAGTCATATCCCCACAGATGTAAAAATTAAAGGATTTTCCGGTGAAGACGCTACTCCGGCGCTGGAAGGCGCGGATGTAGTGCTGATCTCCGCGGGCGTGGCGCGTAAGCCCGGCATGGATCGTTCCGACCTGTTTAATGTGAATGCGGGTATCGTGAAGAACCTCGTGCAGCAGATTGCCAAAACCTGCCCGCAGGCCTGCATCGGCATTATCACCAACCCGGTGAATACCACCGTGGCTATCGCCGCCGAAGTACTGAAAAAAGCCGGCGTGTACGATAAAAACAAACTGTTCGGCGTTACCACGCTGGACATCATCCGTTCCAATACCTTTGTGGCGGAGCTGAAAGGTAAATCGGCAACCGAGGTGGAAGTCCCGGTCATTGGTGGTCACTCCGGGGTCACCATTCTGCCTTTACTGTCGCAGATCCCCGGCGTCAGCTTTAGCGATCAGGAAATTGCCGACCTGACTAAACGTATTCAGAACGCCGGTACCGAAGTCGTGGAAGCGAAAGCGGGCGGCGGGTCGGCGACCTTGTCGATGGGCCAGGCGGCTGCCCGTTTTGGTCTCTCTCTGGTTCGCGCCATGCAGGGGGAAAAAGGCGTGGTGGAGTGCGCCTACGTGGAAGGCGACGGCCACTATGCGCGTTTCTTCTCCCAGCCGCTGCTGCTGGGAAAAAACGGA

>pgi

CTGGGGTCACAACTTCTTCGGTGCGGAACCGAAGCGATTCTGCCGTACGACCAGTACATGCACCGCTTTGCCGCTTACTTCCAGCAGGGCAACATGGAGTCCAACGGTAAGTATGTTGACCGTAACGGCCACGCGGTAGACTACCAGACTGGCCCAATCATCTGGGGTGAGCCGGGCACCAACGGTCAGCACGCGTTCTACCAGCTGATCCACCAGGGCACCAAAATGGTACCGTGCGATTTCATCGCTCCGGCTATCACCCACAACCCGCTGTCTGACCACCATCAGAAACTGCTGTCTAACTTCTTCGCCCAGACCGAGGCCCTGGCCTTTGGTAAATCCCGCGAAGTGGTTGAGCAGGAATATCGCGATCAGGGTAAAGACCCGGCGACCCTGGAGCACGTGGTGCCGTTCAAAGTGTTCGAAGGTAACCGCCCGACTAACTCCATCCTGCTGCGTGAGATCACCCCGTTCAGCCTCGGGGCGCTGATTGCCCTGTACGAGCACAAAATCTTCACCCAGGGCGCGATCCTCAACATCTTCACCTTTGACCAGTGGGGCGTTGAGCTGGGCAAACAGCTGGCTAACCGCATCCTGCCGGAGCTGAAAGACGGCAGCGAAGTTAGCAGCCACGACAGCTCTACTAACGGCCTGATTAACCGCTATAAAGGGTGGGCGCGA

>phoE

CTAGGCTGCTTGCTGACTCTGACCTGCAGTACCAGGGTAAAACGAAGGCCGTGAAGCGAAGAAACAGAACGGCGACGGCGTCGGCACCTCGTTAAGCTATGATTTCGGCGGCAGCGACTTCGCCGTCAGCGCAGCCTACACCAGCTCCGACCGTACCAACGATCAGAACCTGCTGGCCCGCGGCCAGGGTTCGAAAGCGGAAGCCTGGGCGACCGGCCTGAAATATGACGCCAACAATATCTACCTGGCGACCATGTACTCTGAAACCCGCAAAATGACCCCGATCAGCGGCGGCTTTGCCAACAAAGCGCAGAACTTTGAAGCGGTGGCGCAGTATCAGTTCGACTTCGGTCTGCGTCCGTCCCTCGGCTATGTGCTGTCGAAAGGGAAGGATATCGAAGGGGTGGGGAGTGAGGATCTGGTTAACTACATTGACGTGGGCCTGACCTACTACTTCAACAAAAACATGAACGCCTTCGTGGATTACAAAATCAACCAGCTGAAAAGCGATAACAAACTCGGCATCAACGATGACGACATCGTCGCGCTGGGTATCACCTACCAGTTTCCTGGATCAA

>infB

TAGTGCTCGGCGAGCGGGTGGTATTACCCAGCACATCGGTGCTTACCACGTCGAAACCGACAACGGCATGATCACCTTCCTGGATACCCCGGGCCACGCCGCGTTTACCTCCATGCGTGCTCGTGGCGCGCAGGCGACGGATATCGTGGTTCTGGTGGTGGCGGCAGACGACGGCGTGATGCCGCAGACTATCGAAGCTATCCAGCACGCTAAAGCGGCGCAGGTACCGGTGGTAGTGGCGGTGAACAAGATCGATAAGCCAGAAGCCGATCCGGATCGCGTGAAGAACGAACTGTCCCAGTACGGCATCCTGCCGGAAGAGTGGGGCGGCGAGAGCCAGTTCGTCCACGTTTCCGCGAAAGCGGGTACCGGCATCGACGACCTGCTGGACGCGATCCTGCTGCAGGCTGAAGTTCTTGGCTTTGAAAGCGA

>tonB

GGGTACTCATCTCGACGCAGCCGATAGAGATCACAATGGTGGCGCCGGCCGATCTTGAGCCGCCTCCGGCGGCGCAGCCTGTCGTGGAGCCCGTTGTTGAACCCGAACCTGAGCCGGAGCCAGAGGTAGTGCCTGAACCGCCGAAAGAGGCGCCGGTGGTGATCCATAAACCGGAACCTAAGCCGAAGCCCAAACCTAAACTCAAGCCTAAGCCGGAGAAAAAGGTTGAACAGCCGAAGCGGGAAGTGAAGCCGGCTGCAGAGCCGCGTCCGGCCTCGCCGTTTGAAAACAACAATACGGCGCCGGCGCGTACAGCGCCAAGCACCTCGACAGCAGCGGCTAAACCCACCGTTACTGCTCCAAGCGGCCCGCGGGCGATCAGCCGCGTTCAGCCGTCCTATCCGGCGCGCGCTCAGGCGCTGCGCATTGAAGGGACGGTACGGGTGAAGTTTGACGTTTCGCCTGATGGCCGCATTGATAATCTGCAGATCCTCTCTGCTCAGCCGGGGCGAAT

FO35 (ST378)

>rpoB

AAAACTCGCTGTCGTGTAGAGCGTGCGGTGATGAGCGTCTGTCTCTTGGCGATCTGGATACCCTGATGCCTCAGGATATGATCAACGCCAAGCCGATTTCCGCAGCAGTGAAAGAGTTCTTTGGTTCCAGCCAGCTGTCTCAGTTTATGGACCAGAACAACCCGCTGTCTGAGATTACGCACAAACGTCGTATCTCCGCACTCGGCCCAGGCGGTCTGACCCGTGAGCGCGCAGGCTTCGAAGTTCGAGACGTACACCCGACCCACTACGGTCGCGTATGTCCGATCGAAACGCCTGAAGGTCCGAACATCGGTCTGATTAACTCCCTGTCCGTGTACGCGCAGACCAACGAATATGGCTTCCTTGAGACGCCGTATCGTAAAGTGACCAACGGTGTGGTTACTGACGAAATTCACTACCTGTCTGCTATCGAAGAAGGCAACTACGTTATCGCTCAGGCGAACTCCAACCTGGATGAAAACGGCCACTTCGTAGAAGATCTGGTTACCTGCCGTAGCAAAGGCGAATCCAGCTTGTTCAGCCGCGACCAGGTTGACTACATGGACGTATCCACCCAGCAGGTGGTATCCGTCGGTGCGTCCCTGATCCCGTTCCTGGAACACGATGACGCCAACCGTGCATTGATGGGTGCGAACATGCAACGTCAGGCGGTTCCGACTCTGCGCGCTGATAAGCCGCTGGTTGGTACCGGTATGGAACGTGCTGTTGCCGTTGACTCCGGTGTTACTGCCGTGGCTAAACGTGGCGGTACCGTTCAGTACGTGGATGCTTCCCGTATCGTTATCAAAGTTAACGAAGACGAGATGTACCCGGGCGAAGCAGTATCGACATCTATAACCTGACCAAGTACACCCGTTCTAACCAGAACACCTGCATCAACCAGATGCCTTGCGTGTCCCTGGGCGAACCTATTGAGCGCGGCGACGTGCTGGCAGACGCCCGTCCACCGACCTCGGTGAGCTGGCGCTGGGTCAGACATGCGTGTAGCGTTCATGCCGTGGACGTACTTCTTCTTCAAGACACTACCA

>gapA

CATCTGGTCGTTAACGGTAAAAAAATCCGTGTTACCGCTGAACGTGACCCGGCTAACCTGAAGTGGGACGAAGTTGGTGTTGACGTTGTTGCTGAAGCAACCGGTATCTTCCTGACCGACGAAACCGCTCGTAAACACATCACCGCTGGCGCGAAAAAAGTCGTTCTGACTGGCCCGTCCAAAGACAACACTCCGATGTTCGTTCGCGGCGCTAACTTCGACGCTTACGCTGGCCAGGACATCGTTTCCAACGCTTCCTGCACCACTAACTGCCTGGCGCCGCTGGCTAAAGTTATCAACGACAACTTCGGTATCGTTGAAGGCCTGATGACCACCGTCCACGCTACCACCGCTACTCAGAAAACCGTTGATGGCCCGTCTCACAAAGACTGGCGCGGCGGCCGCGGCGCAGCTCAGAACATCATCCCGTCCTCTACCGGCGCTGCTAAAGCAGTAGGTAAAGTACTGCCAGAACTGAACGGCAAACTGACCGGTATGGCGTTCCGCGTTCCAACTCCGAACGTATCTGTTGTTGACCTGACCGTTCGTCTGGAAAAAGCAGCGTCCTA

>mdh

CTAAGTCATATCCCCACAGATGTAAAAATTAAAGGATTTTCCGGTGAAGACGCTACTCCGGCGCTGGAAGGCGCGGATGTAGTGCTGATCTCCGCGGGCGTGGCGCGTAAGCCCGGCATGGATCGTTCCGACCTGTTTAATGTGAATGCGGGTATCGTGAAGAACCTCGTGCAGCAGATTGCCAAAACCTGCCCGCAGGCCTGCATCGGCATTATCACCAACCCGGTGAATACCACCGTGGCTATCGCCGCCGAAGTACTGAAAAAAGCCGGCGTGTACGATAAAAACAAACTGTTCGGCGTTACCACGCTGGACATCATCCGTTCCAATACCTTTGTGGCGGAGCTGAAAGGTAAATCGGCAACCGAGGTGGAAGTCCCGGTCATTGGTGGTCACTCCGGGGTCACCATTCTGCCTTTACTGTCGCAGATCCCCGGCGTCAGCTTTAGCGATCAGGAAATTGCCGACCTGACTAAACGTATTCAGAACGCCGGTACCGAAGTCGTGGAAGCGAAAGCGGGCGGCGGGTCGGCGACCTTGTCGATGGGCCAGGCGGCTGCCCGTTTTGGTCTCTCTCTGGTTCGCGCCATGCAGGGGGAAAAAGGCGTGGTGGAGTGCGCCTACGTGGAAGGCGACGGCCACTATGCGCGTTTCTTCTCCCAGCCGCTGCTGCTG

>pgi

CGATTCTGCCGTACGACCAGTACATGCACCGCTTTGCCGCTTACTTCCAGCAGGGCAACATGGAGTCCAACGGTAAGTATGTTGACCGTAACGGCCACGCGGTAGACTACCAGACTGGCCCAATCATCTGGGGTGAGCCGGGCACCAACGGTCAGCACGCGTTCTACCAGCTGATCCACCAGGGCACCAAAATGGTACCGTGCGATTTCATCGCTCCGGCTATCACCCACAACCCGCTGTCTGACCACCATCAGAAACTGCTGTCTAACTTCTTCGCCCAGACCGAGGCCCTGGCCTTTGGTAAATCCCGCGAAGTGGTTGAGCAGGAATATCGCGATCAGGGTAAAGACCCGGCGACCCTGGAGCACGTGGTGCCGTTCAAAGTGTTCGAAGGTAACCGCCCGACTAACTCCATCCTGCTGCGTGAGATCACCCCGTTCAGCCTCGGGGCGCTGATTGCCCTGTACGAGCACAAAATCTTCACCCAGGGCGCGATCCTCAACATCTTCACCTTTGACCAGTGGGGCGTTGAGCTGGGCAAACAGCTGGCTAACCGCATCCTGCCGGAGCTGAAAGACGGCAGCGAAGTTAGCAGCCACGACAGCTCTACTAACGGCCTGATTAACCGCTAT

>phoE

GGCGACGGCGTCGGCACCTCGTTAAGCTATGATTTCGGCGGCAGCGACTTCGCCGTCAGCGCAGCCTACACCAGCTCCGACCGTACCAACGATCAGAACCTGCTGGCCCGCGGCCAGGGTTCGAAAGCGGAAGCCTGGGCGACCGGCCTGAAATATGACGCCAACAATATCTACCTGGCGACCATGTACTCTGAAACCCGCAAGATGACCCCGATCAGCGGCGGCTTTGCCAACAAAGCGCAGAACTTTGAAGCGGTGGCGCAGTATCAGTTCGACTTCGGTCTGCGTCCGTCCCTCGGCTATGTGCTGTCGAAAGGGAAGGATATCGAAGGGGTGGGGAGTGAAGATCTGGTTAACTACATCGACGTGGGCCTGACCTACTACTTCAACAAAAACATGAACGCCTTCGTGGATTACAAAATCAACCAGCTGAAAAGCGATAACAAACTCGGCATCAACGATGACGACATCGTCGCGCTGGGTATCACCTACCA

>infB

CACATCGGTGCTTACCACGTCGAAACCGACAACGGCATGATCACCTTCCTGGATACCCCGGGCCACGCCGCGTTTACCTCCATGCGTGCTCGTGGCGCGCAGGCGACGGATATCGTGGTTCTGGTGGTGGCGGCAGACGACGGCGTGATGCCGCAGACTATCGAAGCTATCCAGCACGCTAAAGCGGCGCAGGTACCGGTGGTAGTGGCGGTGAACAAGATCGATAAGCCAGAAGCCGATCCGGATCGCGTGAAGAACGAACTGTCCCAGTACGGCATCCTGCCGGAAGAGTGGGGCGGCGAGAGCCAGTTTGTCCACGTTTCCGCGAAAGCGGGTACCGGCATCGACGACCTGCTGGACGCGATCCTGCTGCAGGCTGAAGTTCTT

>tonB

AGATCCCCATTCTCGGACGCAGCCGATAGAGATCACAATGGTGGCGCCGGCCGATCTTGAGCCGCCTCCGGCGGCGCAGCCTGTCGTGGAGCCCGTTGTTGAACCCGAACCTGAGCCGGAGCCAGAGGTAGCGCCTGAACCGCCGAAAGAGGCGCCGGTGGTGATCCATAAACCGGAACCTAAGCCGAAGCCCAAACCTAAACCCAAGCCTAAGCCGGAGAAAAAGGTTGAACAGCCGAAGCGGGAAGTGAAGCCGGCAGCAGAGCCGCGTCCGGCCTCGCCGTTTGAAAACAACAATACGGCGCCGGCGCGTACAGCGCCAAGTACCTCGACCGCAGCGGCTAAACCCACCGTTACTGCTCCGAGCGGCCCGCGGGCGATCAGCCGCGTTCAGCCGTCCTATCCGCCGCGCGCTCAGGCGCTGCGCATTGAAGGGACGGTACGGGTGAAGTTTGACGTTTCGCCTGATGGCCGCATTGATAATCTGCAGATCCTCTCTGCTCAGCCCGGCGAATAA

FO36 (ST11)

>rpoB

TAACTTGCTGTCGTGTAGAGCGTGCGGTGAGAGCGTCTGTCTCTTGGCGATCTGGATACCCTGATGCCTCAGGATATGATCAACGCCAAGCCGATTTCCGCAGCAGTGAAAGAGTTCTTTGGTTCCAGCCAGCTGTCTCAGTTTATGGACCAGAACAACCCGCTGTCTGAGATTACGCACAAACGTCGTATCTCCGCACTCGGCCCAGGCGGTCTGACCCGTGAGCGCGCAGGCTTCGAAGTTCGAGACGTACACCCGACCCACTACGGTCGCGTATGTCCGATCGAAACGCCTGAAGGTCCGAACATCGGTCTGATTAACTCCCTGTCCGTGTACGCGCAGACCAACGAATATGGCTTCCTTGAGACGCCGTATCGTAAAGTGACCAACGGTGTGGTTACTGACGAAATTCACTACCTGTCTGCTATCGAAGAAGGCAACTACGTTATCGCTCAGGCGAACTCCAACCTGGATGAAAACGGCCACTTCGTAGAAGATCTGGTTACCTGCCGTAGCAAAGGCGAATCCAGCTTGTTCAGCCGCGACCAGGTTGACTACATGGACGTATCCACCCAGCAGGTGGTATCCGTCGGTGCGTCCCTGATCCCGTTCCTGGAACACGATGACGCCAACCGTGCATTGATGGGTGCGAACATGCAACGTCAGGCGGTTCCGACTCTGCGCGCTGATAAGCCGCTGGTTGGTACCGGTATGGAACGTGCTGTTGCCGTTGACTCCGGTGTTACTGCCGTGGCTAAACGTGGCGGTACCGTTCAGTACGTGGATGCTTCCCGTATCGTTATCAAAGTTAACGAAGACGAGATGTACCCGGGCGAAGCAGTATCGACATCTATAACCTGACCAAGTACACCCGTTCTAACCAGAACACCTGCATCAACCAGATGCCTTGCGTGTCCCTGGGCGAACCTATTGAGCGCGGCGACGTGCTGGCAGACGGCCCGTCCACCGACCTCGGTGAGCTGGCGCTGGGTCAGAACATGCGTGTAGCGTTCATGCGTGACGTCTCTTTTTTAAAAAAAAAAAAAAAATTAAATG

>gapA

CGATCCGTCGTTGAGTGAAGACGGTCATCTGGTCGTTAACGGTAAAAAAATCCGTGTTACCGCTGAACGTGACCCGGCTAACCTGAAGTGGGACGAAGTTGGTGTTGACGTTGTTGCTGAAGCAACCGGTATCTTCCTGACCGACGAAACCGCTCGTAAACACATCACCGCTGGCGCGAAAAAAGTCGTTCTGACTGGCCCGTCCAAAGACAACACTCCGATGTTCGTTCGCGGCGCTAACTTCGACGCTTACGCTGGCCAGGACATCGTTTCCAACGCTTCCTGCACCACTAACTGCCTGGCGCCGCTGGCTAAAGTTATCAACGACAACTTCGGTATCGTTGAAGGCCTGATGACCACCGTCCACGCTACCACCGCTACTCAGAAAACCGTTGATGGCCCGTCTCACAAAGACTGGCGCGGCGGCCGCGGCGCAGCTCAGAACATCATCCCGTCCTCTACCGGCGCTGCTAAAGCAGTAGGTAAAGTACTGCCAGAACTGAACGGCAAACTGACCGGTATGGCGTTCCGCGTTCCAACTCCGAACGTATCTGTTGTTGACCTGACCGTTCGTCTGGAAAAAGCAGCGTCCTACGAAGAAATCAAGAAAGCCATCAAGGTTTTTCTTTTGAAGAAGAG

>mdh

AGCATTTGACTCGCTCGGTTACGCCGGGCGTGGCGGTAGATCTAAGTCATATCCCCACAGATGTAAAAATTAAAGGATTTTCCGGTGAAGACGCTACTCCGGCGCTGGAAGGCGCGGATGTAGTGCTGATCTCCGCGGGCGTGGCGCGTAAGCCCGGCATGGATCGTTCCGACCTGTTTAATGTGAATGCGGGTATCGTGAAGAACCTCGTGCAGCAGATTGCCAAAACCTGCCCGCAGGCCTGCATCGGCATTATCACCAACCCGGTGAATACCACCGTGGCTATCGCCGCCGAAGTACTGAAAAAAGCCGGCGTGTACGATAAAAACAAACTGTTCGGCGTTACCACGCTGGACATCATCCGTTCCAATACCTTTGTGGCGGAGCTGAAAGGTAAATCGGCAACCGAGGTGGAAGTCCCGGTCATTGGTGGTCACTCCGGGGTCACCATTCTGCCTTTACTGTCGCAGATCCCCGGCGTCAGCTTTAGCGATCAGGAAATTGCCGACCTGACTAAACGTATTCAGAACGCCGGTACCGAAGTCGTGGAAGCGAAAGCGGGCGGCGGGTCGGCGACCTTGTCGATGGGCCAGGCGGCTGCCCGTTTTGGTCTCTCTCTGGTTCGCGCCATGCAGGGGGAAAAAGGCGTGGTGGAGTGCGCCTACGTGGAAGGCGACGGCCACTATGCGCGTTTCTTCTCCCAGCCGCTGTCGGGAGAAAAAAAAGAGAGAAAA

>pgi

TTATATCGGTCGTTCTCTGGTCACACTTCTTCGGTGCGGAACCGAAGCGATTCTGCCGTACGACCAGTACATGCACCGCTTTGCCGCTTACTTCCAGCAGGGCAACATGGAGTCCAACGGTAAGTATGTTGACCGTAACGGCCACGCGGTAGACTACCAGACTGGCCCAATCATCTGGGGTGAGCCGGGCACCAACGGTCAGCACGCGTTCTACCAGCTGATCCACCAGGGCACCAAAATGGTACCGTGCGATTTCATCGCTCCGGCTATCACCCACAACCCGCTGTCTGACCACCATCAGAAACTGCTGTCTAACTTCTTCGCCCAGACCGAGGCCCTGGCCTTTGGTAAATCCCGCGAAGTGGTTGAGCAGGAATATCGCGATCAGGGTAAAGACCCGGCGACCCTGGAGCACGTGGTGCCGTTCAAAGTGTTCGAAGGTAACCGCCCGACTAACTCCATCCTGCTGCGTGAGATCACCCCGTTCAGCCTCGGGGCGCTGATTGCCCTGTACGAGCACAAAATCTTCACCCAGGGCGCGATCCTCAACATCTTCACCTTTGACCAGTGGGGCGTTGAGCTGGGCAAACAGCTGGCTAACCGCATCCTGCCGGAGCTGAAAGACGGCAGCGAAGTTAGCAGCCACGACAGCTCTACTAACGGCCTGATTAACCGTTAGGGGGGGGGGGGGGGGGGA

>phoE

GCTACGAGTAGCTGAGAGGTATGATCTCTGACCTGCAGTACCAGGGTAAAAACGAAGGCCGTGAAGCGAAGAAACAGAACGGCGACGGCGTCGGCACCTCGTTAAGCTATGATTTCGGCGGCAGCGACTTCGCCGTCAGCGCAGCCTACACCAGCTCCGACCGTACCAACGATCAGAACCTGCTGGCCCGCGGCCAGGGTTCGAAAGCGGAAGCCTGGGCGACCGGCCTGAAATATGACGCCAACAATATCTACCTGGCGACCATGTACTCTGAAACCCGCAAGATGACCCCGATCAGCGGCGGCTTTGCCAACAAAGCGCAGAACTTTGAAGCGGTGGCGCAGTATCAGTTCGACTTCGGTCTGCGTCCGTCCCTCGGCTATGTGCTGTCGAAAGGGAAGGATATCGAAGGGGTGGGGAGTGAAGATCTGGTTAACTACATCGACGTGGGCCTGACCTACTACTTCAACAAAAACATGAACGCCTTCGTGGATTACAAAATCAACCAGCTGAAAAGCGATAACAAACTCGGCATCAACGATGACGACATCGTCGCGCTGGGTATCACCTACCGTTTTTTGATAACAA

>infB

ATAATGATGCTCGGCGAGCGGGTGGTATTACCCAGCACATCGGTGCTTACCACGTCGAAACCGACAACGGCATGATCACCTTCCTGGATACCCCGGGCCACGCCGCGTTTACCTCCATGCGTGCTCGTGGCGCGCAGGCGACGGATATCGTGGTTCTGGTGGTGGCGGCAGACGACGGCGTGATGCCGCAGACTATCGAAGCTATCCAGCACGCTAAAGCGGCGCAGGTACCGGTGGTAGTGGCGGTGAACAAGATCGATAAGCCAGAAGCCGATCCGGATCGCGTGAAGAACGAACTGTCCCAGTACGGCATCCTGCCGGAAGAGTGGGGCGGCGAGAGCCAGTTTGTCCACGTTTCCGCGAAAGCGGGTACCGGCATCGACGACCTGCTGGACGCGATCCTGCTGCAGGCTGAAGTTCTTGCCTGGAAAAAGCGGA

>tonB

AATGAGCGTCTCGACGCAGCCGATAGAGATCACAATGGTGGCGCCGGCCGATCTTGAGCCGCCTCCGGCGGCGCAGCCTGTCGTGGAGCCCGTTGTTGAACCCGAACCTGAGCCGGAGCCAGAGGTAGCGCCTGAACCGCCGAAAGAGGCGCCGGTGGTGATCCATAAACCGGAACCTAAGCCGAAGCCCAAACCTAAACCCAAGCCTAAGCCGGAGAAAAAGGTTGAACAGCCGAAGCGGGAAGTGAAGCCGGCAGCAGAGCCGCGTCCGGCCTCGCCGTTTGAAAACAACAATACGGCGCCGGCGCGTACAGCGCCAAGTACCTCGACCGCAGCGGCTAAACCCACCGTTACTGCTCCGAGCGGCCCGCGGGCGATCAGCCGCGTTCAGCCGTCCTATCCGCCGCGCGCTCAGGCGCTGCGCATTGAAGGGACGGTACGGGTGAAGTTTGACGTTTCGCCTGATGGCCGCATTGATAATCTGCAGATCCTCTCTGCCCCCCGGGGGAAAAAAA

FO37 (ST11)

>rpoB

AAACTAGCTGTCGTGTAGAGCGTGCGGTGATGAGCGTCTGTCTCTTGGCGATCTGGATACCCTGATGCCTCAGGATATGATCAACGCCAAGCCGATTTCCGCAGCAGTGAAAGAGTTCTTTGGTTCCAGCCAGCTGTCTCAGTTTATGGACCAGAACAACCCGCTGTCTGAGATTACGCACAAACGTCGTATCTCCGCACTCGGCCCAGGCGGTCTGACCCGTGAGCGCGCAGGCTTCGAAGTTCGAGACGTACACCCGACCCACTACGGTCGCGTATGTCCGATCGAAACGCCTGAAGGTCCGAACATCGGTCTGATTAACTCCCTGTCCGTGTACGCGCAGACCAACGAATATGGCTTCCTTGAGACGCCGTATCGTAAAGTGACCAACGGTGTGGTTACTGACGAAATTCACTACCTGTCTGCTATCGAAGAAGGCAACTACGTTATCGCTCAGGCGAACTCCAACCTGGATGAAAACGGCCACTTCGTAGAAGATCTGGTTACCTGCCGTAGCAAAGGCGAATCCAGCTTGTTCAGCCGCGACCAGGTTGACTACATGGACGTATCCACCCAGCAGGTGGTATCCGTCGGTGCGTCCCTGATCCCGTTCCTGGAACACGATGACGCCAACCGTGCATTGATGGGTGCGAACATGCAACGTCAGGCGGTTCCGACTCTGCGCGCTGATAAGCCGCTGGTTGGTACCGGTATGGAACGTGCTGTTGCCGTTGACTCCGGTGTTACTGCCGTGGCTAAACGTGGCGGTACCGTTCAGTACGTGGATGCTTCCCGTATCGTTATCAAAGTTAACGAAGACGAGATGTACCCGGGCGAAGCAGTATCGACATCTATAACCTGACCAAGTACACCCGTTCTAACCAGAACACCTGCATCAACCAGATGCCTTGCGTGTCCCTGGGCGAACCTATTGAGCGCGGCGACGTGCTGGCAGACGCCCGTCCACCGACTCGGTGAGCTGGCGCTGGGTCAGACATGCGTGTAGCGTTCATGCCGTGACGTCTCCTTCTTAAAACAACACATCACACAA

>gapA

TCCGTGTTACCGCTGAACGTGACCCGGCTAACCTGAAGTGGGACGAAGTTGGTGTTGACGTTGTTGCTGAAGCAACCGGTATCTTCCTGACCGACGAAACCGCTCGTAAACACATCACCGCTGGCGCGAAAAAAGTCGTTCTGACTGGCCCGTCCAAAGACAACACTCCGATGTTCGTTCGCGGCGCTAACTTCGACGCTTACGCTGGCCAGGACATCGTTTCCAACGCTTCCTGCACCACTAACTGCCTGGCGCCGCTGGCTAAAGTTATCAACGACAACTTCGGTATCGTTGAAGGCCTGATGACCACCGTCCACGCTACCACCGCTACTCAGAAAACCGTTGATGGCCCGTCTCACAAAGACTGGCGCGGCGGCCGCGGCGCAGCTCAGAACATCATCCCGTCCTCTACCGGCGCTGCTAAAGCAGTAGGTAAAGTACTGCCAGAACTGAACGGCAAACTGACCGGTATGGCGTTCCGCGTTCCAACTCCGAACGTATCTGTTGTTGACCTGACCGTTCGTCTGGAAAAAGCAGCGTCCTACG

>mdh

TTTCCGGTGAAGACGCTACTCCGGCGCTGGAAGGCGCGGATGTAGTGCTGATCTCCGCGGGCGTGGCGCGTAAGCCCGGCATGGATCGTTCCGACCTGTTTAATGTGAATGCGGGTATCGTGAAGAACCTCGTGCAGCAGATTGCCAAAACCTGCCCGCAGGCCTGCATCGGCATTATCACCAACCCGGTGAATACCACCGTGGCTATCGCCGCCGAAGTACTGAAAAAAGCCGGCGTGTACGATAAAAACAAACTGTTCGGCGTTACCACGCTGGACATCATCCGTTCCAATACCTTTGTGGCGGAGCTGAAAGGTAAATCGGCAACCGAGGTGGAAGTCCCGGTCATTGGTGGTCACTCCGGGGTCACCATTCTGCCTTTACTGTCGCAGATCCCCGGCGTCAGCTTTAGCGATCAGGAAATTGCCGACCTGACTAAACGTATTCAGAACGCCGGTACCGAAGTCGTGGAAGCGAAAGCGGGCGGCGGGTCGGCGACCTTGTCGATGGGCCAGGCGGCTGCCCGTTTTGGTCTCTCTCTGGTTCGCGCCATGCA

>pgi

CTGCCGTACGACCAGTACATGCACCGCTTTGCCGCTTACTTCCAGCAGGGCAACATGGAGTCCAACGGTAAGTATGTTGACCGTAACGGCCACGCGGTAGACTACCAGACTGGCCCAATCATCTGGGGTGAGCCGGGCACCAACGGTCAGCACGCGTTCTACCAGCTGATCCACCAGGGCACCAAAATGGTACCGTGCGATTTCATCGCTCCGGCTATCACCCACAACCCGCTGTCTGACCACCATCAGAAACTGCTGTCTAACTTCTTCGCCCAGACCGAGGCCCTGGCCTTTGGTAAATCCCGCGAAGTGGTTGAGCAGGAATATCGCGATCAGGGTAAAGACCCGGCGACCCTGGAGCACGTGGTGCCGTTCAAAGTGTTCGAAGGTAACCGCCCGACTAACTCCATCCTGCTGCGTGAGATCACCCCGTTCAGCCTCGGGGCGCTGATTGCCCTGTACGAGCACAAAATCTTCACCCAGGGCGCGATCCTCAACATCTTCACCTTTGACCAGTGGGGCGTTGAGCTGGGCAAACAGCTGGCTAACCGC

>phoE

AGAACGGCGACGGCGTCGGCACCTCGTTAAGCTATGATTTCGGCGGCAGCGACTTCGCCGTCAGCGCAGCCTACACCAGCTCCGACCGTACCAACGATCAGAACCTGCTGGCCCGCGGCCAGGGTTCGAAAGCGGAAGCCTGGGCGACCGGCCTGAAATATGACGCCAACAATATCTACCTGGCGACCATGTACTCTGAAACCCGCAAGATGACCCCGATCAGCGGCGGCTTTGCCAACAAAGCGCAGAACTTTGAAGCGGTGGCGCAGTATCAGTTCGACTTCGGTCTGCGTCCGTCCCTCGGCTATGTGCTGTCGAAAGGGAAGGATATCGAAGGGGTGGGGAGTGAAGATCTGGTTAACTACATCGACGTGGGCCTGACCTACTACTTCAACAAAAACATGAACGCCTTCGTGGATTACAAAATCAACCAGCTGAAAAGCGATAACAAACTCGGCATCAACGATGACGACATCGTCGCGCTGGGTATCAC

>infB

CGGCATGATCACCTTCCTGGATACCCCGGGCCACGCCGCGTTTACCTCCATGCGTGCTCGTGGCGCGCAGGCGACGGATATCGTGGTTCTGGTGGTGGCGGCAGACGACGGCGTGATGCCGCAGACTATCGAAGCTATCCAGCACGCTAAAGCGGCGCAGGTACCGGTGGTAGTGGCGGTGAACAAGATCGATAAGCCAGAAGCCGATCCGGATCGCGTGAAGAACGAACTGTCCCAGTACGGCATCCTGCCGGAAGAGTGGGGCGGCGAGAGCCAGTTTGTCCACGTTTCCGCGAAAGCGGGTACCGGCATCGACGACCTGCTGGACGCGATCCTGCTGCAGGCTGAAGTT

>tonB

AATGAGCGTCTCGACGCAGCCGATAGAGATCACAATGGTGGCGCCGGCCGATCTTGAGCCGCCTCCGGCGGCGCAGCCTGTCGTGGAGCCCGTTGTTGAACCCGAACCTGAGCCGGAGCCAGAGGTAGCGCCTGAACCGCCGAAAGAGGCGCCGGTGGTGATCCATAAACCGGAACCTAAGCCGAAGCCCAAACCTAAACCCAAGCCTAAGCCGGAGAAAAAGGTTGAACAGCCGAAGCGGGAAGTGAAGCCGGCAGCAGAGCCGCGTCCGGCCTCGCCGTTTGAAAACAACAATACGGCGCCGGCGCGTACAGCGCCAAGTACCTCGACCGCAGCGGCTAAACCCACCGTTACTGCTCCGAGCGGCCCGCGGGCGATCAGCCGCGTTCAGCCGTCCTATCCGCCGCGCGCTCAGGCGCTGCGCATTGAAGGGACGGTACGGGTGAAGTTTGACGTTTCGCCTGATGGCCGCATTGATAATCTGCAGATCCTCTCTGTAGCCAGGGGGAAATAA

FO38 (ST11)

>rpoB

ATCGGTAGGTTGTAGGAGCGTGCGGTGAGAGCGTCTGTCTCTTGGCGATCTGGATACCCTGATGCCTCAGGATATGATCAACGCCAAGCCGATTTCCGCAGCAGTGAAAGAGTTCTTTGGTTCCAGCCAGCTGTCTCAGTTTATGGACCAGAACAACCCGCTGTCTGAGATTACGCACAAACGTCGTATCTCCGCACTCGGCCCAGGCGGTCTGACCCGTGAGCGCGCAGGCTTCGAAGTTCGAGACGTACACCCGACCCACTACGGTCGCGTATGTCCGATCGAAACGCCTGAAGGTCCGAACATCGGTCTGATTAACTCCCTGTCCGTGTACGCGCAGACCAACGAATATGGCTTCCTTGAGACGCCGTATCGTAAAGTGACCAACGGTGTGGTTACTGACGAAATTCACTACCTGTCTGCTATCGAAGAAGGCAACTACGTTATCGCTCAGGCGAACTCCAACCTGGATGAAAACGGCCACTTCGTAGAAGATCTGGTTACCTGCCGTAGCAAAGGCGAATCCAGCTTGTTCAGCCGCGACCAGGTTGACTACATGGACGTATCCACCCAGCAGGTGGTATCCGTCGGTGCGTCCCTGATCCCGTTCCTGGAACACGATGACGCCAACCGTGCATTGATGGGTGCGAACATGCAACGTCAGGCGGTTCCGACTCTGCGCGCTGATAAGCCGCTGGTTGGTACCGGTATGGAACGTGCTGTTGCCGTTGACTCCGGTGTTACTGCCGTGGCTAAACGTGGCGGTACCGTTCAGTACGTGGATGCTTCCCGTATCGTTATCAAAGTTAACGAAGACGAGATGTACCCGGGCGAAGCAGGTATCGACATCTATAACCTGACCAAGTACACCCGTTCTAACCAGAACACCTGCATCAACCAGATGCCTTGCGTGTCCCTGGGCGAACCTATTGAGCGCGGCGACGTGCTGGCAGACGGCCCGTCCACCGACCTCGGTGAGCTGGCGCTGGGTCAGAACATGCGTGTAGCGTTCATGCCGCG

>gapA

CGGTTTAAGATCGTTGAGTGAAGACGGTCATCTGGTCGTTAACGGTAAAAAAATCCGTGTTACCGCTGAACGTGACCCGGCTAACCTGAAGTGGGACGAAGTTGGTGTTGACGTTGTTGCTGAAGCAACCGGTATCTTCCTGACCGACGAAACCGCTCGTAAACACATCACCGCTGGCGCGAAAAAAGTCGTTCTGACTGGCCCGTCCAAAGACAACACTCCGATGTTCGTTCGCGGCGCTAACTTCGACGCTTACGCTGGCCAGGACATCGTTTCCAACGCTTCCTGCACCACTAACTGCCTGGCGCCGCTGGCTAAAGTTATCAACGACAACTTCGGTATCGTTGAAGGCCTGATGACCACCGTCCACGCTACCACCGCTACTCAGAAAACCGTTGATGGCCCGTCTCACAAAGACTGGCGCGGCGGCCGCGGCGCAGCTCAGAACATCATCCCGTCCTCTACCGGCGCTGCTAAAGCAGTAGGTAAAGTACTGCCAGAACTGAACGGCAAACTGACCGGTATGGCGTTCCGCGTTCCAACTCCGAACGTATCTGTTGTTGACCTGACCGTTCGTCTGGAAAAAGCAGCGTCCTACGAAGAAATCAAGAAAGCCATCAAAGCCCTTTTCTGAAG

>mdh

GCATTAGACTCGCTCGGTTACGCCGGGCGTGGCGGTAGATCTAAGTCATATCCCCACAGATGTAAAAATTAAAGGATTTTCCGGTGAAGACGCTACTCCGGCGCTGGAAGGCGCGGATGTAGTGCTGATCTCCGCGGGCGTGGCGCGTAAGCCCGGCATGGATCGTTCCGACCTGTTTAATGTGAATGCGGGTATCGTGAAGAACCTCGTGCAGCAGATTGCCAAAACCTGCCCGCAGGCCTGCATCGGCATTATCACCAACCCGGTGAATACCACCGTGGCTATCGCCGCCGAAGTACTGAAAAAAGCCGGCGTGTACGATAAAAACAAACTGTTCGGCGTTACCACGCTGGACATCATCCGTTCCAATACCTTTGTGGCGGAGCTGAAAGGTAAATCGGCAACCGAGGTGGAAGTCCCGGTCATTGGTGGTCACTCCGGGGTCACCATTCTGCCTTTACTGTCGCAGATCCCCGGCGTCAGCTTTAGCGATCAGGAAATTGCCGACCTGACTAAACGTATTCAGAACGCCGGTACCGAAGTCGTGGAAGCGAAAGCGGGCGGCGGGTCGGCGACCTTGTCGATGGGCCAGGCGGCTGCCCGTTTTGGTCTCTCTCTGGTTCGCGCCATGCAGGGGGAAAAAGGCGTGGTGGAGTGCGCCTACGTGGAAGGCGACGGCCACTATGCGCGTTTCTTCTCCCAGCCGCTGCTCGGGGAGAAAAAAACACGGAGA

>pgi

TGCCAGAAATAGTACAGCTGTCGTCGGCTGCTAACTTCGCTGCCGTCTTTCAgCTCCGGCAGGATGCGGTTAGCCAGCTGTTTGCCCAGCTCAACGCCCCACTGGTCAAAGGTGAAGATGTTGAGGATCGCGCCCTGGGTGAAGATTTTGTGCTCGTACAGGGCAATCAGCGCCCCGAGGCTGAACGGGGTGATCTCACGCAGCAGGATGGAGTTAGTCGGGCGGTTACCTTCGAACACTTTGAACGGCACCACGTGCTCCAGGGTCGCCGGGTCTTTACCCTGATCGCGATATTCCTGCTCAACCACTTCGCGGGATTTACCAAAGGCCAGGGCCTCGGTCTGGGCGAAGAAGTTAGACAGCAGTTTCTGATGGTGGTCAGACAGCGGGTTGTGGGTGATAGCCGGAGCGATGAAATCGCACGGTACCATTTTGGTGCCCTGGTGGATCAGCTGGTAGAACGCGTGCTGACCGTTGGTGCCCGGCTCACCCCAGATGATTGGGCCAGTCTGGTAGTCTACCGCGTGGCCGTTACGGTCAACATACTTACCGTTGGACTCCATGTTGCCCTGCTGGAAGTAAGCGGCAAAGCGGTGCATGTACTGGTCGTACGGCAGAATCGCTTCGGTTTCCGCACCGAAGAAGTTGTTGTACCAGATGCCGATCAGAGCCAGCAGTACAGGAGGTTTTTTTCCCACACA

>phoE

ACCGTAGCTCTCAGTATGAGATCTGACCCTGCAGTACCAGGGTAAAAACGAAGGCCGTGAAGCGAAGAAACAGAACGGCGACGGCGTCGGCACCTCGTTAAGCTATGATTTCGGCGGCAGCGACTTCGCCGTCAGCGCAGCCTACACCAGCTCCGACCGTACCAACGATCAGAACCTGCTGGCCCGCGGCCAGGGTTCGAAAGCGGAAGCCTGGGCGACCGGCCTGAAATATGACGCCAACAATATCTACCTGGCGACCATGTACTCTGAAACCCGCAAGATGACCCCGATCAGCGGCGGCTTTGCCAACAAAGCGCAGAACTTTGAAGCGGTGGCGCAGTATCAGTTCGACTTCGGTCTGCGTCCGTCCCTCGGCTATGTGCTGTCGAAAGGGAAGGATATCGAAGGGGTGGGGAGTGAAGATCTGGTTAACTACATCGACGTGGGCCTGACCTACTACTTCAACAAAAACATGAACGCCTTCGTGGATTACAAAATCAACCAGCTGAAAAGCGATAACAAACTCGGCATCAACGATGACGACATCGTCGCGCTGGGTATCACCTACCTTTTTCTGGATCAAA

>infB

GACCAAAAAGTGCTCGGCGAGCGGGTGGTATTACCCAGCACATCGGTGCTTACCACGTCGAAACCGACAACGGCATGATCACCTTCCTGGATACCCCGGGCCACGCCGCGTTTACCTCCATGCGTGCTCGTGGCGCGCAGGCGACGGATATCGTGGTTCTGGTGGTGGCGGCAGACGACGGCGTGATGCCGCAGACTATCGAAGCTATCCAGCACGCTAAAGCGGCGCAGGTACCGGTGGTAGTGGCGGTGAACAAGATCGATAAGCCAGAAGCCGATCCGGATCGCGTGAAGAACGAACTGTCCCAGTACGGCATCCTGCCGGAAGAGTGGGGCGGCGAGAGCCAGTTTGTCCACGTTTCCGCGAAAGCGGGTACCGGCATCGACGACCTGCTGGACGCGATCCTGCTGCAGGCTGAAGTTCTTGACCTGAAAGCGTT

>tonB

AGGTTGGGCATCTCGACGCAGCCGATAGAGATCACAATGGTGGCGCCGGCCGATCTTGAGCCGCCTCCGGCGGCGCAGCCTGTCGTGGAGCCCGTTGTTGAACCCGAACCTGAGCCGGAGCCAGAGGTAGCGCCTGAACCGCCGAAAGAGGCGCCGGTGGTGATCCATAAACCGGAACCTAAGCCGAAGCCCAAACCTAAACCCAAGCCTAAGCCGGAGAAAAAGGTTGAACAGCCGAAGCGGGAAGTGAAGCCGGCAGCAGAGCCGCGTCCGGCCTCGCCGTTTGAAAACAACAATACGGCGCCGGCGCGTACAGCGCCAAGTACCTCGACCGCAGCGGCTAAACCCACCGTTACTGCTCCGAGCGGCCCGCGGGCGATCAGCCGCGTTCAGCCGTCCTATCCGCCGCGCGCTCAGGCGCTGCGCATTGAAGGGACGGTACGGGTGAAGTTTGACGTTTCGCCTGATGGCCGCATTGATAATCTGCAGATCCTCTCTGCTCAGCCGGGGCGAATCT

FO39 (ST11)

>rpoB

ATGCTGCTGGCGTGTAGAGCGTGCGGTGATGAGCGTCTGTCTCTTGGCGATCTGGATACCCTGATGCCTCAGGATATGATCAACGCCAAGCCGATTTCCGCAGCAGTGAAAGAGTTCTTTGGTTCCAGCCAGCTGTCTCAGTTTATGGACCAGAACAACCCGCTGTCTGAGATTACGCACAAACGTCGTATCTCCGCACTCGGCCCAGGCGGTCTGACCCGTGAGCGCGCAGGCTTCGAAGTTCGAGACGTACACCCGACCCACTACGGTCGCGTATGTCCGATCGAAACGCCTGAAGGTCCGAACATCGGTCTGATTAACTCCCTGTCCGTGTACGCGCAGACCAACGAATATGGCTTCCTTGAGACGCCGTATCGTAAAGTGACCAACGGTGTGGTTACTGACGAAATTCACTACCTGTCTGCTATCGAAGAAGGCAACTACGTTATCGCTCAGGCGAACTCCAACCTGGATGAAAACGGCCACTTCGTAGAAGATCTGGTTACCTGCCGTAGCAAAGGCGAATCCAGCTTGTTCAGCCGCGACCAGGTTGACTACATGGACGTATCCACCCAGCAGGTGGTATCCGTCGGTGCGTCCCTGATCCCGTTCCTGGAACACGATGACGCCAACCGTGCATTGATGGGTGCGAACATGCAACGTCAGGCGGTTCCGACTCTGCGCGCTGATAAGCCGCTGGTTGGTACCGGTATGGAACGTGCTGTTGCCGTTGACTCCGGTGTTACTGCCGTGGCTAAACGTGGCGGTACCGTTCAGTACGTGGATGCTTCCCGTATCGTTATCAAAGTTAACGAAGACGAGATGTACCCGGGCGAAGCAGTATCGACATCTATAACCTGACCAAGTACACCCGTTCTAACCAGAACACCTGCATCAACCAGATGCCTTGCGTGTCCCTGGGCGAACCTATTGAGCGCGGCGACGTGCTGGCAGACGGCCCGTCCACCGACCTCGGTGAGCTGGCGCTGGGTCAGAACATGCGTGTAGCGTTCATGCCGTGGAACGGTATTCTTCTTGGC

>gapA

GGCATCCAGTCGTTGAGTGAaGACGGTCATCTGGTCGTTAACGGTAAAAAAATCCGTGTTACCGCTGAACGTGACCCGGCTAACCTGAAGTGGGACGAAGTTGGTGTTGACGTTGTTGCTGAAGCAACCGGTATCTTCCTGACCGACGAAACCGCTCGTAAACACATCACCGCTGGCGCGAAAAAAGTCGTTCTGACTGGCCCGTCCAAAGACAACACTCCGATGTTCGTTCGCGGCGCTAACTTCGACGCTTACGCTGGCCAGGACATCGTTTCCAACGCTTCCTGCACCACTAACTGCCTGGCGCCGCTGGCTAAAGTTATCAACGACAACTTCGGTATCGTTGAAGGCCTGATGACCACCGTCCACGCTACCACCGCTACTCAGAAAACCGTTGATGGCCCGTCTCACAAAGACTGGCGCGGCGGCCGCGGCGCAGCTCAGAACATCATCCCGTCCTCTACCGGCGCTGCTAAAGCAGTAGGTAAAGTACTGCCAGAACTGAACGGCAAACTGACCGGTATGGCGTTCCGCGTTCCAACTCCGAACGTATCTGTTGTTGACCTGACCGTTCGTCTGGAAAAAGCAGCGTCCTACGAAGAAATCAAGAAAGCCATCAAAGACTCTCTTTCTCGAAGAGA

>mdh

CACCGACGTCGATCGCTCGGTTACGCCGGGCGTGGCGGTAGATCTAAGTCATATCCCCACAGATGTAAAAATTAAAGGATTTTCCGGTGAAGACGCTACTCCGGCGCTGGAAGGCGCGGATGTAGTGCTGATCTCCGCGGGCGTGGCGCGTAAGCCCGGCATGGATCGTTCCGACCTGTTTAATGTGAATGCGGGTATCGTGAAGAACCTCGTGCAGCAGATTGCCAAAACCTGCCCGCAGGCCTGCATCGGCATTATCACCAACCCGGTGAATACCACCGTGGCTATCGCCGCCGAAGTACTGAAAAAAGCCGGCGTGTACGATAAAAACAAACTGTTCGGCGTTACCACGCTGGACATCATCCGTTCCAATACCTTTGTGGCGGAGCTGAAAGGTAAATCGGCAACCGAGGTGGAAGTCCCGGTCATTGGTGGTCACTCCGGGGTCACCATTCTGCCTTTACTGTCGCAGATCCCCGGCGTCAGCTTTAGCGATCAGGAAATTGCCGACCTGACTAAACGTATTCAGAACGCCGGTACCGAAGTCGTGGAAGCGAAAGCGGGCGGCGGGTCGGCGACCTTGTCGATGGGCCAGGCGGCTGCCCGTTTTGGTCTCTCTCTGGTTCGCGCCATGCAGGGGGAAAAAGGCGTGGTGGAGTGCGCCTACGTGGAAGGCGACGGCCACTATGCGCGTTTCTTCTCCCAGCCGCTGCTGCAGAGAAAAAAAAACGAA

>pgi

CTCTGTGCGGTTAGCTCTGGTCACACTTCTTCGGTGCGGAACCGAAGCGATTCTGCCGTACGACCAGTACATGCACCGCTTTGCCGCTTACTTCCAGCAGGGCAACATGGAGTCCAACGGTAAGTATGTTGACCGTAACGGCCACGCGGTAGACTACCAGACTGGCCCAATCATCTGGGGTGAGCCGGGCACCAACGGTCAGCACGCGTTCTACCAGCTGATCCACCAGGGCACCAAAATGGTACCGTGCGATTTCATCGCTCCGGCTATCACCCACAACCCGCTGTCTGACCACCATCAGAAACTGCTGTCTAACTTCTTCGCCCAGACCGAGGCCCTGGCCTTTGGTAAATCCCGCGAAGTGGTTGAGCAGGAATATCGCGATCAGGGTAAAGACCCGGCGACCCTGGAGCACGTGGTGCCGTTCAAAGTGTTCGAAGGTAACCGCCCGACTAACTCCATCCTGCTGCGTGAGATCACCCCGTTCAGCCTCGGGGCGCTGATTGCCCTGTACGAGCACAAAATCTTCACCCAGGGCGCGATCCTCAACATCTTCACCTTTGACCAGTGGGGCGTTGAGCTGGGCAAACAGCTGGCTAACCGCATCCTGCCGGAGCTGAAAGACGGCAGCGAAGTTAGCAGCCACGACAGCTCTACTAACGGCCTGATTAACCGCTAAACGGGGGGGGGCGGGAGA

>phoE

GTACTAGCTCTCAGTCTGCATCTGACCTGCAGTACCAGGGTAAAACGAAGGCCGTGAAGCGAAGAAACAGAACGGCGACGGCGTCGGCACCTCGTTAAGCTATGATTTCGGCGGCAGCGACTTCGCCGTCAGCGCAGCCTACACCAGCTCCGACCGTACCAACGATCAGAACCTGCTGGCCCGCGGCCAGGGTTCGAAAGCGGAAGCCTGGGCGACCGGCCTGAAATATGACGCCAACAATATCTACCTGGCGACCATGTACTCTGAAACCCGCAAGATGACCCCGATCAGCGGCGGCTTTGCCAACAAAGCGCAGAACTTTGAAGCGGTGGCGCAGTATCAGTTCGACTTCGGTCTGCGTCCGTCCCTCGGCTATGTGCTGTCGAAAGGGAAGGATATCGAAGGGGTGGGGAGTGAAGATCTGGTTAACTACATCGACGTGGGCCTGACCTACTACTTCAACAAAAACATGAACGCCTTCGTGGATTACAAAATCAACCAGCTGAAAAGCGATAACAAACTCGGCATCAACGATGACGACATCGTCGCGCTGGGTATCACCTACTTTTTTTTGATATCAA

>infB

GTAAGTTTGCTCGGCGAGCGGGTGGTATTACCCAGCACATCGGTGCTTACCACGTCGAAACCGACAACGGCATGATCACCTTCCTGGATACCCCGGGCCACGCCGCGTTTACCTCCATGCGTGCTCGTGGCGCGCAGGCGACGGATATCGTGGTTCTGGTGGTGGCGGCAGACGACGGCGTGATGCCGCAGACTATCGAAGCTATCCAGCACGCTAAAGCGGCGCAGGTACCGGTGGTAGTGGCGGTGAACAAGATCGATAAGCCAGAAGCCGATCCGGATCGCGTGAAGAACGAACTGTCCCAGTACGGCATCCTGCCGGAAGAGTGGGGCGGCGAGAGCCAGTTTGTCCACGTTTCCGCGAAAGCGGGTACCGGCATCGACGACCTGCTGGACGCGATCCTGCTGCAGGCTGAAGTTCTTGCCTTTTGAAAGCGA

>tonB

GGGAAGCATCTCGACGCAGCCGATAGAGATCACAATGGTGGCGCCGGCCGATCTTGAGCCGCCTCCGGCGGCGCAGCCTGTCGTGGAGCCCGTTGTTGAACCCGAACCTGAGCCGGAGCCAGAGGTAGCGCCTGAACCGCCGAAAGAGGCGCCGGTGGTGATCCATAAACCGGAACCTAAGCCGAAGCCCAAACCTAAACCCAAGCCTAAGCCGGAGAAAAAGGTTGAACAGCCGAAGCGGGAAGTGAAGCCGGCAGCAGAGCCGCGTCCGGCCTCGCCGTTTGAAAACAACAATACGGCGCCGGCGCGTACAGCGCCAAGTACCTCGACCGCAGCGGCTAAACCCACCGTTACTGCTCCGAGCGGCCCGCGGGCGATCAGCCGCGTTCAGCCGTCCTATCCGCCGCGCGCTCAGGCGCTGCGCATTGAAGGGACGGTACGGGTGAAGTTTGACGTTTCGCCTGATGGCCGCATTGATAATCTGCAGATCCTCTCTGCTCGCCGGGGGCGAATAAA

FO40 (ST11)

>rpoB

CGGGGGAACGTGGTAGGAGCGTGCGGTGAaGAGCGTCTGTCTCTTGGCGATCTGGATACCCTGATGCCTCAGGATATGATCAACGCCAAGCCGATTTCCGCAGCAGTGAAAGAGTTCTTTGGTTCCAGCCAGCTGTCTCAGTTTATGGACCAGAACAACCCGCTGTCTGAGATTACGCACAAACGTCGTATCTCCGCACTCGGCCCAGGCGGTCTGACCCGTGAGCGCGCAGGCTTCGAAGTTCGAGACGTACACCCGACCCACTACGGTCGCGTATGTCCGATCGAAACGCCTGAAGGTCCGAACATCGGTCTGATTAACTCCCTGTCCGTGTACGCGCAGACCAACGAATATGGCTTCCTTGAGACGCCGTATCGTAAAGTGACCAACGGTGTGGTTACTGACGAAATTCACTACCTGTCTGCTATCGAAGAAGGCAACTACGTTATCGCTCAGGCGAACTCCAACCTGGATGAAAACGGCCACTTCGTAGAAGATCTGGTTACCTGCCGTAGCAAAGGCGAATCCAGCTTGTTCAGCCGCGACCAGGTTGACTACATGGACGTATCCACCCAGCAGGTGGTATCCGTCGGTGCGTCCCTGATCCCGTTCCTGGAACACGATGACGCCAACCGTGCATTGATGGGTGCGAACATGCAACGTCAGGCGGTTCCGACTCTGCGCGCTGATAAGCCGCTGGTTGGTACCGGTATGGAACGTGCTGTTGCCGTTGACTCCGGTGTTACTGCCGTGGCTAAACGTGGCGGTACCGTTCAGTACGTGGATGCTTCCCGTATCGTTATCAAAGTTAACGAAGACGAGATGTACCCGGGCGAAGCAGTATCGACATCTATAACCTGACCAAGTACACCCGTTCTAACCAGAACACCTGCATCAACCAGATGCCTTGCGTGTCCCTGGGCGAACCTATTGAGCGCGGCGACGTGCTGGCAGACGGCCCGTCCACCGACCTCGGTGAGCTGGCGCTGGGTTCAGAAACATGCGTGTAGCGTTC

>gapA

CATCTGGTCGTTAACGGTAAAAAAATCCGTGTTACCGCTGAACGTGACCCGGCTAACCTGAAGTGGGACGAAGTTGGTGTTGACGTTGTTGCTGAAGCAACCGGTATCTTCCTGACCGACGAAACCGCTCGTAAACACATCACCGCTGGCGCGAAAAAAGTCGTTCTGACTGGCCCGTCCAAAGACAACACTCCGATGTTCGTTCGCGGCGCTAACTTCGACGCTTACGCTGGCCAGGACATCGTTTCCAACGCTTCCTGCACCACTAACTGCCTGGCGCCGCTGGCTAAAGTTATCAACGACAACTTCGGTATCGTTGAAGGCCTGATGACCACCGTCCACGCTACCACCGCTACTCAGAAAACCGTTGATGGCCCGTCTCACAAAGACTGGCGCGGCGGCCGCGGCGCAGCTCAGAACATCATCCCGTCCTCTACCGGCGCTGCTAAAGCAGTAGGTAAAGTACTGCCAGAACTGAACGGCAAACTGACCGGTATGGCGTTCCGCGTTCCAACTCCGAACGTATCTGTTGTTGACCTGACCGTTCGTCTGGAAAAAGCAGCGTCCTA

>mdh

CTAAGTCATATCCCCACAGATGTAAAAATTAAAGGATTTTCCGGTGAAGACGCTACTCCGGCGCTGGAAGGCGCGGATGTAGTGCTGATCTCCGCGGGCGTGGCGCGTAAGCCCGGCATGGATCGTTCCGACCTGTTTAATGTGAATGCGGGTATCGTGAAGAACCTCGTGCAGCAGATTGCCAAAACCTGCCCGCAGGCCTGCATCGGCATTATCACCAACCCGGTGAATACCACCGTGGCTATCGCCGCCGAAGTACTGAAAAAAGCCGGCGTGTACGATAAAAACAAACTGTTCGGCGTTACCACGCTGGACATCATCCGTTCCAATACCTTTGTGGCGGAGCTGAAAGGTAAATCGGCAACCGAGGTGGAAGTCCCGGTCATTGGTGGTCACTCCGGGGTCACCATTCTGCCTTTACTGTCGCAGATCCCCGGCGTCAGCTTTAGCGATCAGGAAATTGCCGACCTGACTAAACGTATTCAGAACGCCGGTACCGAAGTCGTGGAAGCGAAAGCGGGCGGCGGGTCGGCGACCTTGTCGATGGGCCAGGCGGCTGCCCGTTTTGGTCTCTCTCTGGTTCGCGCCATGCAGGGGGAAAAAGGCGTGGTGGAGTGCGCCTACGTGGAAGGCGACGGCCACTATGCGCGTTTCTTCTCCCAGCCGCTGCTGCTG

>pgi

CGATTCTGCCGTACGACCAGTACATGCACCGCTTTGCCGCTTACTTCCAGCAGGGCAACATGGAGTCCAACGGTAAGTATGTTGACCGTAACGGCCACGCGGTAGACTACCAGACTGGCCCAATCATCTGGGGTGAGCCGGGCACCAACGGTCAGCACGCGTTCTACCAGCTGATCCACCAGGGCACCAAAATGGTACCGTGCGATTTCATCGCTCCGGCTATCACCCACAACCCGCTGTCTGACCACCATCAGAAACTGCTGTCTAACTTCTTCGCCCAGACCGAGGCCCTGGCCTTTGGTAAATCCCGCGAAGTGGTTGAGCAGGAATATCGCGATCAGGGTAAAGACCCGGCGACCCTGGAGCACGTGGTGCCGTTCAAAGTGTTCGAAGGTAACCGCCCGACTAACTCCATCCTGCTGCGTGAGATCACCCCGTTCAGCCTCGGGGCGCTGATTGCCCTGTACGAGCACAAAATCTTCACCCAGGGCGCGATCCTCAACATCTTCACCTTTGACCAGTGGGGCGTTGAGCTGGGCAAACAGCTGGCTAACCGCATCCTGCCGGAGCTGAAAGACGGCAGCGAAGTTAGCAGCCACGACAGCTCTACTAACGGCCTGATTAACCGCTAT

>phoE

GGCGACGGCGTCGGCACCTCGTTAAGCTATGATTTCGGCGGCAGCGACTTCGCCGTCAGCGCAGCCTACACCAGCTCCGACCGTACCAACGATCAGAACCTGCTGGCCCGCGGCCAGGGTTCGAAAGCGGAAGCCTGGGCGACCGGCCTGAAATATGACGCCAACAATATCTACCTGGCGACCATGTACTCTGAAACCCGCAAGATGACCCCGATCAGCGGCGGCTTTGCCAACAAAGCGCAGAACTTTGAAGCGGTGGCGCAGTATCAGTTCGACTTCGGTCTGCGTCCGTCCCTCGGCTATGTGCTGTCGAAAGGGAAGGATATCGAAGGGGTGGGGAGTGAAGATCTGGTTAACTACATCGACGTGGGCCTGACCTACTACTTCAACAAAAACATGAACGCCTTCGTGGATTACAAAATCAACCAGCTGAAAAGCGATAACAAACTCGGCATCAACGATGACGACATCGTCGCGCTGGGTATCACCTACCA

>infB

CACATCGGTGCTTACCACGTCGAAACCGACAACGGCATGATCACCTTCCTGGATACCCCGGGCCACGCCGCGTTTACCTCCATGCGTGCTCGTGGCGCGCAGGCGACGGATATCGTGGTTCTGGTGGTGGCGGCAGACGACGGCGTGATGCCGCAGACTATCGAAGCTATCCAGCACGCTAAAGCGGCGCAGGTACCGGTGGTAGTGGCGGTGAACAAGATCGATAAGCCAGAAGCCGATCCGGATCGCGTGAAGAACGAACTGTCCCAGTACGGCATCCTGCCGGAAGAGTGGGGCGGCGAGAGCCAGTTTGTCCACGTTTCCGCGAAAGCGGGTACCGGCATCGACGACCTGCTGGACGCGATCCTGCTGCAGGCTGAAGTTCTT

>tonB

AGATCCCCATTCTCGGACGCAGCCGATAGAGATCACAATGGTGGCGCCGGCCGATCTTGAGCCGCCTCCGGCGGCGCAGCCTGTCGTGGAGCCCGTTGTTGAACCCGAACCTGAGCCGGAGCCAGAGGTAGCGCCTGAACCGCCGAAAGAGGCGCCGGTGGTGATCCATAAACCGGAACCTAAGCCGAAGCCCAAACCTAAACCCAAGCCTAAGCCGGAGAAAAAGGTTGAACAGCCGAAGCGGGAAGTGAAGCCGGCAGCAGAGCCGCGTCCGGCCTCGCCGTTTGAAAACAACAATACGGCGCCGGCGCGTACAGCGCCAAGTACCTCGACCGCAGCGGCTAAACCCACCGTTACTGCTCCGAGCGGCCCGCGGGCGATCAGCCGCGTTCAGCCGTCCTATCCGCCGCGCGCTCAGGCGCTGCGCATTGAAGGGACGGTACGGGTGAAGTTTGACGTTTCGCCTGATGGCCGCATTGATAATCTGCAGATCCTCTCTGCTCAGCCCGGCGAATAA
